# A role for myosin II clusters and membrane energy in cortex rupture for *Dictyostelium discoideum*

**DOI:** 10.1371/journal.pone.0265380

**Published:** 2022-04-25

**Authors:** Emmanuel Asante-Asamani, Daniel Grange, Devarshi Rawal, Zully Santiago, John Loustau, Derrick Brazill

**Affiliations:** 1 Department of Mathematics, Clarkson University, Potsdam, New York, United States of America; 2 Department of Applied Mathematics, Stony Brook University, New York, New York, United States of America; 3 Mathematics and Statistics Department, Hunter College, Manhattan, New York, United States of America; 4 Department of Natural Science, Baruch College, New York, New York, United States of America; 5 Biological Science Department, Hunter College, Manhattan, New York, United States of America; University of Illinois at Chicago, UNITED STATES

## Abstract

Blebs, pressure driven protrusions of the cell membrane, facilitate the movement of eukaryotic cells such as the soil amoeba *Dictyostelium discoideum*, white blood cells and cancer cells. Blebs initiate when the cell membrane separates from the underlying cortex. A local rupture of the cortex, has been suggested as a mechanism by which blebs are initiated. However, much clarity is still needed about how cells inherently regulate rupture of the cortex in locations where blebs are expected to form. In this work, we examine the role of membrane energy and the motor protein myosin II (myosin) in facilitating the cell driven rupture of the cortex. We perform under-agarose chemotaxis experiments, using *Dictyostelium discoideum* cells, to visualize the dynamics of myosin and calculate changes in membrane energy in the blebbing region. To facilitate a rapid detection of blebs and analysis of the energy and myosin distribution at the cell front, we introduce an autonomous bleb detection algorithm that takes in discrete cell boundaries and returns the coordinate location of blebs with its shape characteristics. We are able to identify by microscopy naturally occurring gaps in the cortex prior to membrane detachment at sites of bleb nucleation. These gaps form at positions calculated to have high membrane energy, and are associated with areas of myosin enrichment. Myosin is also shown to accumulate in the cortex prior to bleb initiation and just before the complete disassembly of the cortex. Together our findings provide direct spatial and temporal evidence to support cortex rupture as an intrinsic bleb initiation mechanism and suggests that myosin clusters are associated with regions of high membrane energy where its contractile activity leads to a rupture of the cortex at points of maximal energy.

## Introduction

Chemotaxis, or chemically directed cell motility, is important for a variety of eukaryotic biological processes such as the development of an embryo, the search for pathogens by neutrophils, organ patterning, migration of fibroblasts to heal wounds and invasion of surrounding tissues by cancer cells [1–4]. Traditionally, chemotaxis has been facilitated by three actin-based motility structures filopodia, pseudopodia and lamellipodia [5]. These motility structures gained prominence in part because cells were predominantly observed while moving on a culture dish or crawling freely on the surface of a slide, all representative of a two-dimensional (2D) environment with little to no mechanical resistance or environmental compression. However, in their native environments, these cells often have to overcome significant mechanical resistance as they crawl through dense tissues in a three dimensional space (3D).

Recent observations of chemotaxis in human cancer cells [6–8], zebrafish primordial germ cells [9] and *D. discoideum* cells [10, 11], in compressive environments such as imposed by a 3D collagen matrix, agarose gel or a microfluidic channel respectively, have confirmed the use of a fourth motility structure, the bleb. Blebs are pressure driven, blister-like protrusions of the cell membrane. They are characterized by an initial detachment of the cell membrane from the cortex (nucleation), an expansion of the membrane into a spherical cap due to the force of flowing cytosol, a disintegration of the old cortex (actin scar) and a stabilization of the protrusion by formation of a new cortex directly beneath it [1, 12–14].

Blebs have been studied from several viewpoints. They have been described as geometric objects (Euclidean) [15], the result of forces on a smooth manifold (differential geometry) [10, 16–18], the result of physical force (pressure and cortical tension) [11, 19–22], the result of fluid dynamics [23, 24]. In spite of the substantial progress made in the study of blebbing, the precise mechanism by which cytoskeletal proteins interact with biophysical and geometric forces to generate blebs and coordinate cell movement using these protrusions is still unclear. As a first step towards a comprehensive understanding of bleb-based motility we are interested in clarifying the interaction between cytoskeletal proteins and biophysical forces leading to bleb nucleation. We take advantage of the genetic simplicity, ease of maintenance and chemotactic ability of *Dictyostelium discoideum* [25], a soil living amoeba, to study nucleation of blebs during chemotaxis.

Most researchers agree that blebs nucleate when a small patch of membrane is detached from the cortex [1, 13]. What differs among the theories of nucleation is the mechanism by which the plasma membrane detaches. One mechanism suggests that a local increase in cytoplasmic pressure, driven in part by local acto-myosin contraction results in the initial detachment of adhesion proteins (linker proteins) linking the cortex to the membrane [19, 21, 26]. Alternately, it’s been proposed that a local weakening or rupture of the actin cortex can initiate the formation of blebs [27–29]. At these locations of cortex rupture, the membrane no longer has the support of the cortex. The force of cytoplasmic flow is then capable of breaking the surrounding adhesive bonds and detaching a significant portion of the membrane to form a bleb. Both mechanisms have been supported by micropipette aspiration of the membrane and laser ablation of a local region of the cortex, all of which led to the formation of blebs [22, 30].

Outside of experimental intervention, it is unclear if cells naturally rupture their cortex to make blebs and which forces and proteins they use. Blebs induced in HT1080 fibrosarcoma cells by inhibiting the Arp2/3 complex showed signs of a weakened cytoskeleton, mostly at the base of filopodia, where blebs preferentially formed [29]. However, it is unclear whether blebbing cells with uninhibited Arp2/3 complex can generate the level of cortex weakening observed. Myosin II (myosin) has been shown to be necessary for bleb formation in both mammalian [13] and *D. discoideum* [14] cells. Its contractile force has been suggested to initiate blebs by locally elevating fluid pressure or rupturing the cortex in the cell front [1, 26], expand blebs by building global cytoplasmic pressure [11, 22], and retract expanding blebs [29]. In Zebrafish premordial germ cells, blebs were shown to form in regions of elevated calcium ions at the cell front, where myosin contraction was suspected to be elevated [9]. Myosin was also observed to be enriched at the base of early-stage blebs in HT1080 fibrosarcoma cells [29]. However, experimental evidence that directly correlates myosin and gap formation in naturally blebbing cells remains to be shown.

The mechanism by which cells select positions on their periphery to bleb is still not fully understood. Mathematical models have been extremely useful in elucidating the mechanism at play by clarifying the forces influencing bleb nucleation [18, 31, 32]. For instance, the distribution of linker proteins modeled as elastic springs able to generate tension on the cell periphery has been shown to play a critical role in localizing blebs to the front of the cell [18, 31, 32]. Cell boundary curvature has also been suggested to aid in the detachment of the membrane from the cortex through the outward component of membrane tension generated at locations of negative curvature [16]. However, it is unclear if such locations of high negative curvature correlate with sites of gap formation.

In this study, we provide experimental evidence to support the formation of gaps at nucleation sites of blebbing *Dictyostelium discoideum* cells crawling under the compressive force of a layer of agarose. To help clarify the mechanism for the formation and location of these gaps, we examine the dynamics of myosin at the front of the cell and use a mathematical model of free-energy to examine a role for membrane energy in gap formation. A bleb detection algorithm is introduced to ease the data processing workload by autonomously detecting blebs from microscopy images. Shape characteristics such as curvature, shoulder points (end points of the bleb) and a reasonable location of initial membrane detachment (furthest extent marker) are all computed by the algorithm for further analysis. Our experiments and theoretical model correlate myosin clusters and a pre-nucleation accumulation of myosin in the actin scar with the formation of cortex gaps at locations of maximum membrane energy.

## Results

### An algorithm for bleb detection and boundary digitization

**Fig 1:**
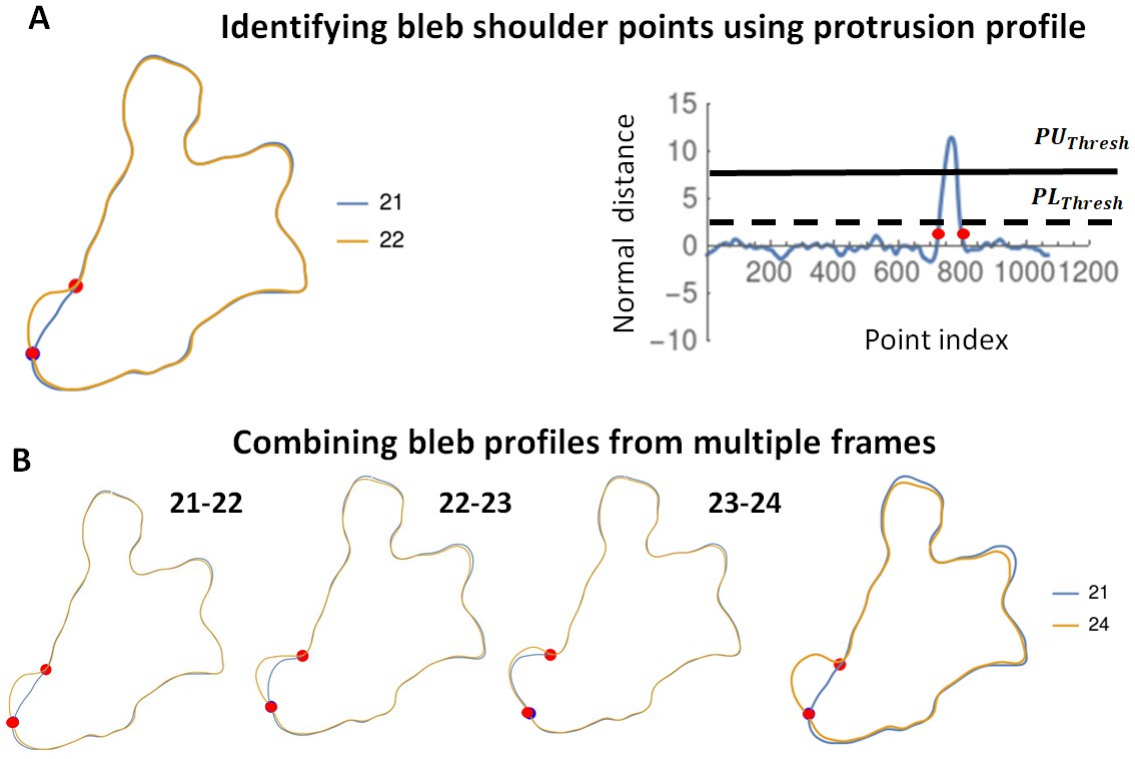
Computational scheme used to detect blebs from cell boundary profiles. A) Two successive boundaries (frame 21 and 22) of a chemotaxing *D. discoideum* cell blebbing under a layer of agarose are shown (left image) with the prior cell in blue and post cell in orange. The red dots indicate the shoulder points of the bleb. The normal displacement between consecutive cell boundaries is used to generate a protrusion profile (right image) from which the bleb shoulder points (red dots) are identified. The two horizontal lines denote the *PU*_*thresh*_(upper) and *PL*_*thresh*_(lower). B) Successive frames over which a bleb expands are combined to obtain the complete bleb shape.

The manual detection of blebs and extraction of their boundary points for energy calculations tends to be a very time consuming process using available software. In our application, we imaged cells for about 30 seconds at an average frame rate of 1.66 seconds. This generated between 18 and 20 frames per cell translating into a total of 700 frames for 35 cells. To reduce the time and energy required to identify blebs as well as improve the accuracy of measurements made on those blebs, we developed a bleb detection program to automatically find and process blebs from a sequence of cell boundary points (Fig 1). Details about the algorithm and the improvements we made on our existing image processing workflow [33] are presented in the Methods section.

### Cortex gaps form at bleb nucleation sites

**Fig 2:**
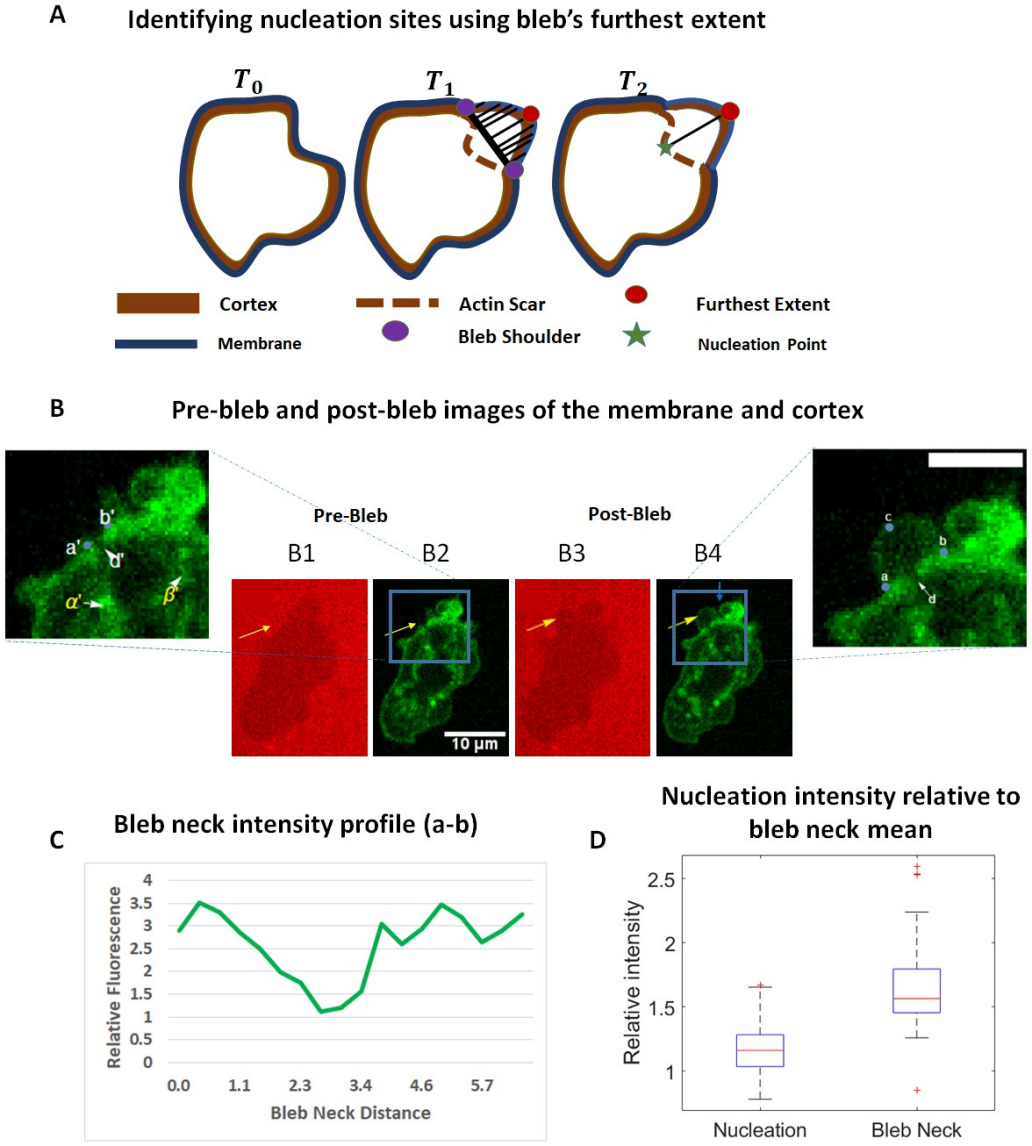
The furthest extent of the bleb identifies nucleation sites. A) A cartoon of a cell before bleb initiation (prior cell, *T*_0_) initiates and stabilizes a bleb at time *T*_1_ (post cell). The perpendicular distance between points on the fully formed bleb and a line segment connecting the bleb shoulder points (bleb neck) is used to determine the furthest extent as shown by black lines. In *T*_2_ the furthest extent is projected onto the actin scar to identify the nucleation point. B) Pre-bleb (B1,B2) and post-bleb (B3,B4) images of a chemotaxing *D. discoideum* cell expressing lifeAct GFP with membrane images (B1,B3) and cortex images (B2,B4). Enlarged portions of the cortex images are shown to clearly identify the location of the gap. The yellow arrows point to the location of the bleb. The locations *α*′ and *β*′ in the pre-bleb image point to actin structures that are also present in the post-bleb images. In the post-bleb image, the furthest extent, location *c*, is applied to identify nucleation site, location *d*, where a visible gap is seen. The shoulder points are identified by *a*, *b*. Scale bar is 5 um. C) The relative intensity of F-actin in the actin scar of the post-bleb image is shown. The gap here corresponds to a local minimum of 1.1. D) Local minima of intensity measurements at the nucleation sites for 39 blebs is compared with the average intensity at the bleb neck. Difference in medians is significant (Kruskal-Wallis test, *p* − *value* < 0.001).

Bleb initiation sites correspond to locations on the actin scar where the membrane first detached from the cortex. We constructed a geometric marker that uses the shape of the stabilized bleb, obtained from our bleb detection algorithm, to identify where the bleb most likely first detached from the cortex. Since bleb expansion is driven primarily by fluid flow, we reasoned that the location on the newly formed bleb boundary, furthest from the actin scar, would be contained in the membrane patch that initially detached from the cortex at nucleation. We refer to this location as the furthest extent of the bleb. To determine the nucleation site, we projected the furthest extent of the bleb perpendicularly onto the actin scar (Fig 2A). We subsequently examined these locations and neighboring regions for any signs of cortex rupture prior to membrane detachment for *D. discoidium* cells compressed by a layer of agarose.

Using the furthest extent nucleation indicator, we were able to visually detect nucleation points for 109 blebs out of the 142 examined (S1 File, S1 Data). Failure to detect the nucleation site was usually due to a very poor image of the stabilized bleb. As shown in Fig 2B4 (enlarged), the furthest extent of the bleb *c* projects perpendicularly unto the initiation site *d*. This initiation site corresponds with the location in the pre-bleb cortex image (Fig 2B2) where a distinct gap is seen as well as in the enlarged portion marked as *d*′. The gap is also visible in the relative intensity profile (cortex to cytoplasm) of the scar in Fig 2C where the local minimum at the gap is 1.1, which is almost at the intensity of the cytoplasm. It is noteworthy that this gap is already present before the membrane first detached from the cortex (Fig 2B1). The presence of the visible gap in the pre-bleb cortex image, before any sign of membrane detachment, distinguishes it from normal cortex disassembly during bleb stabilization and associates it with bleb initiation. We measured the relative intensity at the nucleation site for 39 blebs, using the frame with the clearest image of the gap, and compared it with the mean intensity of the actin scar. Our results in Fig 2D show a significantly lower nucleation site actin intensity compared with the mean intensity of the cortex (S1 Data, Kruskall-Wallis Test: p-value < 0.001). The median actin intensity at the nucleation sites was 1.2 which is close the local minimum intensity observed at the gap in (Fig 2C). These results support the existence of gaps or significant weakening of the cortex at nucleation sites.

### Cortex gaps form at maximal energy locations within the bleb neck

When under compression forces, such as experienced during multicellular development or under a uniform block of agarose gel, *D. discoideum* cells flatten and can be considered, for modeling simplicity, as two dimensional objects with a one dimensional boundary [16, 18, 34]. Any shape has an energy cost and when unconstrained will seek to transform into a configuration with lower potential energy [35, 36]. We define the energy of the cell membrane as the total work done to maintain its shape. There are four major components to this energy, the energetic cost of bending which is regulated by the bending rigidity of the membrane and its local curvature, the cost of stretching which is regulated by membrane stiffness and the local tangent vector, the adhesion energy between the membrane and cortex which is regulated by the density of cortex-membrane binding proteins (linker proteins), and the cytoplasmic pressure induced work which results from fluid pushing the membrane outwards. The parameters of the model are chosen to correspond to physiological values of bending rigidity, membrane stiffness, stiffness of adhesion bonds and cytoplasmic pressure. We will refer to the energy contribution from the first two components of membrane energy as geometric energy and the force contribution to the energetic cost of stretching as membrane tension. Whereas our use of membrane tension is biologically relevant by the use of physiological values of membrane stiffness, it is determined geometrically and not measured experimentally. The mathematical formulation of total energy is expressed in terms of the Helfrich bending energy functional [37] with additional terms to account for work done by in-plane membrane tension, adhesion energy and pressure induced work. Details about the functional and its parameters are presented in the Methods section.

We investigated any potential influence of membrane energy on the location of nucleation sites within the bleb neck. Prior to bleb nucleation and expansion, the cell membrane is near the cortex and largely admits the shape of the cortex with possibly small perturbations resulting from the interaction between the cytosol and the lipid bilayer. We recovered the shape of the membrane by minimizing the energy functional with the cortex shape (obtained from the F-actin intensity) as an initial condition. Consequently, we treated the geometric force measurements from the membrane and cortex as related but different. The resulting minimal energy configuration of the membrane was used for our predictive analysis.

**Fig 3:**
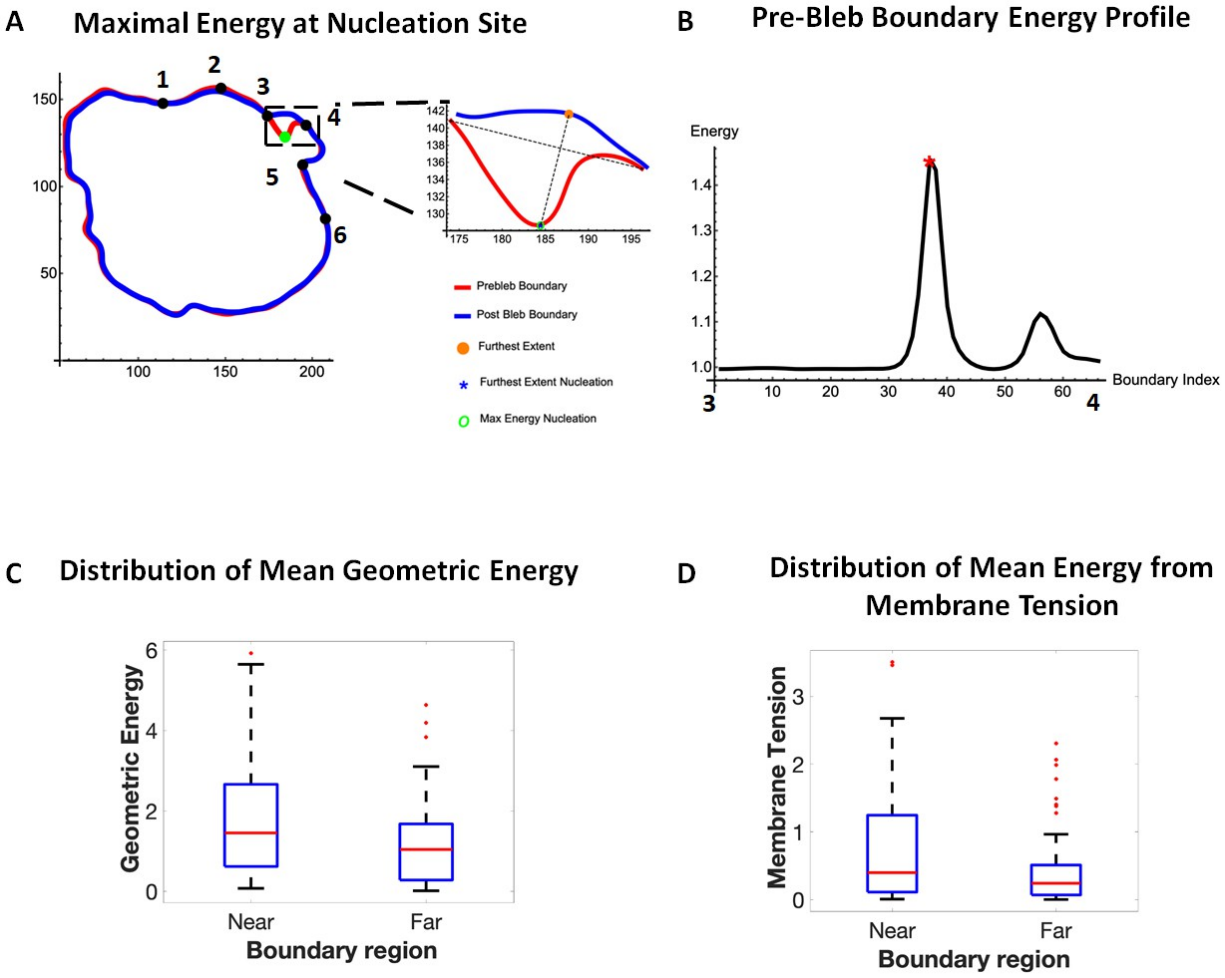
Nucleation sites predominantly form in locations of maximum energy. A) Pre-bleb and Post-bleb boundaries of a cell showing the bleb shoulder points and regions near the bleb (2–3,4-5), far from the bleb (1–2,5-6). An enlarged image of the bleb shows the furthest extent nucleation site at the location of maximal energy. B) The energy profile along the pre-bleb neck (3–4) showing the furthest extent nucleation site (red asterisk) having maximum energy. C). Distribution of mean geometric energy (bending and tension) near the bleb and far from the bleb. Difference in median is significant (Kruskal-Wallis test, p-value = 0.012). D) Distribution of energy from membrane tension near the bleb and far from the bleb. Data is taken from 28 blebs and 9 cells. Difference in median is significant (Kruskal-Wallis test, p-value = 0.0062).

We measured the energy at the nucleation site predicted by the furthest extent of the bleb for 109 blebs, 33 cells. In 96% of the nucleation sites we examined, the energy corresponded to a global maximum along the bleb neck (region between bleb shoulder points) (Fig 3B, S1 File). This result together with the observation of gaps or significant weakening in the cortex at nucleation sites suggest that cortex gaps may preferentially form in positions within the bleb neck where the membrane energy is maximal.

### Blebs form near regions with high geometric energy

To investigate any potential influence of membrane energy on the location of the bleb (not just its nucleation point), we compared energy due to membrane bending and stretching, cumulatively referred to as geometric energy, within the vicinity of the bleb. Using the length of the bleb neck, we identified regions near the bleb as being one bleb neck length away from each shoulder point (Fig 3A region 2–3,4-5) and regions two bleb neck lengths away from each shoulder point as being far from the bleb (Fig 3A region 1–2,5-6). Our results show a significantly higher geometric energy near the bleb than far from it (Fig 3C, S1 Data, Kruskal Wallis Test: pvalue = 0.0225). In particular, the energy due to membrane tension near the bleb was also significantly elevated compared to regions far from the bleb (Fig 3C). These data suggest that blebs preferentially form near regions of high geometric energy where the energy contribution from membrane tension is high.

### Degradation of the cortex is correlated with accumulation and clustering of myosin

**Fig 4:**
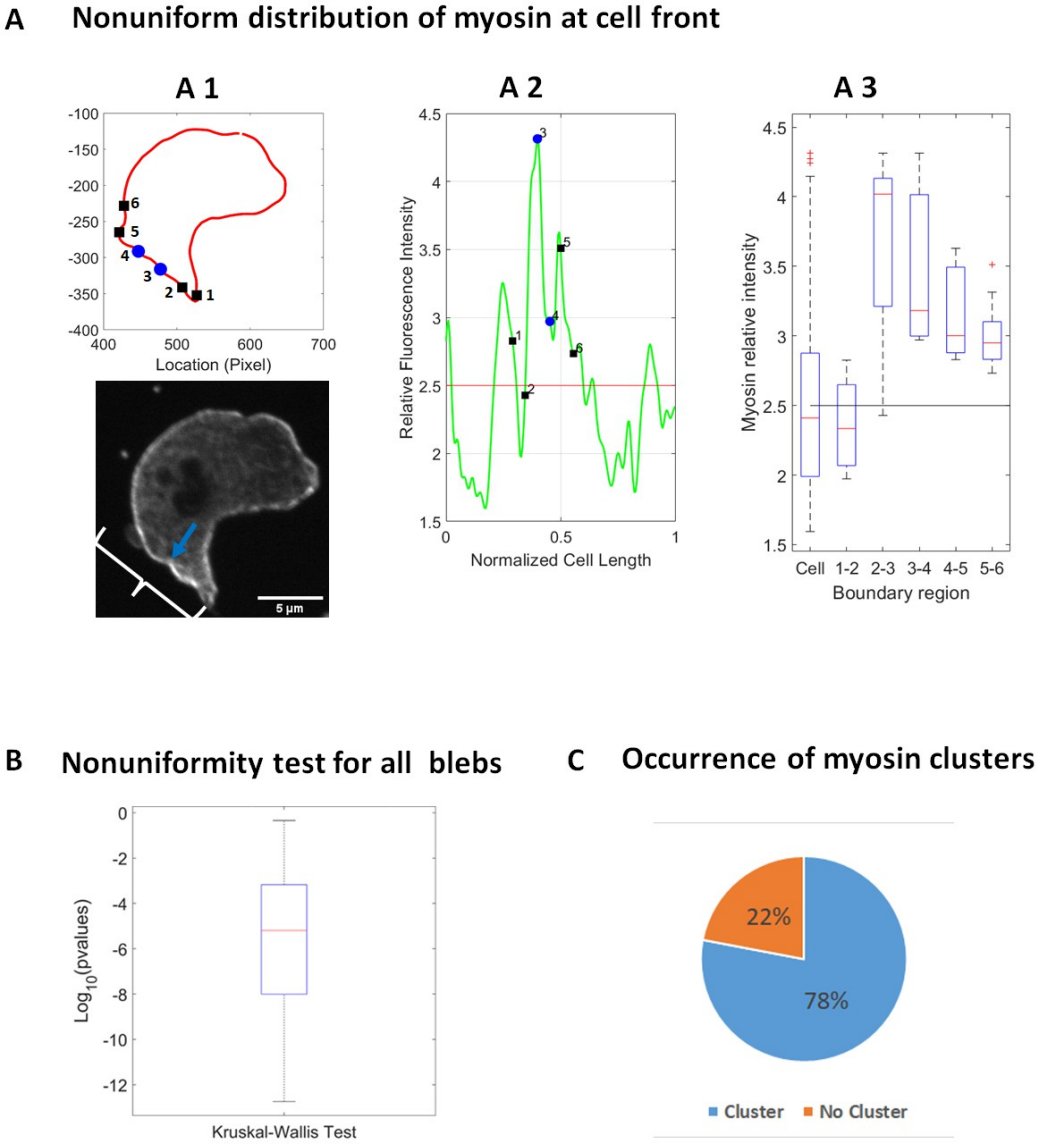
Microscopy evidence for non-uniform distribution and anterior clustering of myosin II at the cell front prior to bleb initiation. A1) (Top) The boundary of the cell (in red) showing the bleb shoulder points enclosing the bleb neck (blue dots) and the neighboring regions (black squares). (Bottom) Microscopy image shows a *Dictyoselium discoideum* cell expressing myosin-GFP prior to the formation of a bleb. The front of the cell just before the emergence of a bleb is marked with the curly bracket and the location of the incoming bleb by the blue arrow. A2) Smoothed myosin fluorescence intensity (relative to the cytoplasmic intensity) around the cell boundary showing the bleb shoulder points (blue dots) and neighboring boundary regions (black squares). The mean whole cell myosin intensity is marked by the red line. A3) A box plot showing the distribution of myosin intensity around the cell and in the neighboring regions of the bleb. Mean intensity is marked by black line. B) Distribution of p-values from Kruskal-Wallis tests of nonuniformity in the myosin intensity within regions at the front of the cell. C) A pie chart showing the percentage of blebs that formed with at least one myosin cluster in their surrounding region (Chi-square test, pvalue = 0.012).

To pursue the potential importance of myosin in gap formation suggested in the literature, we examined its localization in the cell cortex for *D. discoideum* cells actively blebbing while chemotaxing under an agarose gel (0.7%). Line scans of myosin intensity along the cortex prior to bleb nucleation were smoothed with a Gaussian kernel to reduce noise in our analysis. We sought to identify any distinguishing characteristics of the blebbing region (boundary region between bleb shoulder points) and its neighboring regions (two bleb-region lengths on either side of the bleb)(Fig 4A1, Top). We considered the bleb and its neighboring regions as our cell front.

The distribution of myosin at the cell front was observed to be nonuniform (Fig 4A1 Bottom—A3). We used the Kruskal-Wallis test to examine the differences between the distribution of myosin in each subregion of the cell front (1–2,2-3,3–4,4-5,5–6). Using data from 51 blebs and 20 cells, 94% of cell fronts showed a significant variation in the distribution of myosin within each subregion (Fig 4B). Regions of the cell front with a median myosin intensity significantly exceeding the whole cell median intensity were classified as myosin clusters. The Dunn’s test was used to investigate the statistical significance of pairwise differences in myosin distribution between specific cell front locations and the whole cortex. Myosin clusters returned a p-value<0.05. We observed at least one myosin cluster at the cell front in 78% of observed blebs about 72% of which occurred at or immediately next to a bleb (S1 Data, Chi-squared test: pvalue = 0.012). These results suggest an association between myosin clusters and bleb nucleation sites.

**Fig 5:**
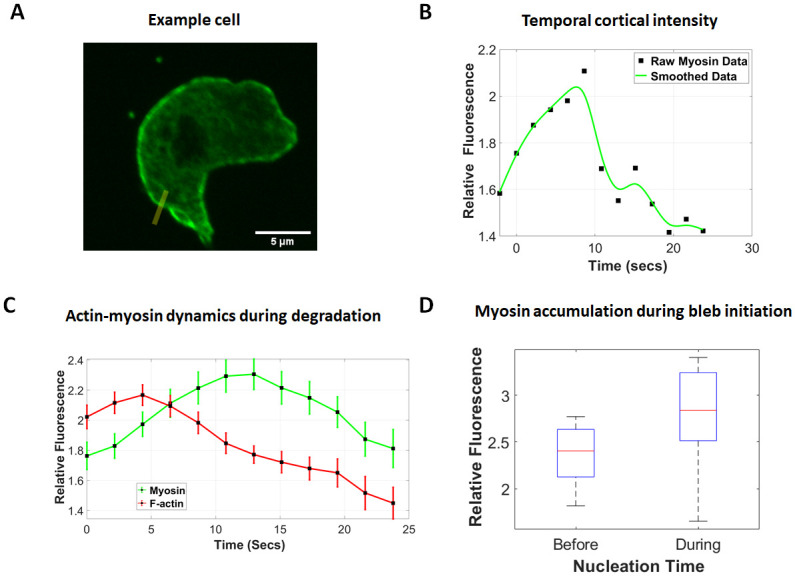
Myosin accumulation in the cortex correlates with cortex degradation. The fluorescence intensity of myosin II (myosin-GFP) and F-Actin (LifeAct-RFP) normalized to the level in the cytoplasm A) along a line through a location where a bleb develops. B) Intensity measurements at the cortex over the bleb development time frame are smoothed using regression splines. C) Time shifted mean myosin and F-Actin intensity for 39 blebs from 19 cells with error bars representing SEM. The point of bleb nucleation is set to time 0. D) Box plot showing the distribution of myosin before and at bleb nucleation. The frame rate is 2.14 seconds. Observed difference in medians is significant (Kruskal-Wallis test: p-value = 0.0226).

Additionally, we tracked the amount of myosin within a developing bleb by measuring the relative fluorescence intensity (cortex to cytoplasm) along line scans drawn across the cortex (cytoplasm to cell exterior) (Fig 5A). The intensity of myosin at the cortex during bleb development was smoothed using regression splines to reduce temporal noise (Fig 5B). In order to account for possible variation in myosin intensity along the cortex, we repeated the analysis for a different part of the cortex and averaged the results. We analyzed 39 blebs taken from 19 cells. Since blebs occurred at different times we had to temporally shift the intensity data to begin from the frame just before any evidence of nucleation was detected. The same analysis was repeated for F-actin intensity in the cortex. Our results show an accumulation of myosin in the cortex as the cortex degrades with myosin reaching a peak intensity within the first 12 seconds (Fig 5C). Interestingly, the distribution of myosin in the cortex before and at bleb nucleation showed a significant increase leading up to nucleation (Fig 5D, Kruskal- Wallis test: p-value = 0.0226). Together, these results correlate myosin accumulation and cortex degradation and point to a potential role for prenucleation accumulation of myosin in the rupture of the cortex.

## Discussion

Blebs nucleate when the cell membrane locally separates from its underlying cortex. One proposed mechanism for this separation is a local rupture of the cortex, based on the observation that blebs can be artificially formed by laser ablation of a part of the cortex. We sought in this work to investigate cortex rupture as a cell driven nucleation mechanism, clarify the role of myosin and investigate any role for membrane energy in the localization of resulting gaps in the cortex. By studying the actin scar of chemotaxing *D. discoideum* cells, we observed narrow gaps form in the cortex either before or immediately after nucleation. These gaps were observed at the furthest extent nucleation sites where we suspect the membrane most likely first detached from the cortex. An analysis of the energy distribution along the pre-bleb boundary using a theoretical free energy model revealed that the proposed nucleation sites occurred at locations where the membrane energy was maximized. In particular, regions immediately next to the bleb had higher energy contributions from membrane tension than surrounding regions further away from the bleb. Also, there was greater total geometric energy near the bleb. Additionally, we observed accumulation of myosin just prior to bleb nucleation and before the complete degradation of the cortex. Together, these findings point to a mechanistic connection between geometric energy, bleb initiation and myosin accumulation which merits further discussion.

### Myosin is enriched in regions of high geometric energy

We observed that the distribution of myosin in the cell front is nonuniform and exhibits clustering (Fig 4A3) which suggests a non-uniform distribution of cortical tension at the cell front. We also observed a majority of blebs forming at or near myosin clusters which are likely to have higher cortical tension. Since cortical tension is a major contributor to cell shape, our theoretical observation of higher geometric energy near blebs (Fig 3C), in particular higher membrane tension, is consistent with our biological observation of myosin clustering and suggests that myosin clusters may form in locations of high geometric energy where membrane tension is high.

In recent work, *D. discoideum* cells with increased membrane tension due to elevated confinement, recruited more myosin to the cortex [11]. In another study on red blood cells, non-muscle myosin IIA contraction was suggested to increase membrane tension [38]. It is thus reasonable to suggest that a nonuniform distribution of myosin at the cell front could lead to or result from spatial variations in membrane tension, which has been suggested to regulate cell motility and other cell behaviors [39]. Such spatial variations in membrane tension can be sustained by membrane to cortex proteins [39], contrary to other suggestions that tension is constant in the membrane [40].

### Myosin accumulation and formation of cortex gaps

We observed a significant accumulation of myosin in the actin scar prior to its complete degradation during bleb stabilization (Fig 5C) and leading up to bleb nucleation (Fig 5D). It is noteworthy that the build up in myosin occured without a commensurate increase in F-actin (Fig 5C), suggesting that this accumulation is not in response to an increase in binding sites. Rather, our observations suggest that myosin may be actively recruited to aid in the weakening and disassembly of the actin scar, pointing to the existence of a potential causative relationship between myosin accumulation and gap formation. Such a causative relationship is supported by experimental evidence on reduction in actin filament persistence length in response to excess myosin [41], as well as microrheological and viscoelastic experiments on stress induced cortex weakening [42–44].

It is not surprising therefore that majority of nucleation sites, determined by furthest extent, correlated with locations of maximal energy. Since more membrane tension induced work will be done at these locations than elsewhere on the bleb neck, more myosin could be recruited into the cortex there. The extra myosin-induced contractile stress could further increase the membrane tension at these points, potentially setting up a positive feedback loop that draws sufficient myosin to then rupture the cortex and create gaps.

While membrane tension is thought to oppose bleb expansion [26], it is reasonable for there to exist a threshold and distribution of membrane tension that facilitates myosin accumulation during bleb nucleation while restraining the extent to which hydrostatic pressure can push the membrane outward during bleb expansion.

### A model for bleb nucleation via cortex gaps in confined migration

**Fig 6:**
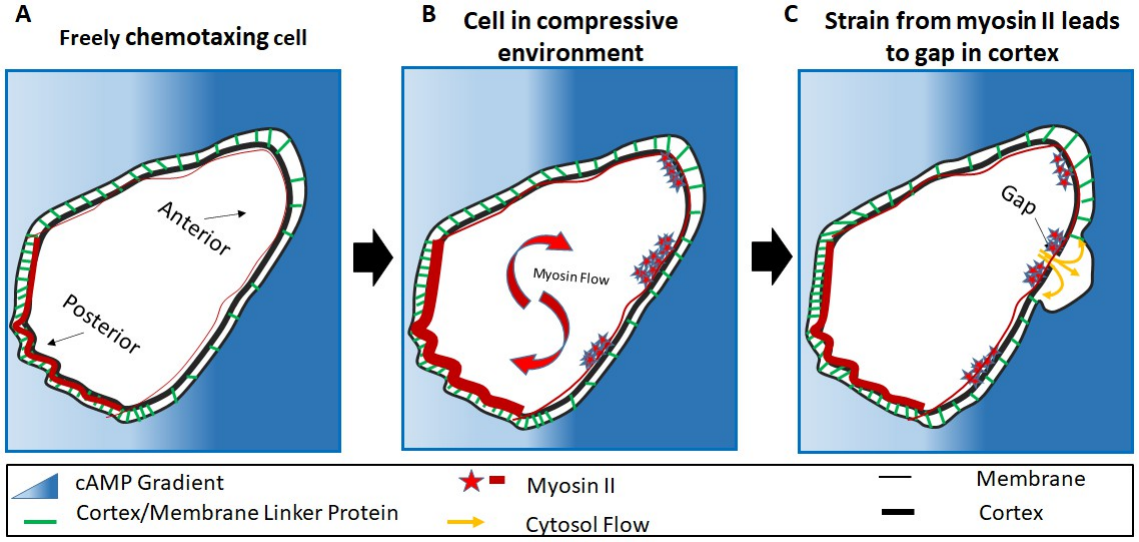
Model for bleb initiation. The image illustrates the cortex/membrane complex with connector protein distribution, posterior and anterior identified. A) The cell detects the cAMP gradient. B) Myosin density increases due to compression from the environment. Myosin is attracted by the cAMP causing a forward recruitment. it then forms clusters at the anterior. C) Myosin contractions results in cortex degradation, a gap forms weakening the connection to the membrane. The bleb follows at the cortex.

Based on the evidence presented so far in this study and the stated results from literature, we propose the following mechanistic model for bleb initiation due to the formation of cortex gaps during confined migration. For chemotaxing cells subjected to compression from the environment, the overall increase in membrane tension due to the flattening or squeezing of cells draws more myosin from the cytoplasm to the cortex in an effort to sustain cortical tension (Fig 6A). The presence of a cAMP gradient and its associated signal transduction events at the anterior of the cell recruits myosin to the cell anterior where it distributes non-uniformly, forming clusters in response to variation in geometric energy (Fig 6B). The clustering weakens the actin network potentially through increased myosin contraction, causing increased strain on the cortex. In locations where the total membrane energy is high, elevated membrane tension recruits additional myosin which eventually leads to a local rupture of the cortex (Fig 6C). Membrane to cortex binding proteins are consequently detached permitting pressurized cytosol to push the membrane out to initiate the formation of a bleb.

## Materials and methods

### Strain and culture conditions

All *D. discoideum* cells were grown axenically in shaking culture in HL5 nutrient medium with glucose (ForMedium) supplemented with 100 *μ*g/mL penicillin and streptomycin (Amresco) at 150 rpm at 22°C. Ax2 cells expressing LifeAct-GFP were grown in 4—20 *ug*/*ml* G418 (Geneticin) and transformed according to the protocol described in [33]. The LifeAct-GFP plasmid was obtained from Dr. Chang-Hoon Choi, University of California. Ax2 cells expressing both LifeAct-RFP and Myosin-GFP were grown in 50 *ug*/*ml* Hygromycin B (Thermo Fisher Scientific) and 4 *ug*/*ml* G418. The LifeAct-RFP plasmid was also obtained from Dr. Chang-Hoon Choi, University of California. The Myosin-GFP plasmids were obtained from Kuwayama Laboratory, University of Tsukuba, Japan. Ax2 cells were first transformed with Myosin-GFP plasmid. Successful cell lines were then subsequently transformed with LifeAct-RFP. The transformation protocol in [33] was used in both cases. In preparation for the under-agarose assay, cells were starved for cAMP competency on filter pads using the method described in [33].

### cyclic-AMP(cAMP) under agarose assay

Two cAMP under agarose assays were used to induce blebs as described [33] with minor modifications. The first under agarose assay was used to collect data on blebbing cells for validating the nucleation site predictions of the membrane energy functional. For this assay, wild type cells expressing LifeAct-GFP crawled under a 0.7% Omnipur agarose (EMD Millipore) gel that was laced with 1 mg/mL of 70,000 MW Rhodamine B isothiocyanate-Dextran (Sigma-Aldrich). In the second assay, used for myosin localization, the agarose gel was not laced with Rhodamine B isothiocyanate-Dextran. Rather, the membrane position was identified using a bright field light source. This was done to permit the identification of the cortex through the expression of LifeAct-RFP.

### Image acquisition

Images for validating nucleation site predictions were collected using a Leica DMI-4000B inverted microscope (Leica Microsystems Inc.) mounted on a TMC isolation platform (Technical Manufacturing Corporation) with a Yokogawa CSU 10 spinning disc head and Hamamatsu C9100–13 EMCCD camera (Perkin Elmer) with diode lasers of 491 nm, 561 nm and 638 nm (Spectra Services Inc.). LifeAct-GFP and RITC-Dextran were excited using the 491 nm and 561 nm lasers. Cells were imaged for 30 seconds using either 80x magnification (40x/1.25–0.75 oil objective with a 2x C-mount) or 100x 1.44 oil immersion objective. Data collected using both GFP and RITC channels resulted in a frame rate of 1.66 seconds whereas data collected using only GFP resulted in one frame rate of 0.800 seconds. Images for examining myosin localization were collected using a Perkin Elmer UltraView ERS spinning disk confocal microscope 103 (Perkin Elmer) equipped with 491 nm and 561 nm lasers (Spectra Services Inc.), as above, which were used to excite myosin-GFP and LifeAct-RFP, respectively. A bright field light source was used to illuminate the membrane. Data was collected at frame rate of 2.14 seconds.*Image J* was used to adjust brightness and contrast of the images, which were then imported into our Mathematica based geometric system for edge detection, boundary digitization and automatic bleb detection.

### Statistical analysis

All statistical test were performed using MATLAB r2020a. The Kruskal Wallis Test was used to test the significance of differences in geometric energy within regions close to the bleb and regions far from the bleb. Data on geometric energy values for these two regions was taken from 28 blebs. Outliers were removed prior to performing the statistical tests. A similar test was used to evaluate the significance of difference in myosin intensity within regions at the front of the cell. P-values for tests on the uniformity of myosin, performed on 51 blebs have been made available. The Kruskal Wallis test was also used to evaluate differences in the average intensity of myosin before and after nucleation of blebs for 48 blebs. All tests were performed at a 5% level of significance. All data have been made available in S1 Data.

### Edge detection and boundary digitization

We modified an in-house geometric platform [33] which extracts the boundary of photographic images and renders them as objects in two-dimensional Cartesian space. In our modified algorithm, the boundary of the image is thinned to a width of one pixel (using the *Thinning* function in *Mathematica*) prior to using a Canny edge detect function [45] to extract the boundary. In addition the procedure implements a more efficient algorithm for ordering boundary points, enforces a clockwise orientation of boundary points, and finally applies a Gaussian convolution to smooth the boundary. These additional processes were necessary to facilitate the automatic detection of blebs. The digitization of the boundary into equally spaced points using B-splines [46] is also implemented more efficiently.

#### Ordering boundary nodes

We implemented a straightforward algorithm to order boundary points. Let $P_{list}={\left\{p_{i}\right\}}_{i=1}^{n}$ be the set of boundary points, beginning from the first point in the list, *p*_1_ = (*p*_1*x*_, *p*_1*y*_) we obtain a set of candidates for the next ordered point, *C*_*list*_ = {*p*_*i*_ ∈ *P*_*list*_ \ *p*_1_ | (|*p*_*ix*_ − *p*_1*x*_| + |*p*_*iy*_ − *p*_1*y*_|) < *searchRadius*}. The next ordered point after *p*_1_ is then chosen to be the point in *C*_*list*_ closest to *p*_1_ in Euclidean distance (S1A Fig). *P*_*list*_ is reset to *C*_*list*_ and the process is repeated.

#### Orientation of boundary nodes

We chose to fix a clockwise orientation for all boundary points, *P*_*list*_ as follows. Let *P*_*convex*_ denote the boundary of the convex hull of *P*_*list*_, a portion of which is shown in S1B Fig. For each triplicate of points, {*p*_*i*_, *p*_*i*+1_, *p*_*i*+2_} we set the vectors $\hat{u}=p_{i+1}-p_{i}$ and $\hat{v}=p_{i+2}-p_{i+1}$ and calculate the turning angle, *α*_*i*_ using the formula

$$\alpha_{i}=\text{arccos}(\frac{\hat{u}\cdot \hat{v}}{\left\|\hat{u}\|\|\hat{v}\right\|}).$$

The ordering of the triplicate is clockwise if $Det[\hat{u},\hat{v}]<0$ and counterclockwise otherwise. The turning angle is assigned a negative or positive sign depending on whether the orientation of the triplicate is clockwise or counter clockwise. We consider the ordering of *P*_*list*_ to be clockwise if the sum of all turning angles is −2*π* and counterclockwise otherwise. In the event of the latter, we reverse the order of *P*_*list*_ to achieve a clockwise orientation. The algorithm can order about 1000 points in 0.36 seconds.

#### Gaussian smoothing of boundary nodes

We performed a discrete Gaussian convolution on boundary points by setting each point, $p_{i}=\sum_{j=-d}^{d}G_{j}p_{i+j}$ with weights *G*_*j*_ sampled from a standard normal density function with standard deviation, *d*.

#### Generation of equally spaced boundary nodes

A B-spline representation of the cell boundary, using *n* oriented boundary points, $P_{list}={\left\{p_{i}\right\}}_{i=1}^{n}$ requires fitting *n* − 3 segments of twice continuously differentiable curves with the *i*^*th*^ segment, $\sigma_{i}\left(t\right):\left[0,1\right]\to R^{2}$ constructed to lie in the convex hull of four consecutive (guide) points {*p*_*i*_, *p*_*i*+1_, *p*_*i*+2_, *p*_*i*+3_}. The result is a cubic polynomial formed from the convex combination of the guide points,

$$\sigma_{i}\left(t\right)=\sum_{j=0}^{3}\beta_{j+i}p_{j+i}$$

using basis functions

$$\beta_{1}\left(t\right)=\frac{1}{6}\left(-t^{3}+3t^{2}-3t+1\right),\;\beta_{2}\left(t\right)=\frac{1}{6}\left(3t^{3}-6t^{2}+4\right)$$

$$\beta_{3}\left(t\right)=\frac{1}{6}\left(-3t^{3}+3t^{2}+3t+1\right),\;\beta_{4}\left(t\right)=\frac{1}{6}\left(t^{3}\right).$$

Let $\Gamma^{i}\left(s\right)≔\int_{0}^{s}\|\sigma_{i}^{{}^{\prime }}\left(t\right)\|dt$ then the total distance along the cell boundary can be expressed as

$$\underset{s\to 1}{\text{lim}}\sum_{i=1}^{n-3}\Gamma^{i}\left(s\right).$$

In our previous work [33], we partitioned the boundary into equally spaced segments (0.5 pixels) using the following iterative process:

1. While |Γ(*s*) − 0.5| > *tol*
2. Set *s* = *k*Δ*s*
3. Numerically integrate Γ(*s*) using the *NIntegrate* function in Mathematica.
4. Set *k* = *k* + 1 and go to step 1.

Much of the computational time was spent adjusting the limits of integration until an appropriate limit was obtained to generate the desired segment length. To circumvent this costly search process, we directly integrated a Lagrange polynomial interpolation of the integrand, $f\left(s\right)=\|\sigma_{i}^{{}^{\prime }}\left(t\right)\|$ once and solved for the desired upper limit using a root search algorithm such as Secant method. Given $P_{m}\left(t\right)=\sum_{i=0}^{m}f\left(t_{i}\right)L_{i}\left(t\right),\;t\in \left[0,s\right]\subset \left[0,1\right]$ an *m*^*th*^ degree Lagrange polynomial with Lagrange basis functions $L_{i}\left(t\right)=\prod_{\begin{array}{c} j=0\\ j\neq i \end{array}}^{m}\frac{\left(t-t_{j}\right)}{\left(t_{i}-t_{j}\right)}$, we defined $Q_{m+1}\left(s\right)≔\int_{0}^{s}P_{m}\left(t\right)dt\approx \Gamma \left(s\right)$, and determine a segment with length 0.5 pixels by solving the nonlinear equation *Q*_*m*+1_ − 0.5 = 0. The process is illustrated in S1D Fig. The resulting equally spaced points are referred to as *Equlists*.

Our revised algorithm speeds up the previous algorithm for parameterizing the boundary [33] from 41.23 seconds/frame to 0.57 seconds/frame. These modifications were necessary to facilitate the efficient detection of shoulder points and calculation of geometric metrics used to isolate blebs from pseudopods in our bleb detection algorithm.

### Automatic detection of blebs

We have developed an algorithm that automatically identifies blebs from a time-lapse sequence of discretized cell boundary profiles (*Equlists*) and calculates desired bleb characteristics. Once the discrete, equally spaced, oriented boundary profiles are entered, the algorithm first identifies all protrusions using consecutive *Equlists* (Fig 1). The remaining parts of the algorithm utilize geometric properties of blebs to distinguish them from actin-based protrusion such as pseudopods (see S3B and S3C Fig). We expect this computational tool to greatly enhance research into bleb-based chemotaxis. In the subsections that follow we present the mathematical constructs used to accomplish this automation.

#### Detecting boundary protrusions

The set of distances between consecutive cell boundaries, taken along the normal direction define a *displacement profile* (Fig 1A, Right). We let the boundary points corresponding to the first cell image be the *prior cell* and use *post cell* for the second cell image. For each boundary point on the prior cell, the displacement is given a positive or negative sign depending on whether the point of intersection with the post cell lies in the exterior or interior of the prior cell. A *protrusion* is defined as a set of points whose displacement exceeds an upper threshold, *PU*_*thresh*_ with a significant change in curvature between successive frames.

#### Detecting shoulder points

For blebs, we refer to the points on the cell boundary where membrane peeling halts as *shoulder points*. These points should ideally be present in the *Equlists* corresponding to both the prior cell and post cell. Since the bleb boundary is usually split between two successive frames, the actin scar on the prior cell and the detached membrane on the post cell, the shoulder points serve as reference points for accurately defining the boundary of the bleb. We first identify a reference location in the displacement profile above *PU*_*thresh*_ for which a forward movement results in a point below *PU*_*thresh*_. Searching forward and backward from this reference point, the shoulder points are identified as the first set of points that lie below a designated *PL*_*thresh*_ to the left and right of the reference point(Fig 1A). For our analysis we set *PU*_*thresh*_ = 4*pixels* and *PL*_*thresh*_ = 1*pixel*.

#### Identifying the resting shape of protrusions

Since blebs develop over multiple frames, it is necessary to identify protrusions not only between successive frames, but by connecting frames over the full duration of bleb expansion. In order to identify the terminal frame of a particular bleb, we kept track of the extent to which the initial shoulder points are pushed outwards along the cell boundary. In particular, assume a clockwise ordering of boundary points and let *u*_1_, *u*_2_ be the indices of the left and right shoulder points for a protrusion on a prior cell and *v*_1_, *v*_2_ be the corresponding shoulder point indices on a post cell. Then the shoulder points correspond to the same protrusion if *v*_1_ < *u*_1_ + *ϵ* * (*u*_2_ − *u*_1_) and *v*_2_ > *u*_2_ − *ϵ* * (*u*_2_ − *u*_1_), where *ϵ* < 1 is determined experimentally. The bleb boundary is then defined as a list of points between *v*_1_, *v*_2_ on the initial frame (actin scar) joined with the points between *v*_1_, *v*_2_ on the terminal frame.

#### Isolating blebs from other actin-based protrusions

Up until this point, the discussion has been focused on identifying protrusions and their shoulder points with no ability to distinguish between blebs and pseudopods. To make this distinction, we used a two fold test which takes advantage of the geometric characteristics of blebs. First, since blebs are mostly spherical, we compare the average curvature of the leading edge of the protrusion (bleb boundary minus actin scar) to the curvature of a fitted circle (S3B Fig). We expect the curvature ratio to be close to one for blebs and further from one for pseudopods and other non-bleb protrusions. Secondly, we compared the protrusion area to half the area of a fitted circle (S3C Fig). Blebs were detected using a curvature threshold range of [0.51, 2.2]. Once the protrusions are determined to be blebs from these tests, the program gives the user an opportunity to make the final call by presenting microscopy images (cortex and membrane) corresponding to the initial and terminal frames of each identified bleb.

### A membrane energy functional for examining bleb nucleation sites

The energy functional is given by

$$\begin{array}{ccc} E_{\text{total}}\left(\bar{x},{\bar{x}}^{c}\right) & = & \oint \{\underset{Streching}{\underset{︸}{\frac{1}{2}\alpha [|\frac{d\bar{x}}{ds}|-1{]}^{2}}}+\underset{Bending}{\underset{︸}{\frac{1}{2}\beta |\frac{d^{2}\bar{x}}{ds^{2}}{|}^{2}}}+\underset{Adhesion}{\underset{︸}{\frac{1}{2}\kappa [|\bar{x}-{\bar{x}}^{c}|-l_{0}{]}^{2}}}\\ & + & \underset{Pressure}{\underset{︸}{-\frac{1}{2}\Pi {\left|\bar{x}-{\bar{x}}^{c}\right|}^{2}}}\}ds. \end{array}$$ (1)

The membrane and cortex configuration, parameterized with respect to arclength *s*, are denoted by $\bar{x}=\left(x\left(s\right),y\left(s\right)\right)$ and ${\bar{x}}^{c}=\left(x^{c}\left(s\right),y^{c}\left(s\right)\right)$ respectively. The first two terms refer to the energy density (energy per unit length) contribution from membrane stretching and bending with membrane stiffness *α*, and flexural rigidity, *β*. Stretching of the membrane is modeled using dimensionless deviations of the unit tangent vector from its value of one in the reference configuration of the cell boundary. This is consistent with current models of membrane tension induced energy for 1D representations of the cell boundary [16, 18]. The third term is a representation of the adhesion energy between the membrane and the cortex. Proteins connecting the membrane to the cortex have been found around the boundary of migrating cells [18, 47]. Their contribution to adhesion energy is commonly modeled using linear elastic spring with spring constant *κ* [17, 18, 23, 24]. The resting linker length, for which there is no tension, is denoted as *l*_0_(*μm*). While linker proteins may be absent (or reduced) at the front of the cell where blebs form, they are still present at the back of the cell. Our energy functional minimizes energy over the entire cell boundary, so we have left the linker term in to give us a good representation of the minimum energy configuration of the membrane. These three energy components are similar to the model in [18] with modified units for the physical constants to yield units of energy (*pNμm*) after integration. Our fourth term is different and models the work done by hyrostatic pressure in moving the membrane away from the cortex. It is consistent with the energy formulation in [34].

#### Implementation of the energy functional

In order to calculate the membrane energy we needed to identify the location of the membrane and cortex across multiple frames, discretize their respective configurations and finally feed that information into a discrete version of the functional. The cortex configuration was determined from the microscopy images as previously discussed. We initialized the membrane to be an equidistant, outward normal projection of the cortex **X**^0^. The separation distance, $l_{i}=\left|{\bar{x}}_{i}-{\bar{x}}_{i}^{c}\right|$ was set to the experimentally determined resting length of the linker proteins (0.04 *μ*m [16]). Since pressure is not equilibrated instantaneously in biological cells [26] leaving spatial variations that can persist on the timescale of bleb nucleation [34], we expect there to be variations in membrane separation from the cortex. Following from the principle of least action, we expect these variations will lead to a membrane shape with minimum energy. Subsequently, we determined the true membrane location to be the configuration $X={\left\{{\bar{x}}_{i}\right\}}_{i=1}^{n}$ which minimized the discrete energy functional, $\varepsilon_{total}\left(X\right)\approx E_{total}\left(\bar{x},{\bar{x}}^{c}\right)$, given by

$$\begin{array}{ccc} \varepsilon_{total}\left({\bar{x}}_{1},{\bar{x}}_{2},\cdots ,{\bar{x}}_{n}\right) & = & \sum_{i=1}^{n}\varepsilon \left({\bar{x}}_{i}\right)\Delta s, \end{array}$$ (2)

with the pointwise energy, $\varepsilon ({\bar{x}}_{i})$, defined as

$$\begin{array}{ccc} \varepsilon ({\bar{x}}_{i}) & = & \frac{1}{2}\alpha [({\left(\frac{x_{i+1}-x_{i-1}}{2\Delta s}\right)}^{2}+{\left(\frac{y_{i+1}-y_{i-1}}{2\Delta s}\right)}^{2}{)}^{1/2}-1{]}^{2}\\ & + & \frac{1}{2}\beta [(\frac{x_{i+1}-2x_{i}+x_{i-1}}{{\left(\Delta s\right)}^{2}}{)}^{2}+(\frac{y_{i+1}-2y_{i}+y_{i-1}}{{\left(\Delta s\right)}^{2}}{)}^{2}]\\ & + & \frac{1}{2}\kappa {\left({\left[{\left(x_{i}-x_{i}^{c}\right)}^{2}+{\left(y_{i}-y_{i}^{c}\right)}^{2}\right]}^{1/2}-l_{0}\right)}^{2}\\ & - & \frac{1}{2}\Pi {\left(x_{i}-x_{i}^{c}\right)}^{2}+{\left(y_{i}-y_{i}^{c}\right)}^{2}. \end{array}$$

Here, the first and second derivatives in the continuous energy functional have been discretized using the first and second central difference schemes with arclength Δ*s*. The minimization was done using the method of steepest descent [46] (See S1 Appendix for details).

We initialized our model parameters with *α* = 17 *pN*, *β* = 0.14 *pN*(*μm*)^2^, *κ* = 10 *pN*/*μm*^2^, Π = 81 *Pa*, magnitudes consistent with the parameter in [16]. Due to the difference in units, we adjusted the values of *α*, *β*, *κ* until a positive energy profile was obtained after minimization.

#### Robustness of energy functional

We investigated the extent to which variation in the model parameters, *α*, *β*, *κ*, Π would affect the minimum membrane energy configuration. We allowed each parameter to vary by 20% 40%, 60%, 80% and 100% of their measured values, and generated 1000 uniform random samples from each interval. Each sample from a particular interval was used to determine the location of maximal energy within a chose bleb. This was compared with the nucleation point of the same bleb. The Euclidean distance between the predicted site and the observed site was used to measure the level of variation in model output. There was effectively no change in model predictions for up to 60% variation in the measured values (S4 Fig). Since our perturbations to account for unit changes in model parameters did not exceed 60% of the initial measured value, we conclude that the model predictions are robust to reasonable perturbations.

## Supporting information

S1 Fig Cell boundary ordering, orientation, smoothing and parameterization. The techniques used to order, orient and smooth the boundary of cells after edge detection are shown. A) A scatter plot of boundary points with the shaded region denoting the nearest neighborhood of the current boundary point (shown in red) obtained using the Manhattan distance metric. The next ordered point (shown in green) is subsequently obtained as the closest point (in Euclidean distance) to the current point. B) The turning angles *α*_1_ and *α*_2_ for the triplicate *p*_1_, *p*_2_, *p*_3_ are shown. C) The edge detected from a portion of the cell shows artificial sharp changes in curvature which are resolved using Gaussian smoothing. D) Equally spaced points are generated from a B-spline representation of the ordered, oriented boundary points.
(TIF)

S2 Fig A flowchart showing the methodology for detecting blebs from a time-lapse of discretized cell boundaries. (TIF)

S3 Fig Final output of the bleb detection algorithm for selected blebs. Figure shows results of all four geometric tests used to validate the presence of a bleb as well the major geometric markers used. Blue outline indicates the cell boundary before the bleb forms, the orange line indicates the cell boundary after the bleb forms. Blue dots indicate the bleb shoulder points. Green dot is the furthest extent of the bleb and the red dot shows the observed nucleation point. A) True positive and true negative classification of blebs. B) The mean curvature of the bleb boundary relative to a fitted circle used to distinguish blebs from pseudopods. C) The area enclosed by the bleb boundary relative to the area of a fitted semi circle used as a final confirmation of blebs. Scale bar is 5*μ*m.
(TIF)

S4 Fig Robustness of energy functional 1000 uniform random vectors of the model parameters, *α*, *β*, *κ*, Π, are simulated and used to investigate the robustness of the energy functional. The box plot shows the distribution of model output when model parameters are varied by 20%, 40%, 60%, 80% and 100% of their measured values.
(TIF)

S1 Video Myosin accumulation prior to cortex degradation during bleb stabilization. Video of cell shown in Fig 5.
(AVI)

S1 Data Excel spreadsheet containing data supporting results in figures and tables. (XLSX)

S1 Appendix Minimization of membrane energy functional. Details of the organization of the discrete energy functional for application of gradient descent minimization.
(PDF)

S1 File Microscopy and boundary images showing energy predictions of nucleation sites. Microscopy images of Ax2 *D. discoideum* cells expressing LifeAct-GFP, boundary images with bleb shoulder points obtained from bleb detection algorithm with energy predictions of nucleation sites and energy profile over bleb neck.
(PDF)
