# Supplementary figures and images for: A role for myosin II clusters and membrane energy in cortex rupture for Dictyostelium discoideum
Source: PLoS One. 2022 Apr 25;17(4):e0265380. doi: 10.1371/journal.pone.0265380 (PMC9037949; doi:10.1371/journal.pone.0265380)

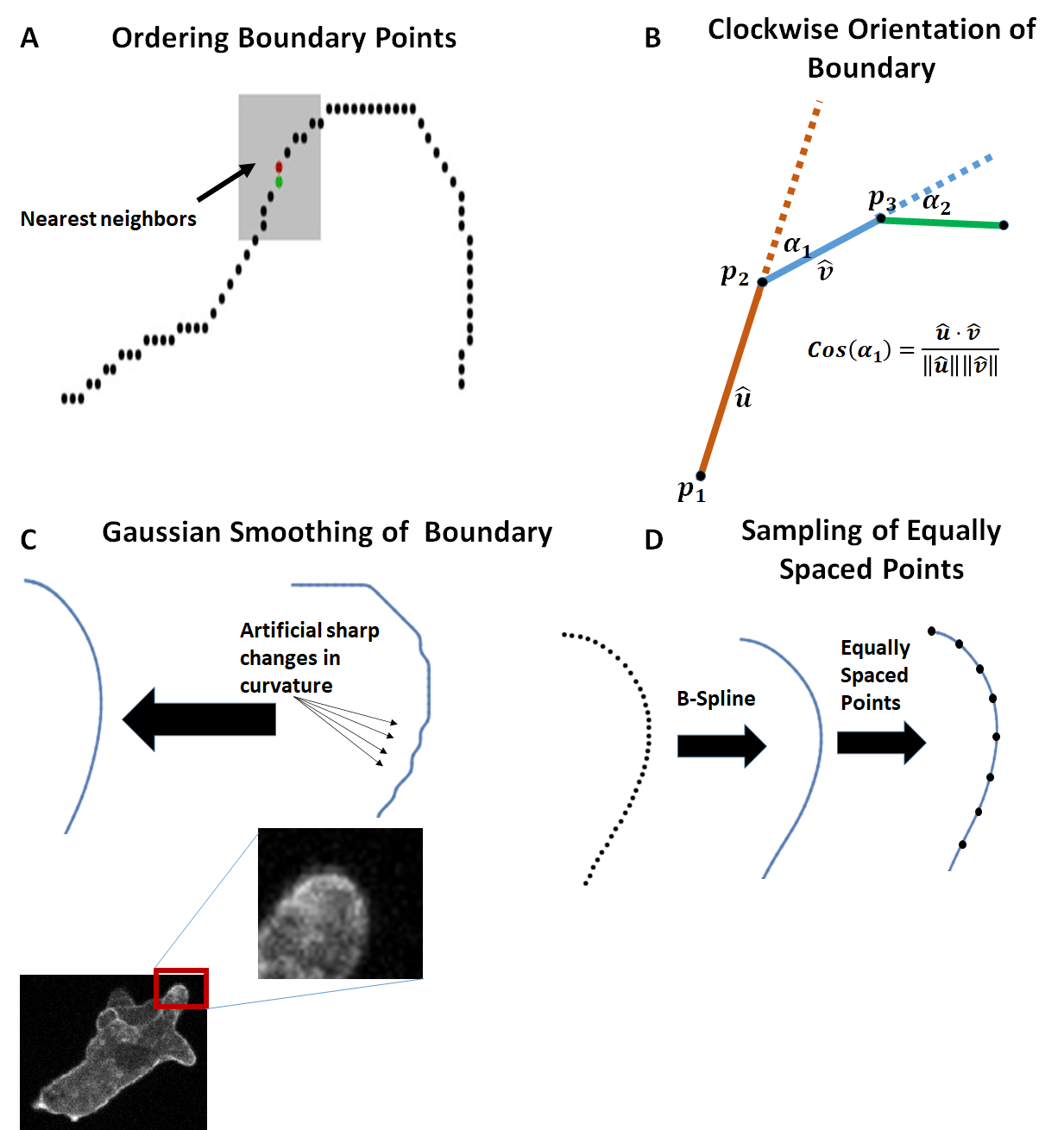

Supplement: S1 Fig — The techniques used to order, orient and smooth the boundary of cells after edge detection are shown. A) A scatter plot of boundary points with the shaded region denoting the nearest neighborhood of the current boundary point (shown in red) obtained using the Manhattan distance metric. The next ordered point (shown in green) is subsequently obtained as the closest point (in Euclidean distance) to the current point. B) The turning angles α1 and α2 for the triplicate p1, p2, p3 are shown. C) The edge detected from a portion of the cell shows artificial sharp changes in curvature which are resolved using Gaussian smoothing. D) Equally spaced points are generated from a B-spline representation of the ordered, oriented boundary points. (TIF) [file pone.0265380.s001.tif]

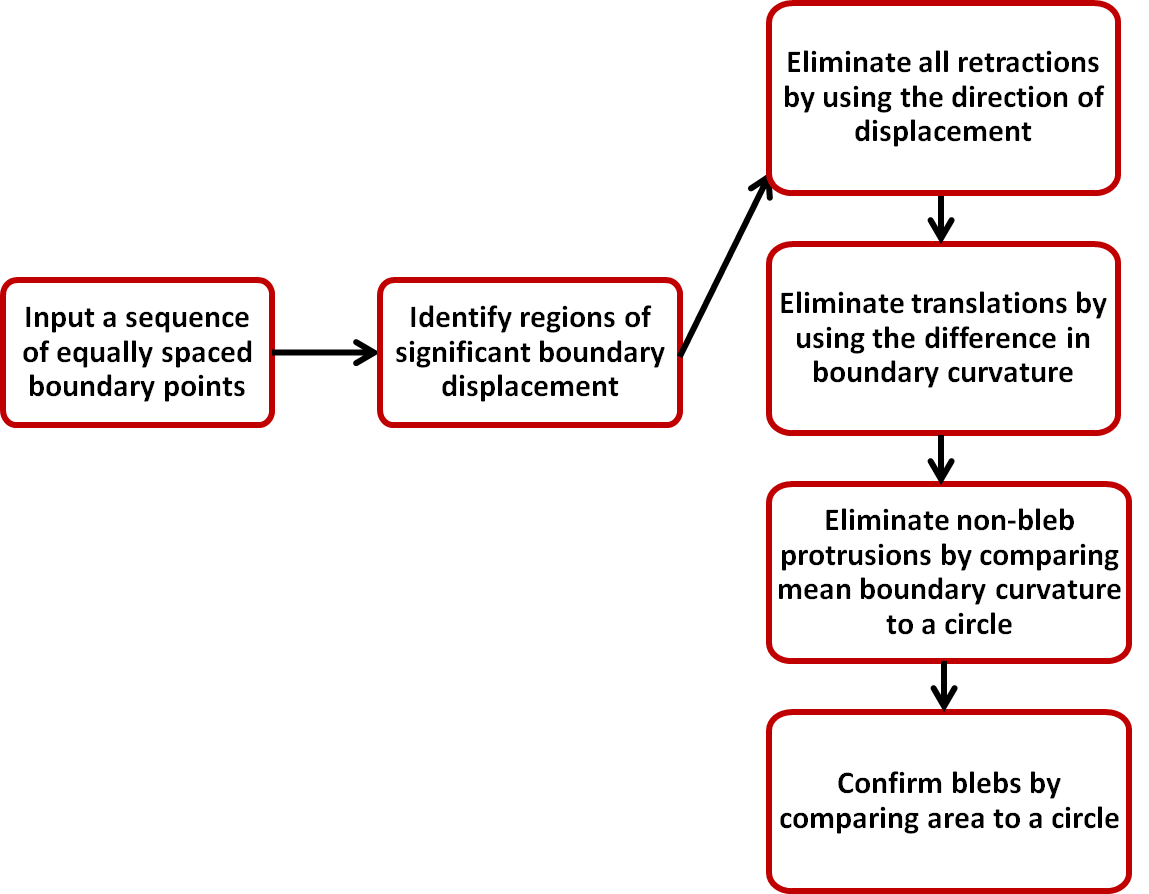

Supplement: S2 Fig — (TIF) [file pone.0265380.s002.tif]

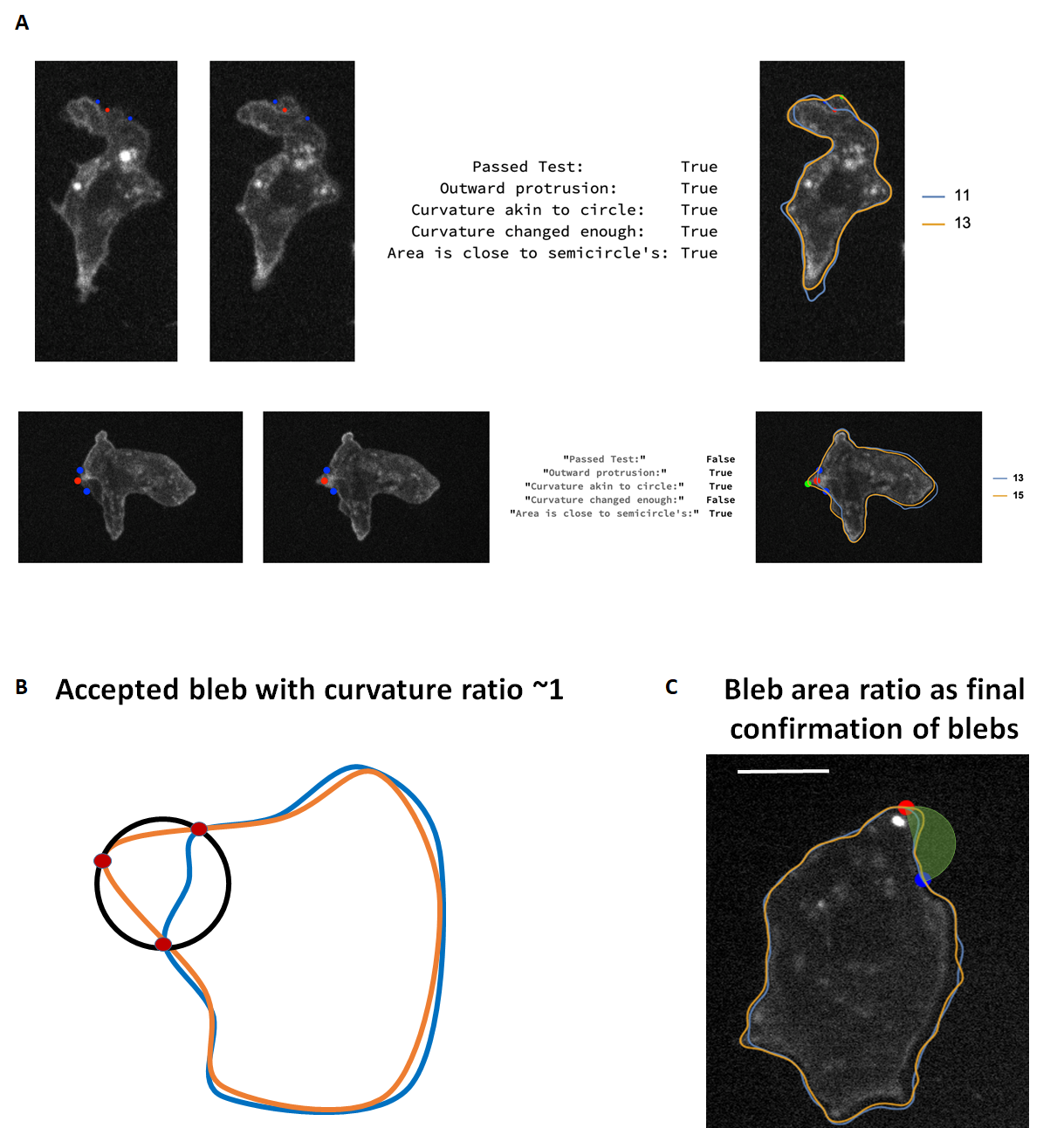

Supplement: S3 Fig — Figure shows results of all four geometric tests used to validate the presence of a bleb as well the major geometric markers used. Blue outline indicates the cell boundary before the bleb forms, the orange line indicates the cell boundary after the bleb forms. Blue dots indicate the bleb shoulder points. Green dot is the furthest extent of the bleb and the red dot shows the observed nucleation point. A) True positive and true negative classification of blebs. B) The mean curvature of the bleb boundary relative to a fitted circle used to distinguish blebs from pseudopods. C) The area enclosed by the bleb boundary relative to the area of a fitted semi circle used as a final confirmation of blebs. Scale bar is 5μm. (TIF) [file pone.0265380.s003.tif]

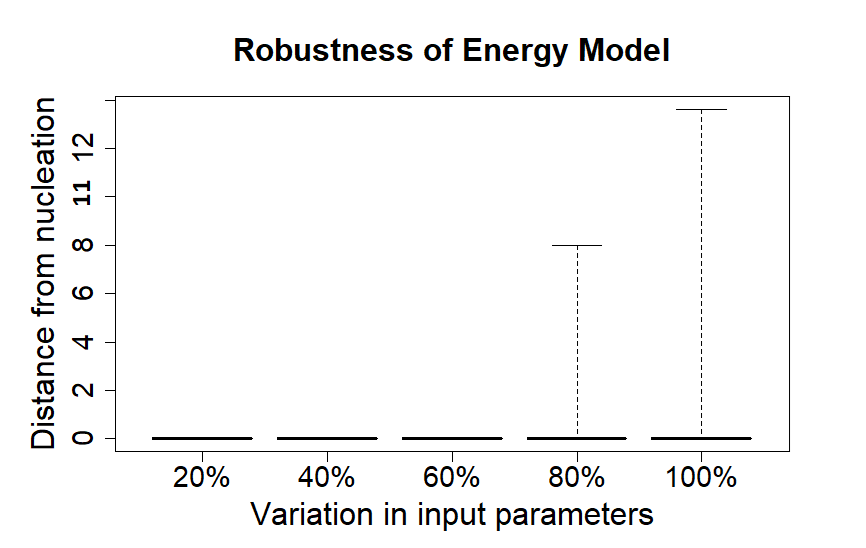

Supplement: S4 Fig — The box plot shows the distribution of model output when model parameters are varied by 20%, 40%, 60%, 80% and 100% of their measured values. (TIF) [file pone.0265380.s004.tif]
