# Supplementary material for: A role for myosin II clusters and membrane energy in cortex rupture for Dictyostelium discoideum
Source: PLoS One. 2022 Apr 25;17(4):e0265380. doi: 10.1371/journal.pone.0265380 (PMC9037949; doi:10.1371/journal.pone.0265380)
Supplement: S1 Appendix — Details of the organization of the discrete energy functional for application of gradient descent minimization. (PDF) [file pone.0265380.s007.pdf]

Membrane Energy Minimization Using Gradient Descent

Minimizing the Energy Functional

We proposed the following functional representation of the cell membrane energy

$$\mathrm{E}_{\mathrm{total}}(\bar{x},\bar{x}^c) = \oint \left\{ \underbrace{\frac{1}{2}\alpha\left[\left|\frac{d\bar{x}}{ds}\right| - 1\right]^2}_{Membrane\ Tension} + \underbrace{\frac{1}{2}\beta\left|\frac{d^2\bar{x}}{ds^2}\right|^2}_{Bending} + \underbrace{\frac{1}{2}\kappa\left[|\bar{x}-\bar{x}^c| - l_0\right]^2}_{Linker\ Tension} \underbrace{-\frac{1}{2}\Pi|\bar{x}-\bar{x}^c|^2}_{Pressure} \right\} ds.$$

The notation and significance of each component of the model have been clearly discussed in the Materials and Methods section. The continuous model was discretized using finite differences to obtain the following discrete energy function

$$\varepsilon_{total}(\hat{x}_1,\hat{x}_2,\cdots,\hat{x}_n) = \varepsilon_1(\hat{x}_1) + \sum_{i=2}^{n-1}\varepsilon_2(\hat{x}_i)\Delta s + \varepsilon_3(\hat{x}_n),$$

with the pointwise energy density, $\varepsilon(\bar{x}_i)$, defined as

$$\begin{aligned} \varepsilon_2(\hat{\mathrm{x}}_{\mathrm{i}}) &= \frac{1}{2}\alpha\left[\left(\left(\frac{x_{i+1}-x_{i-1}}{2\Delta s}\right)^2 + \left(\frac{y_{i+1}-y_{i-1}}{2\Delta s}\right)^2\right)^{1/2} - 1\right]^2 \\ &+ \frac{1}{2}\beta\left[\left(\frac{x_{i+1}-2x_i+x_{i-1}}{(\Delta s)^2}\right)^2 + \left(\frac{y_{i+1}-2y_i+y_{i-1}}{(\Delta s)^2}\right)^2\right] \\ &+ \frac{1}{2}\kappa([(x_i-x_i^c)^2+(y_i-y_i^c)^2]^{1/2}-l_0)^2 \qquad (1) \\ &- \frac{1}{2}\Pi[(x_i-x_i^c)^2+(y_i-y_i^c)^2] \end{aligned}$$

for $i = 2\cdots n-1$,

$$
\begin{aligned}
\varepsilon_1(\hat{\mathrm{x}}_1) &= \frac{1}{2}\alpha\left[\left(\left(\frac{x_2-x_1}{\Delta s}\right)^2+\left(\frac{y_2-y_1}{\Delta s}\right)^2\right)^{1/2}-1\right]^2 \\
&+ \frac{1}{2}\beta\left[\left(\frac{2x_1-5x_2+4x_3-x_4}{(\Delta s)^2}\right)^2+\left(\frac{2y_1-5y_2+4y_3-y_4}{(\Delta s)^2}\right)^2\right] \\
&+ \frac{1}{2}\kappa([(x_1-x_1^c)^2+(y_1-y_1^c)^2]^{1/2}-l_0)^2 \\
&- \frac{1}{2}\Pi[(x_1-x_1^c)^2+(y_1-y_1^c)^2]
\end{aligned}
$$

for first boundary point and

$$
\begin{aligned}
\varepsilon_3(\hat{\mathrm{x}}_\mathrm{n}) &= \frac{1}{2}\alpha\left[\left(\left(\frac{x_n-x_{n-1}}{\Delta s}\right)^2+\left(\frac{y_n-y_{n-1}}{\Delta s}\right)^2\right)^{1/2}-1\right]^2 \\
&+ \frac{1}{2}\beta\left[\left(\frac{2x_n-5x_{n-1}+4x_{n-2}-x_{n-3}}{(\Delta s)^2}\right)^2+\left(\frac{2y_n-5y_{n-1}+4y_{n-2}-y_{n-3}}{(\Delta s)^2}\right)^2\right] \\
&+ \frac{1}{2}\kappa([(x_n-x_n^c)^2+(y_n-y_n^c)^2]^{1/2}-l_0)^2 \\
&- \frac{1}{2}\Pi[(x_n-x_n^c)^2+(y_n-y_n^c)^2]
\end{aligned}
$$

for the last boundary point.

An implementation of the method of steepest descent requires the gradient of the discrete energy functional. We define it as follows:

$$\nabla\varepsilon_{total} = (\partial_{x_1},\cdots,\partial_{x_n},\partial_{y_1},\cdots,\partial_{y_n})^T\varepsilon_{total}$$

where, $\partial_{x_j}\varepsilon_{total} = \frac{\partial\varepsilon_{total}}{\partial x_j}$. Given the gradient vector $\nabla\varepsilon_{total}$ at the initial membrane location

$$\bar{X}^0 = (x_1^0, x_2^0, \cdots, x_n^0, y_1^0, \cdots, y_n^0)^T$$

a new membrane location

$$\bar{X}^{k+1} = \bar{X}^k + t_k\nabla\varepsilon_{total}(\bar{X}^k)$$

is computed as long as $\varepsilon_{total}(\bar{X}^{k+1}) < \varepsilon_{total}(\bar{X}^k)$. To ensure that this condition is met at each step k, we chose $t_k = \text{Argmin } h(t)$, where the single variable

function $h(t) = \varepsilon_{total}(\bar{X}^k + t\nabla\varepsilon_{total}(\bar{X}^k))$ is minimized using a quadratic interpolation search method ([1], Section 8.1). It remains to compute the gradient of the discrete energy function.

We will derive the gradient for the x derivatives and replace variables to obtain the expression for the y-derivatives. Now,

$$\frac{\partial \varepsilon_{total}}{\partial x_j} = \left(\frac{\partial \varepsilon_1(\hat{x}_1)}{\partial x_j} + \frac{\partial}{\partial x_j} \sum_{i=2}^{n-1} \varepsilon_2(\hat{x}_i) + \frac{\partial \varepsilon_3(\hat{x}_n)}{\partial x_j} \right) \Delta s.$$

where

$$\frac{\partial}{\partial x_j} \sum_{i=2}^{n-1} \varepsilon_2(\hat{x}_i)\Delta s = \begin{cases} \frac{\partial}{\partial x_j}(\varepsilon_2(\hat{x}_{j+1}))\Delta s & \text{if } j = 1 \\ \frac{\partial}{\partial x_j}(\varepsilon_2(\hat{x}_j) + \varepsilon_2(\hat{x}_{j+1}))\Delta s & \text{if } j = 2 \\ \frac{\partial}{\partial x_j}(\varepsilon_2(\hat{x}_{j-1}) + \varepsilon_2(\hat{x}_j) + \varepsilon_2(\hat{x}_{j+1}))\Delta s & \text{if } j = 3, \cdots, n-2 \ . \\ \frac{\partial}{\partial x_j}(\varepsilon_2(\hat{x}_{j-1}) + \varepsilon_2(\hat{x}_j))\Delta s & \text{if } j = n-1 \\ \frac{\partial}{\partial x_j}(\varepsilon_2(\hat{x}_{j-1}))\Delta s & \text{if } j = n. \end{cases}$$

Here

$$\frac{\partial \varepsilon_2(\hat{x}_{j-1})}{\partial x_j}\Delta s = \frac{\alpha}{4\Delta s}(x_j - x_{j-2}) + \frac{\beta}{(\Delta s)^3}(x_j - 2x_{j-1} + x_{j-2}) - \frac{\alpha}{4\Delta s}\frac{(x_j - x_{j-2})}{\| \frac{\hat{x}_j - \hat{x}_{j-2}}{2\Delta s} \|}$$

$$\frac{\partial \varepsilon_2(\hat{x}_j)}{\partial x_j}\Delta s = -\frac{2\beta}{(\Delta s)^3}(x_{j+1} - 2x_j + x_{j-1}) - \Pi(x_j - x_j^c)\Delta s$$

$$\frac{\partial \varepsilon_2(\hat{x}_{j+1})}{\partial x_j}\Delta s = -\frac{\alpha}{4\Delta s}(x_{j+2} - x_j) + \frac{\beta}{(\Delta s)^3}(x_{j+2} - 2x_{j+1} + x_j) + \frac{\alpha}{4\Delta s}\frac{(x_{j+2} - x_j)}{\| \frac{\hat{x}_{j+2} - \hat{x}_j}{2\Delta s} \|}.$$

Beginning with $j = 1$ and assuming $n > 4$ we have,

$$\frac{\partial \varepsilon_{total}}{\partial x_1} = \frac{\partial \varepsilon_1(\hat{x}_1)}{\partial x_1}\Delta s + \frac{\partial \varepsilon_2(\hat{x}_2)}{\partial x_1}\Delta s$$

since $\frac{\partial \varepsilon_3(\hat{x}_n)}{\partial x_j} = 0$ for $j < n - 3$. Now,

$$\frac{\partial \varepsilon_1(\hat{x}_1)}{\partial x_1}\Delta s = -\frac{\alpha}{\Delta s}(x_2 - x_1) + \frac{2\beta}{(\Delta s)^3}(2x_1 - 5x_2 + 4x_3 - x_4) + \frac{\alpha}{\Delta s}\frac{(x_2 - x_1)}{\| \frac{\hat{x}_2 - \hat{x}_1}{\Delta s} \|}$$
$$+ \kappa(x_1 - x_1^c)\Delta s - \frac{\kappa l_0(x_1 - x_1^c)\Delta s}{\| \hat{x}_1 - \hat{x}_1^c \|} - \Pi(x_1 - x_1^c)\Delta s$$

and

$$\frac{\partial \varepsilon_2(\hat{x}_2)}{\partial x_1}\Delta s = -\frac{\alpha}{4\Delta s}(x_3 - x_1) + \frac{\beta}{(\Delta s)^3}(x_3 - 2x_2 + x_1) + \frac{\alpha}{4\Delta s}\frac{(x_3 - x_1)}{\parallel \frac{\hat{x}_3 - \hat{x}_1}{2\Delta s} \parallel}.$$

Consequently,

$$\frac{\partial \varepsilon_{total}}{\partial x_1} = x_1\left(\frac{3\alpha}{4\Delta s} + \frac{5\beta}{(\Delta s)^3} + \kappa\Delta s - \Pi\Delta s\right) + x_2\left(-\frac{\alpha}{\Delta s} - \frac{12\beta}{(\Delta s)^3}\right) + x_3\left(-\frac{\alpha}{4\Delta s} + \frac{9\beta}{(\Delta s)^3}\right)$$
$$+ x_4\left(-\frac{2\beta}{(\Delta s)^3}\right) + (\Pi - \kappa)x_1^c\Delta s + \frac{\alpha}{\Delta s}\frac{(x_2 - x_1)}{\parallel \frac{\hat{x}_2 - \hat{x}_1}{\Delta s} \parallel} + \frac{\alpha}{4\Delta s}\frac{(x_3 - x_1)}{\parallel \frac{\hat{x}_3 - \hat{x}_1}{2\Delta s} \parallel} - \frac{\kappa l_0(x_1 - x_1^c)\Delta s}{\parallel \hat{x}_1 - \hat{x}_1^c \parallel}$$

Now for $j = 2$, we have,

$$\frac{\partial \varepsilon_{total}}{\partial x_2} = \frac{\partial \varepsilon_1(\hat{x}_1)}{\partial x_2}\Delta s + \frac{\partial}{\partial x_2}(\varepsilon_2(\hat{x}_2) + \varepsilon_2(\hat{x}_3))\Delta s.$$
$$\frac{\partial \varepsilon_1(\hat{x}_1)}{\partial x_2}\Delta s = \frac{\alpha}{\Delta s}(x_2 - x_1) - \frac{5\beta}{(\Delta s)^3}(2x_1 - 5x_2 + 4x_3 - x_4) - \frac{\alpha}{\Delta s}\frac{(x_2 - x_1)}{\parallel \frac{\hat{x}_2 - \hat{x}_1}{\Delta s} \parallel}$$

and

$$\frac{\partial \varepsilon_2(\hat{x}_2)}{\partial x_2}\Delta s = -\frac{2\beta}{(\Delta s)^3}(x_3 - 2x_2 + x_1) + (\kappa - \Pi)(x_2 - x_2^c)\Delta s - \frac{\kappa l_0(x_2 - x_2^c)\Delta s}{\parallel \hat{x}_2 - \hat{x}_2^c \parallel},$$
$$\frac{\partial \varepsilon_2(\hat{x}_3)}{\partial x_2}\Delta s = -\frac{\alpha}{4\Delta s}(x_4 - x_2) + \frac{\beta}{(\Delta s)^3}(x_4 - 2x_3 + x_2) + \frac{\alpha}{4\Delta s}\frac{(x_4 - x_2)}{\parallel \frac{\hat{x}_4 - \hat{x}_2}{2\Delta s} \parallel}.$$

Consequently,

$$\frac{\partial \varepsilon_{total}}{\partial x_2} = x_1\left(-\frac{\alpha}{\Delta s} - \frac{12\beta}{(\Delta s)^3}\right) + x_2\left(\frac{5\alpha}{4\Delta s} + \frac{30\beta}{(\Delta s)^3} + (\kappa - \Pi)\Delta s\right) + x_3\left(-\frac{24\beta}{(\Delta s)^3}\right)$$
$$+ x_4\left(\frac{6\beta}{(\Delta s)^3} - \frac{\alpha}{4\Delta s}\right) + (\Pi - \kappa)x_2^c\Delta s - \frac{\alpha}{\Delta s}\frac{(x_2 - x_1)}{\parallel \frac{\hat{x}_2 - \hat{x}_1}{\Delta s} \parallel} + \frac{\alpha}{4\Delta s}\frac{(x_4 - x_2)}{\parallel \frac{\hat{x}_4 - \hat{x}_2}{2\Delta s} \parallel}.$$
$$- \frac{\kappa l_0(x_2 - x_2^c)\Delta s}{\parallel \hat{x}_2 - \hat{x}_2^c \parallel}$$

For $j = 3$ we have,

$$\frac{\partial \varepsilon_{total}}{\partial x_3} = \frac{\partial \varepsilon_1(\hat{x}_1)}{\partial x_3}\Delta s + \frac{\partial}{\partial x_3}(\varepsilon_2(\hat{x}_2) + \varepsilon_2(\hat{x}_3) + \varepsilon_2(\hat{x}_4))\Delta s.$$

$$\frac{\partial \varepsilon_1(\hat{x}_1)}{\partial x_3}\Delta s = \frac{4\beta}{(\Delta s)^3}(2x_1 - 5x_2 + 4x_3 - x_4)$$

and

$$\frac{\partial \varepsilon_2(\hat{x}_2)}{\partial x_3}\Delta s = \frac{\alpha}{4\Delta s}(x_3 - x_1) + \frac{\beta}{(\Delta s)^3}(x_3 - 2x_2 + x_1) - \frac{\alpha}{4\Delta s}\frac{(x_3 - x_1)}{\parallel \frac{\hat{x}_3 - \hat{x}_1}{2\Delta s} \parallel}$$

$$\frac{\partial \varepsilon_2(\hat{x}_3)}{\partial x_3}\Delta s = -\frac{2\beta}{(\Delta s)^3}(x_4 - 2x_3 + x_2) + (\kappa - \Pi)(x_3 - x_3^c)\Delta s - \frac{\kappa l_0(x_3 - x_3^c)\Delta s}{\parallel \hat{x}_3 - \hat{x}_3^c \parallel}$$

$$\frac{\partial \varepsilon_2(\hat{x}_4)}{\partial x_3}\Delta s = -\frac{\alpha}{4\Delta s}(x_5 - x_3) + \frac{\beta}{(\Delta s)^3}(x_5 - 2x_4 + x_3) + \frac{\alpha}{4\Delta s}\frac{(x_5 - x_3)}{\parallel \frac{\hat{x}_5 - \hat{x}_3}{2\Delta s} \parallel}.$$

Consequently,

$$\begin{aligned}\frac{\partial \varepsilon_{total}}{\partial x_3} &= x_1\left(-\frac{\alpha}{4\Delta s} + \frac{9\beta}{(\Delta s)^3}\right) + x_2\left(-\frac{24\beta}{(\Delta s)^3}\right) + x_3\left(\frac{22\beta}{(\Delta s)^3} + \frac{2\alpha}{4\Delta s} + (\kappa - \Pi)\Delta s\right) \\ &+ x_4\left(-\frac{8\beta}{(\Delta s)^3}\right) + x_5\left(\frac{\beta}{(\Delta s)^3} - \frac{\alpha}{4\Delta s}\right) + (\Pi - \kappa)x_3^c\Delta s - \frac{\alpha}{4\Delta s}\frac{(x_3 - x_1)}{\parallel \frac{\hat{x}_3 - \hat{x}_1}{2\Delta s} \parallel} \\ &+ \frac{\alpha}{4\Delta s}\frac{(x_5 - x_3)}{\parallel \frac{\hat{x}_5 - \hat{x}_3}{2\Delta s} \parallel} - \frac{\kappa l_0(x_3 - x_3^c)\Delta s}{\parallel \hat{x}_3 - \hat{x}_3^c \parallel}\end{aligned}$$

For $j = 4$ we have,

$$\frac{\partial \varepsilon_{total}}{\partial x_4} = \frac{\partial \varepsilon_1(\hat{x}_1)}{\partial x_4}\Delta s + \frac{\partial}{\partial x_4}(\varepsilon_2(\hat{x}_3) + \varepsilon_2(\hat{x}_4) + \varepsilon_2(\hat{x}_5))\Delta s.$$

$$\frac{\partial \varepsilon_1(\hat{x}_1)}{\partial x_4}\Delta s = \frac{-\beta}{(\Delta s)^3}(2x_1 - 5x_2 + 4x_3 - x_4)$$

and

$$\frac{\partial \varepsilon_2(\hat{x}_3)}{\partial x_4}\Delta s = \frac{\alpha}{4\Delta s}(x_4 - x_2) + \frac{\beta}{(\Delta s)^3}(x_4 - 2x_3 + x_2) - \frac{\alpha}{4\Delta s}\frac{(x_4 - x_2)}{\parallel \frac{\hat{x}_4 - \hat{x}_2}{2\Delta s} \parallel}$$

$$\frac{\partial \varepsilon_2(\hat{x}_4)}{\partial x_4}\Delta s = -\frac{2\beta}{(\Delta s)^3}(x_5 - 2x_4 + x_3) + (\kappa - \Pi)(x_4 - x_4^c)\Delta s - \frac{\kappa l_0(x_4 - x_4^c)\Delta s}{\parallel \hat{x}_4 - \hat{x}_4^c \parallel}$$

$$\frac{\partial \varepsilon_2(\hat{x}_5)}{\partial x_4}\Delta s = -\frac{\alpha}{4\Delta s}(x_6 - x_4) + \frac{\beta}{(\Delta s)^3}(x_6 - 2x_5 + x_4) + \frac{\alpha}{4\Delta s}\frac{(x_6 - x_4)}{\parallel \frac{\hat{x}_6 - \hat{x}_4}{2\Delta s} \parallel}$$

Consequently,

$$\begin{aligned}\frac{\partial \varepsilon_{total}}{\partial x_4} &= x_1\left(-\frac{2\beta}{(\Delta s)^3}\right) + x_2\left(\frac{6\beta}{(\Delta s)^3} - \frac{\alpha}{4\Delta s}\right) + x_3\left(-\frac{8\beta}{(\Delta s)^3}\right) \\ &+ x_4\left(\frac{7\beta}{(\Delta s)^3} + \frac{2\alpha}{4\Delta s} + (\kappa - \Pi)\Delta s\right) + x_5\left(-\frac{4\beta}{(\Delta s)^3}\right) + x_6\left(\frac{\beta}{(\Delta s)^3} - \frac{\alpha}{4\Delta s}\right) \\ &+ (\Pi - \kappa)x_4^c\Delta s - \frac{\alpha}{4\Delta s}\frac{(x_4 - x_2)}{\parallel \frac{\hat{x}_4 - \hat{x}_2}{2\Delta s} \parallel} + \frac{\alpha}{4\Delta s}\frac{(x_6 - x_4)}{\parallel \frac{\hat{x}_6 - \hat{x}_4}{2\Delta s} \parallel} - \frac{\kappa l_0(x_4 - x_4^c)\Delta s}{\parallel \hat{x}_4 - \hat{x}_4^c \parallel}.\end{aligned}$$

For $j = 5 \cdots n - 3$ we have,

$$\begin{aligned}\frac{\partial \varepsilon_{total}}{\partial x_j} &= \frac{\partial}{\partial x_j}(\varepsilon_2(\hat{x}_{j-1}) + \varepsilon_2(\hat{x}_j) + \varepsilon_2(\hat{x}_{j+1}))\Delta s. \\ \frac{\partial \varepsilon_2(\hat{x}_{j-1})}{\partial x_j}\Delta s &= \frac{\alpha}{4\Delta s}(x_j - x_{j-2}) + \frac{\beta}{(\Delta s)^3}(x_j - 2x_{j-1} + x_{j-2}) - \frac{\alpha}{4\Delta s}\frac{(x_j - x_{j-2})}{\parallel \frac{\hat{x}_j - \hat{x}_{j-2}}{2\Delta s} \parallel} \\ \frac{\partial \varepsilon_2(\hat{x}_j)}{\partial x_j}\Delta s &= -\frac{2\beta}{(\Delta s)^3}(x_{j+1} - 2x_j + x_{j-1})(\kappa - \Pi)(x_j - x_j^c)\Delta s - \frac{\kappa l_0(x_4 - x_4^c)\Delta s}{\parallel \hat{x}_4 - \hat{x}_4^c \parallel} \\ \frac{\partial \varepsilon_2(\hat{x}_{j+1})}{\partial x_j}\Delta s &= -\frac{\alpha}{4\Delta s}(x_{j+2} - x_j) + \frac{\beta}{(\Delta s)^3}(x_{j+2} - 2x_{j+1} + x_j) + \frac{\alpha}{4\Delta s}\frac{(x_{j+2} - x_j)}{\parallel \frac{\hat{x}_{j+2} - \hat{x}_j}{2\Delta s} \parallel}.\end{aligned}$$

Consequently,

$$\begin{aligned}\frac{\partial \varepsilon_{total}}{\partial x_j} &= x_{j-2}\left(\frac{\beta}{(\Delta s)^3} - \frac{\alpha}{4\Delta s}\right) + x_{j-1}\left(-\frac{4\beta}{(\Delta s)^3}\right) + x_j\left(\frac{6\beta}{(\Delta s)^3} + \frac{2\alpha}{4\Delta s} + (\kappa - \Pi)\Delta s\right) \\ &+ x_{j+1}\left(-\frac{4\beta}{(\Delta s)^3}\right) + x_{j+2}\left(\frac{\beta}{(\Delta s)^3} - \frac{\alpha}{4\Delta s}\right) \\ &+ (\Pi - \kappa)x_j^c\Delta s - \frac{\alpha}{4\Delta s}\frac{(x_j - x_{j-2})}{\parallel \frac{\hat{x}_j - \hat{x}_{j-2}}{2\Delta s} \parallel} + \frac{\alpha}{4\Delta s}\frac{(x_{j+2} - x_j)}{\parallel \frac{\hat{x}_{j+2} - \hat{x}_j}{2\Delta s} \parallel} - \frac{\kappa l_0(x_j - x_j^c)\Delta s}{\parallel \hat{x}_j - \hat{x}_j^c \parallel}\end{aligned}$$

Now towards the end of the nodes we have,

$$\frac{\partial \varepsilon_{total}}{\partial x_{n-3}} = \frac{\partial}{\partial x_{n-3}}(\varepsilon_2(\hat{x}_{n-4}) + \varepsilon_2(\hat{x}_{n-3}) + \varepsilon_2(\hat{x}_{n-2}) + \varepsilon_3(\hat{x}_n))\Delta s$$
$$= x_{n-5}\left(\frac{\beta}{(\Delta s)^3} - \frac{\alpha}{4\Delta s}\right) + x_{n-4}\left(-\frac{4\beta}{(\Delta s)^3}\right) + x_{n-3}\left(\frac{7\beta}{(\Delta s)^3} + \frac{2\alpha}{4\Delta s} + (\kappa - \Pi)\Delta s\right)$$
$$+ x_{n-2}\left(-\frac{8\beta}{(\Delta s)^3}\right) + x_{n-1}\left(\frac{6\beta}{(\Delta s)^3} - \frac{\alpha}{4\Delta s}\right) + x_n\left(-\frac{2\beta}{(\Delta s)^3}\right)$$
$$+ (\Pi - \kappa)x^c_{n-3}\Delta s - \frac{\alpha}{4\Delta s}\frac{(x_{n-3} - x_{n-5})}{\parallel \frac{\hat{x}_{n-3}-\hat{x}_{n-5}}{2\Delta s} \parallel} + \frac{\alpha}{4\Delta s}\frac{(x_{n-1} - x_{n-3})}{\parallel \frac{\hat{x}_{n-1}-\hat{x}_{n-3}}{2\Delta s} \parallel} - \frac{\kappa l_0(x_{n-3} - x^c_{n-3})\Delta s}{\parallel \hat{x}_{n-3} - \hat{x}^c_{n-3} \parallel},$$

$$\frac{\partial \varepsilon_{total}}{\partial x_{n-2}} = \frac{\partial}{\partial x_{n-2}}(\varepsilon_2(\hat{x}_{n-3}) + \varepsilon_2(\hat{x}_{n-2}) + \varepsilon_2(\hat{x}_{n-1}) + \varepsilon_3(\hat{x}_n))\Delta s$$
$$= x_{n-4}\left(\frac{\beta}{(\Delta s)^3} - \frac{\alpha}{4\Delta s}\right) + x_{n-3}\left(-\frac{8\beta}{(\Delta s)^3}\right) + x_{n-2}\left(\frac{14\beta}{(\Delta s)^3} + \frac{2\alpha}{4\Delta s} + (\kappa - \Pi)\Delta s\right)$$
$$+ x_{n-1}\left(-\frac{24\beta}{(\Delta s)^3}\right) + x_n\left(\frac{9\beta}{(\Delta s)^3} - \frac{\alpha}{4\Delta s}\right)$$
$$+ (\Pi - \kappa)x^c_{n-2}\Delta s - \frac{\alpha}{4\Delta s}\frac{(x_{n-2} - x_{n-4})}{\parallel \frac{\hat{x}_{n-2}-\hat{x}_{n-4}}{2\Delta s} \parallel} + \frac{\alpha}{4\Delta s}\frac{(x_n - x_{n-2})}{\parallel \frac{\hat{x}_n-\hat{x}_{n-2}}{2\Delta s} \parallel} - \frac{\kappa l_0(x_{n-2} - x^c_{n-2})\Delta s}{\parallel \hat{x}_{n-2} - \hat{x}^c_{n-2} \parallel},$$

$$\frac{\partial \varepsilon_{total}}{\partial x_{n-1}} = \frac{\partial}{\partial x_{n-1}}(\varepsilon_2(\hat{x}_{n-2}) + \varepsilon_2(\hat{x}_{n-1})) + \varepsilon_3(\hat{x}_n))\Delta s$$
$$= x_{n-3}\left(-\frac{4\beta}{(\Delta s)^3}\right) + x_{n-2}\left(-\frac{22\beta}{(\Delta s)^3}\right) + x_{n-1}\left(\frac{30\beta}{(\Delta s)^3} - \frac{\alpha}{\Delta s} + (\kappa - \Pi)\Delta s\right)$$
$$+ x_n\left(-\frac{12\beta}{(\Delta s)^3} - \frac{\alpha}{\Delta s}\right) + (\Pi - \kappa)x^c_{n-1}\Delta s - \frac{\alpha}{4\Delta s}\frac{(x_{n-1} - x_{n-3})}{\parallel \frac{\hat{x}_{n-1}-\hat{x}_{n-3}}{2\Delta s} \parallel}$$
$$+ \frac{\alpha}{\Delta s}\frac{(x_n - x_{n-1})}{\parallel \frac{\hat{x}_n-\hat{x}_{n-1}}{\Delta s} \parallel} - \frac{\kappa l_0(x_{n-1} - x^c_{n-1})\Delta s}{\parallel \hat{x}_{n-1} - \hat{x}^c_{n-1} \parallel},$$

and finally for $j = n$ we have,

$$\begin{aligned}
\frac{\partial \varepsilon_{total}}{\partial x_n} &= \frac{\partial}{\partial x_n}(\varepsilon_2(\hat{x}_{n-1})) + \varepsilon_3(\hat{x}_n))\Delta s \\
&= x_{n-3}\left(-\frac{2\beta}{(\Delta s)^3}\right) + x_{n-2}\left(\frac{9\beta}{(\Delta s)^3} - \frac{\alpha}{4\Delta s}\right) + x_{n-1}\left(-\frac{12\beta}{(\Delta s)^3} - \frac{\alpha}{\Delta s}\right) \\
&+ x_n\left(\frac{5\beta}{(\Delta s)^3} + \frac{5\alpha}{\Delta s}(\kappa - \Pi)\Delta s\right) + (\Pi - \kappa)x_n^c \Delta s - \frac{\alpha}{4\Delta s}\frac{(x_n - x_{n-2})}{\parallel \frac{\hat{x}_n - \hat{x}_{n-2}}{2\Delta s} \parallel} \\
&- \frac{\alpha}{\Delta s}\frac{(x_n - x_{n-1})}{\parallel \frac{\hat{x}_n - \hat{x}_{n-1}}{\Delta s} \parallel} - \frac{\kappa l_0 (x_n - x_n^c)\Delta s}{\parallel \hat{x}_n - \hat{x}_n^c \parallel}.
\end{aligned}$$

References

[1] John H. Mathews and Kurtis D. Fink, *Numerical methods using matlab*, 4th ed., Pearson Education, Inc, 2004.
