# Supplementary material for: A role for myosin II clusters and membrane energy in cortex rupture for Dictyostelium discoideum
Source: PLoS One. 2022 Apr 25;17(4):e0265380. doi: 10.1371/journal.pone.0265380 (PMC9037949; doi:10.1371/journal.pone.0265380)
Supplement: S1 File — Microscopy images of Ax2 D. discoideum cells expressing LifeAct-GFP, boundary images with bleb shoulder points obtained from bleb detection algorithm with energy predictions of nucleation sites and energy profile over bleb neck. (PDF) [file pone.0265380.s008.pdf]

Bleb Nucleation Predictions for Experiment 0117

Bleb0117009-08

Out[•]= {

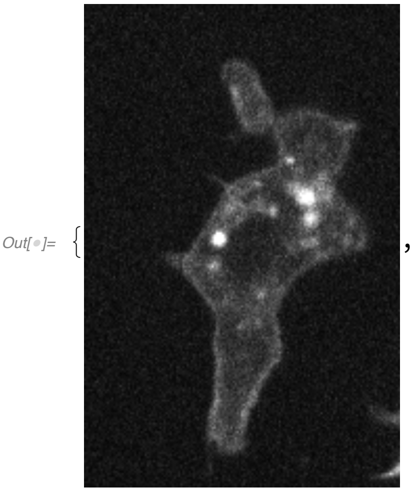

,

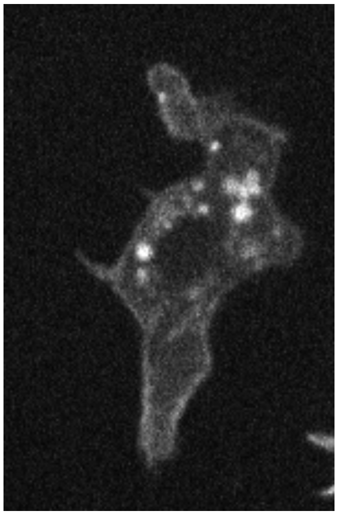

,

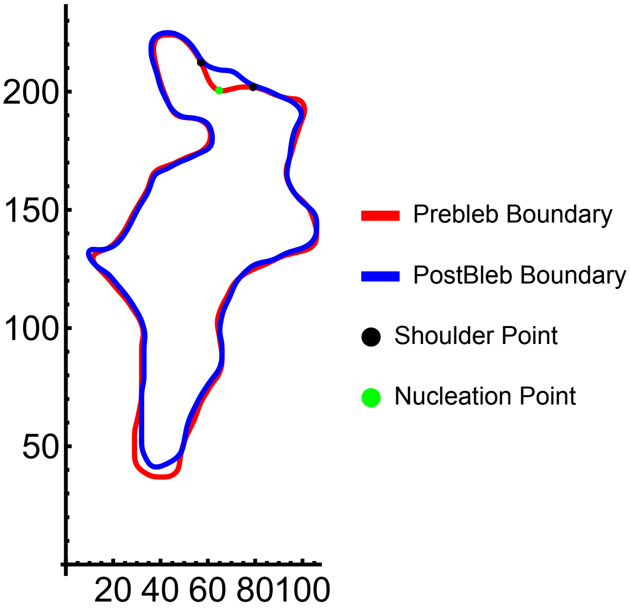
200
150
100
50
20 40 60 80100
Prebleb Boundary
PostBleb Boundary
Shoulder Point
Nucleation Point

,

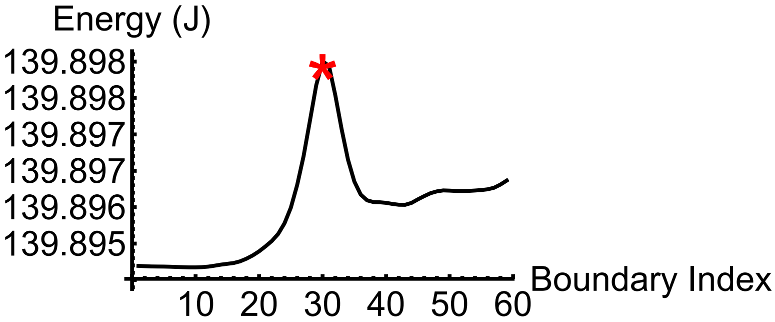
Energy (J)
139.898
139.898
139.897
139.897
139.896
139.895
10 20 30 40 50 60
Boundary Index

}

Bleb0117009-09

Out[•]= {

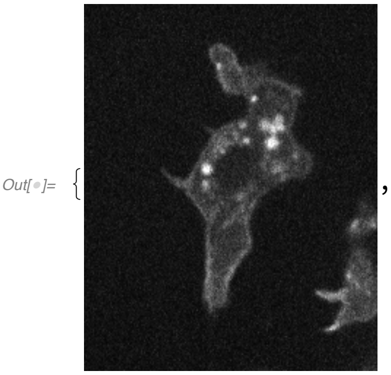

,

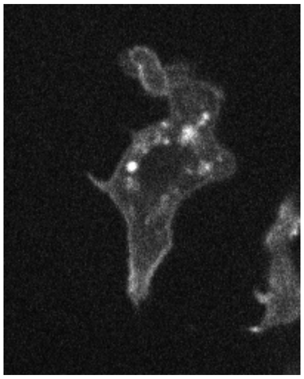

,

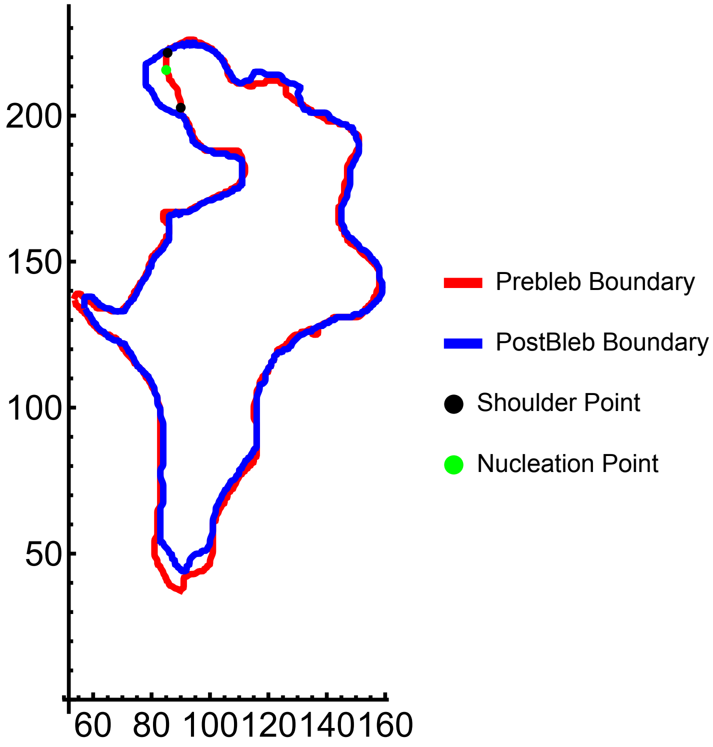

,

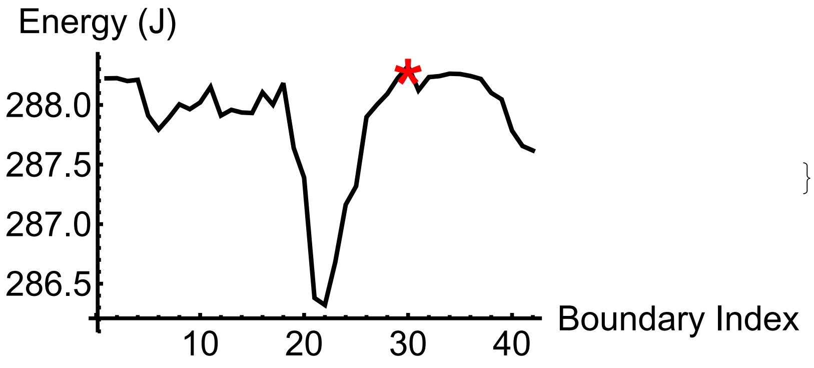

}

Bleb0117009-11

Out[•]= {

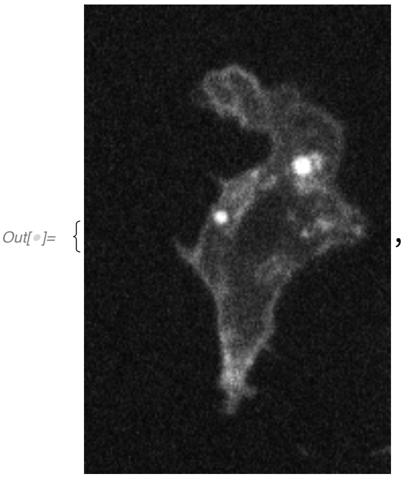

,

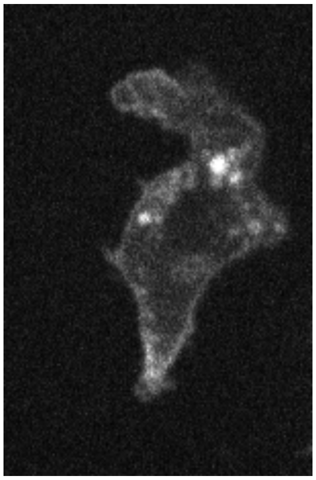

,

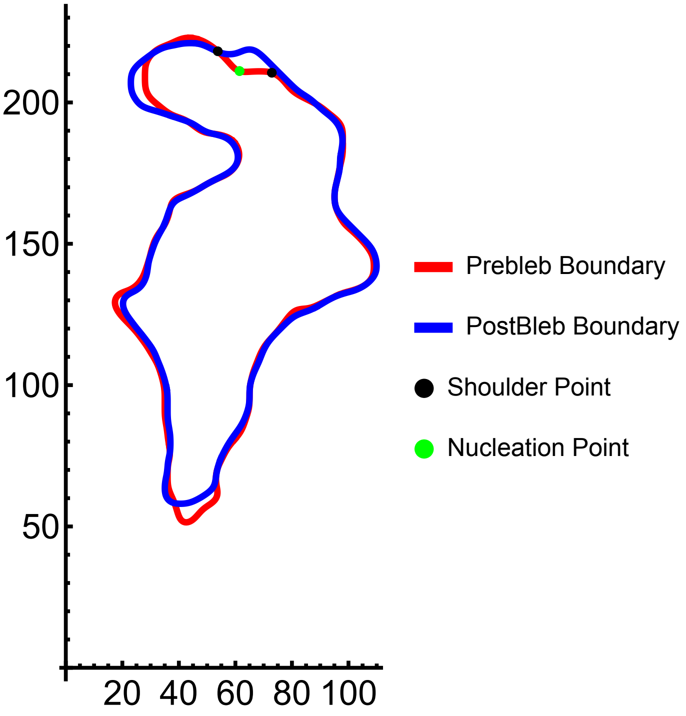
200
150
100
50
20
40
60
80
100
Prebleb Boundary
PostBleb Boundary
Shoulder Point
Nucleation Point

,

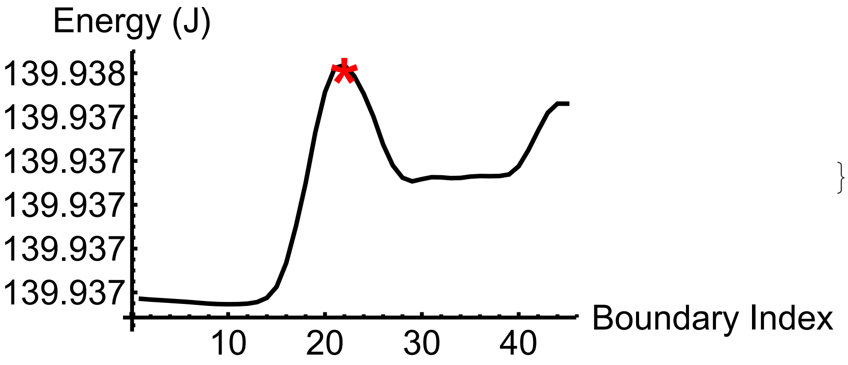
Energy (J)
139.938
139.937
139.937
139.937
139.937
139.937
10
20
30
40
Boundary Index

}

Bleb0117012-01

Out[]= { 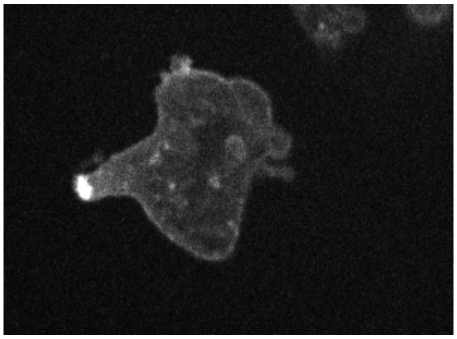 , 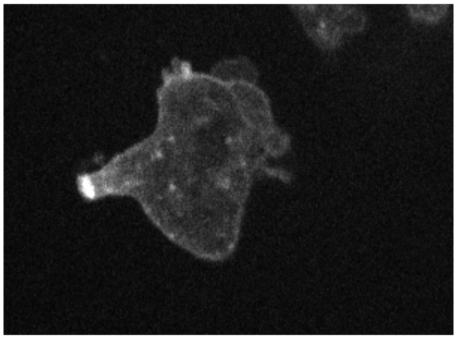 ,

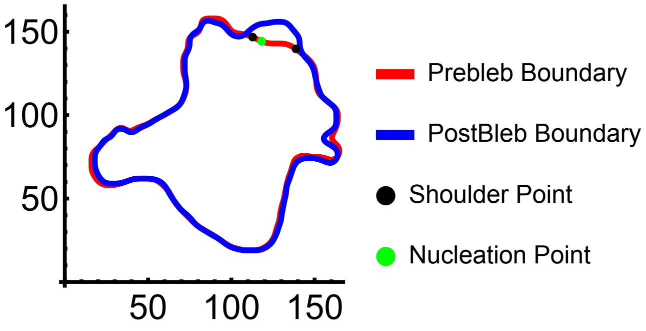

,

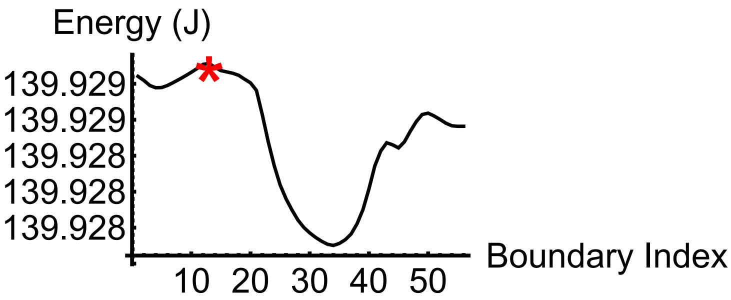

}

Bleb0117012-02

Out[•]= {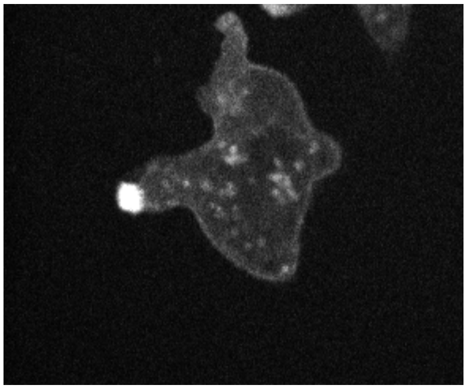, 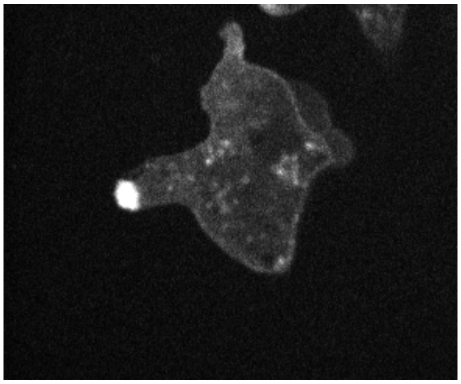,

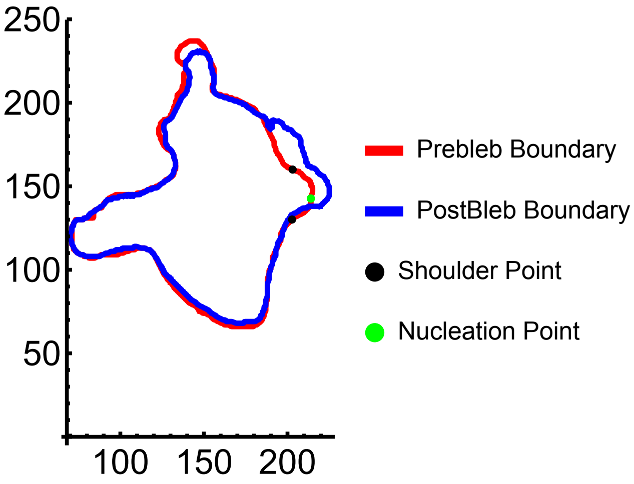

,

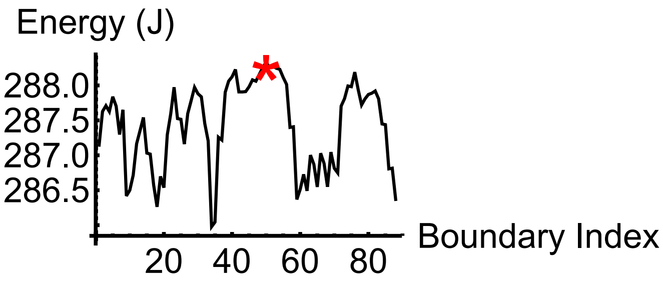

}

Bleb0117012-05

Out[ ]= {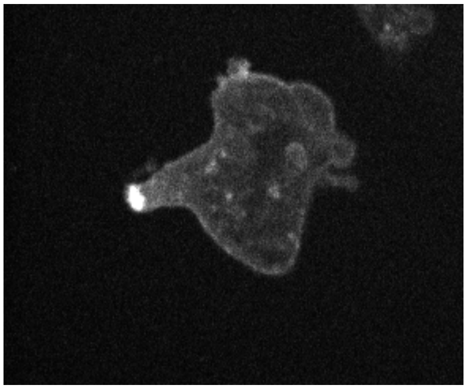, 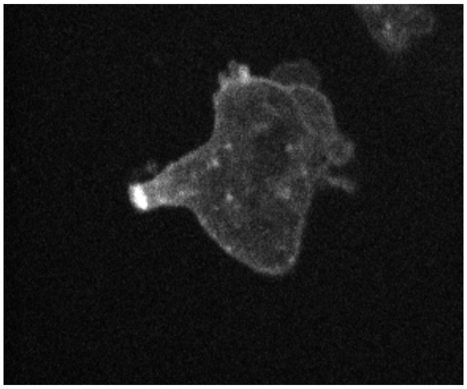,

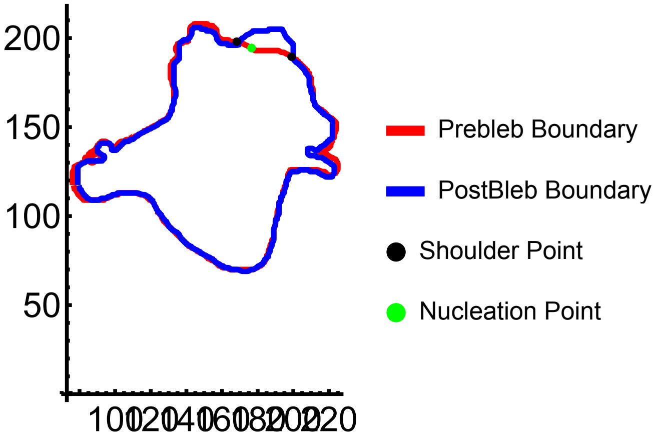

,

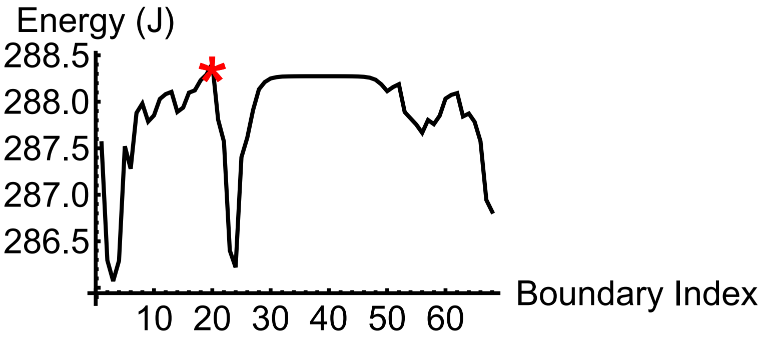

}

Bleb0117015A-04

Out[•]= {

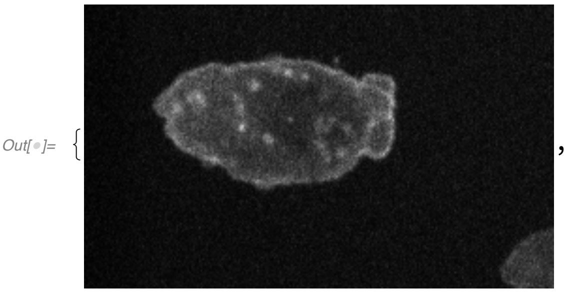

,

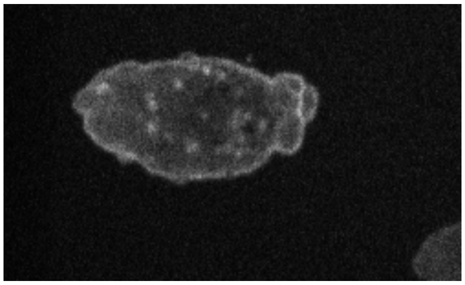

,

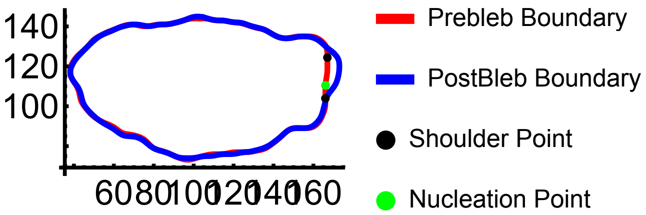

,

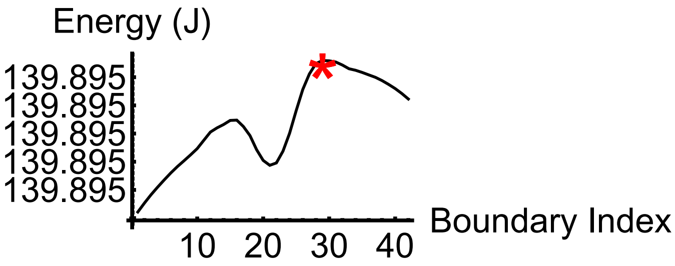

}

Bleb0117015A-05

Out[•]= {

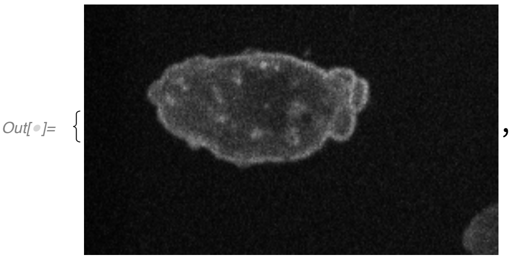

,

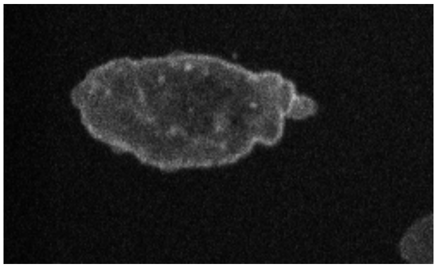

,

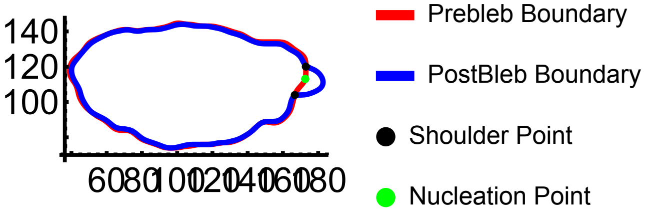

,

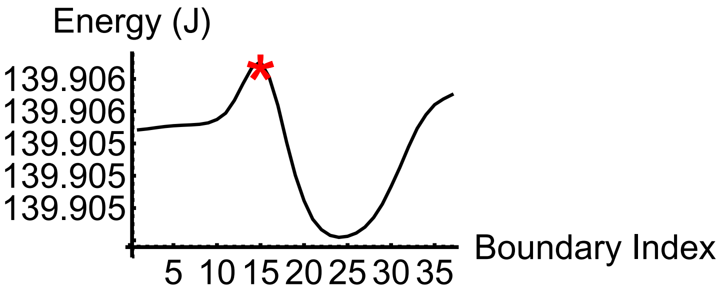

}

Bleb0117015A-06

Out[•]= {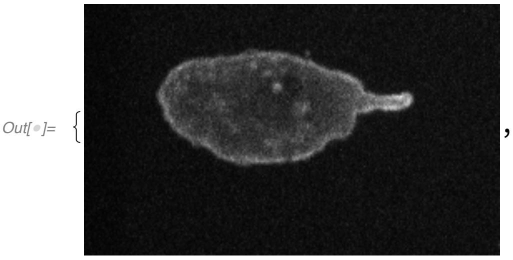, 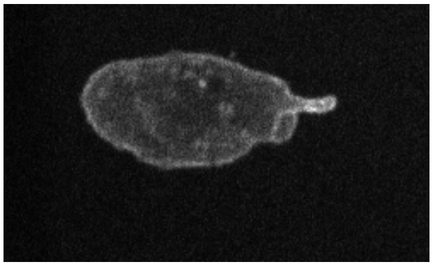,

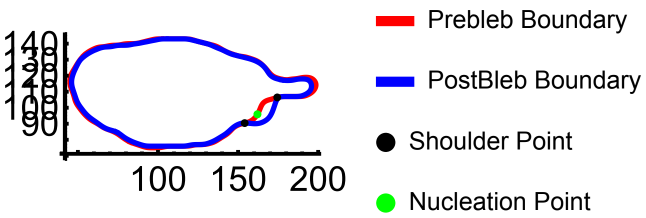

,

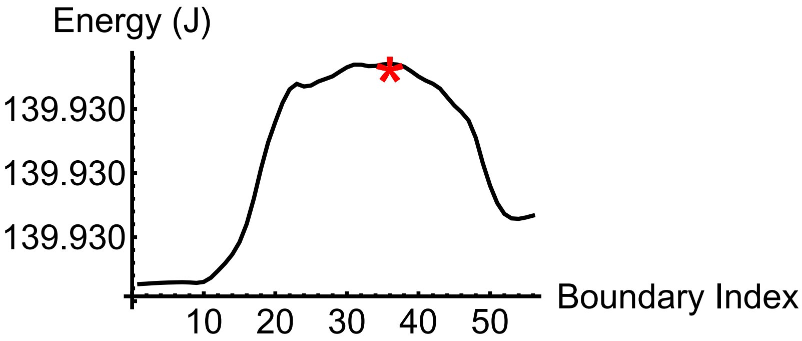

}

Bleb0117015C-02

Out[ ]= {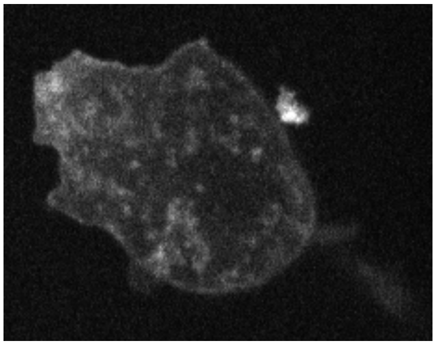, 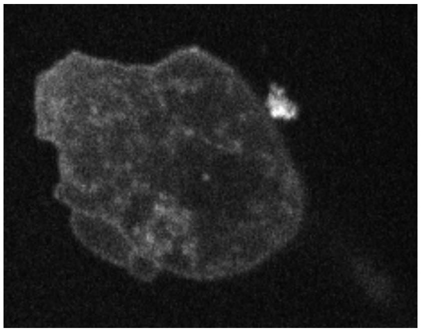,

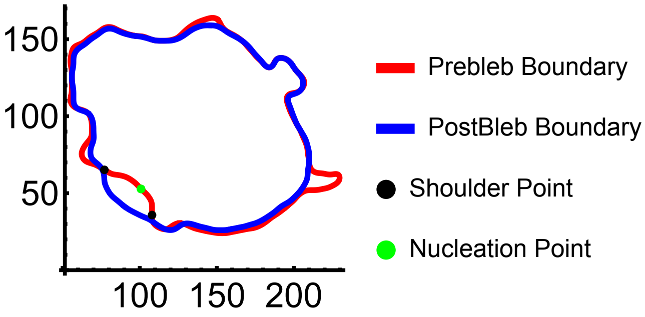

,

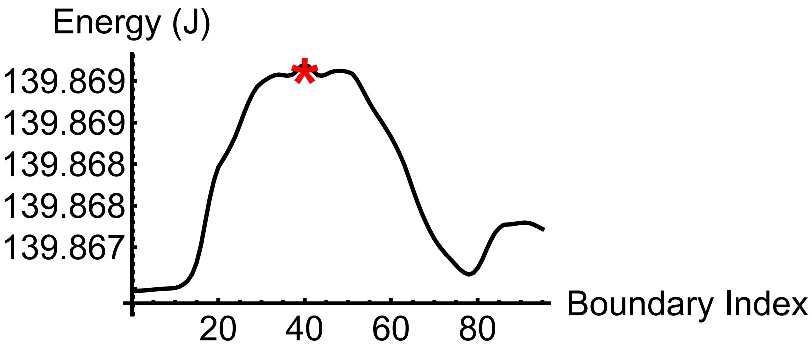

}

Bleb0117015C-04

Out[ ]= {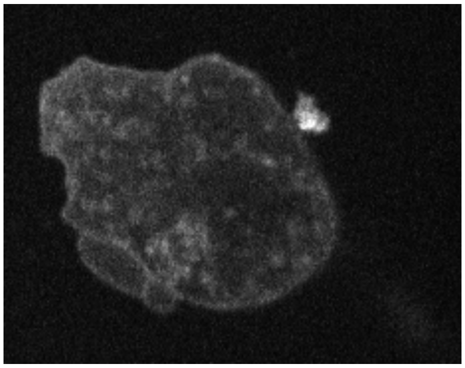, 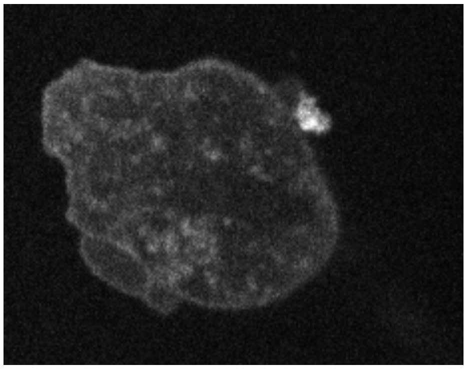, 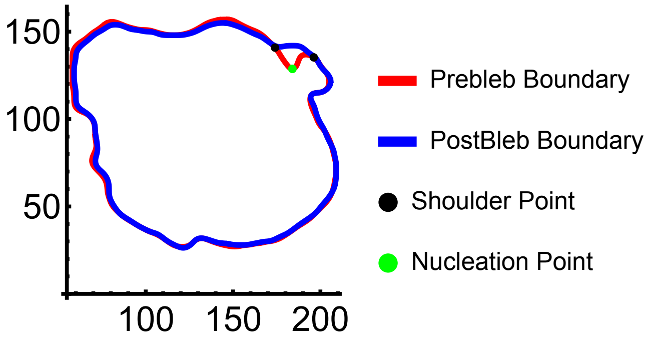

, 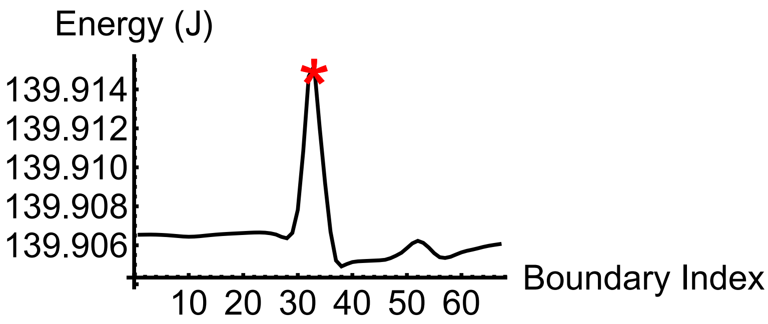

}

Bleb0117017-01

Out[•]= {

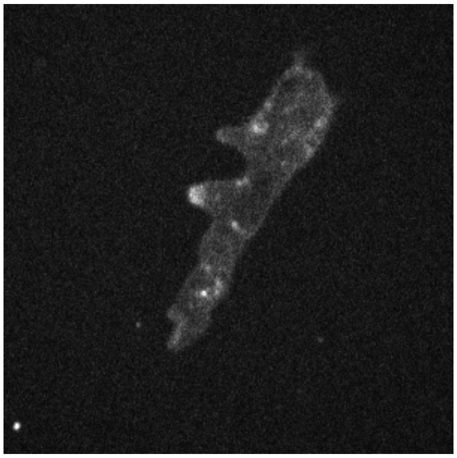

,

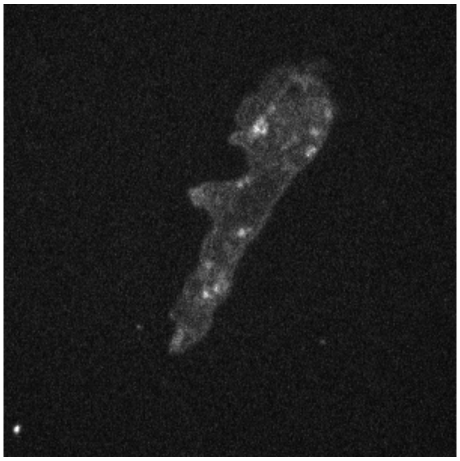

,

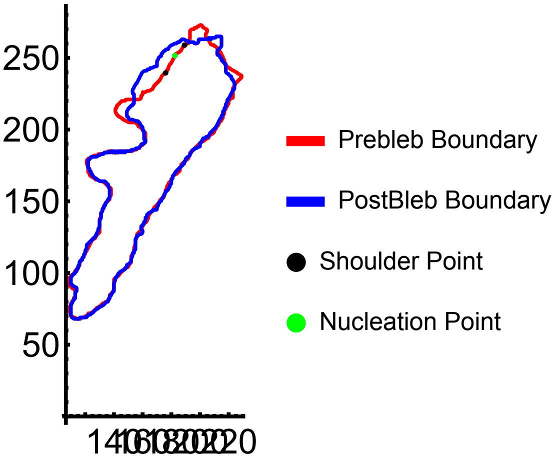

,

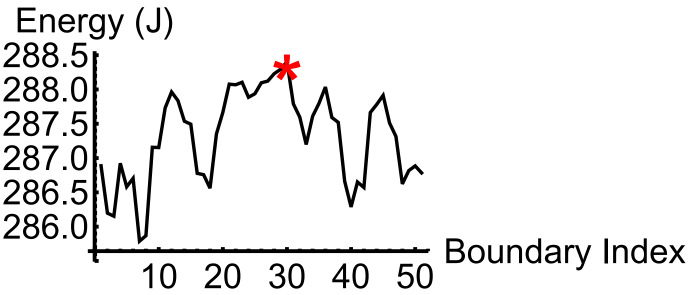

}

Bleb0117017-02

Out[ ]= { 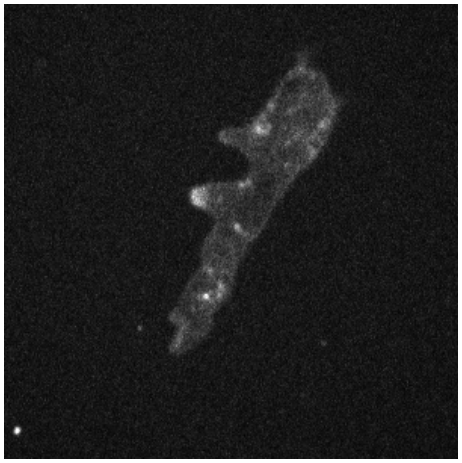 , 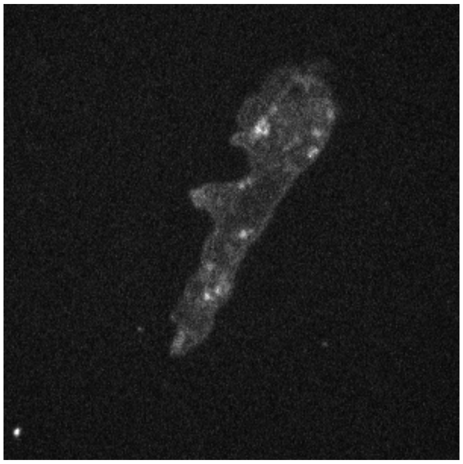 ,

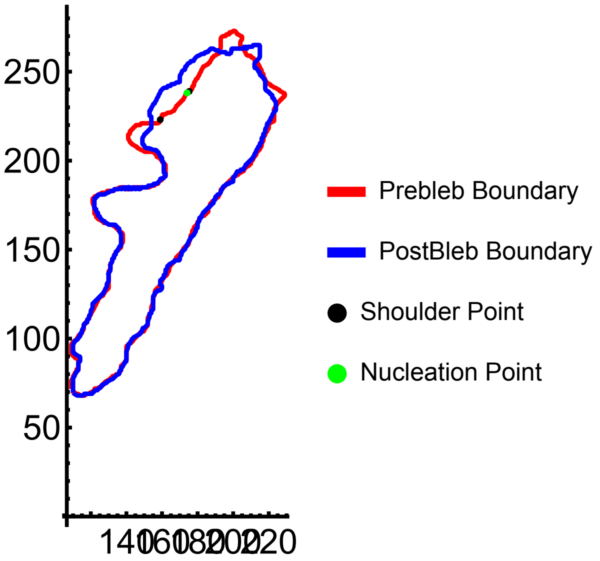

,

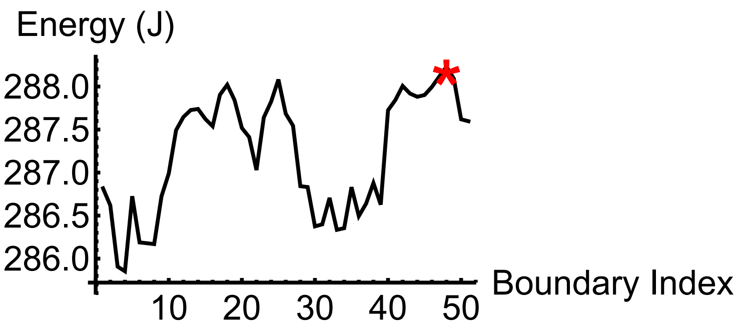

}

Bleb0117017-03

Out[•]= {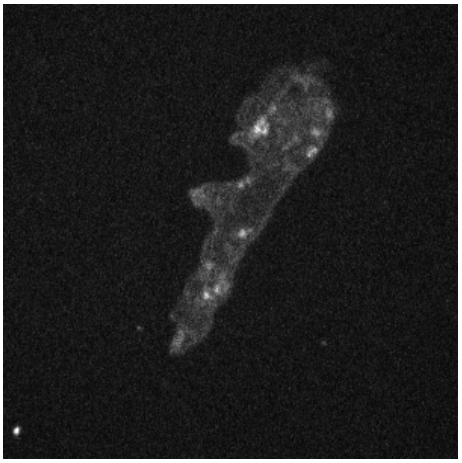, 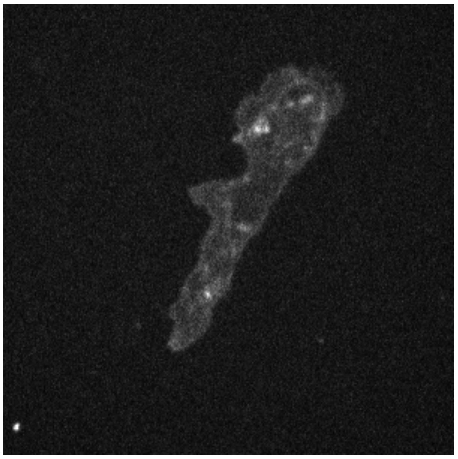,

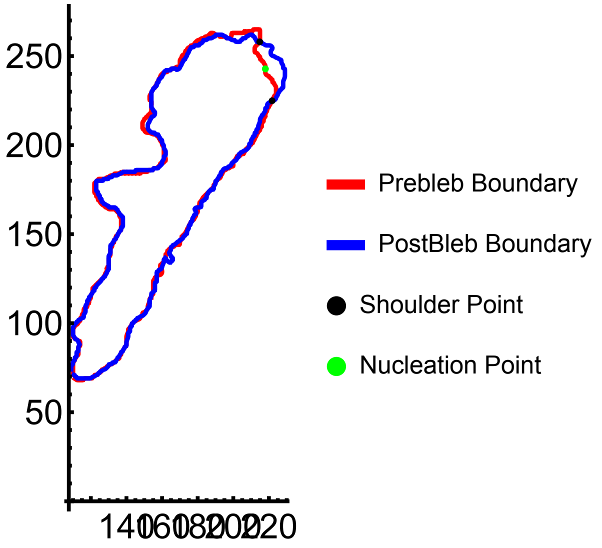

,

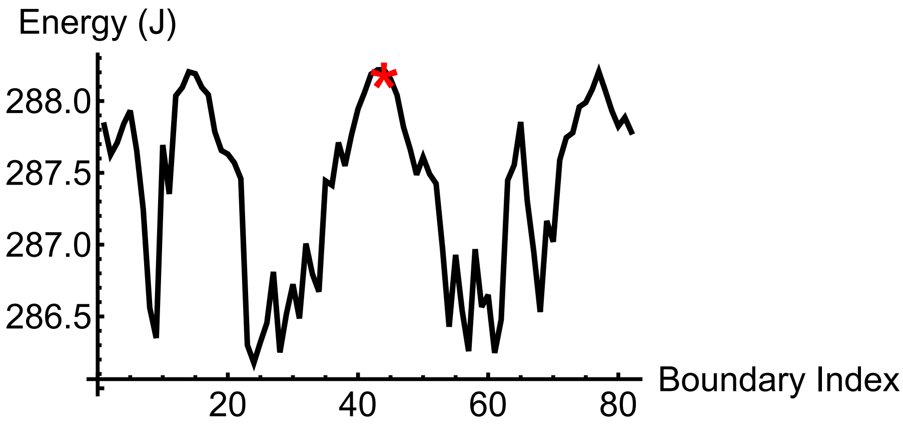

}

Bleb0117017-05

Out[ ]= {

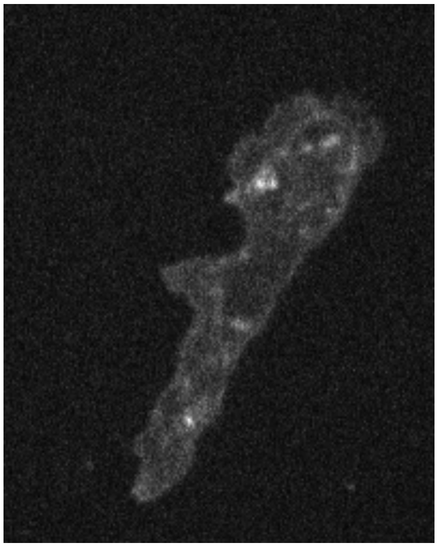

,

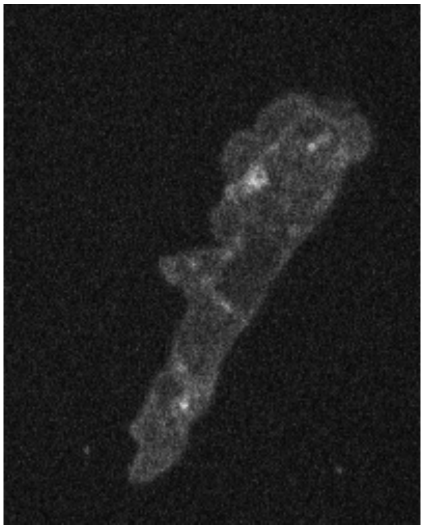

,

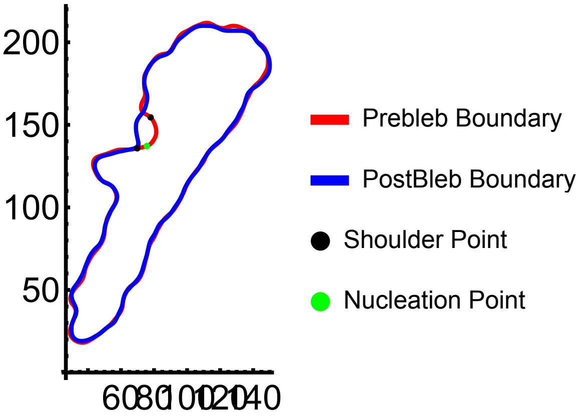

,

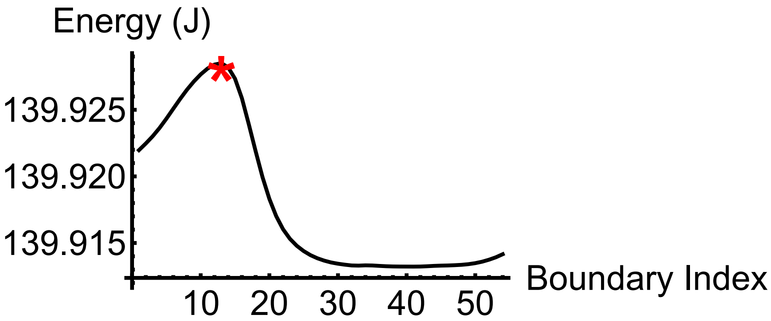

}

Bleb0117017-07

Out[•]= {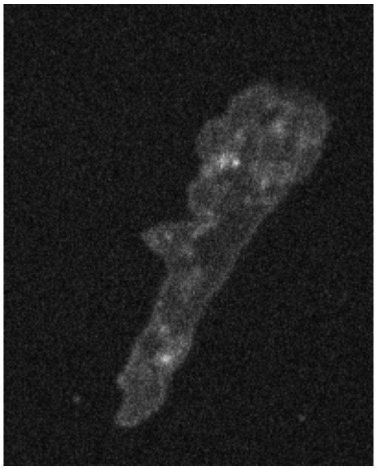, 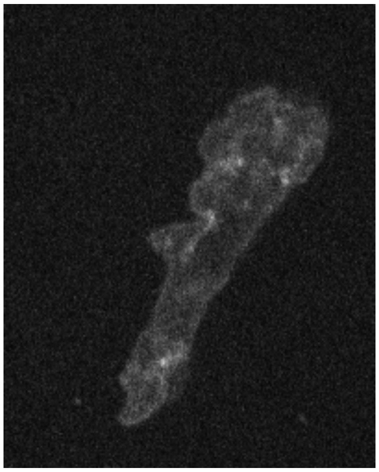,

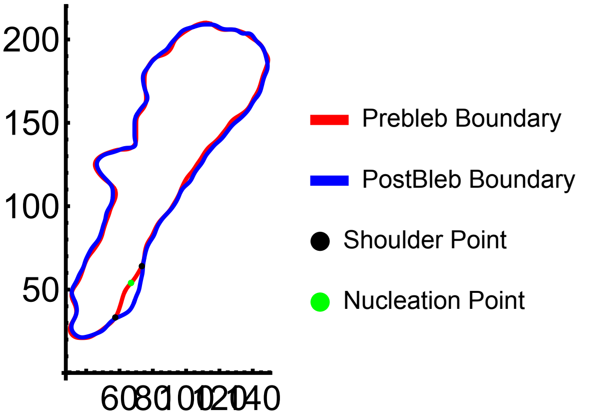

,

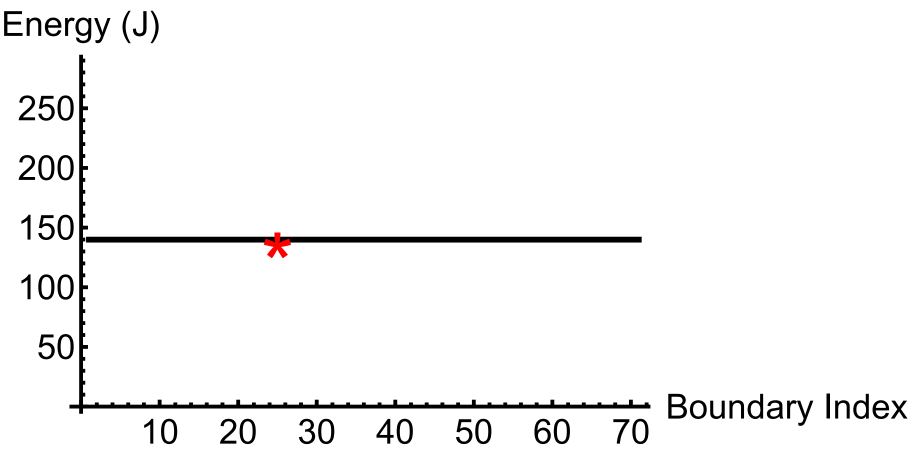

}

Bleb0117017-09

Out[ ]= {

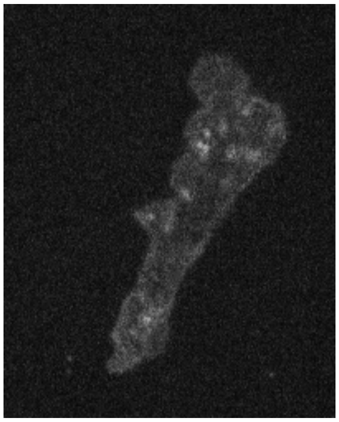

,

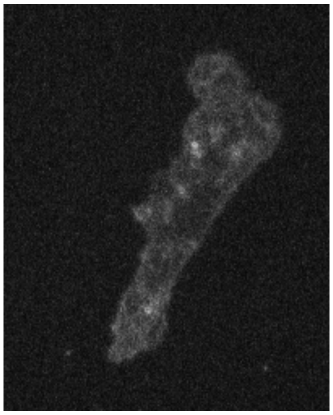

,

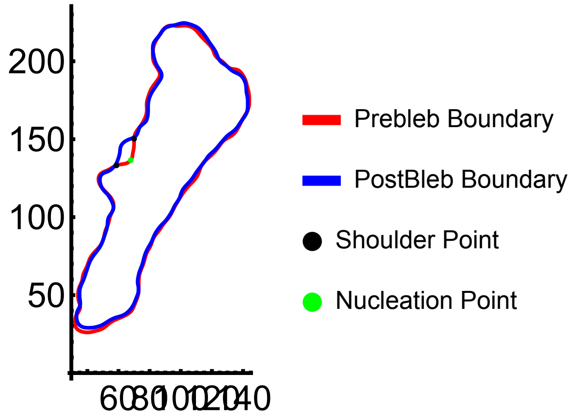

,

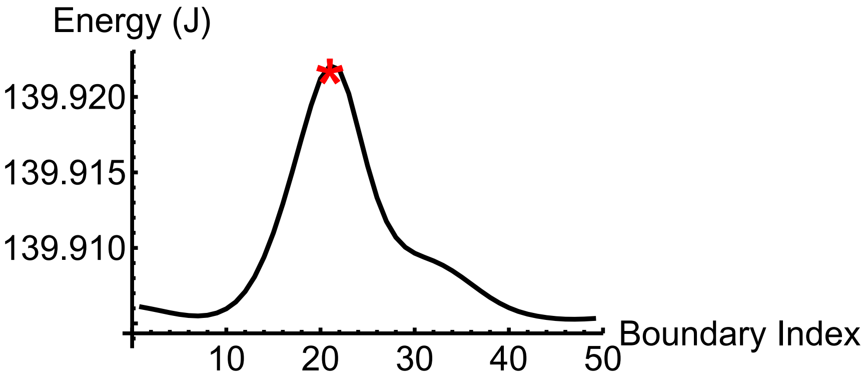

}

Bleb0117018-01

Out[•]= {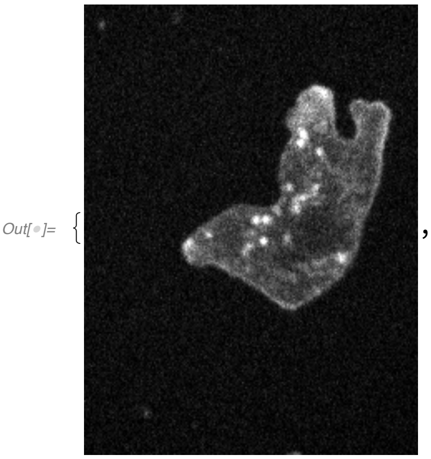, 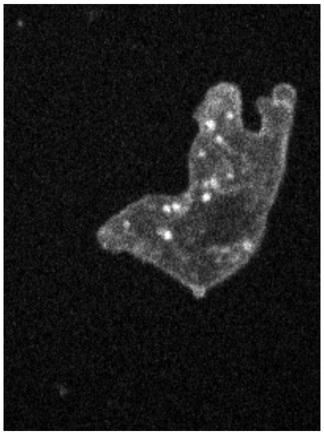,

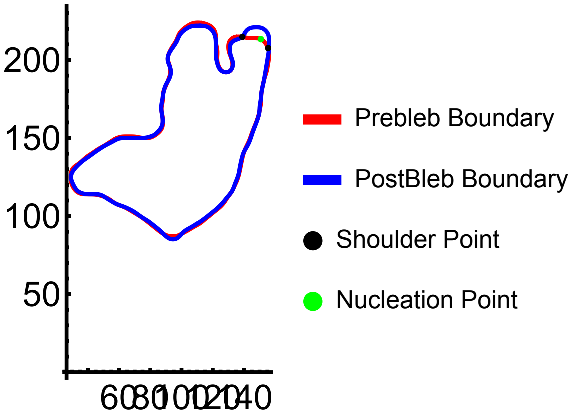

,

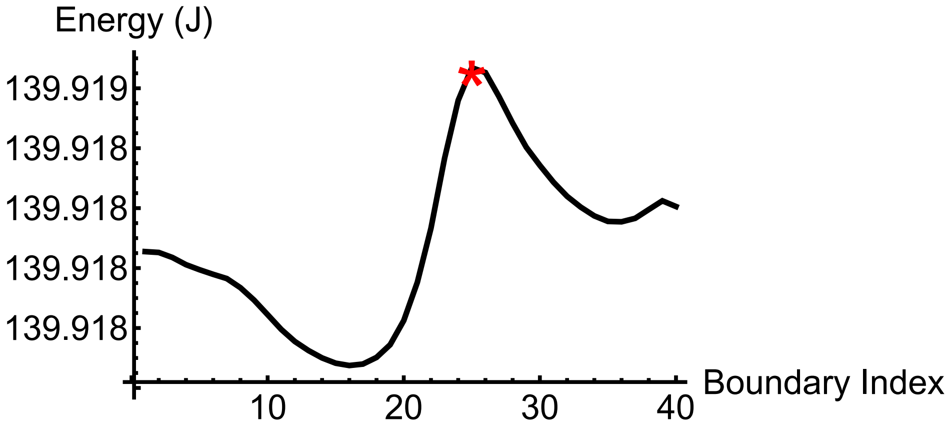

}

Bleb0117018-02

Out[•]= {

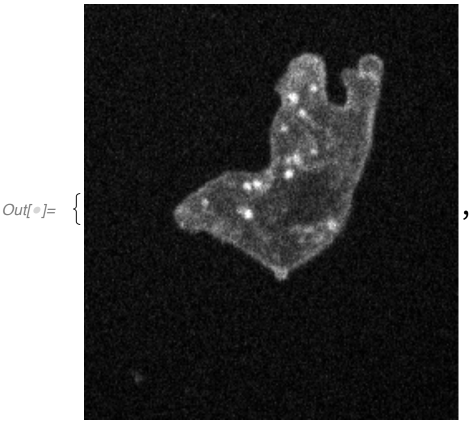

,

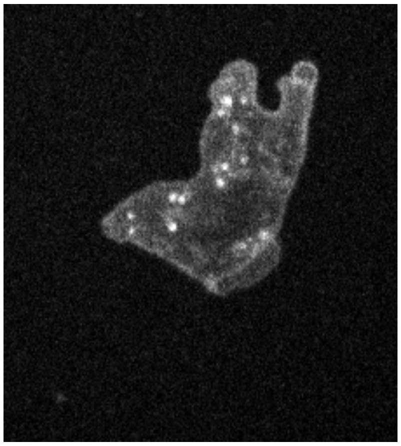

,

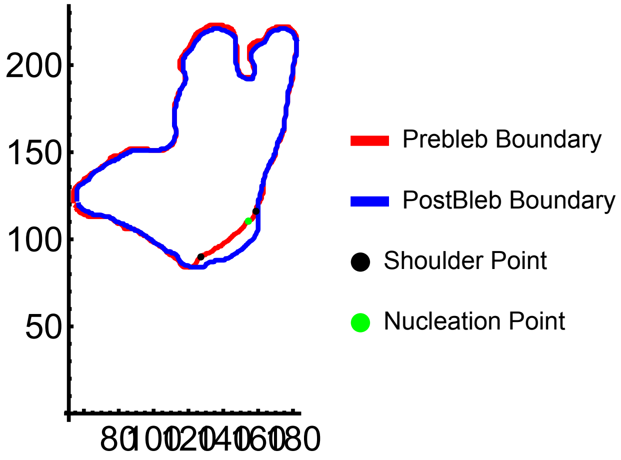

,

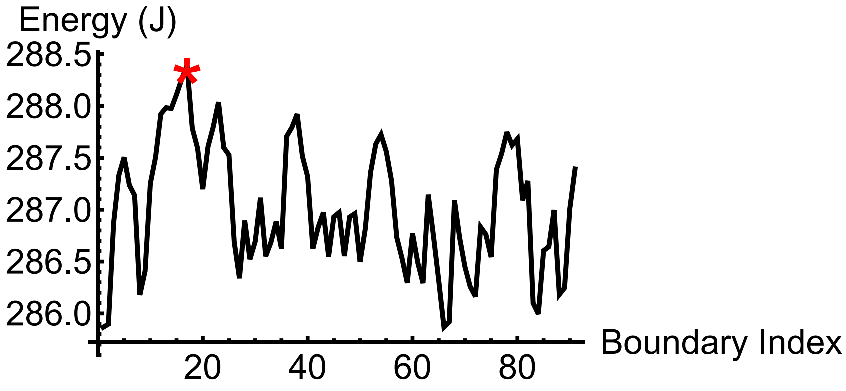

}

Bleb0117018-03

Out[•]= {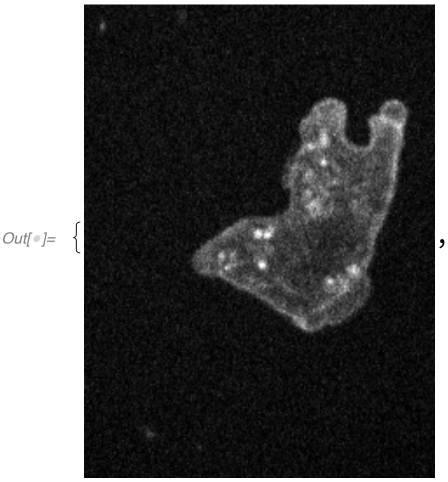, 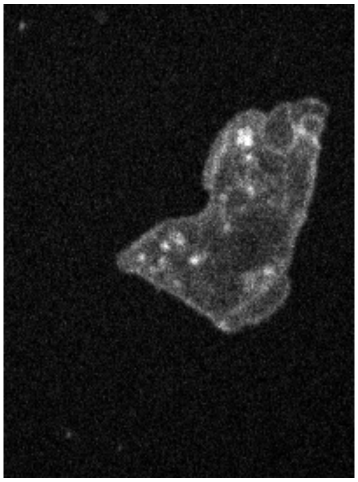,

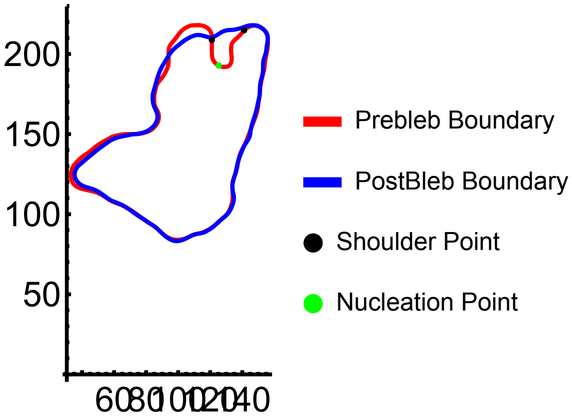

,

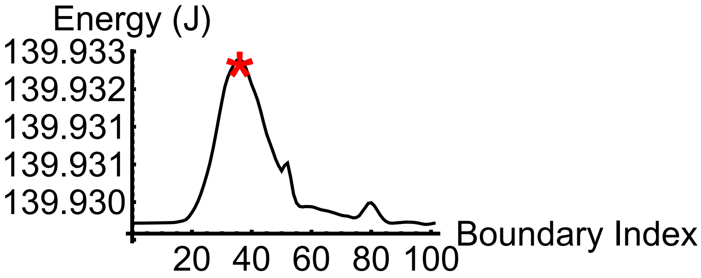

}

Bleb0117018-05

Out[ ]= {

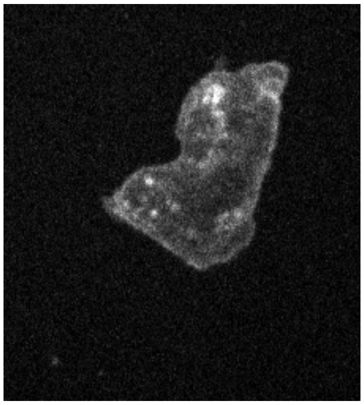

,

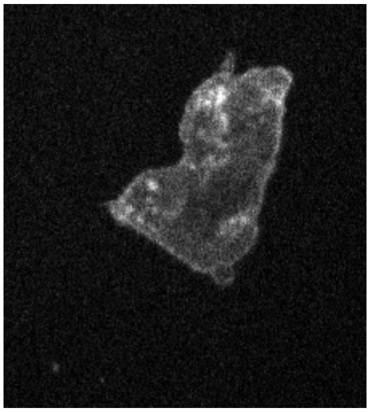

,

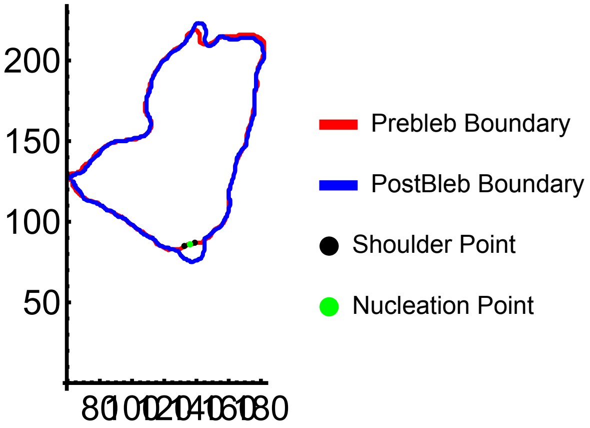

,

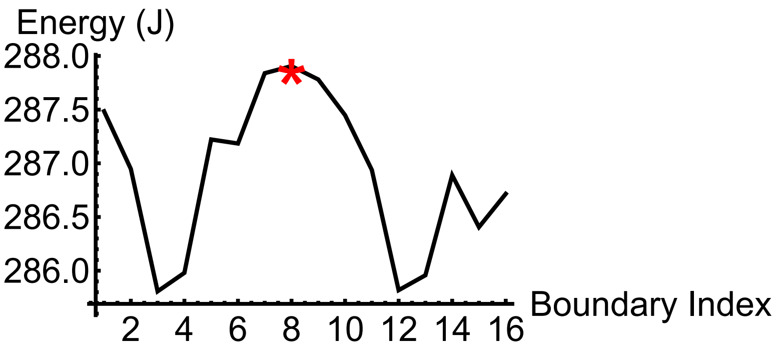

}

Bleb0117019-01

Out[ ]= {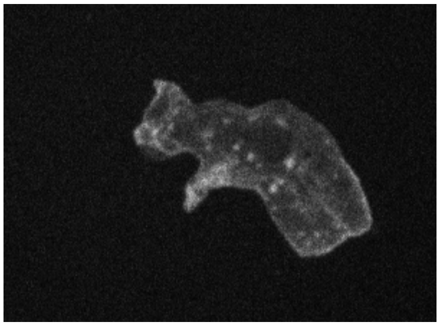, 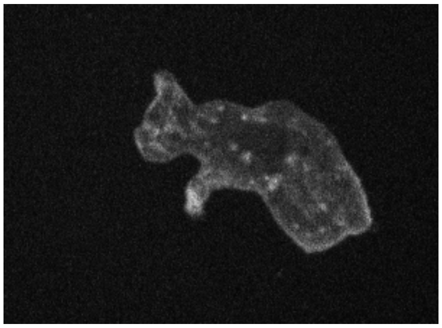,

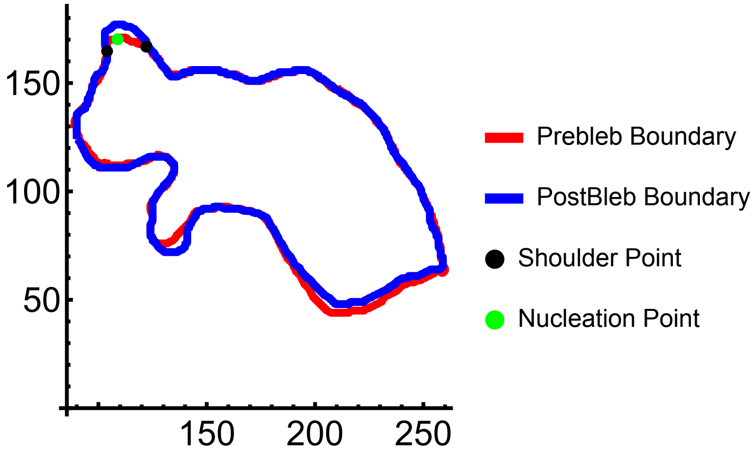

,

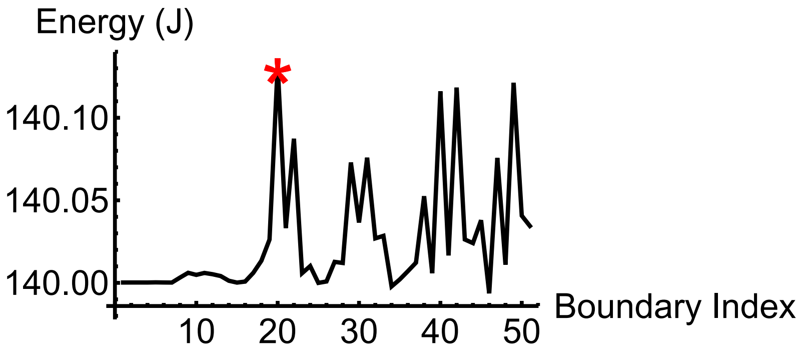

}

Bleb0117019-03

Out[ ]= { 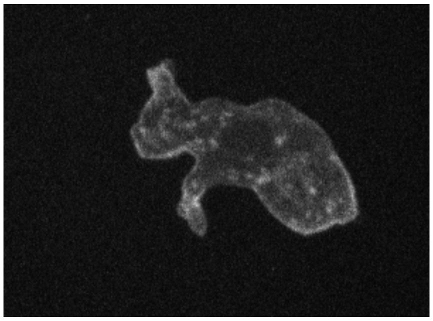 , 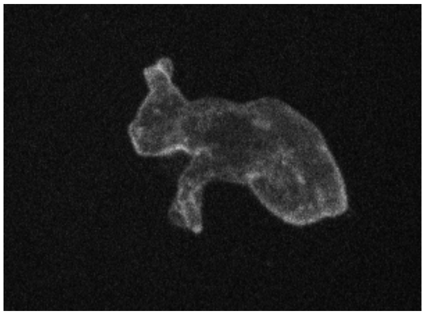 ,

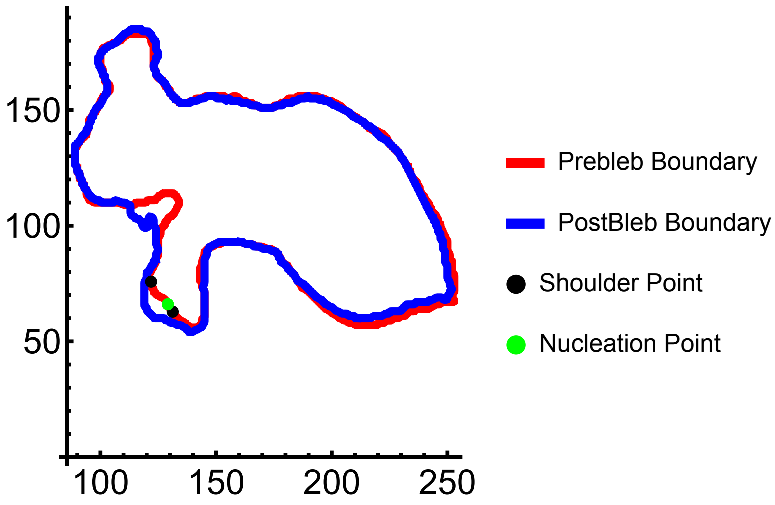

,

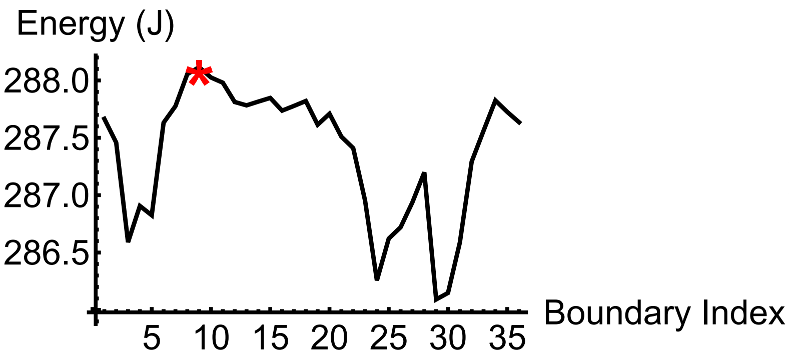

}

Bleb0117019-05

Out[•]= {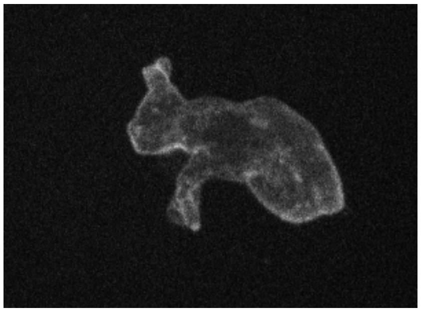, 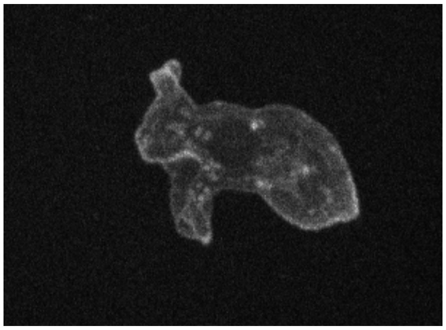,

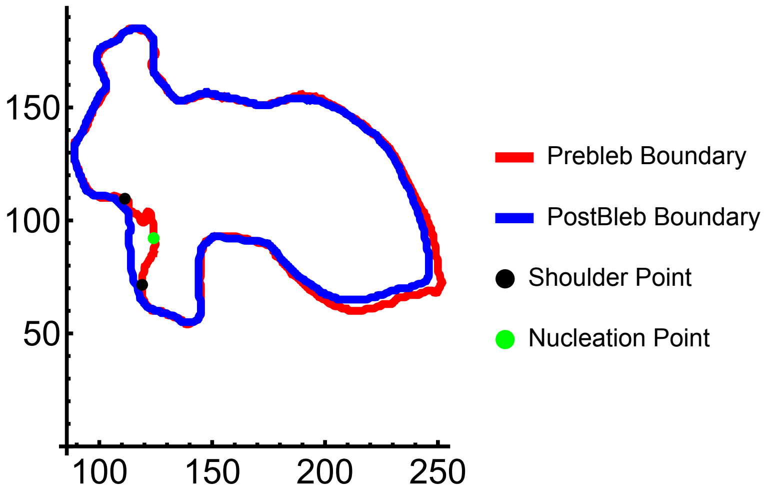

,

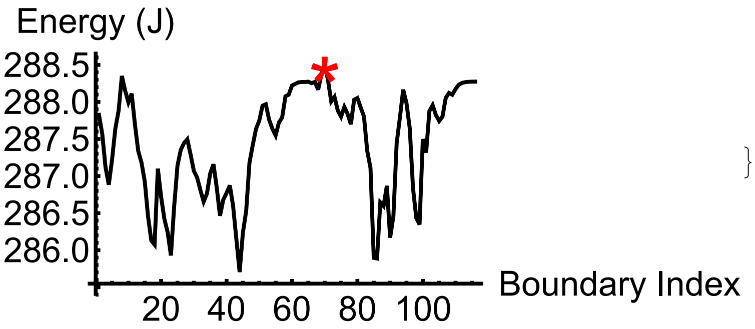

}

Bleb0117019-08

Out[ ]= {

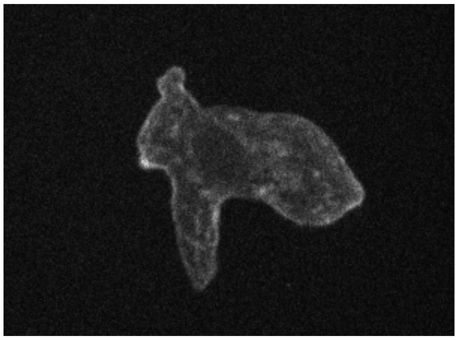

,

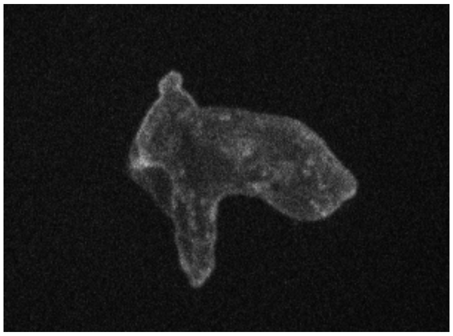

,

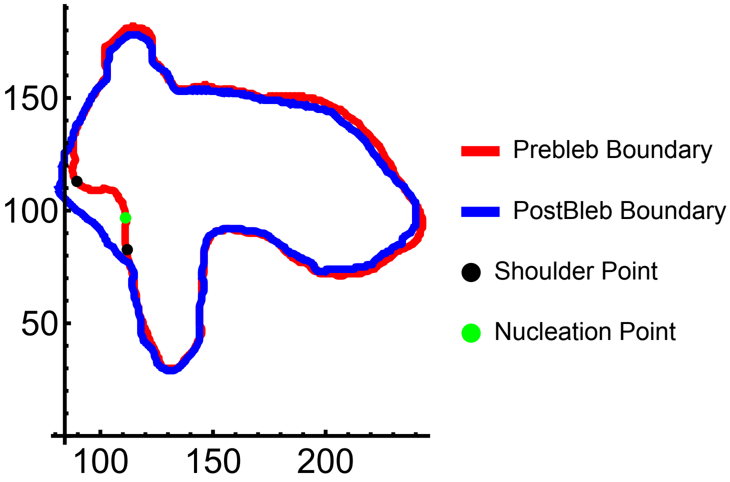

,

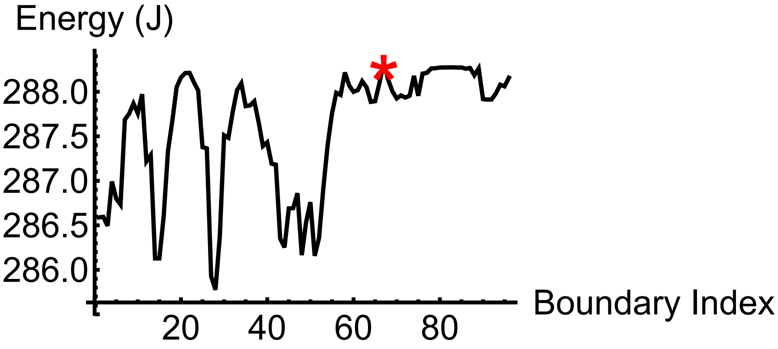

}

Bleb0117019-09

Out[•]= {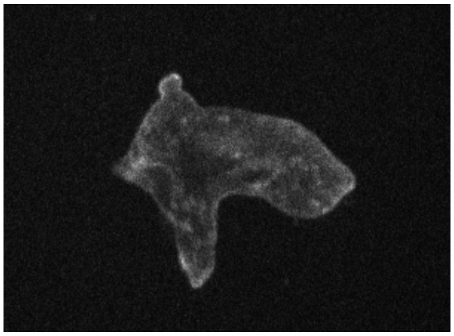, 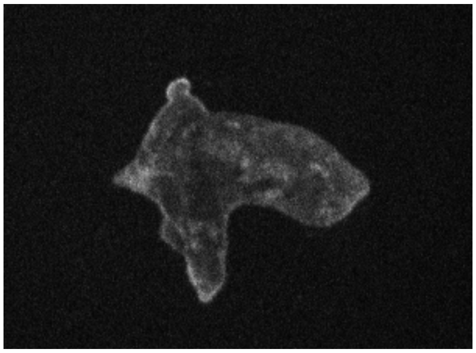,

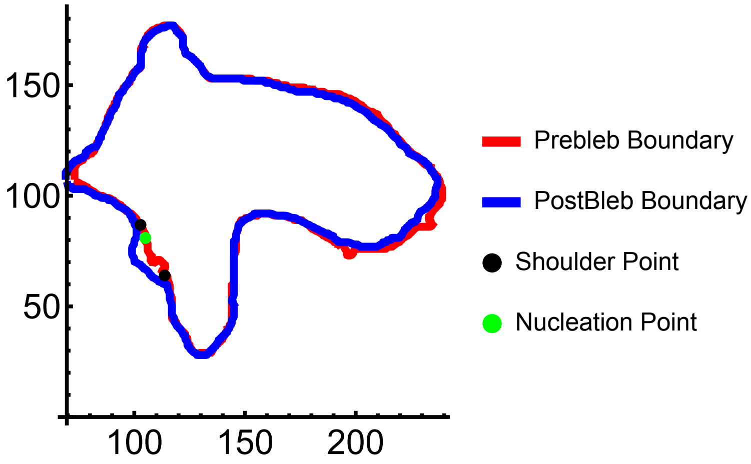

,

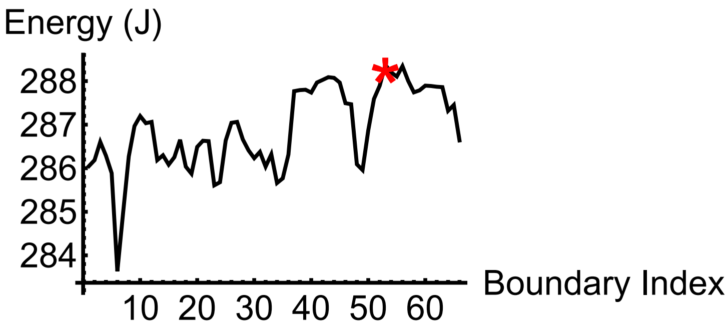

}

Bleb0117019-10

Out[•]= { 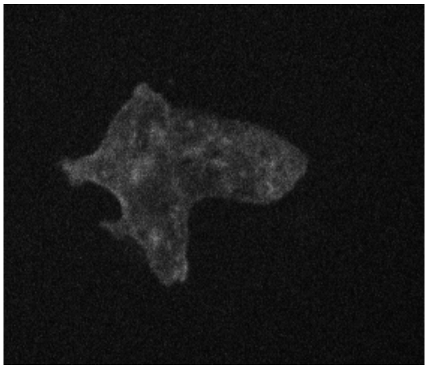 , 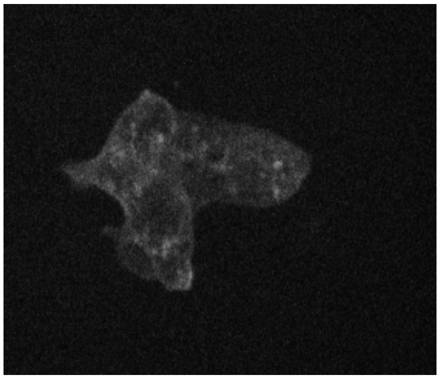 ,

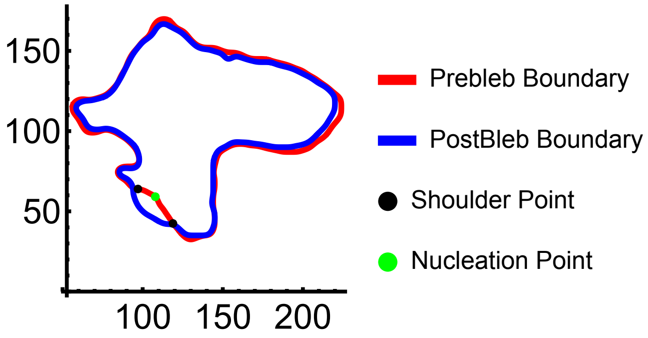

,

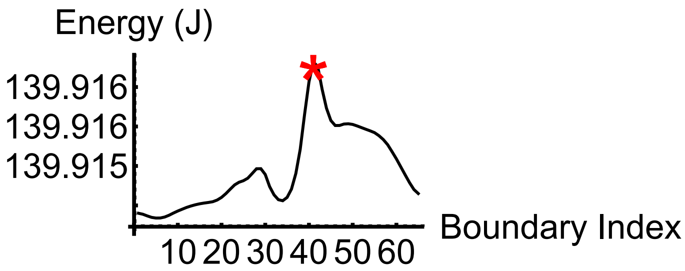

}

Bleb0117019-11

Out[ ]= {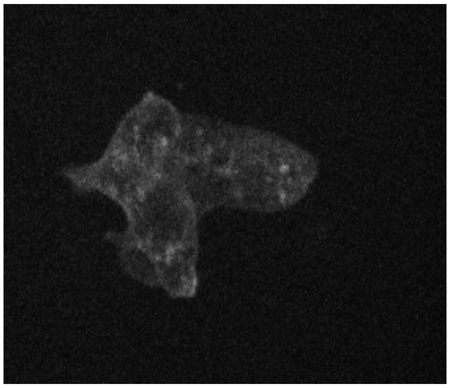, 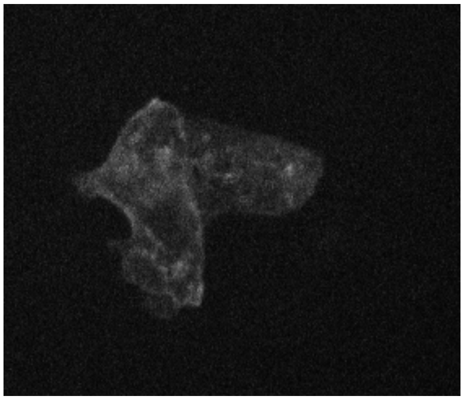,

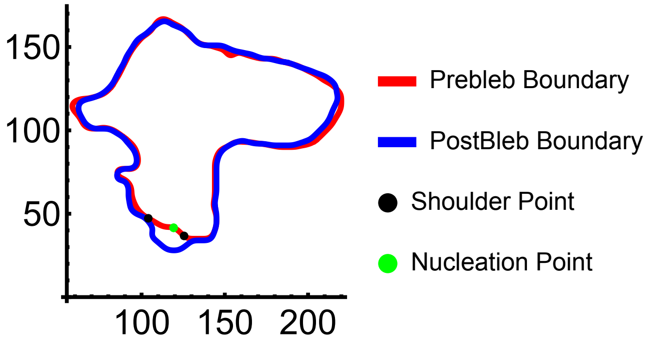

,

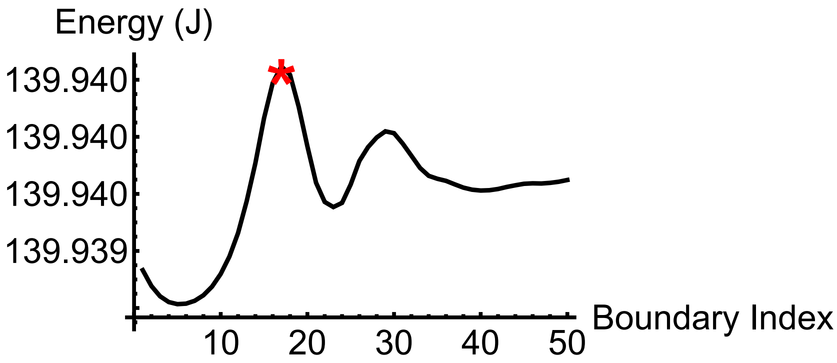

}

Bleb0117019-12

Out[ ]= {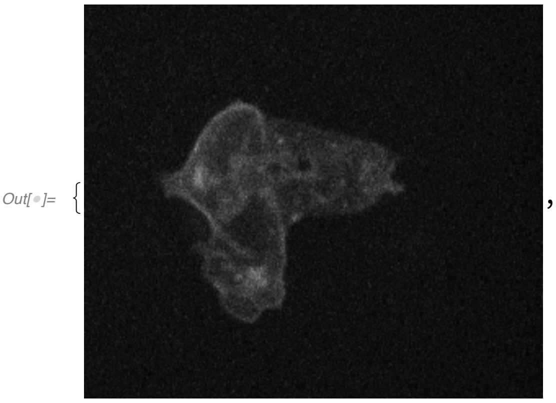, 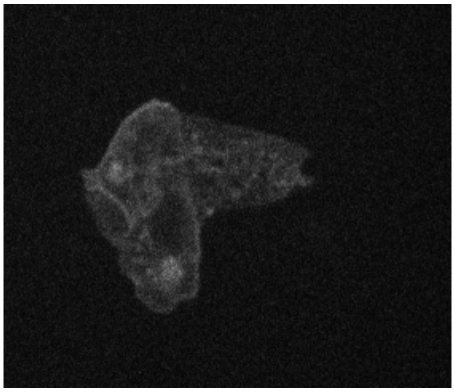,

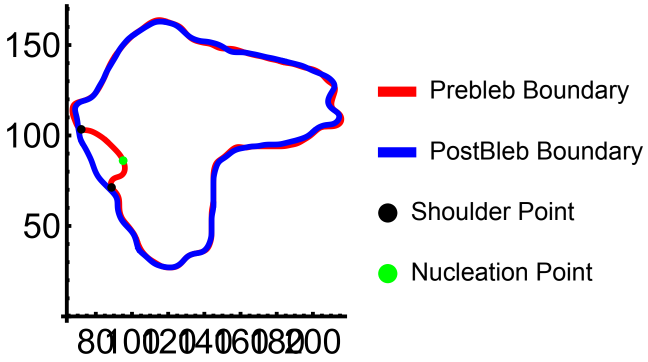

,

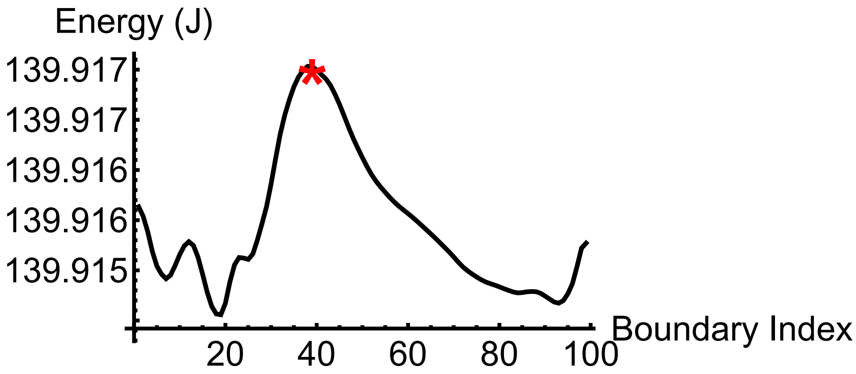

}

Bleb0117020-01

Out[ ]= {

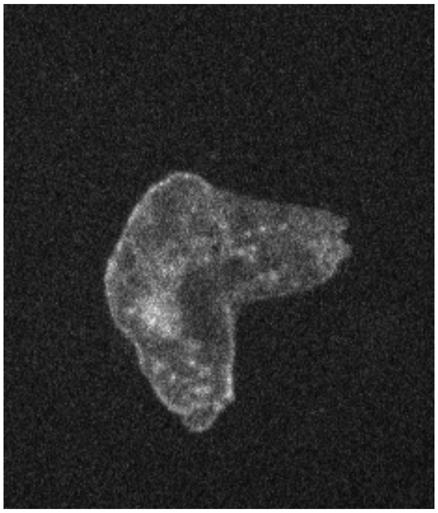

,

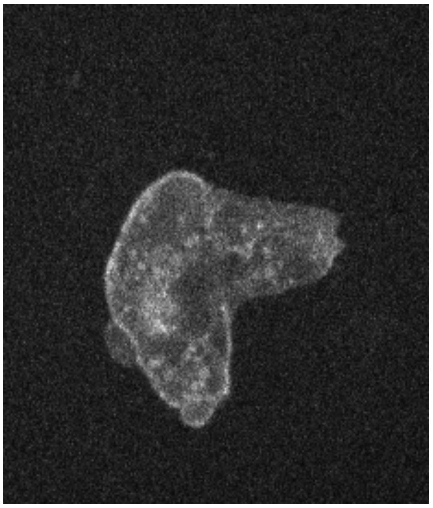

,

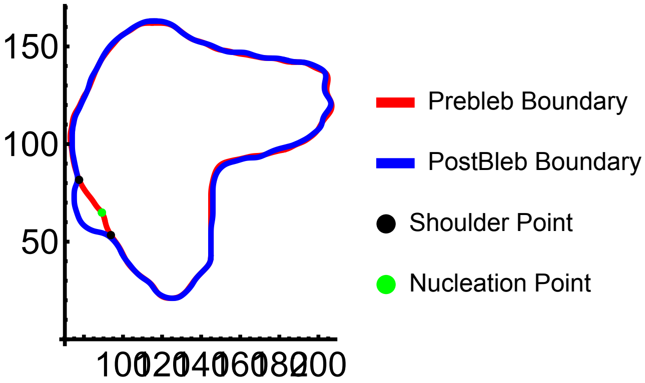

,

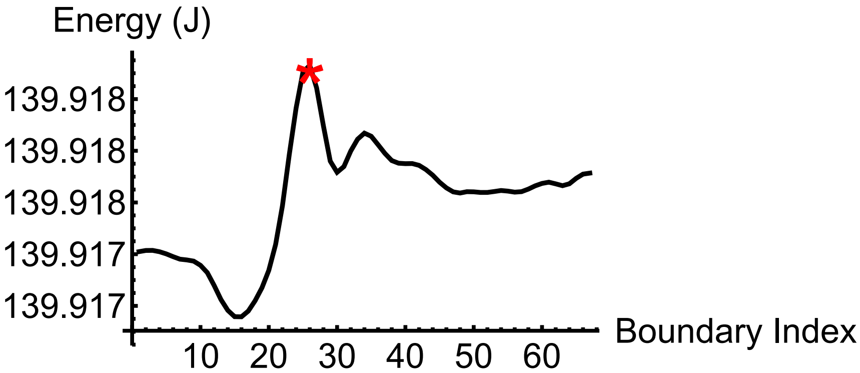

}

Bleb0117020-02

Out[ ]= {

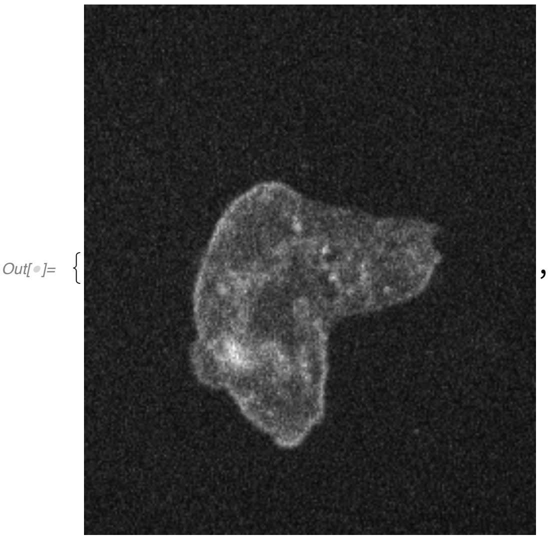

,

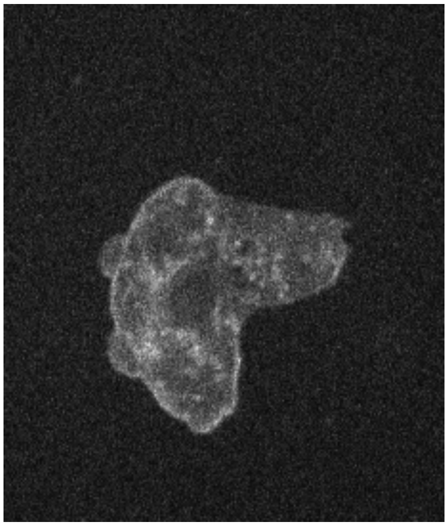

,

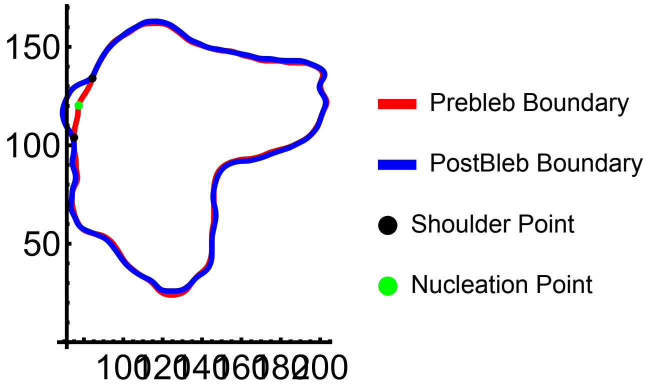

,

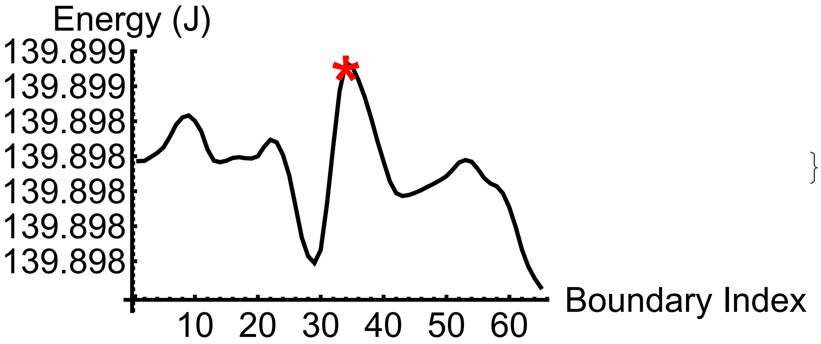

}

Bleb0117021-01

Out[•]= {

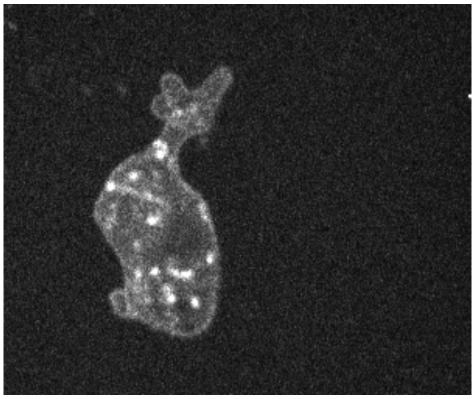

,

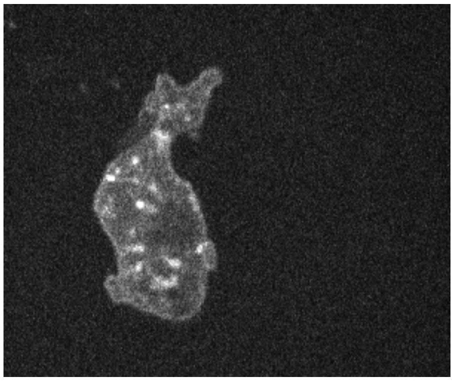

,

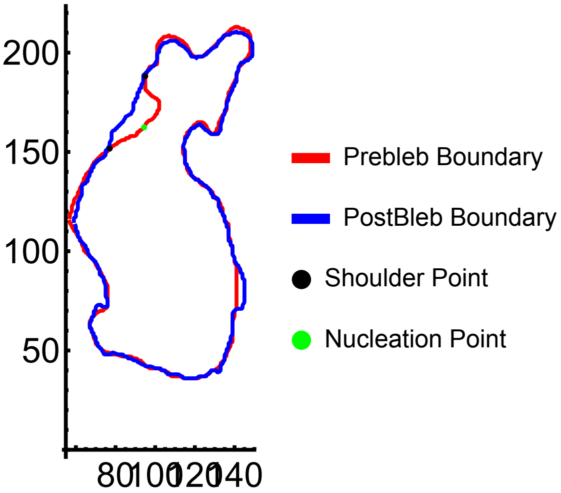

,

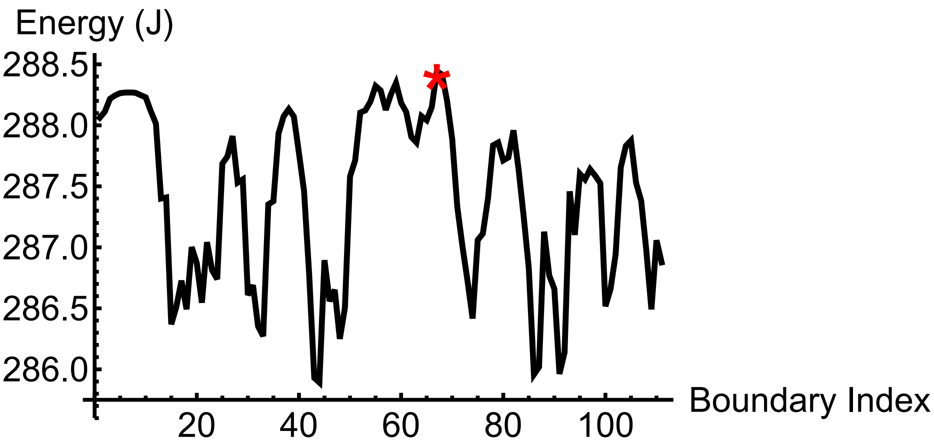

}

Bleb0117021-02

Out[ ]= {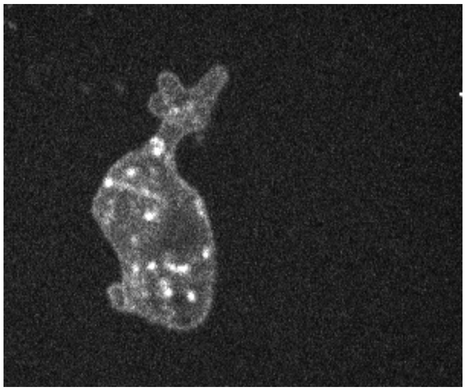, 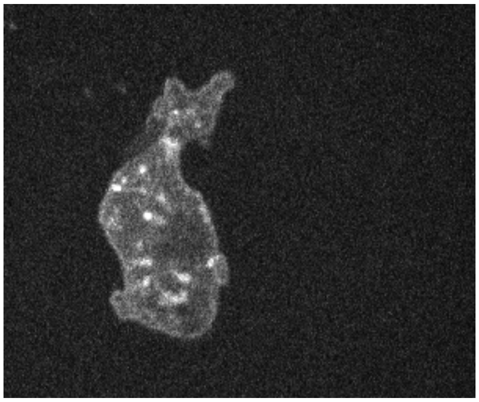,

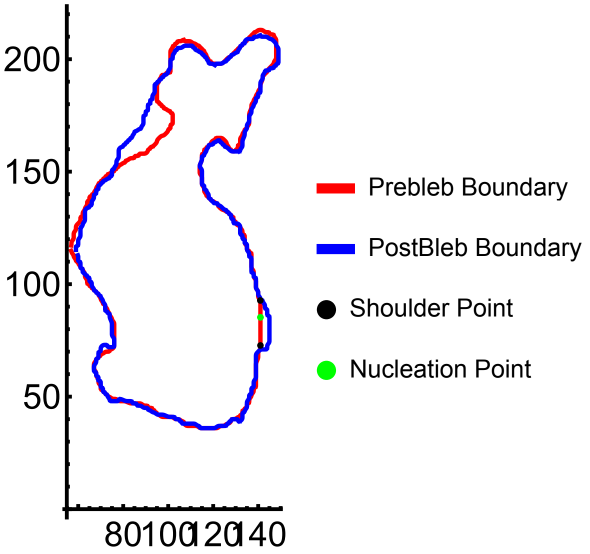

,

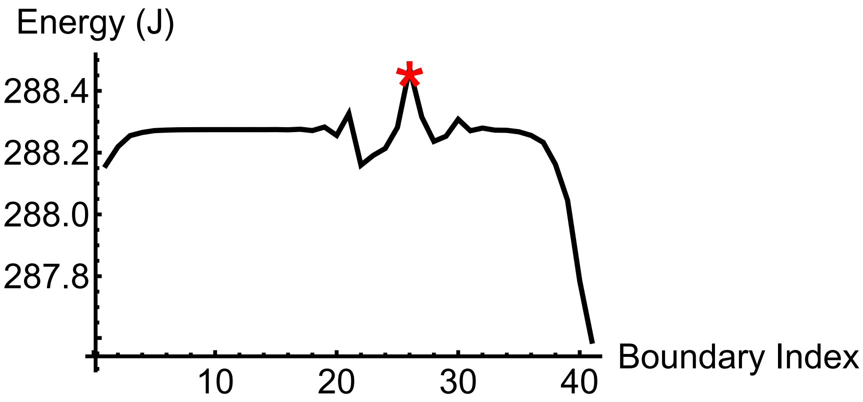

}

Bleb0117021-03

Out[ ]= {

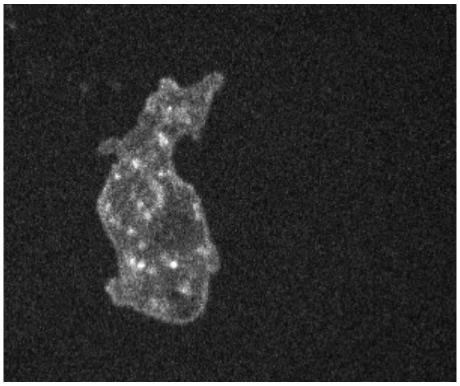

,

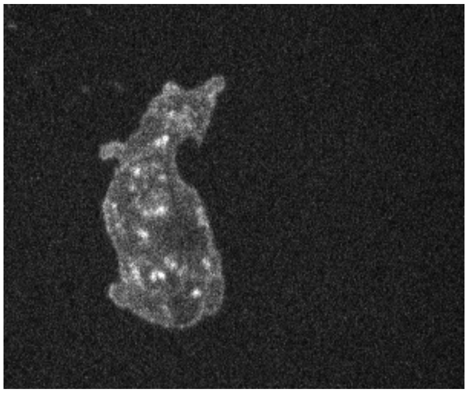

,

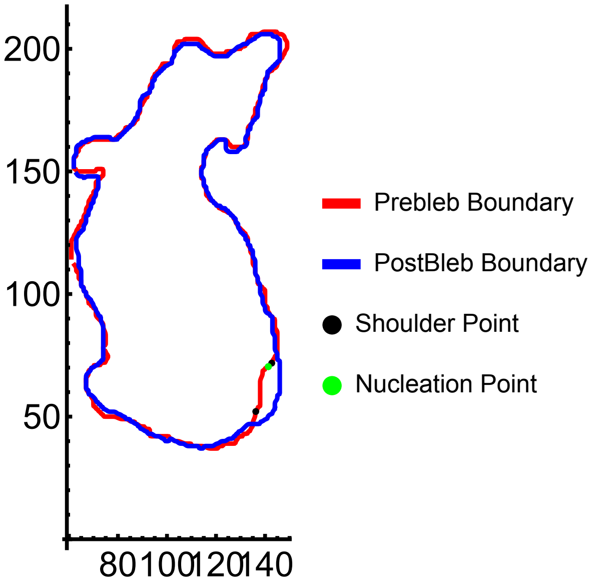

,

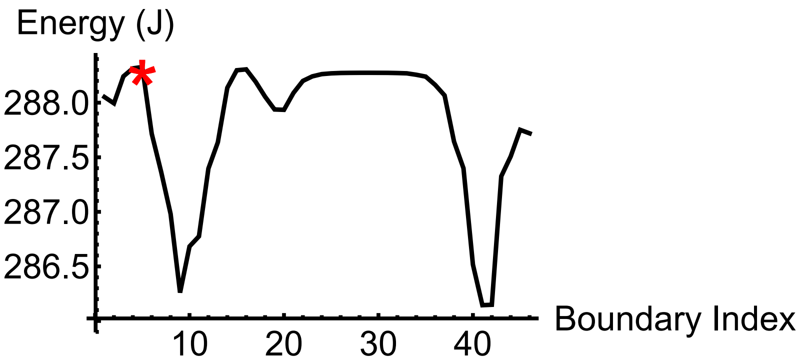

}

Bleb0117021-04

Out[•]= {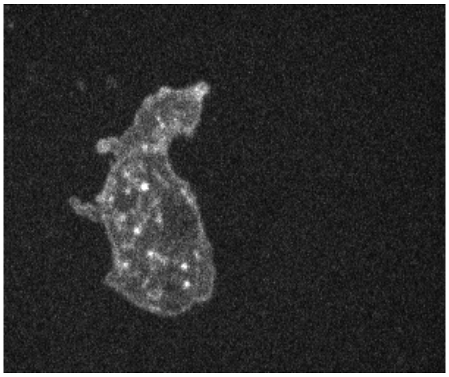, 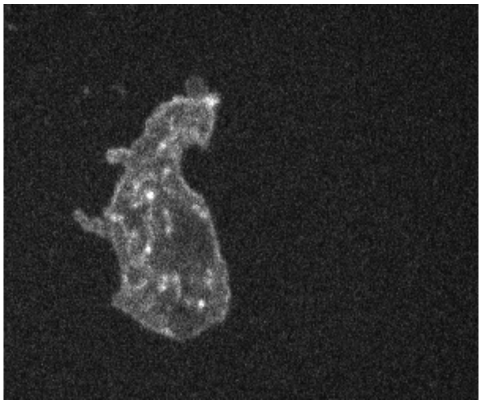,

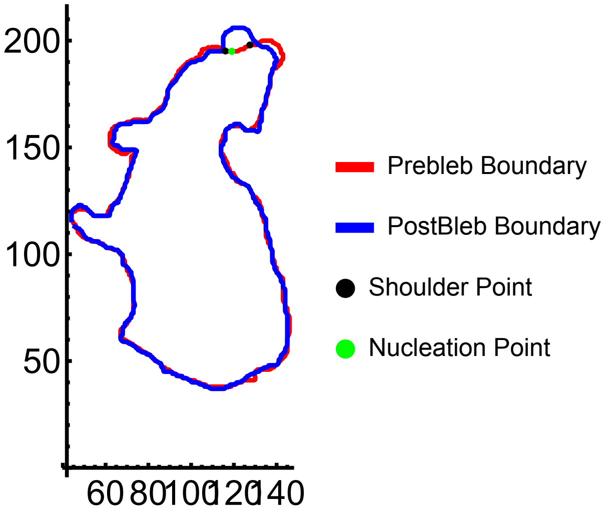

,

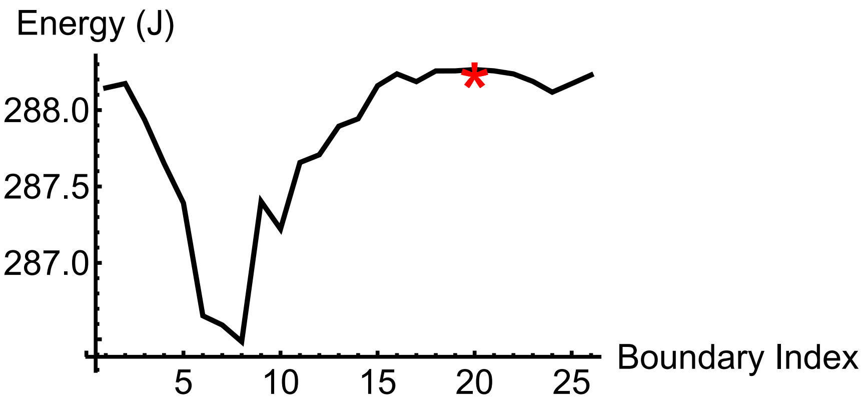

}

Bleb0117021-05

Out[]= {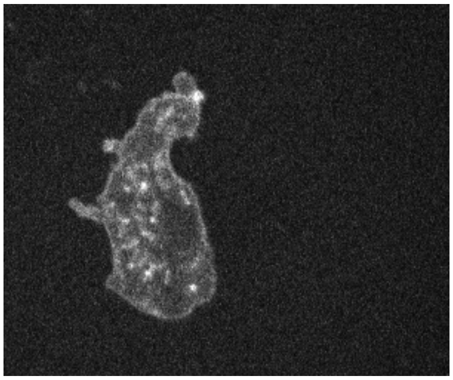, 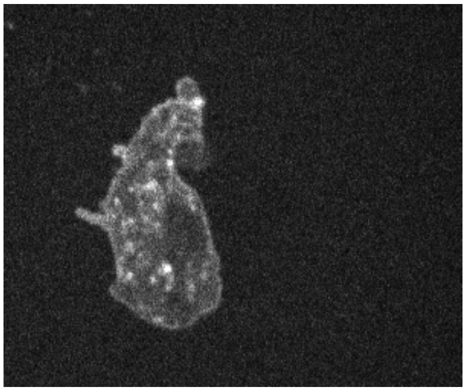,

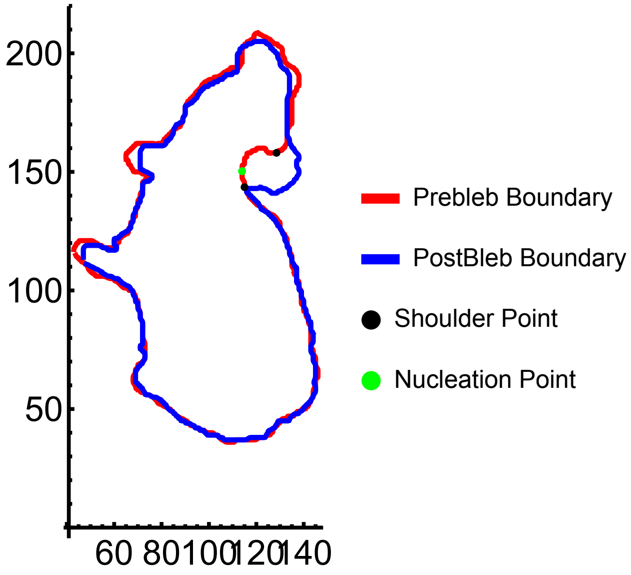

,

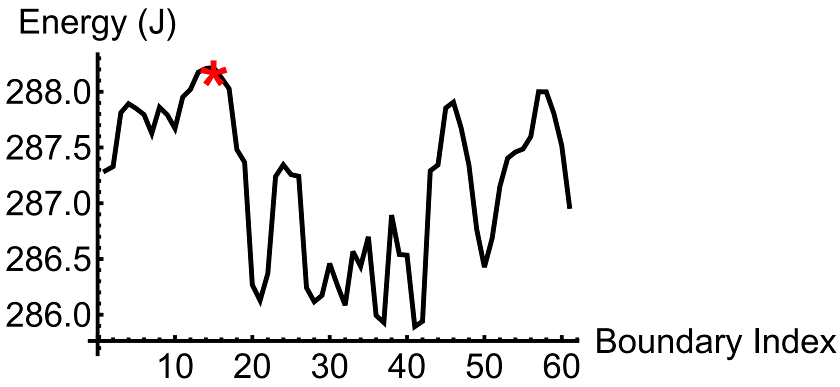

}

Bleb0117021-06

Out[ ]= {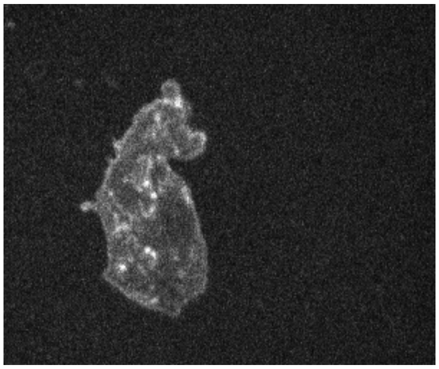, 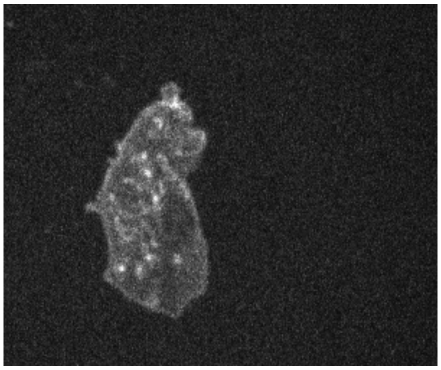,

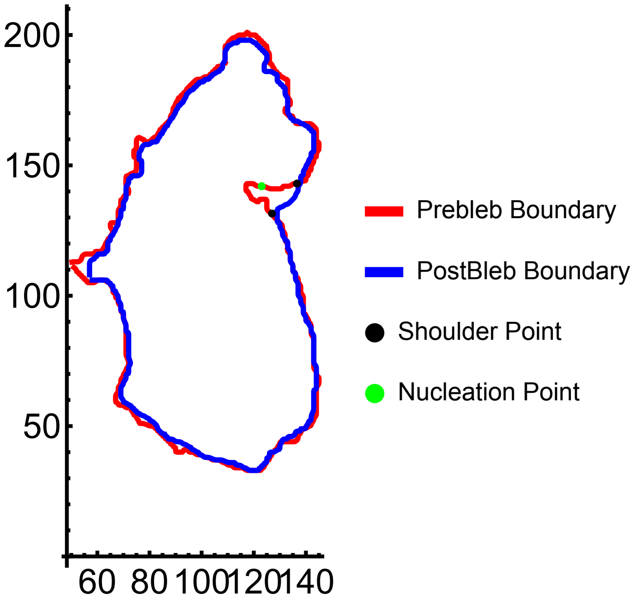

,

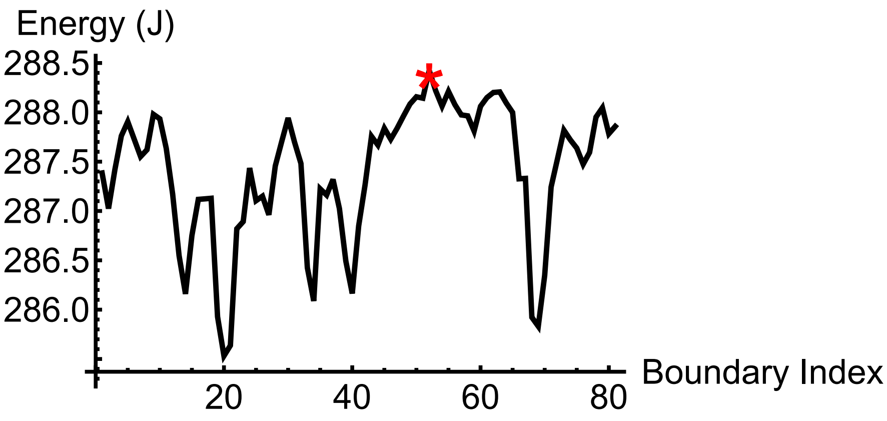

}

Bleb Nucleation Predictions for Experiment 0423

0423003-01

Out[ ]= {

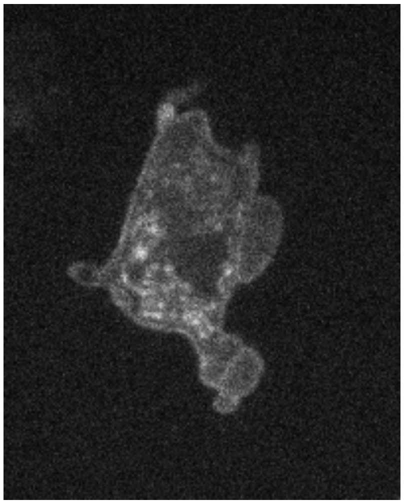

,

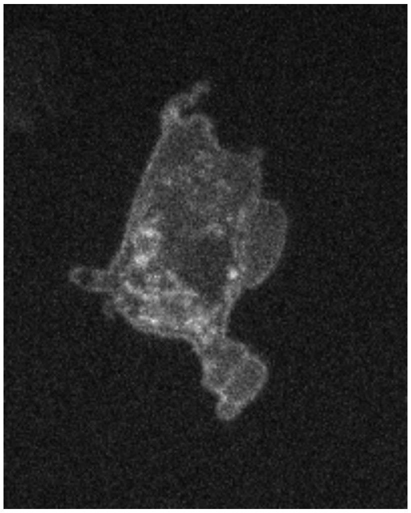

,

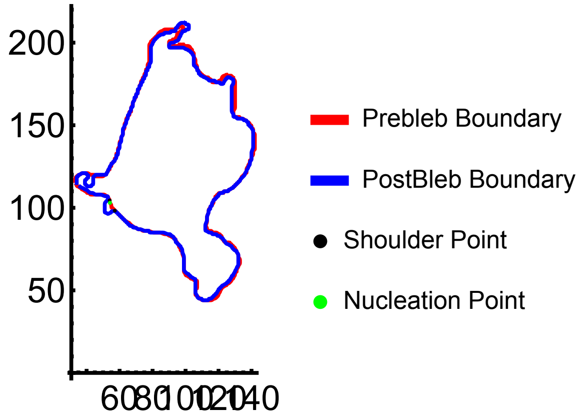

,

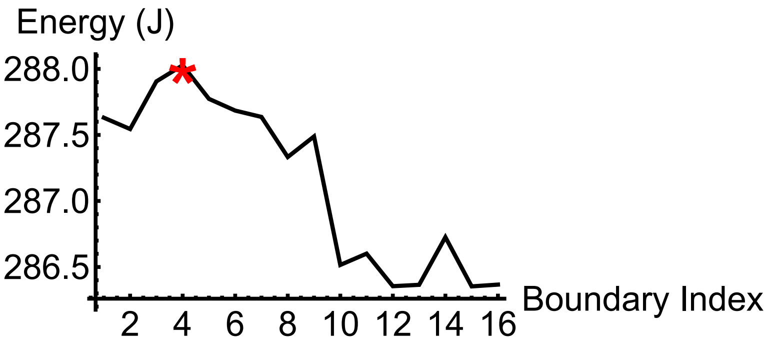

}

0423003-02

Out[]= {

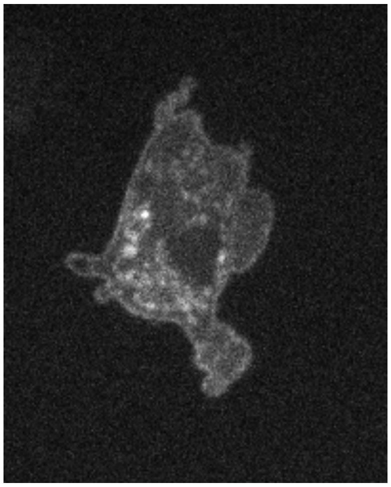

,

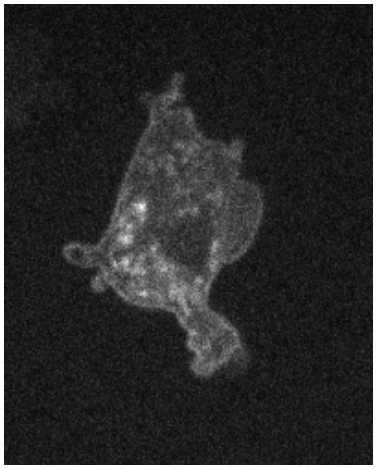

,

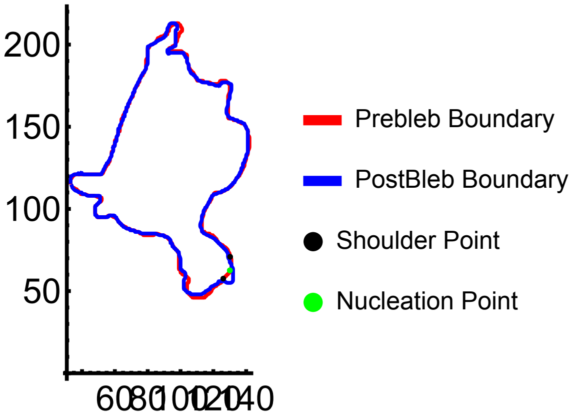

,

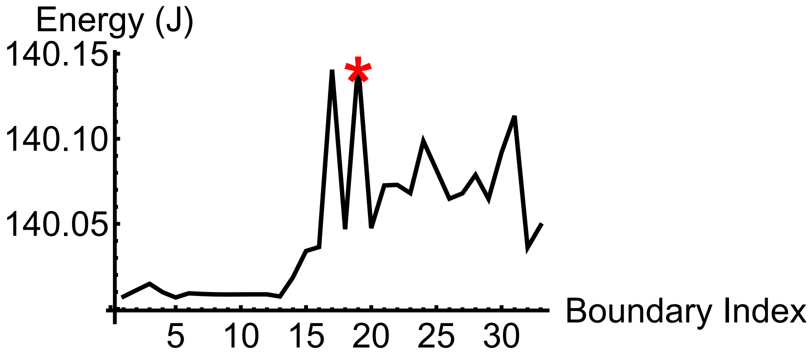

}

0423003-03

Out[ ]= {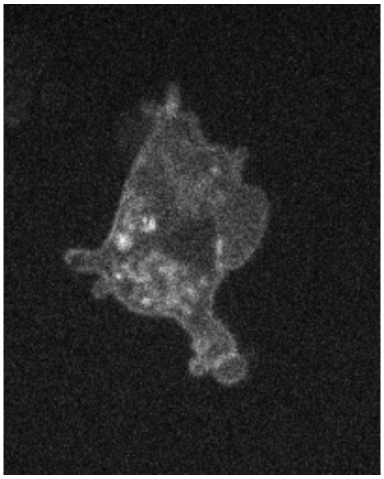, 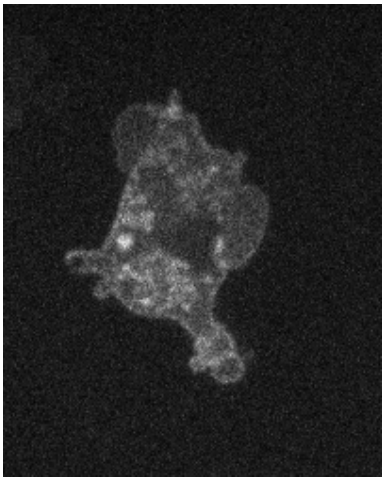,

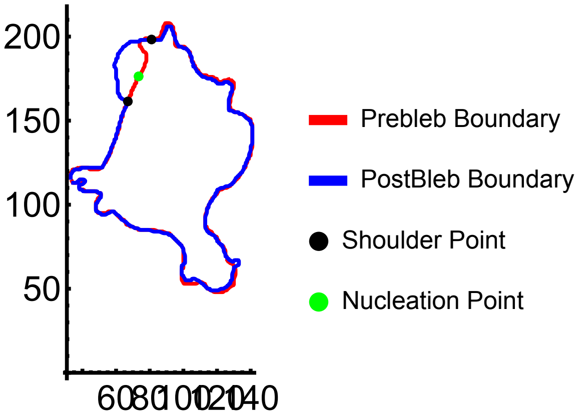

,

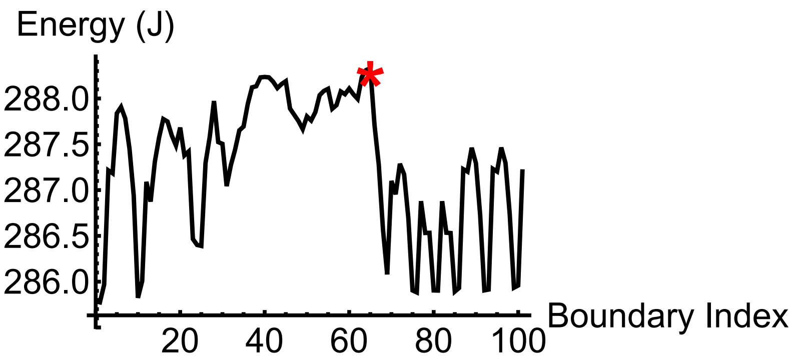

}

0423003-04

Out[ ]= {

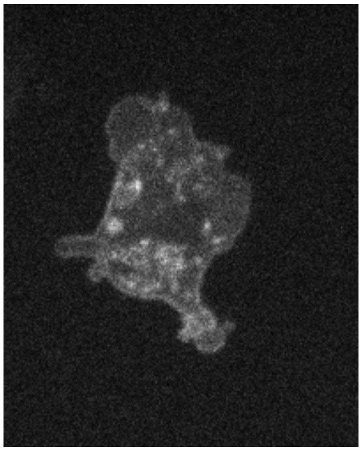

,

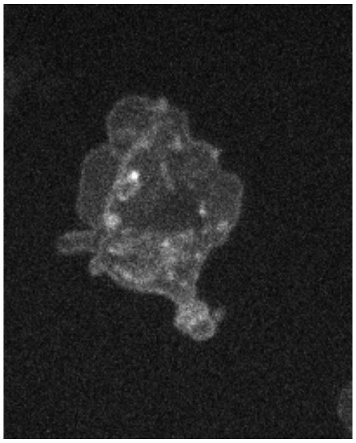

,

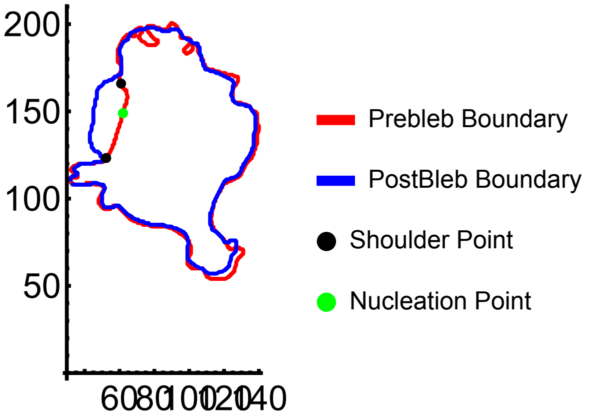

,

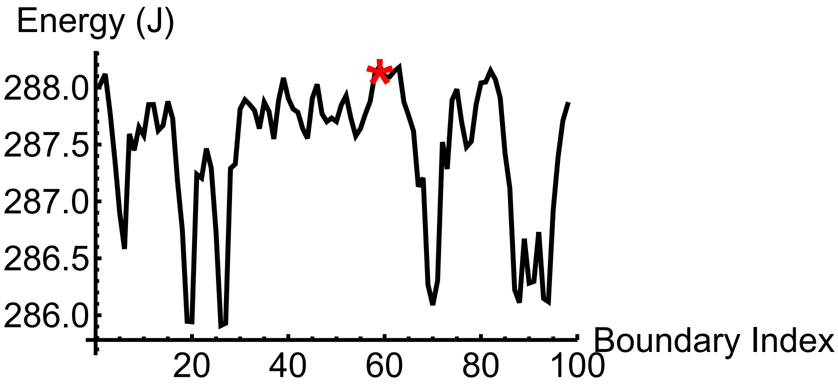

}

0423005-01

Out[]= {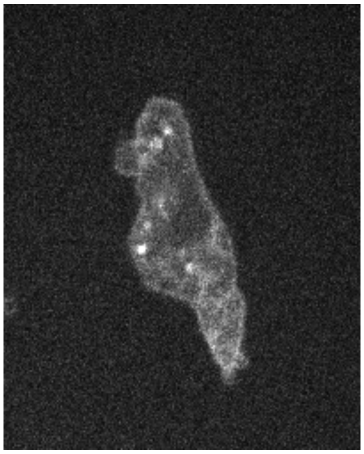, 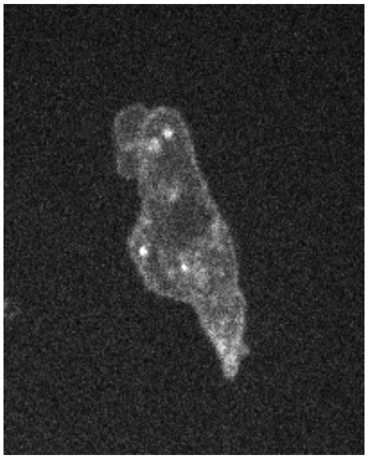, 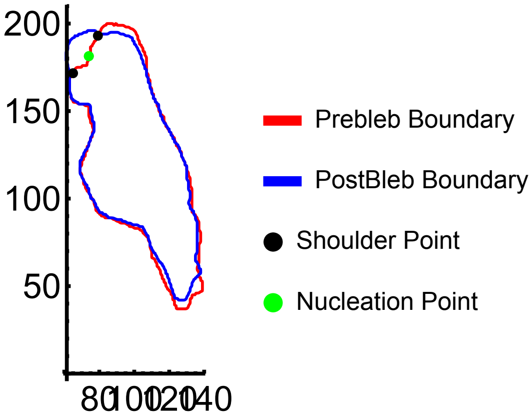

, 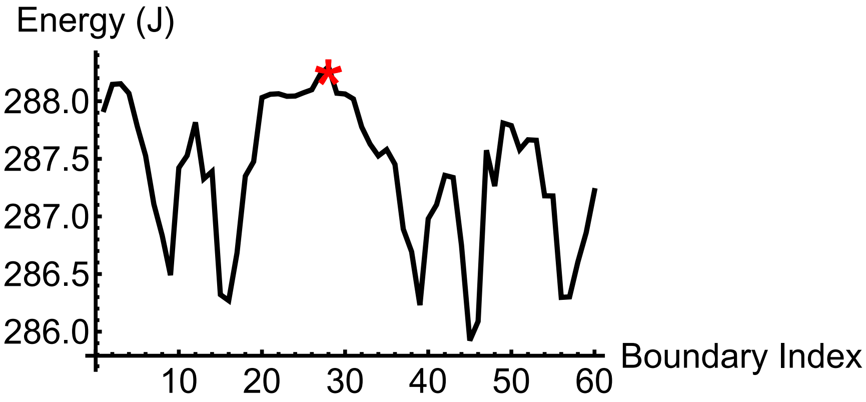

}

0423005-02

Out[ ]= {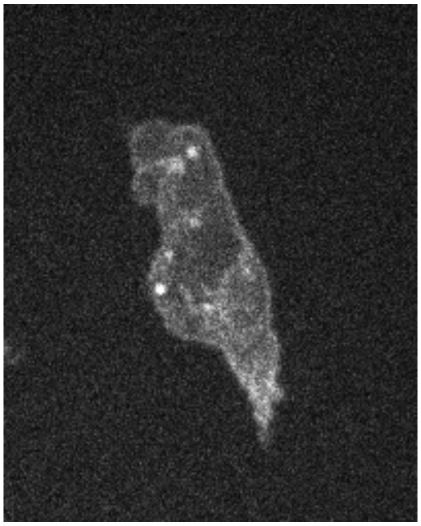,

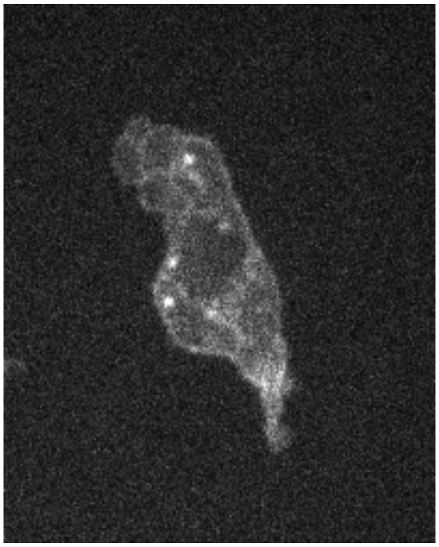, 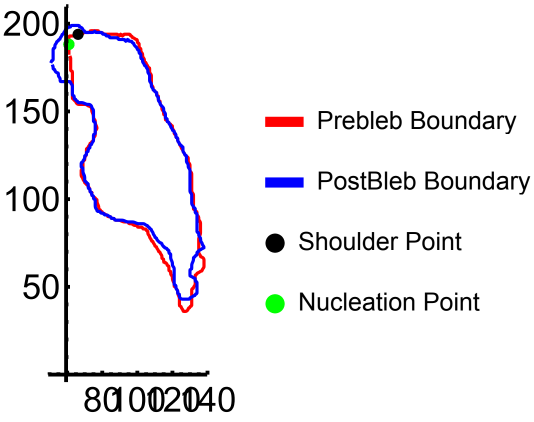

,

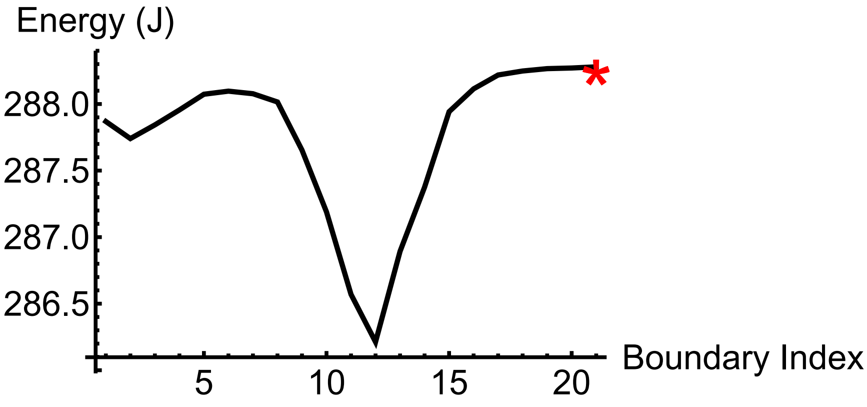

}

0423005-05

Out[ ]= {

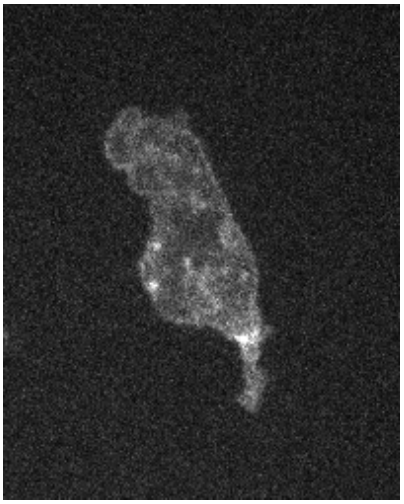

,

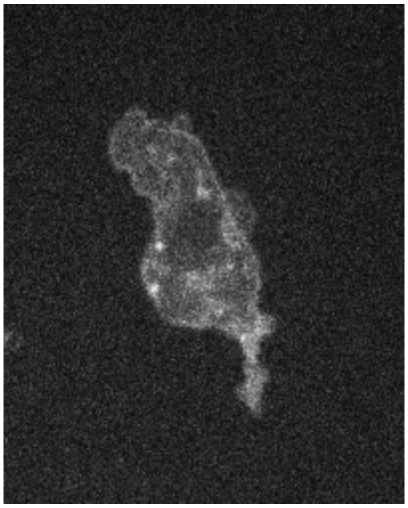

,

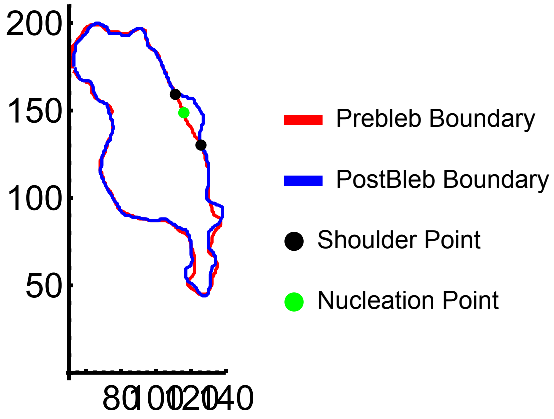

,

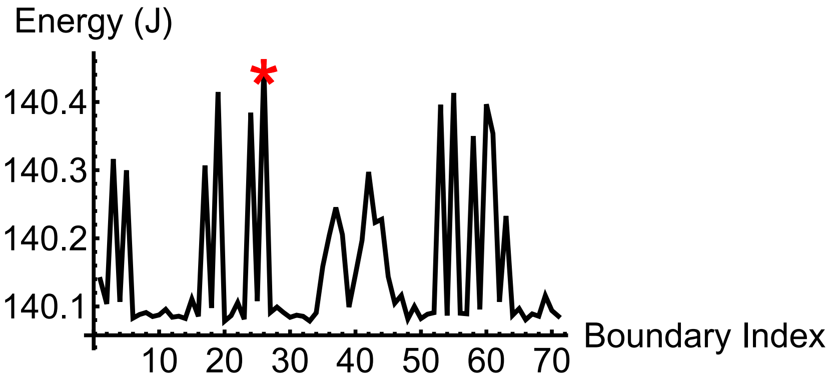

}

0423005-06

Out[ ]= {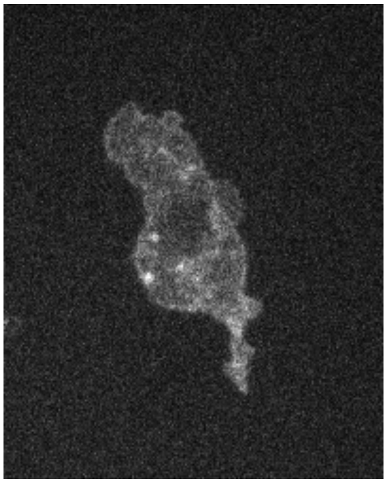, 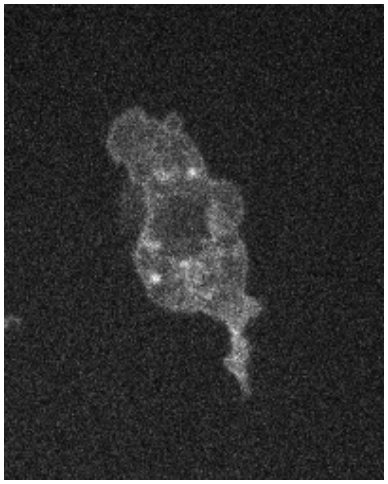,

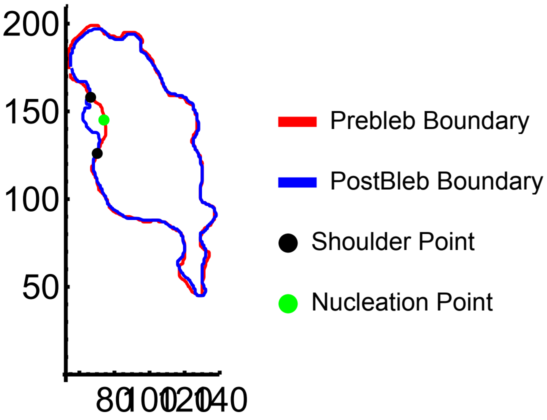

,

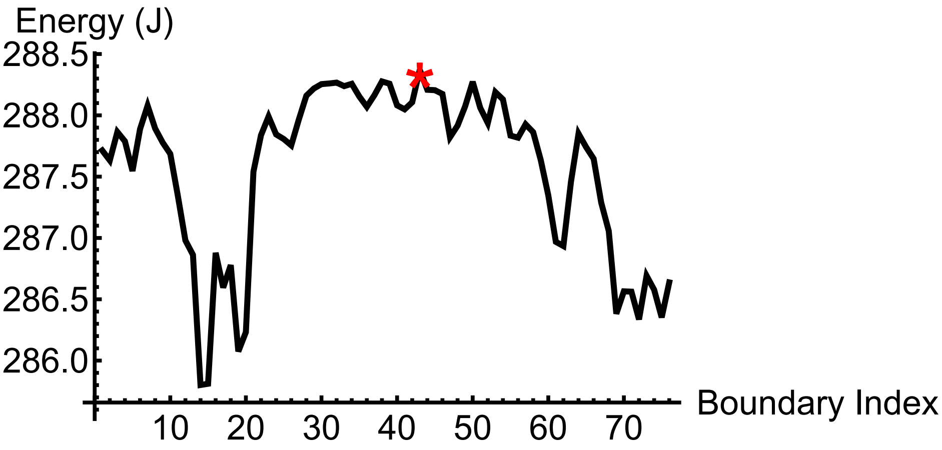

}

0423005-08

Out[ ]= { 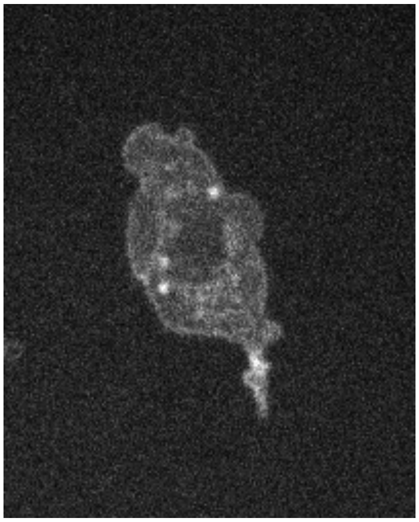,

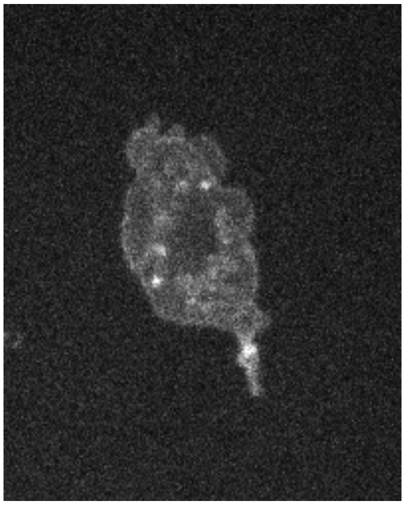, 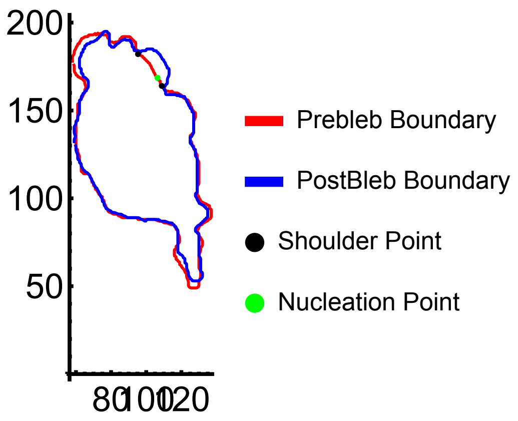

,

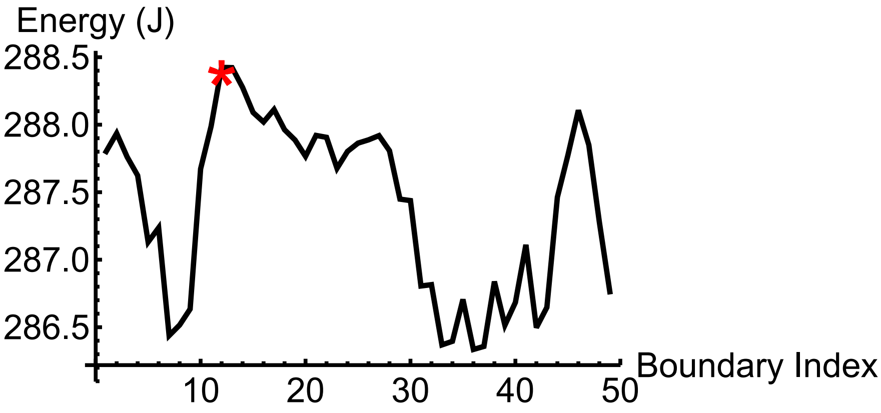

}

0423005-09

Out[•]= {

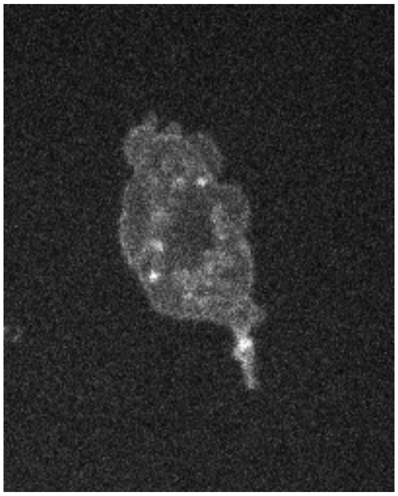

,

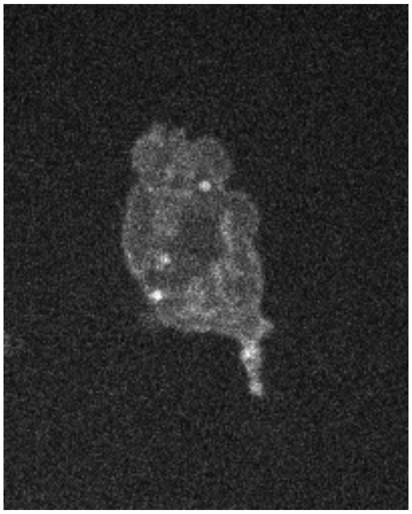

,

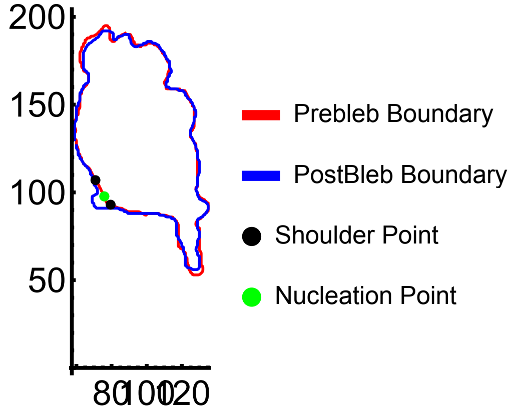

,

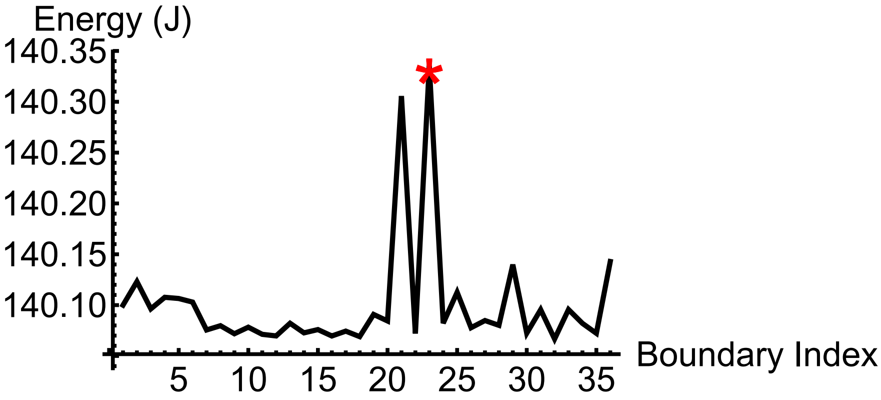

}

0423005-10

Out[ ]= {

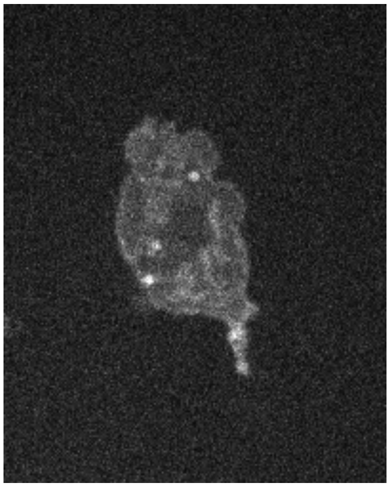

,

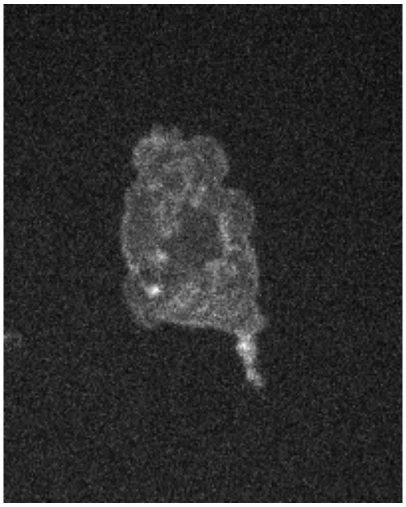

,

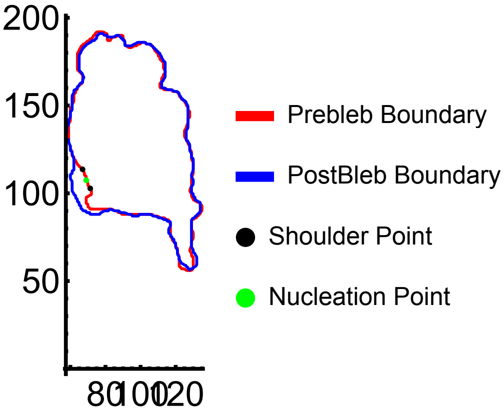

,

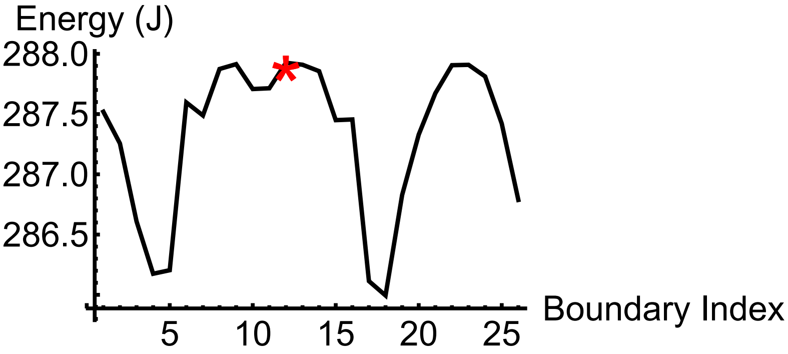

}

0423006-01

Out[•]= {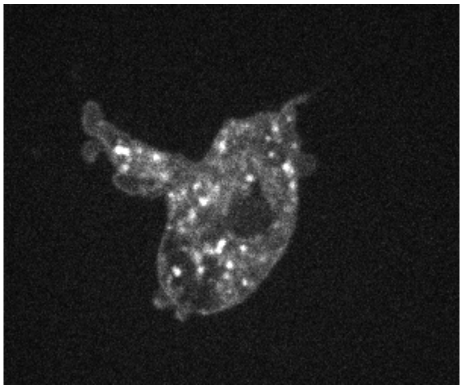, 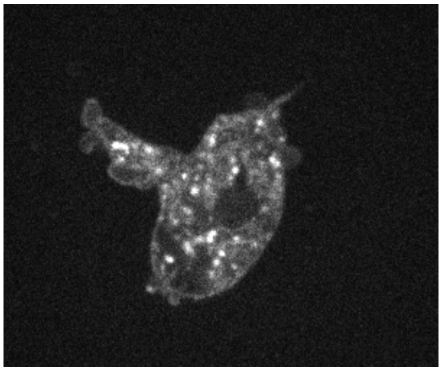,

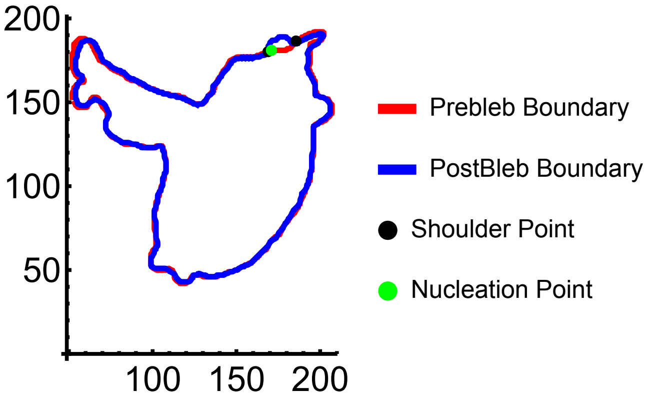

,

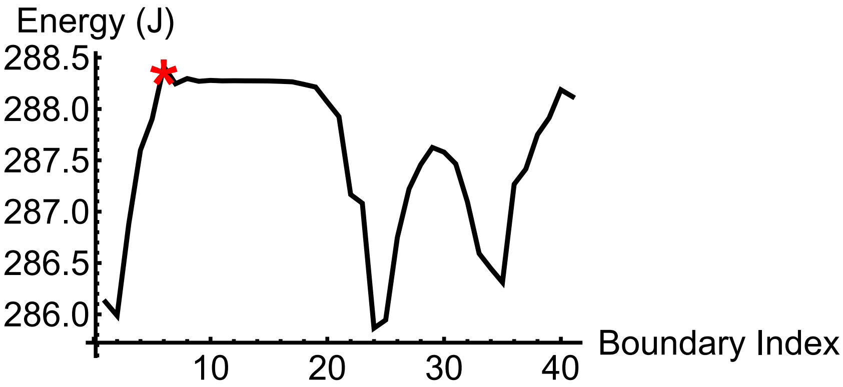

}

0423007-01

Out[•]= {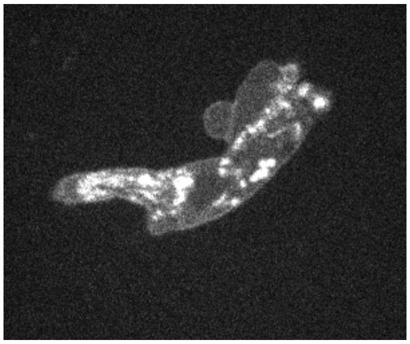, 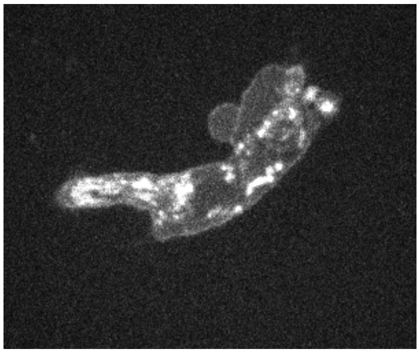,

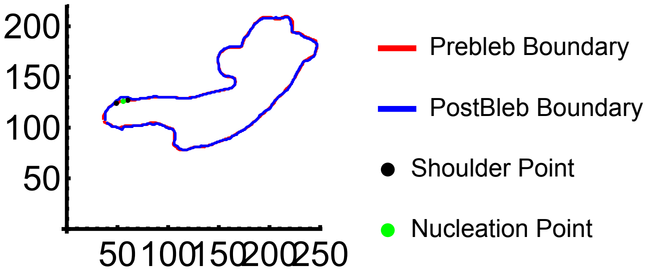

,

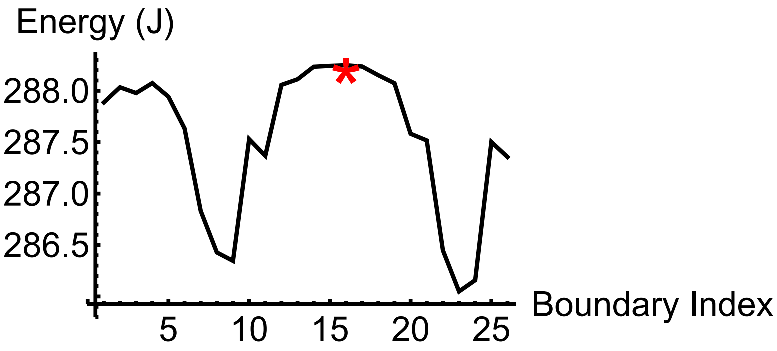

}

0423007-02

Out[ ]= {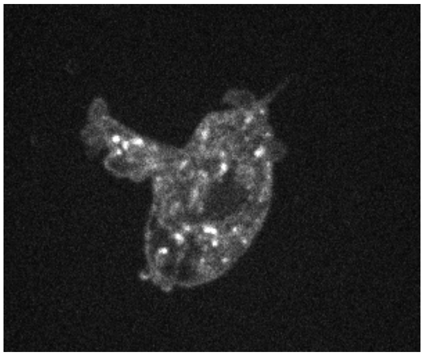, 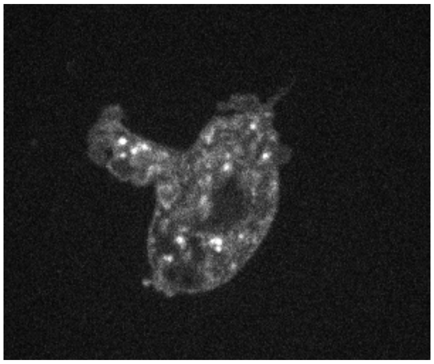,

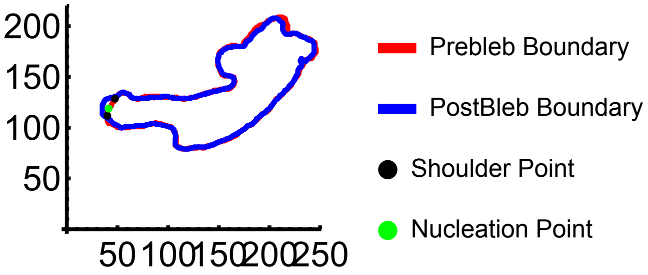

,

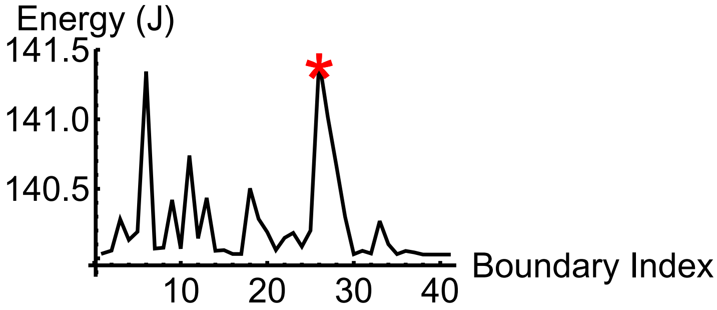

}

0423007-03

Out[ ]= {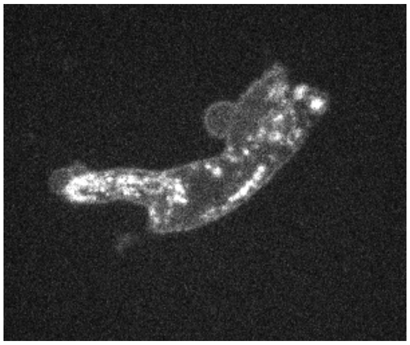, 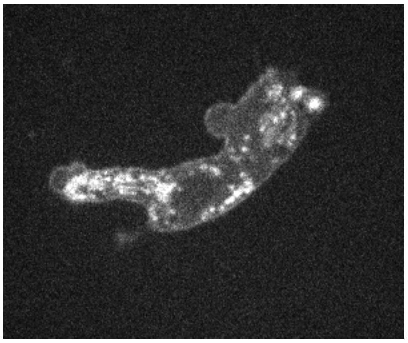,

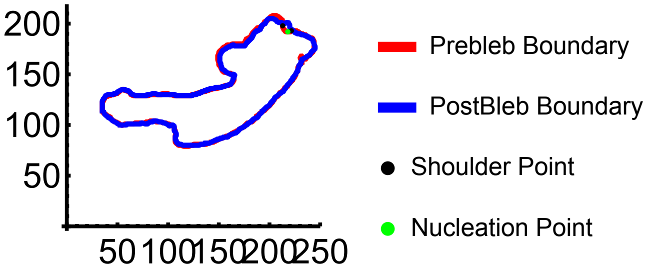

,

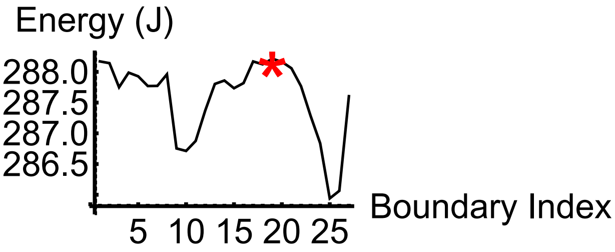

}

0423007-04

Out[ ]= {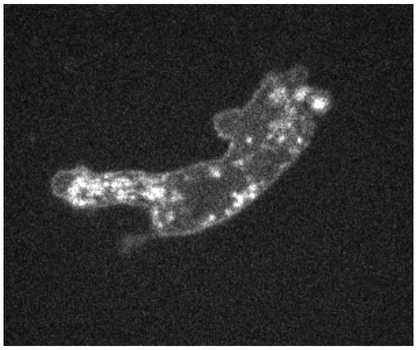, 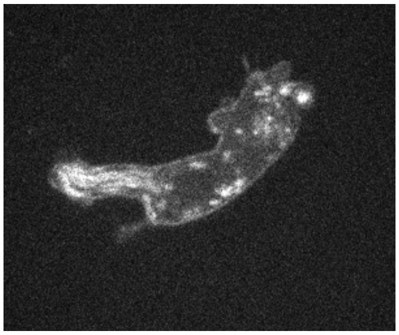,

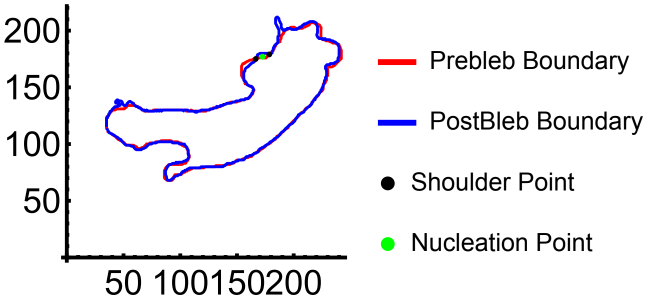

,

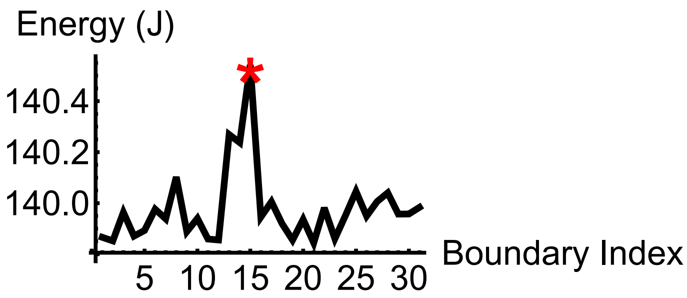

}

0423007-05

Out[ ]= {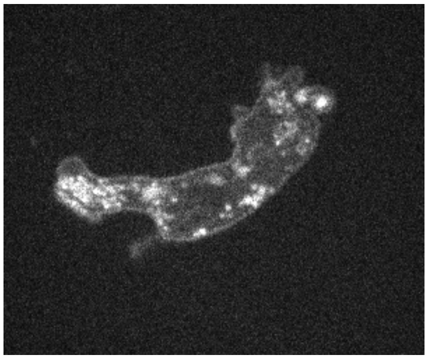, 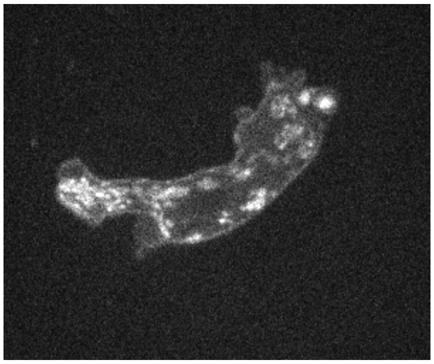,

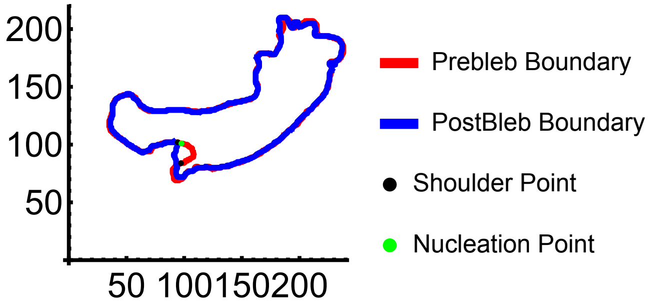

,

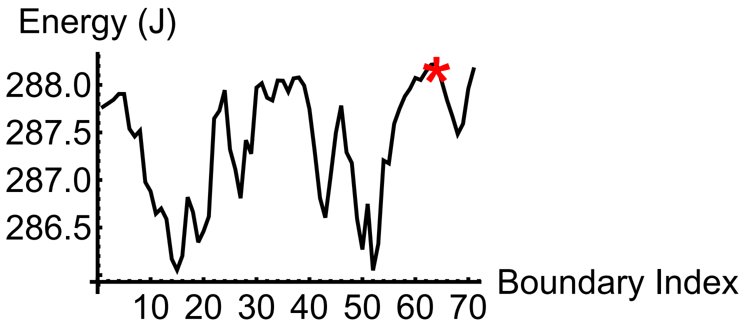

}

0423007-06

Out[ ]= {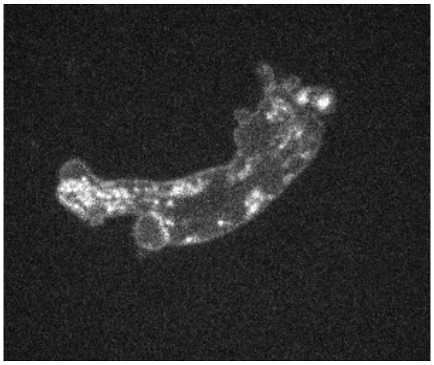, 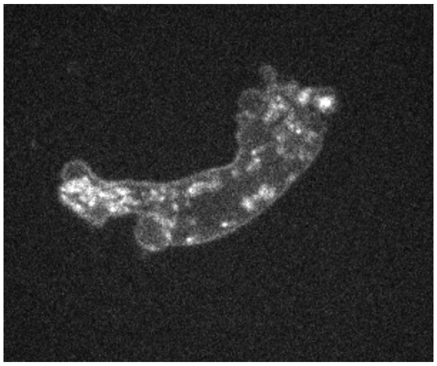,

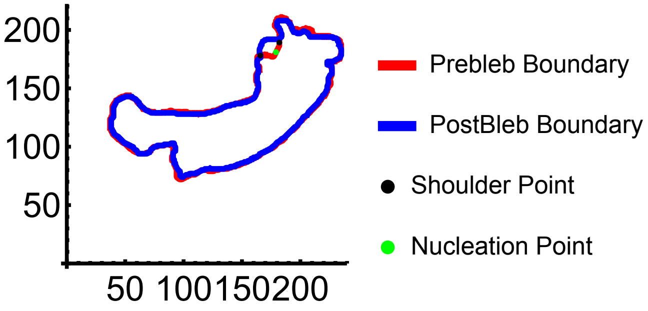

,

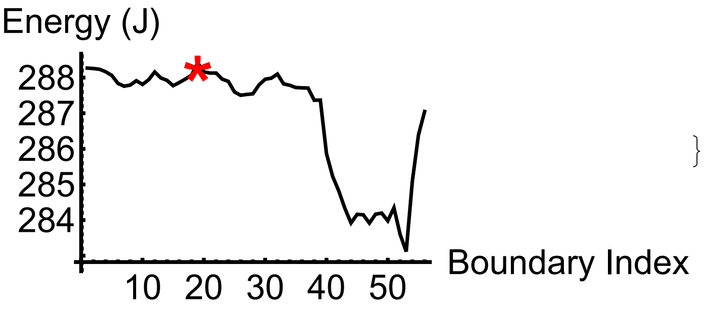

}

0423008-01

Out[ ]= {

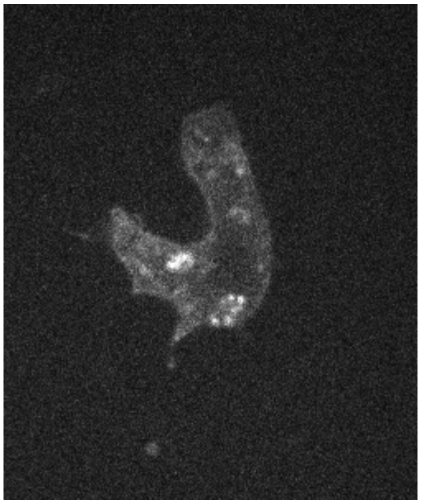

,

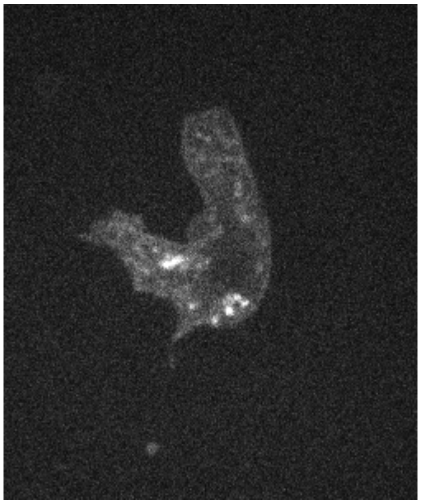

,

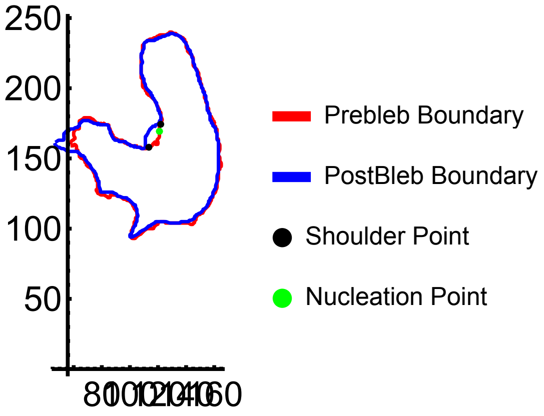

,

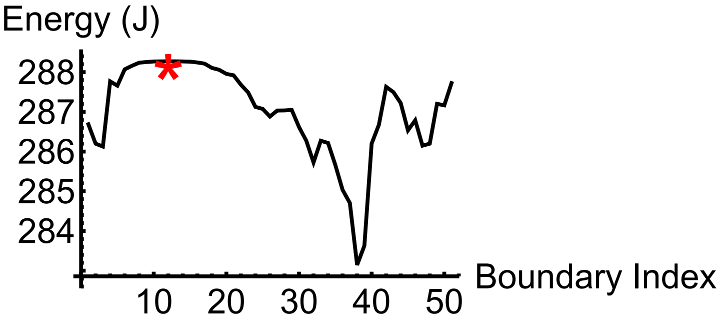

}

0423008-02

Out[•]= {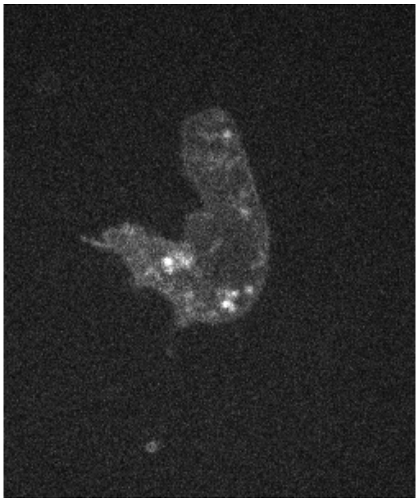,

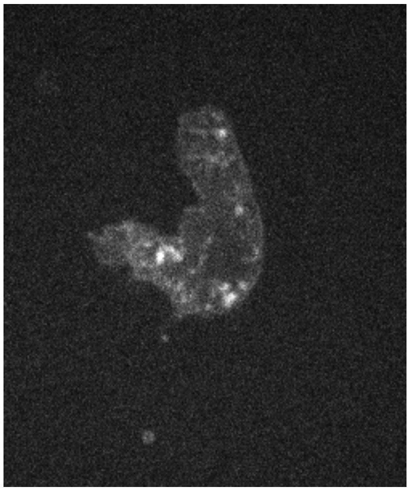, 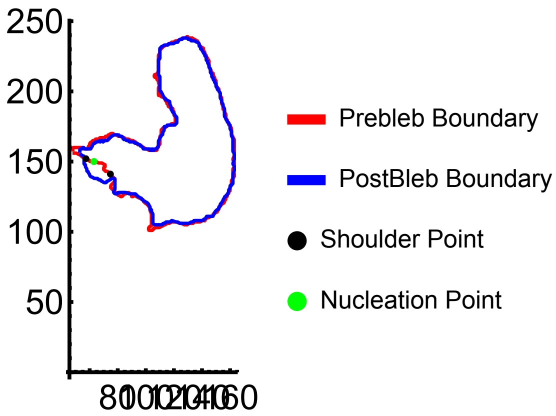

,

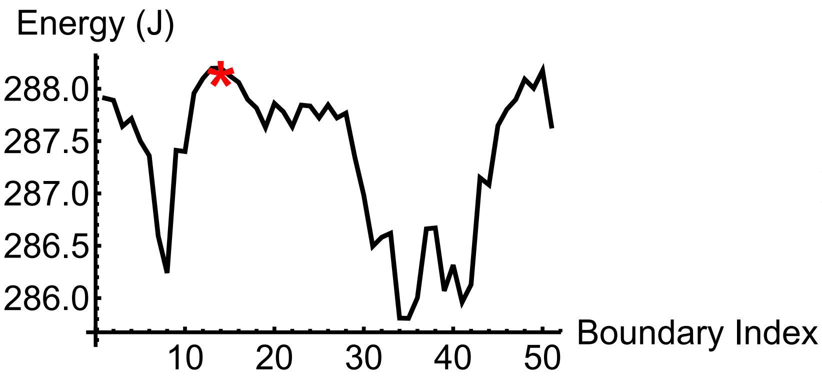

}

0423008-03

Out[•]= {

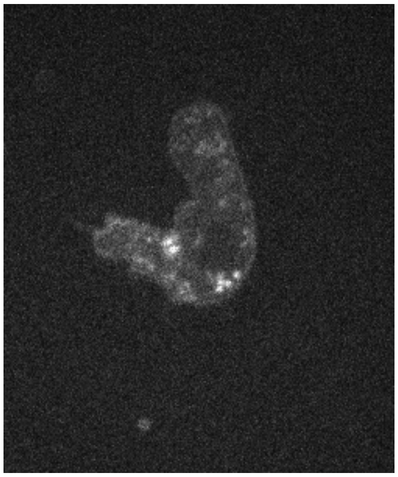

,

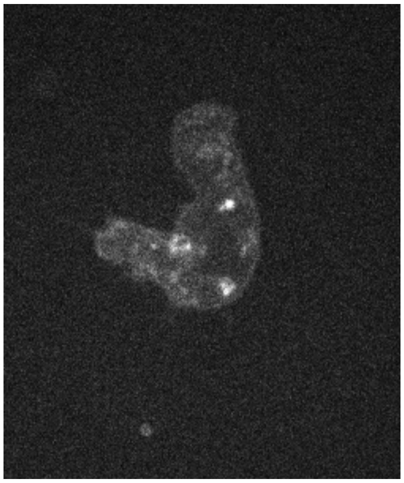

,

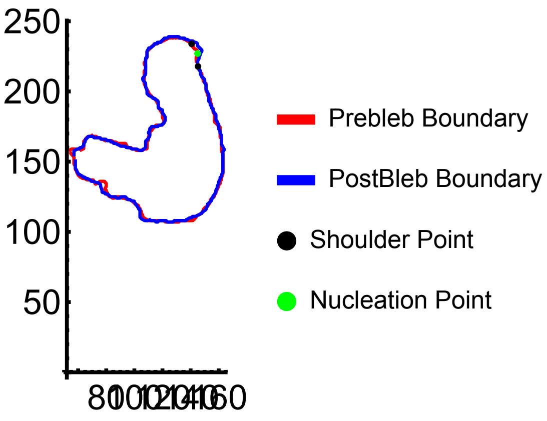

,

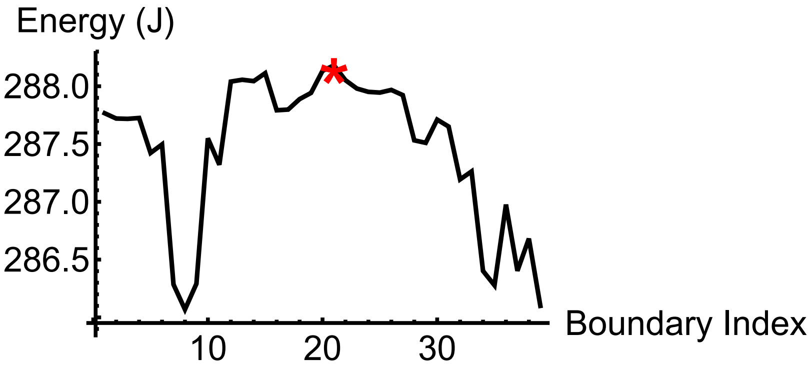

}

0423016-01

{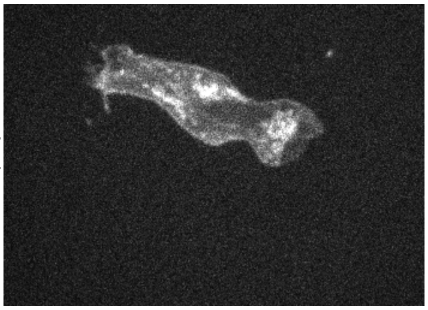, 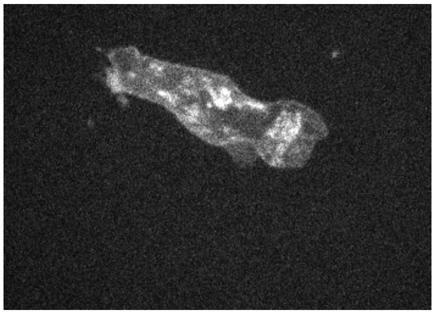,

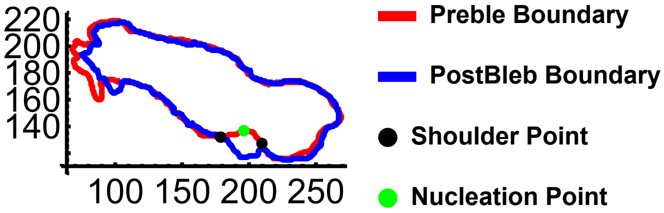
220
200
180
160
140
100 150 200 250
Preble Boundary
PostBleb Boundary
Shoulder Point
Nucleation Point
,

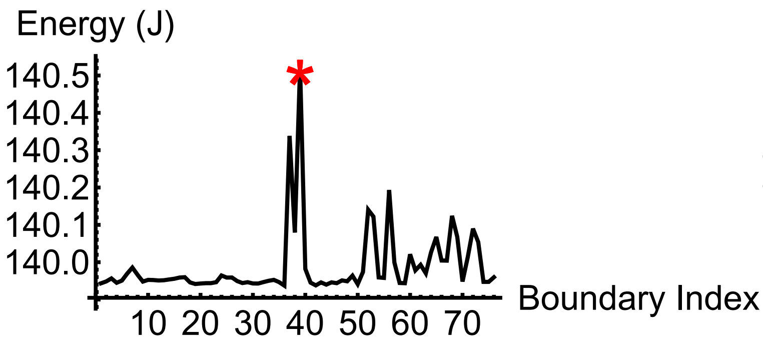
Energy (J)
140.5
140.4
140.3
140.2
140.1
140.0
10 20 30 40 50 60 70
Boundary Index
}

0423016-02

Out[•]= {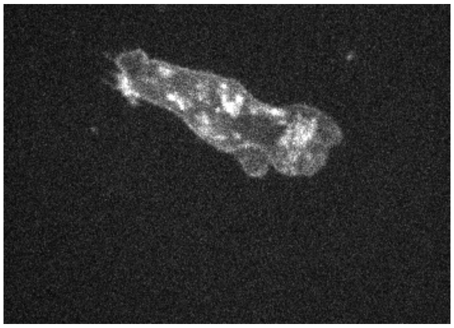, 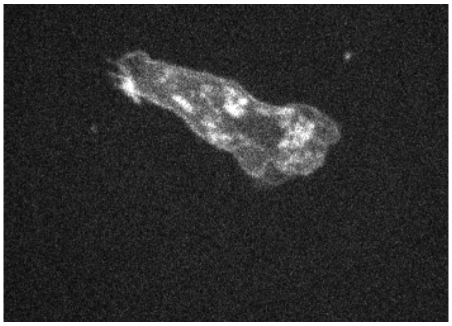,

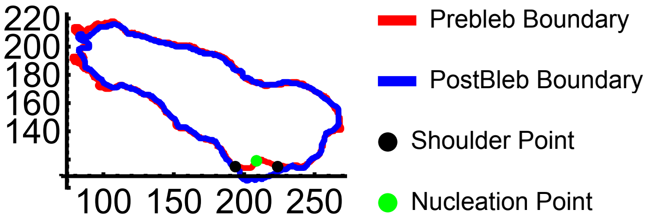

,

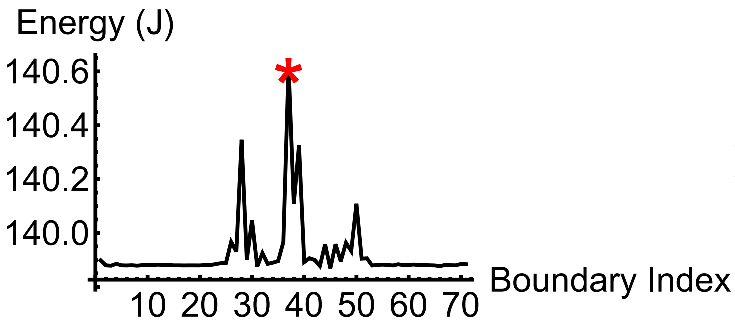

}

0423016-03

Out[ ]= {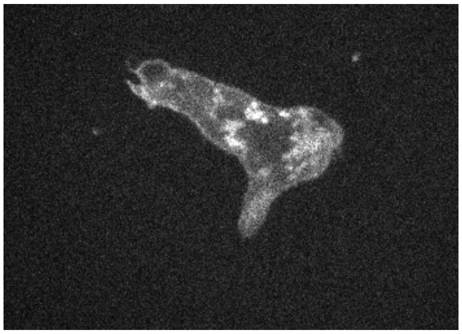, 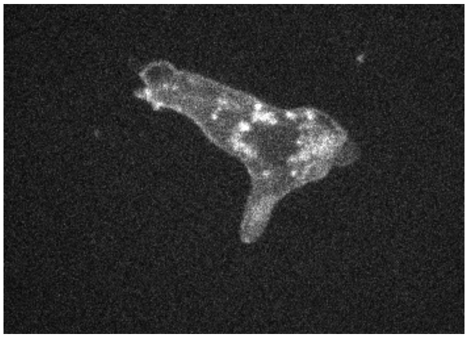,

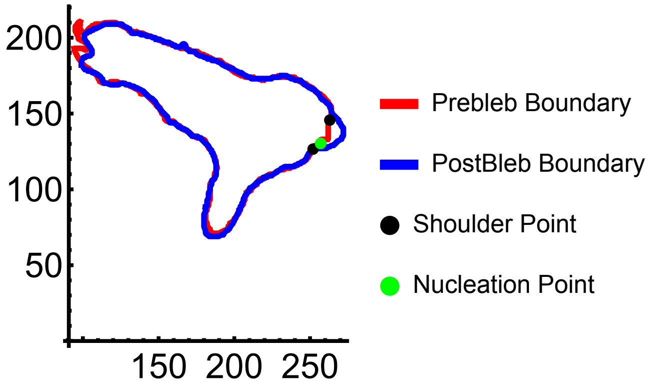

,

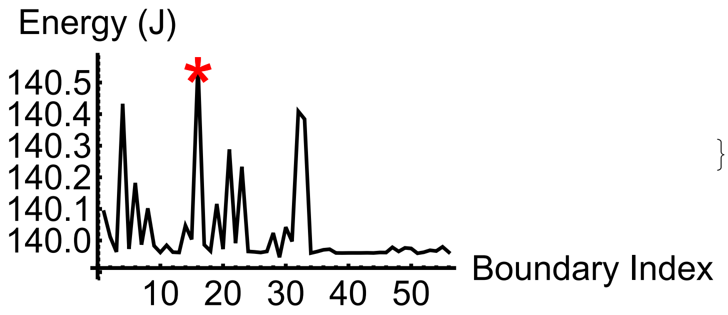

}

0423016-04

Out[•]= {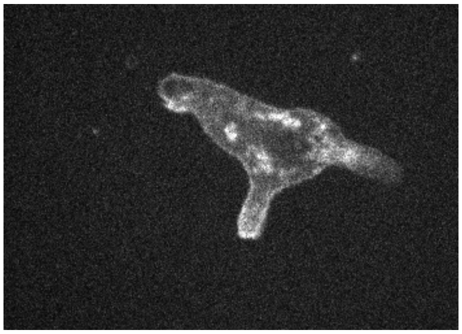, 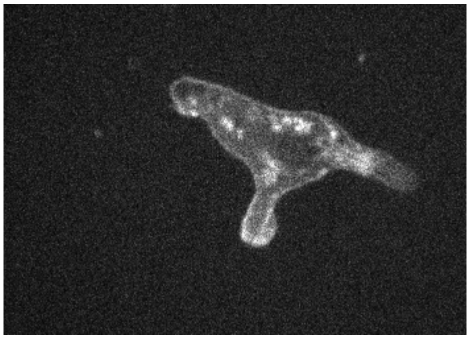,

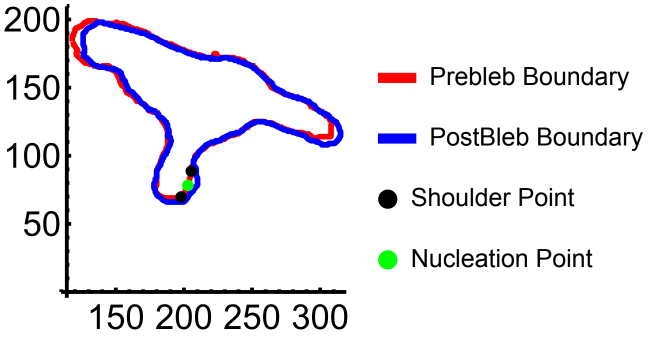

,

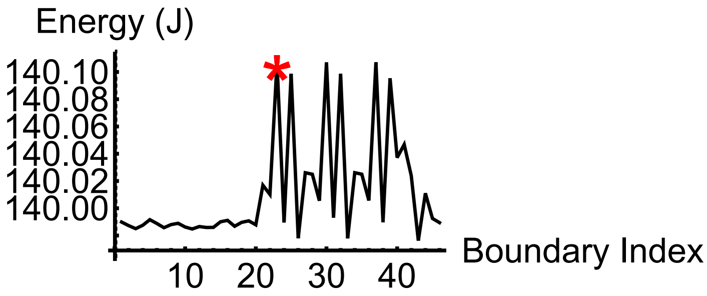

}

0423019-01

Out[•]= {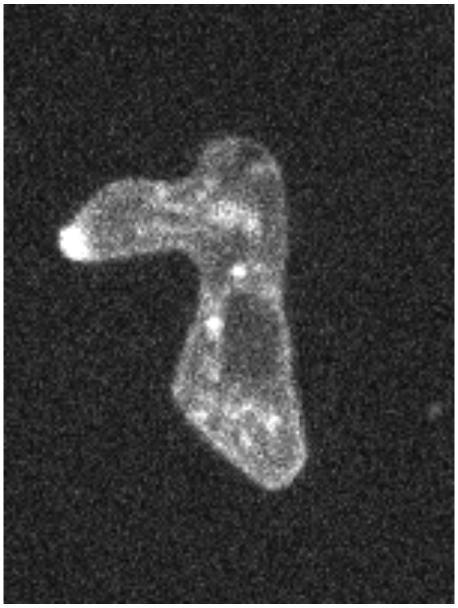, 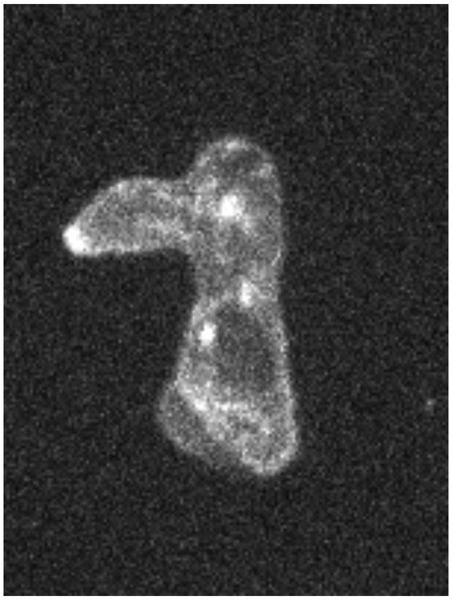,

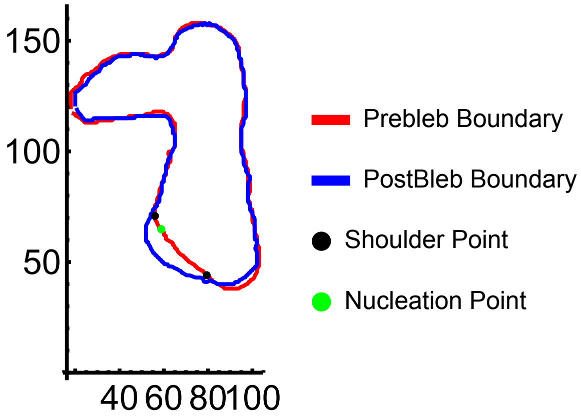

,

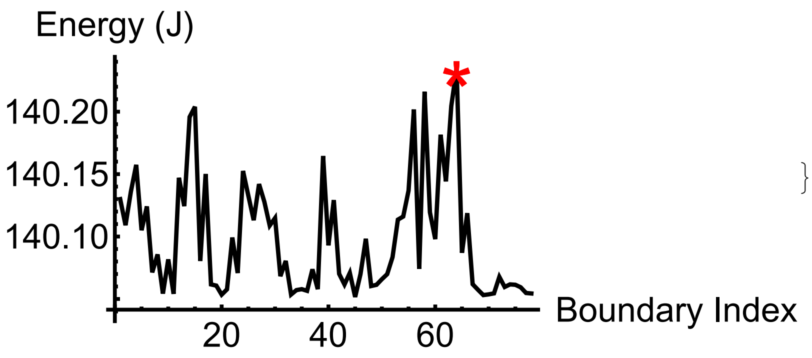

}

0423019-02

Out[ ]= {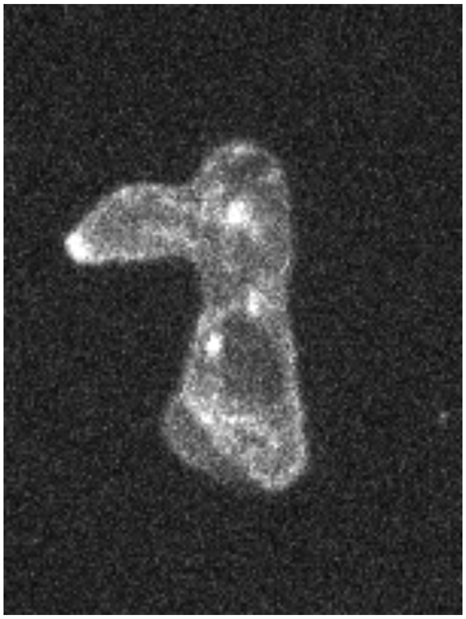,

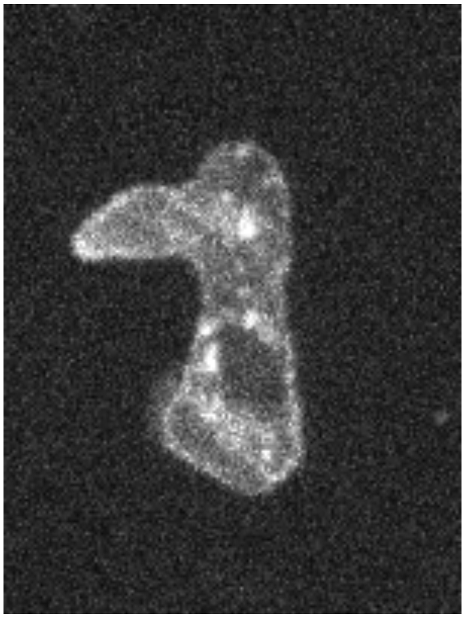,

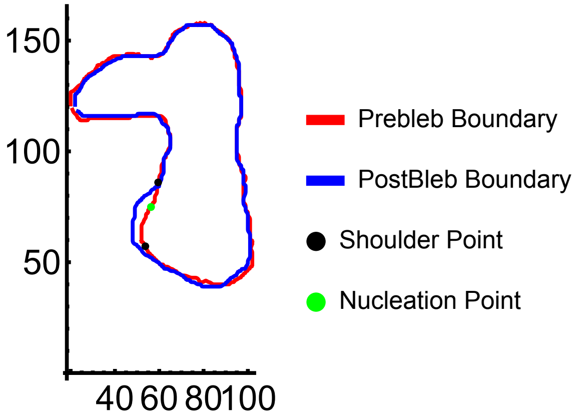
150
100
50
40 60 80100
Prebleb Boundary
PostBleb Boundary
Shoulder Point
Nucleation Point
,

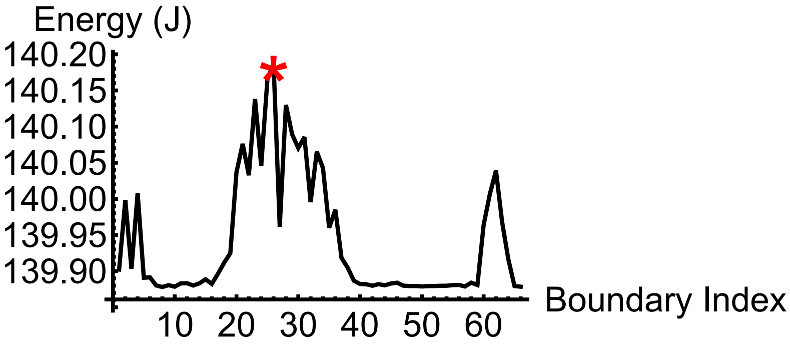
Energy (J)
140.20
140.15
140.10
140.05
140.00
139.95
139.90
*
Boundary Index
10 20 30 40 50 60
}

0423019-03

Out[∘]= {

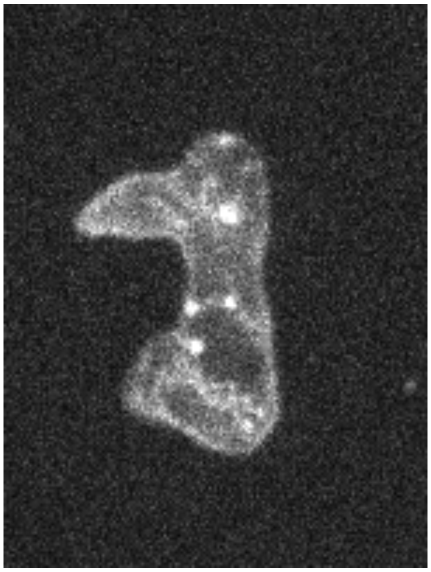

,

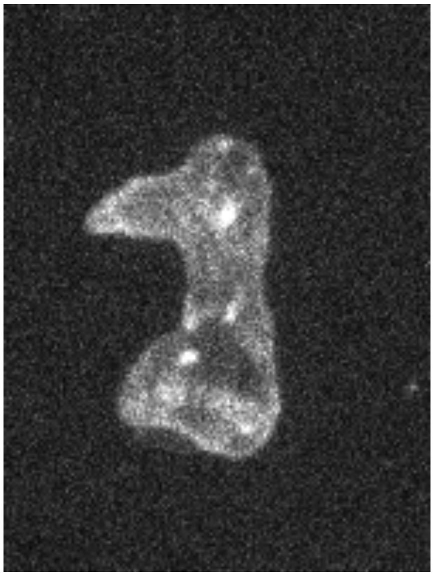

,

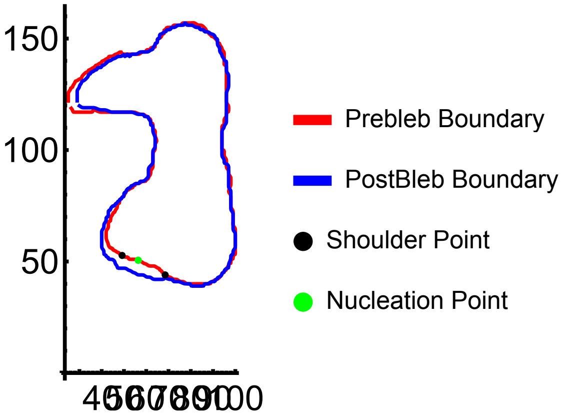

,

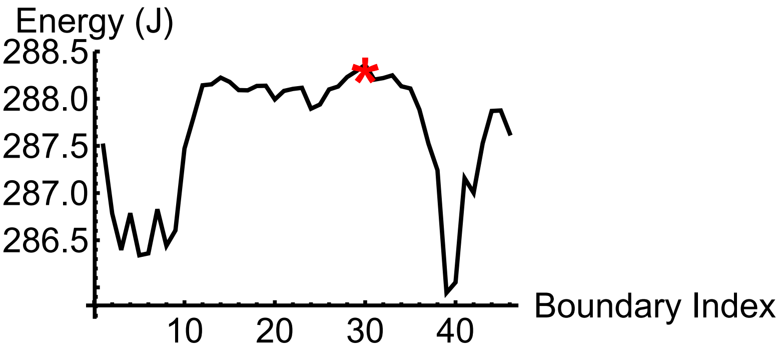

}

0423019-04

Out[•]= {

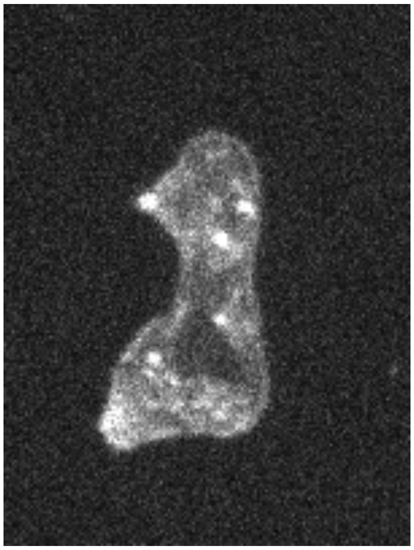

,

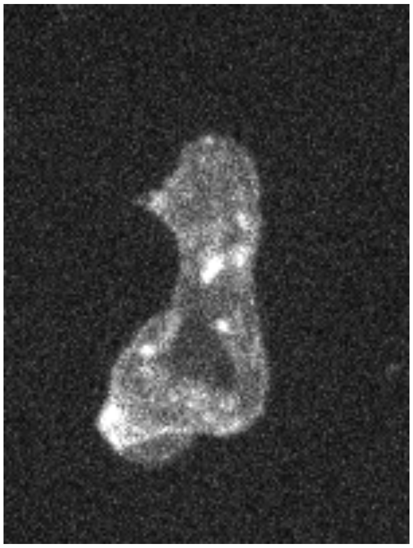

,

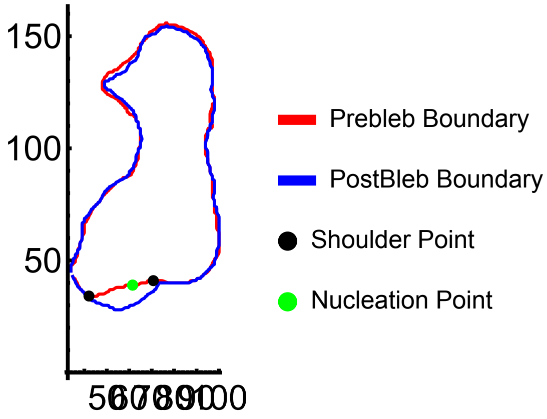

,

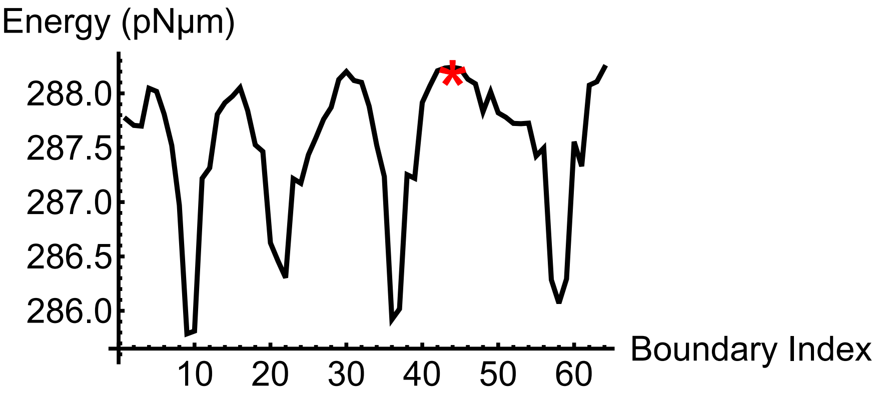

}

0423019-06

Out[●]= {

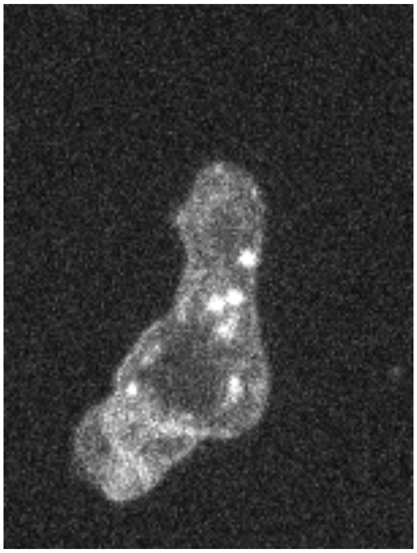

,

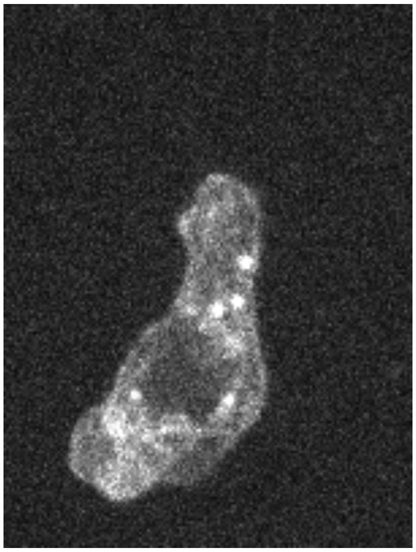

,

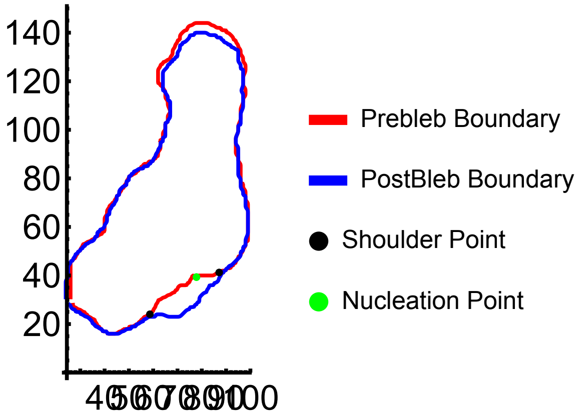

,

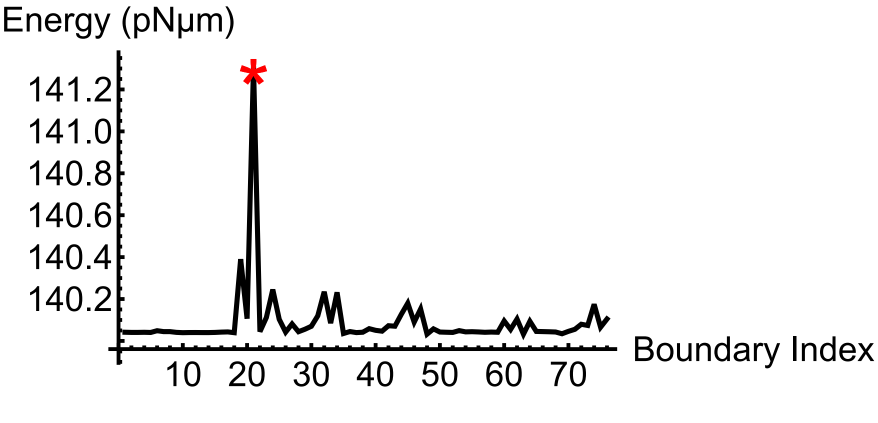

}

0423019-07

Out[ ]= {

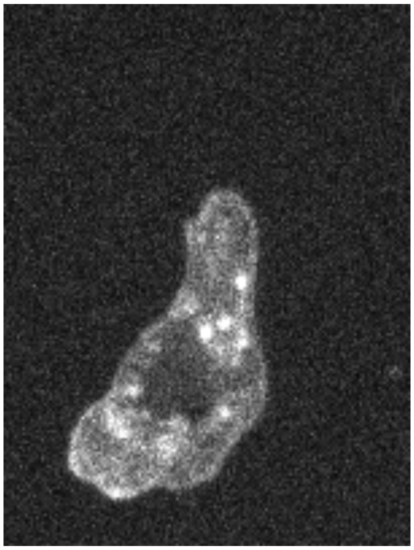

,

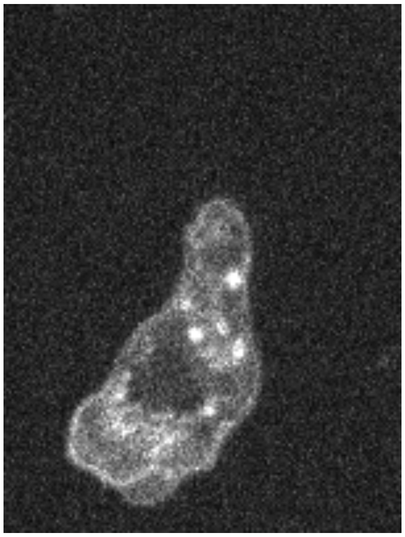

,

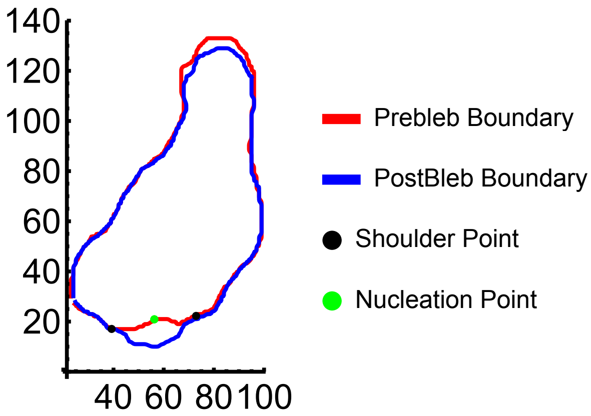

,

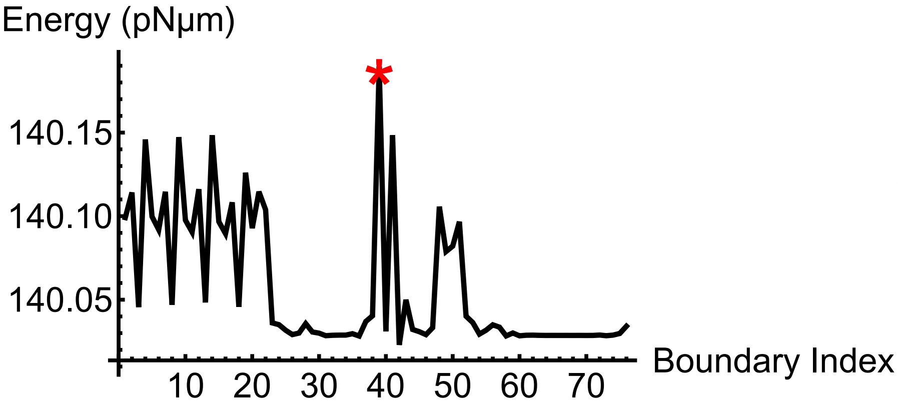

}

0423025-01

Out[•]= {

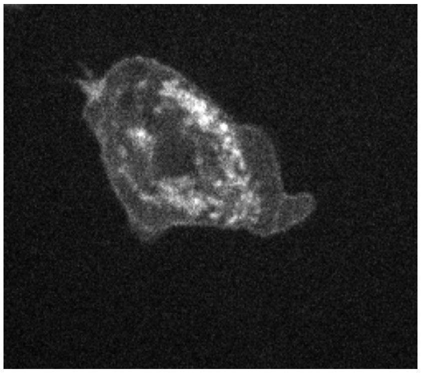

,

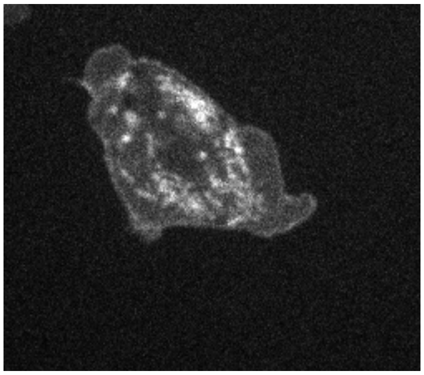

,

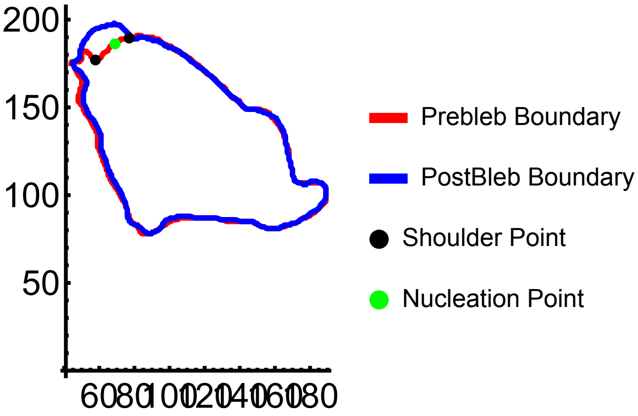

,

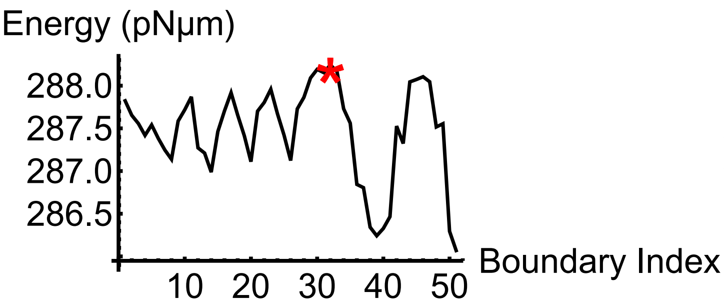

}

0423025-02

Out[•]= {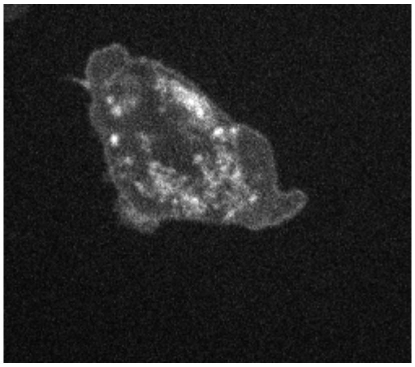, 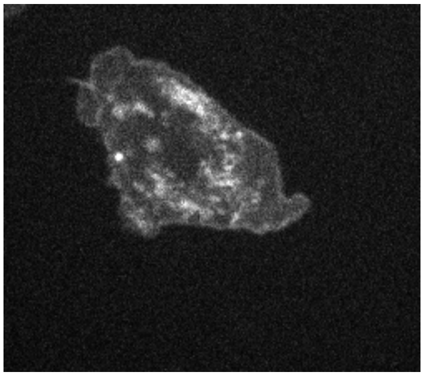,

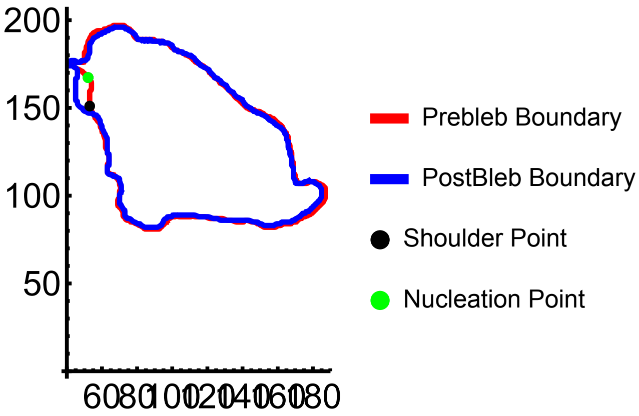

,

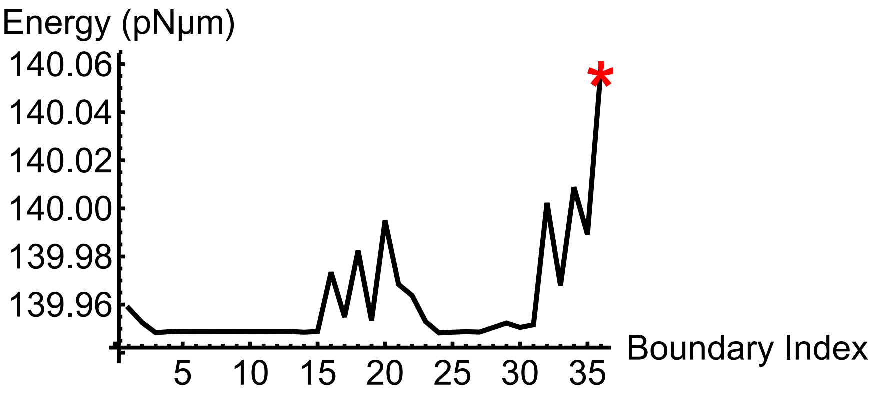

}

0423025-03

Out[•]= {

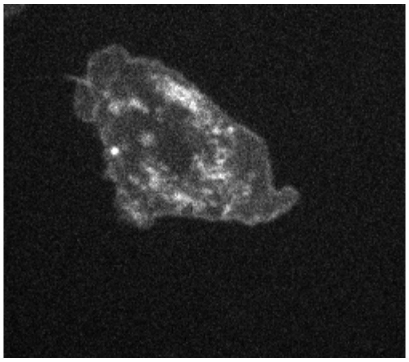

,

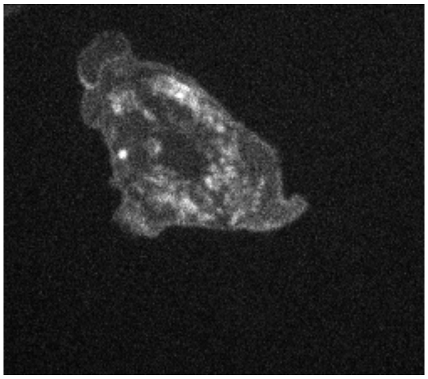

,

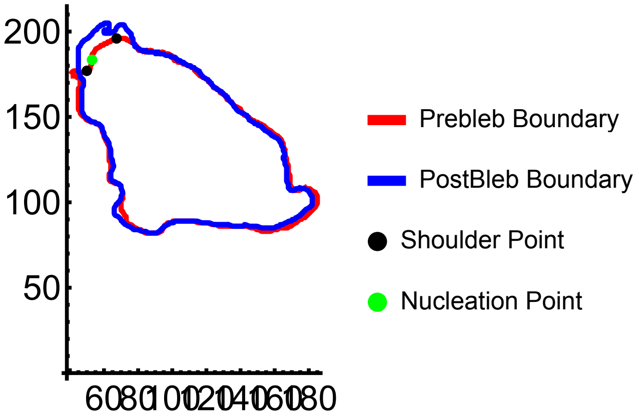

,

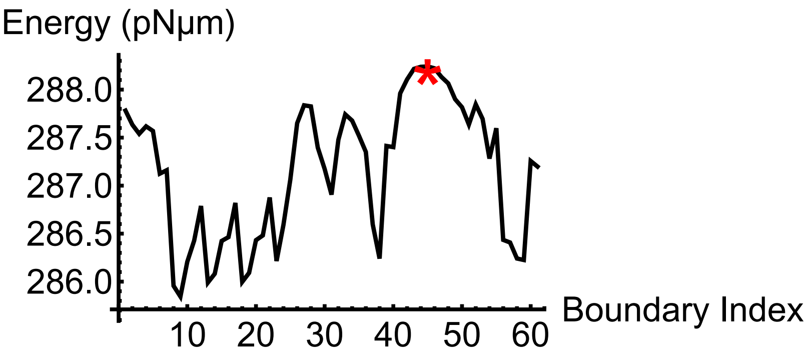

}

0423025-04

Out[ ]= {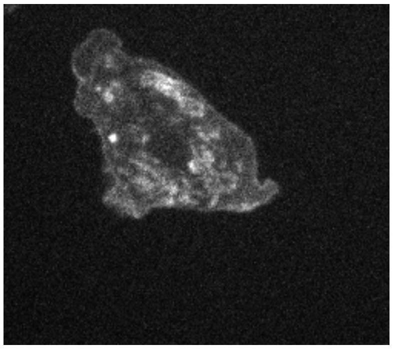, 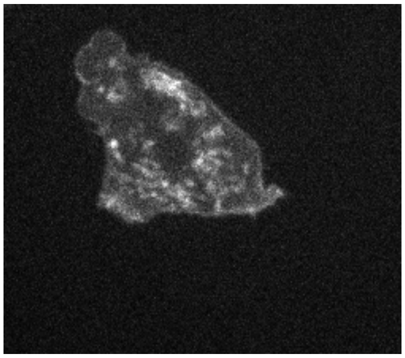,

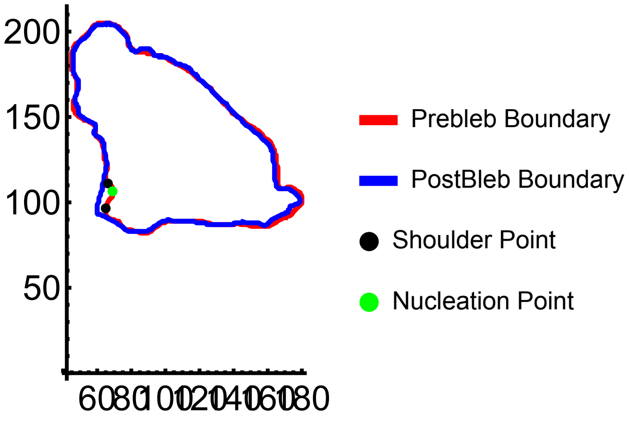

,

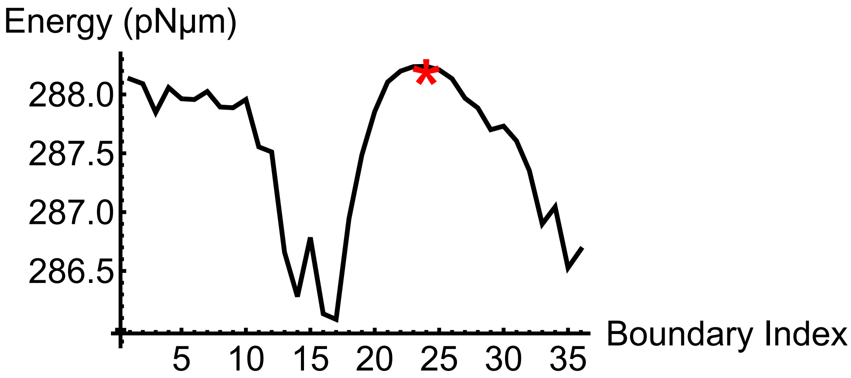

}

0423025-05

Out[ ]= {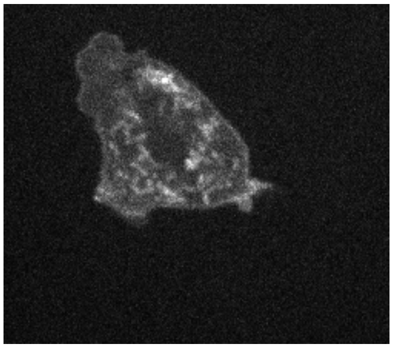, 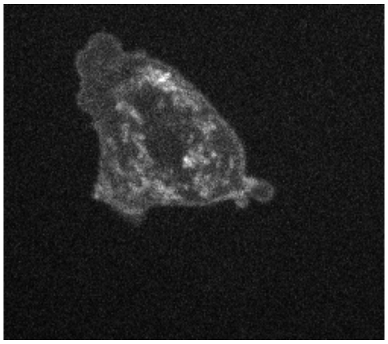,

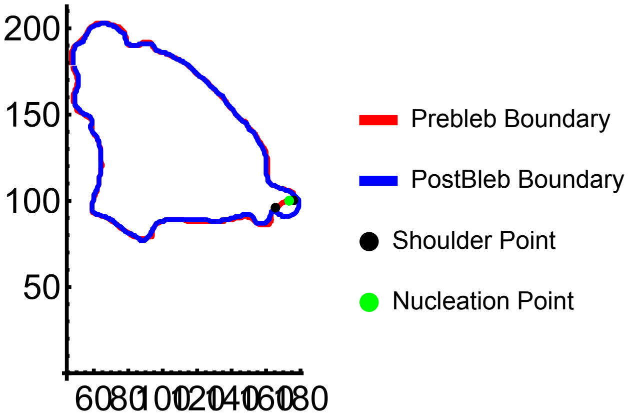

,

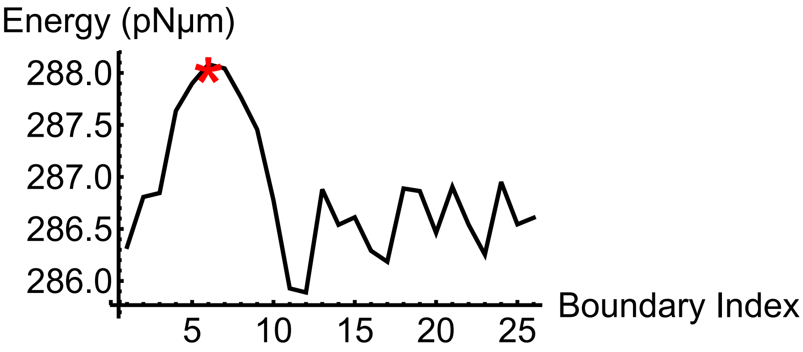

}

0423025-06

Out[•]= {

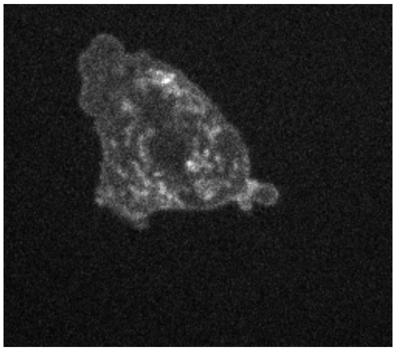

,

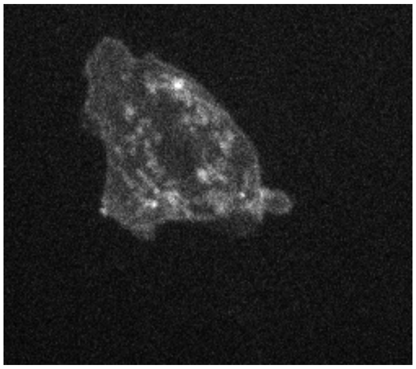

,

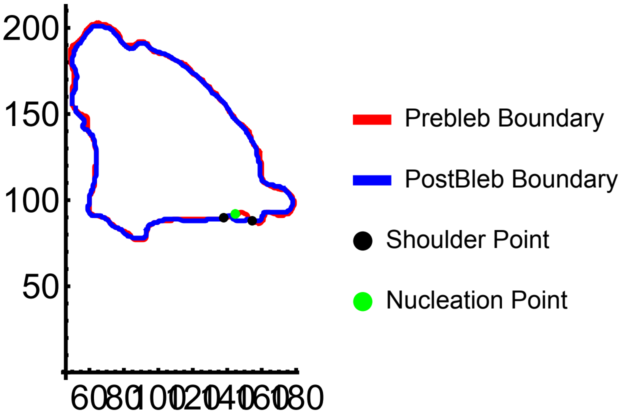

,

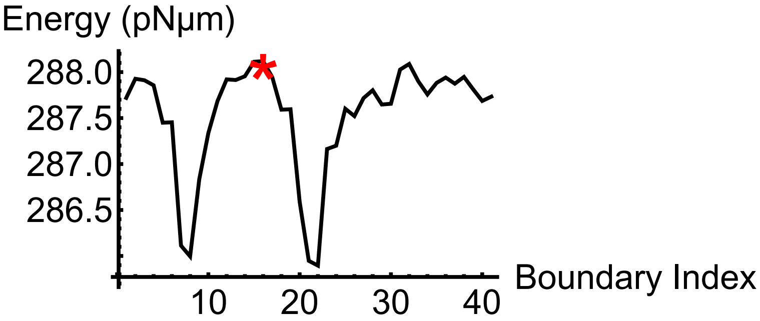

}

0423025-07

Out[•]= {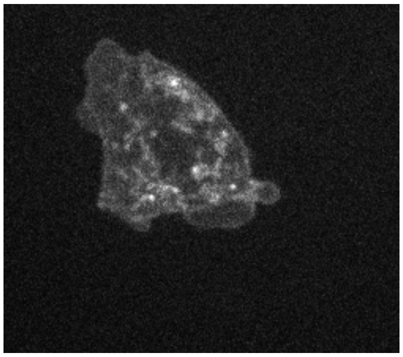, 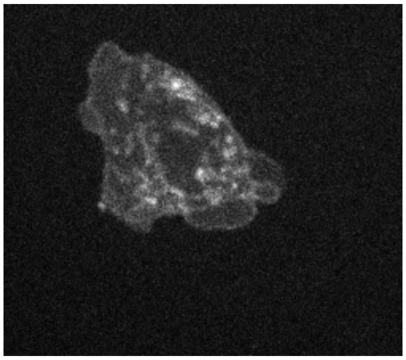,

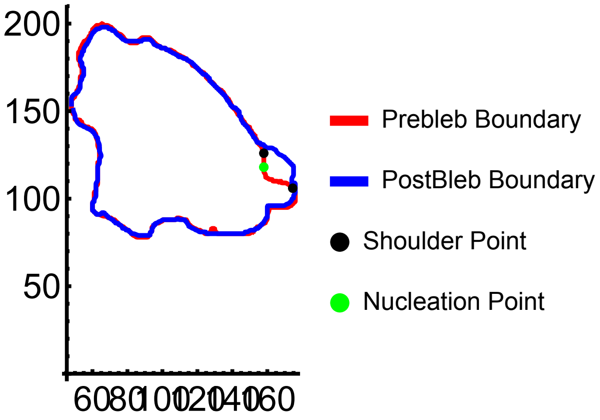

,

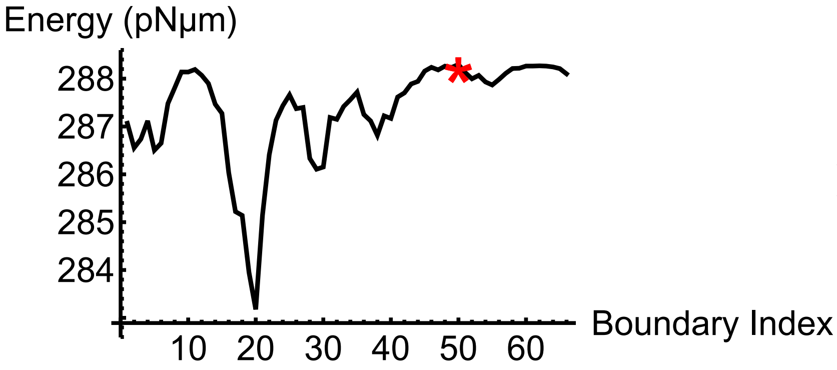

}

0423025-08

Out[ ]= {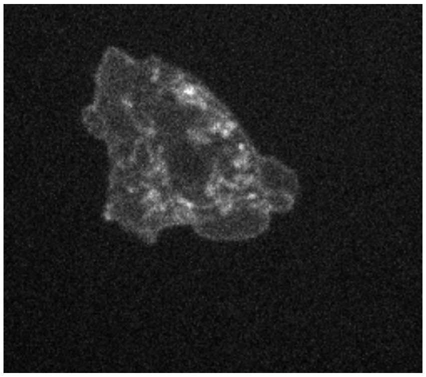, 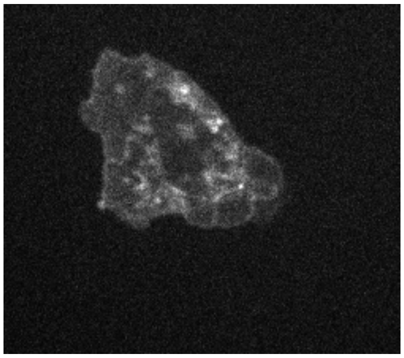,

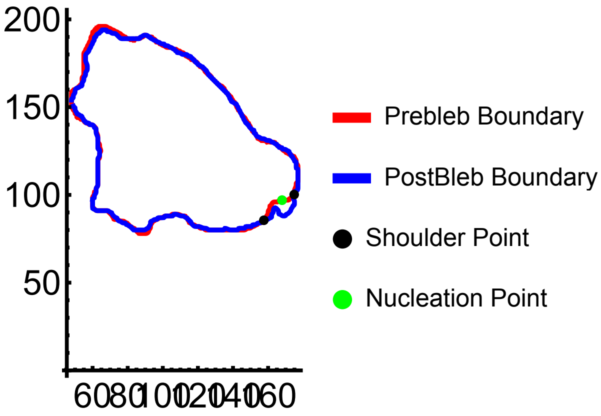

,

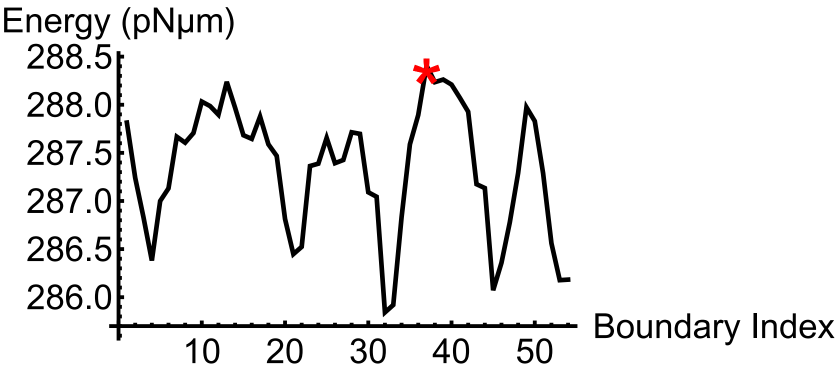

}

0423026-01

Out[ ]= {

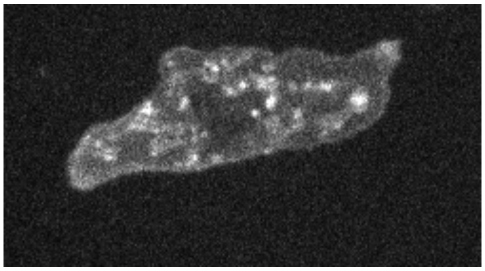

,

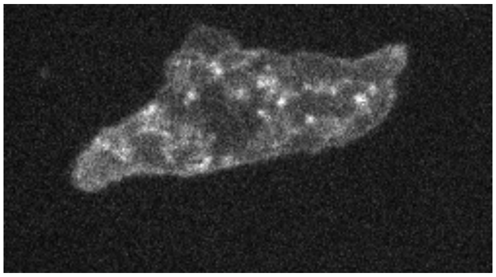

,

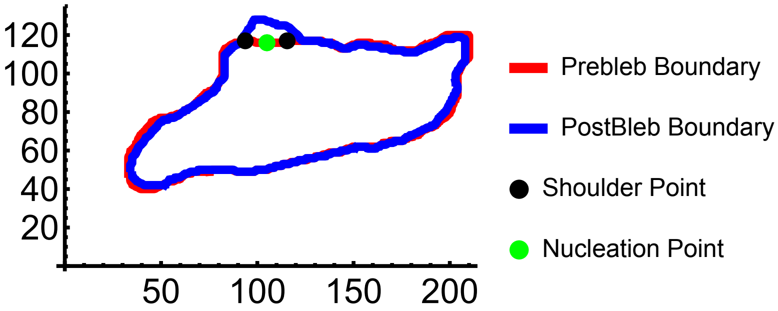

,

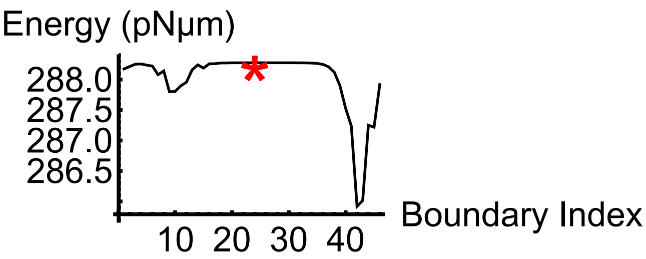

}

0423026-02

Out[•]= {

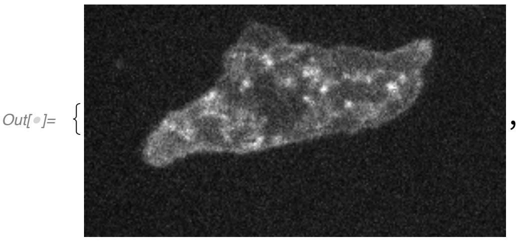

,

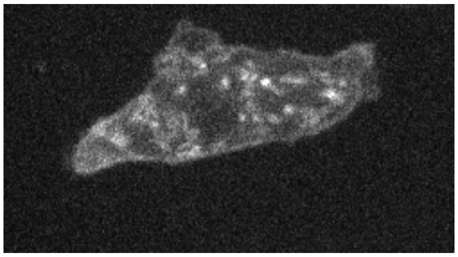

,

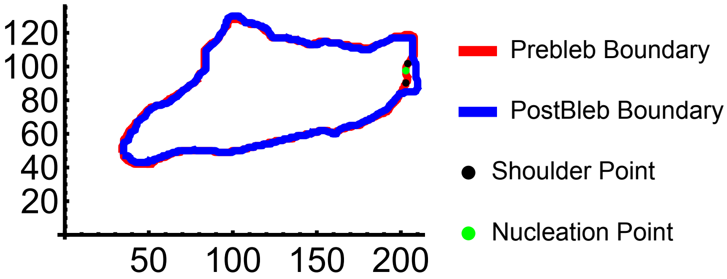

,

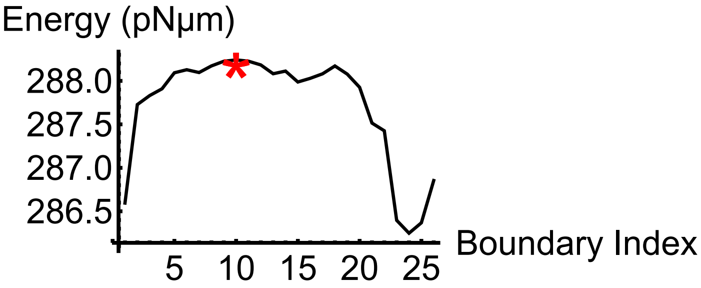

}

0423026-03

Out[ ]= {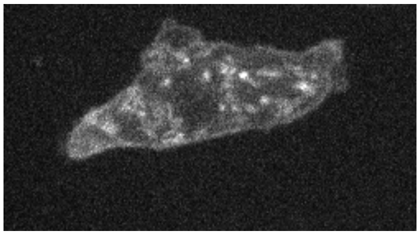, 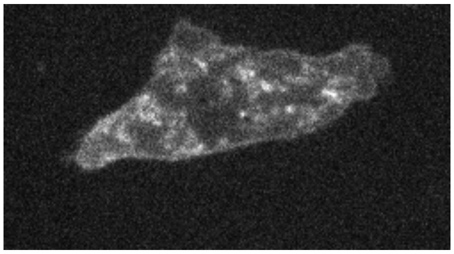,

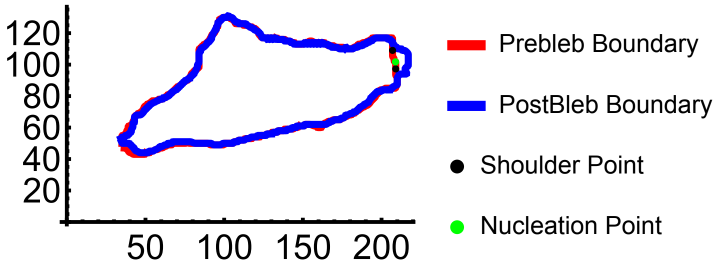

,

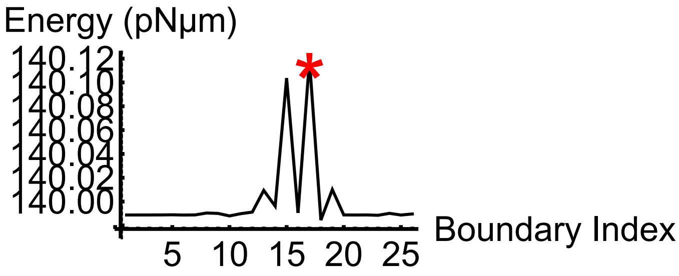

}

0423026-05

Out[•]= {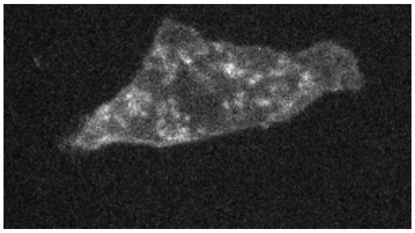, 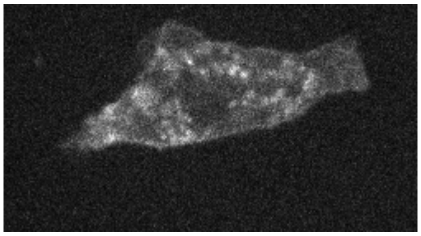,

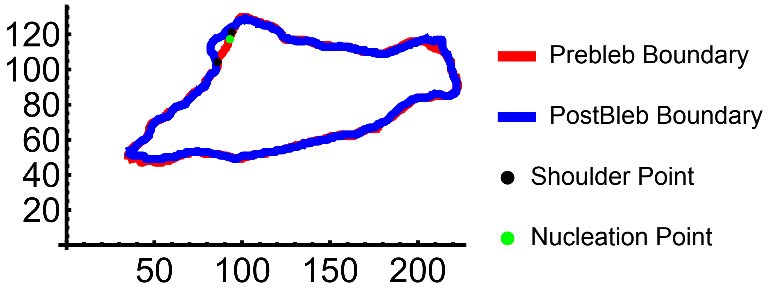

,

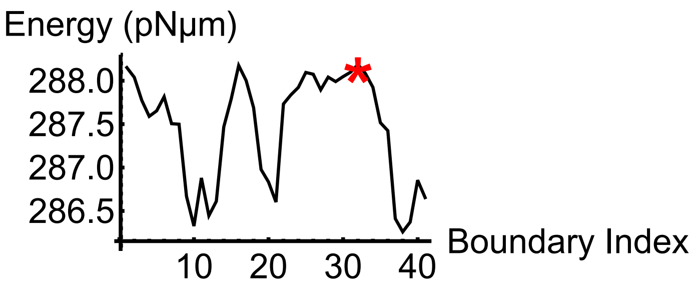

}

0423026-06

Out[ ]= {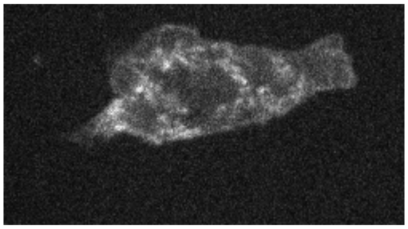, 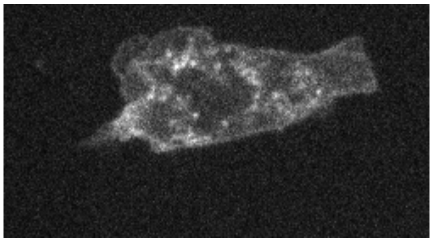,

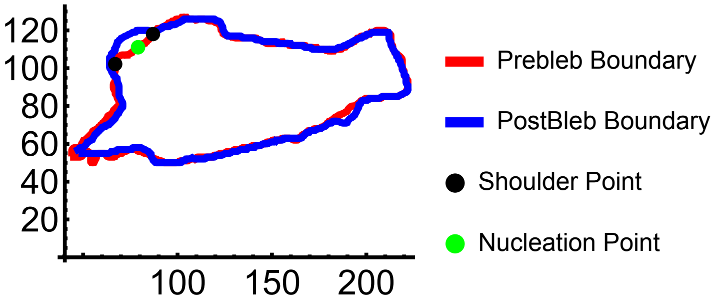

,

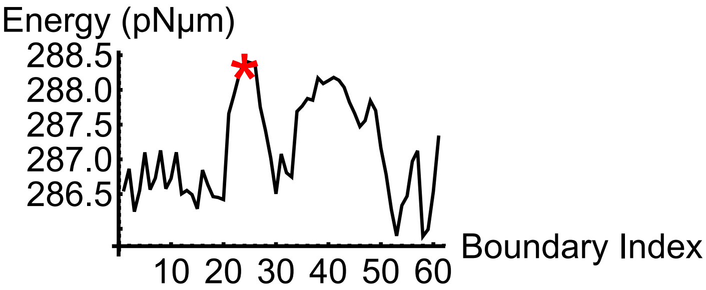

}

0423026-07

Out[ ]= {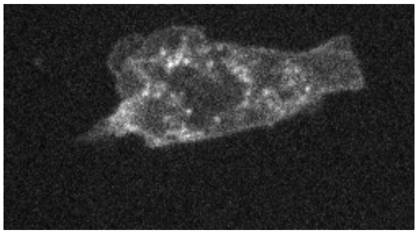, 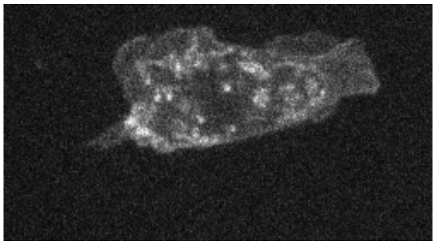,

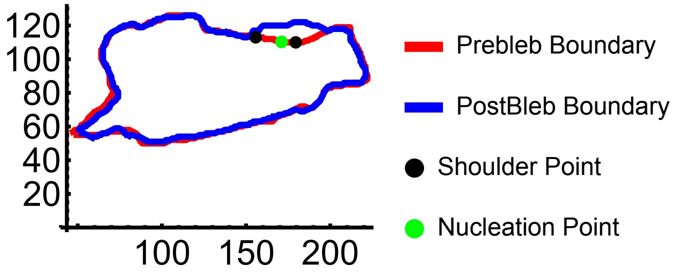

,

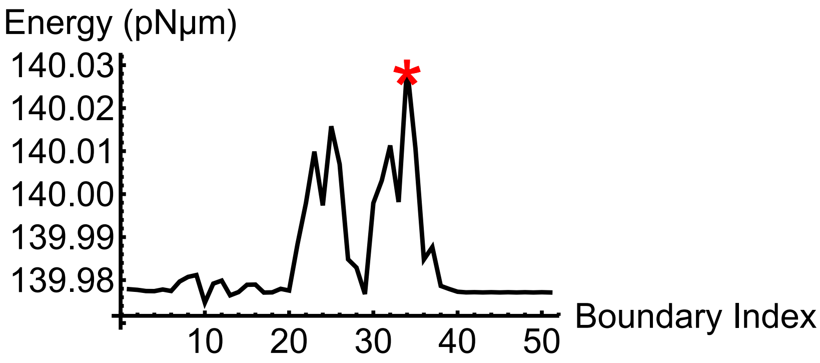

}

0423026-08

Out[]= {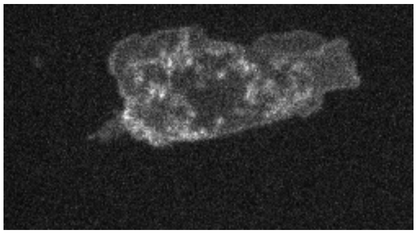, 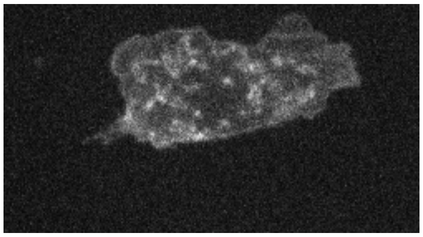,

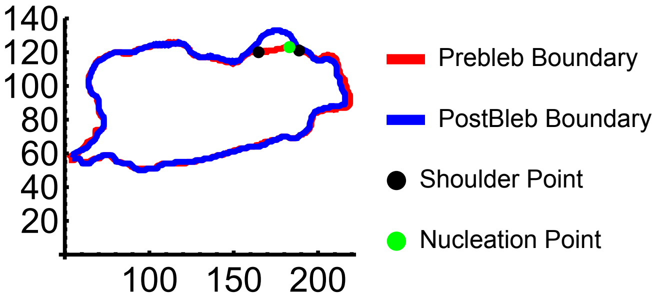

,

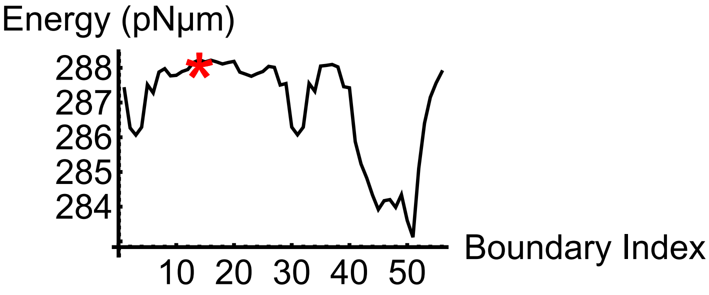

}

0423026-09

Out[ ]= {

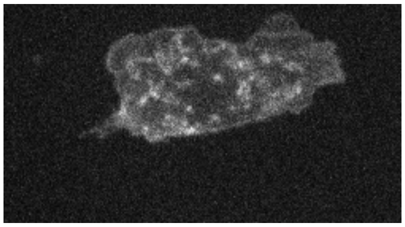

,

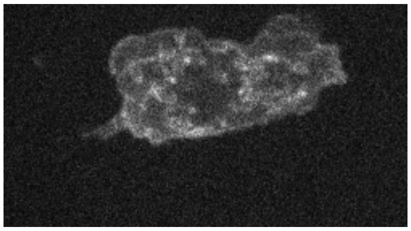

,

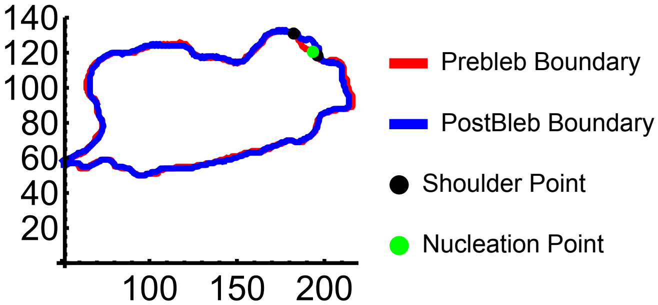

,

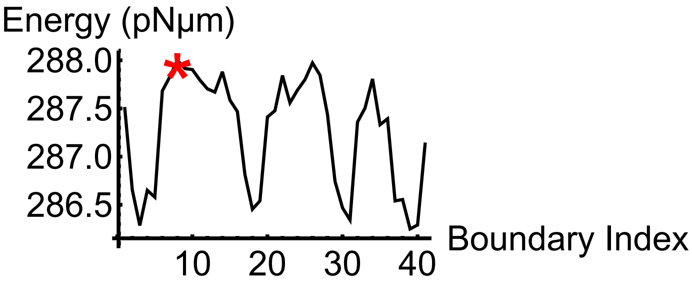

}

0423026-10

Out[ ]= {

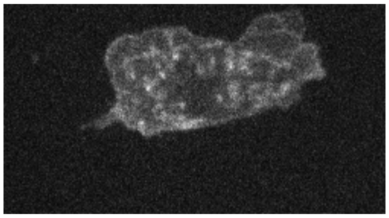

,

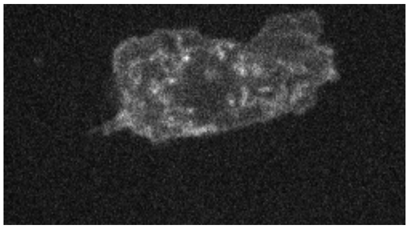

,

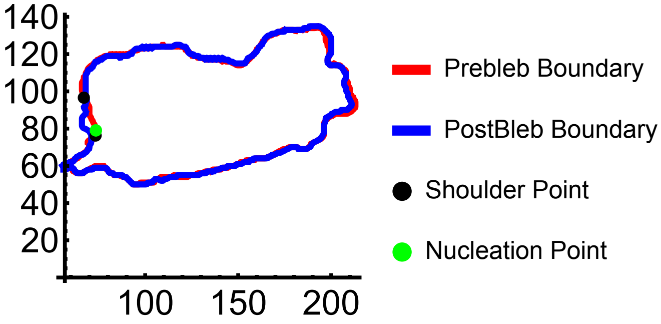

,

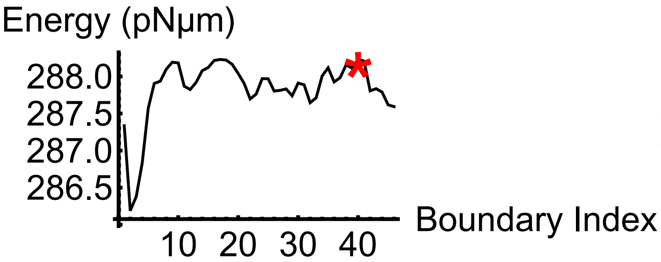

}

0423026-11

Out[ ]= {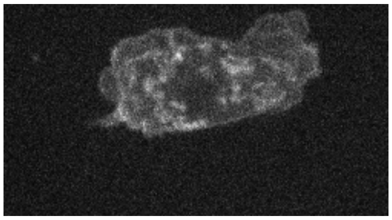, 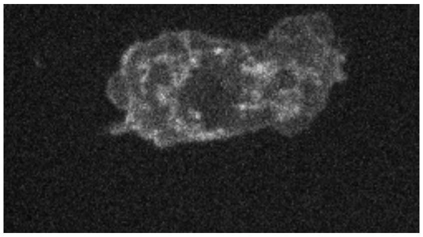,

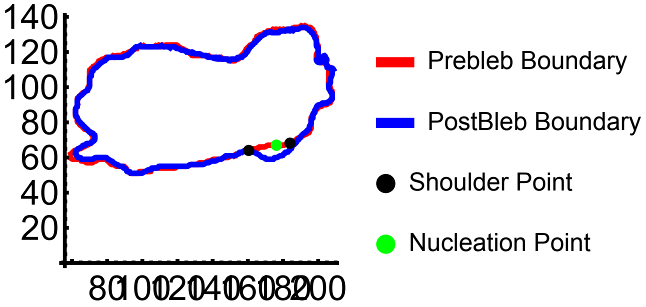

,

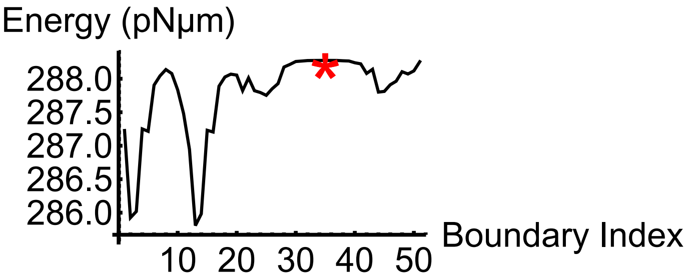

}

0423026-13

Out[•]= {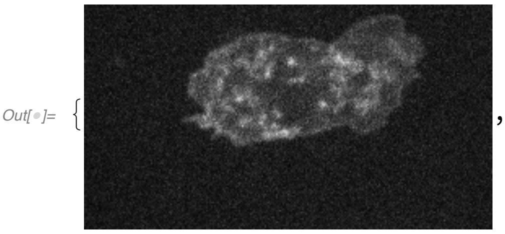, 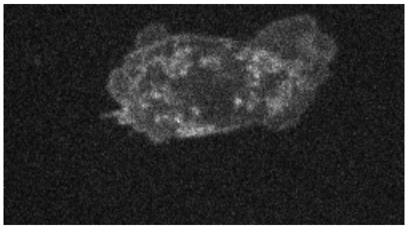,

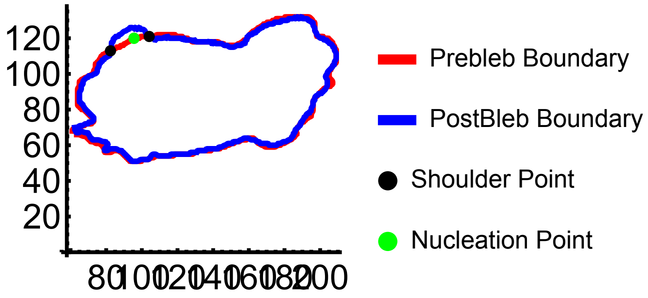

,

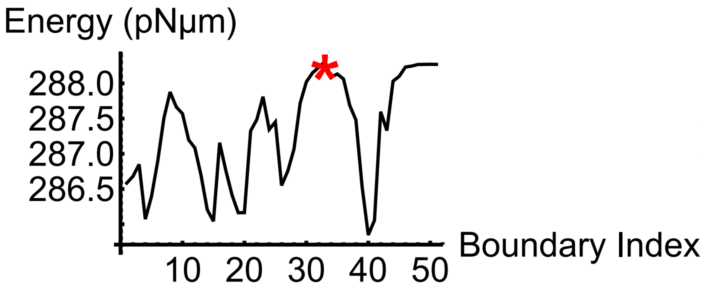

}

0423026-14

Out[•]= {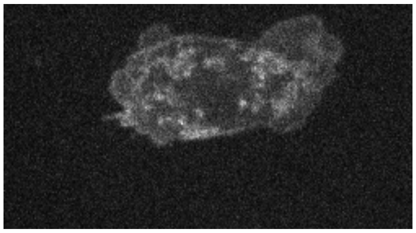, 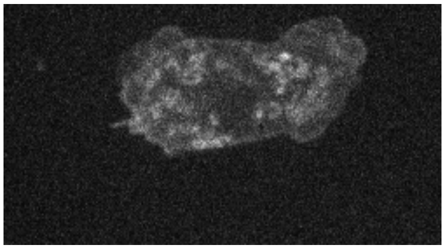,

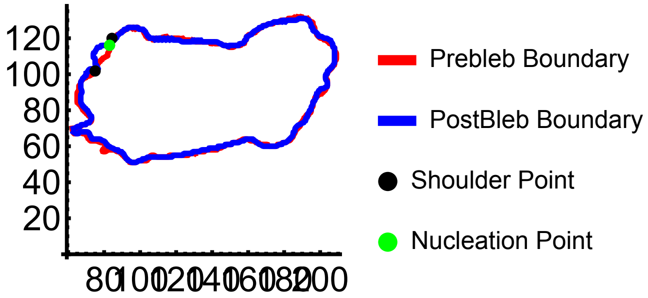

,

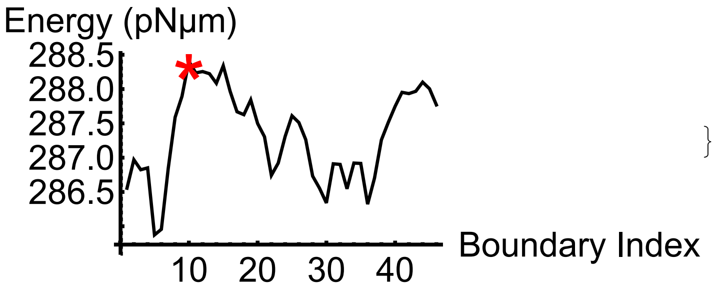

}

0423030-01

Out[•]= {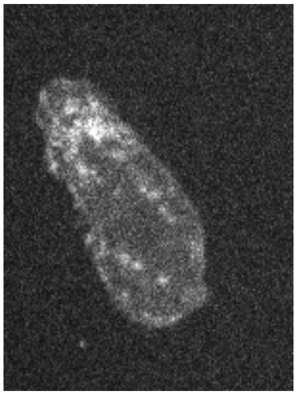, 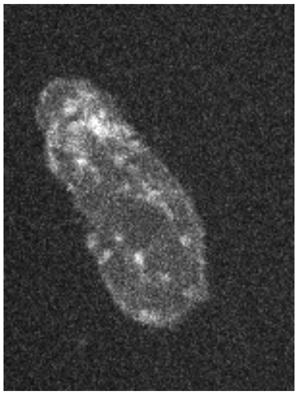,

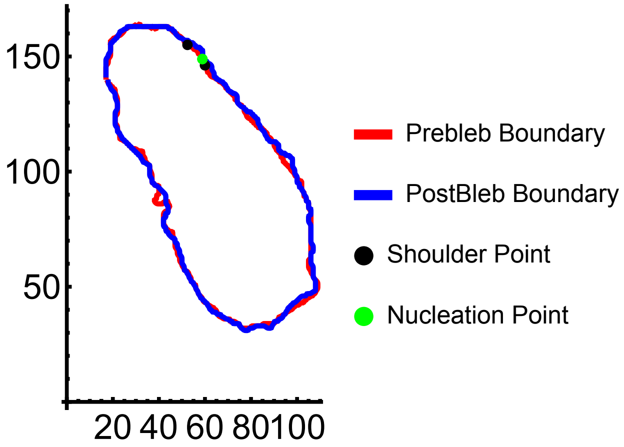

,

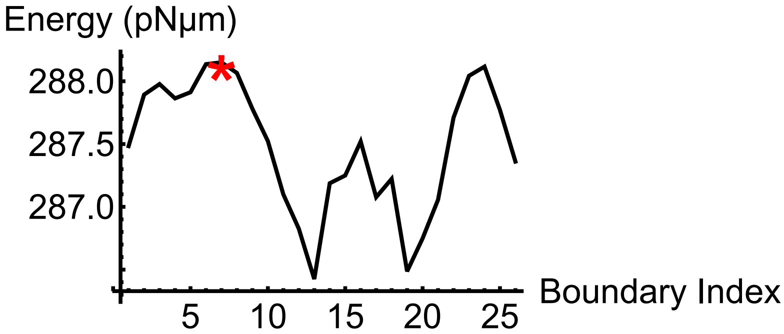

}

0423030-02

Out[ ]= {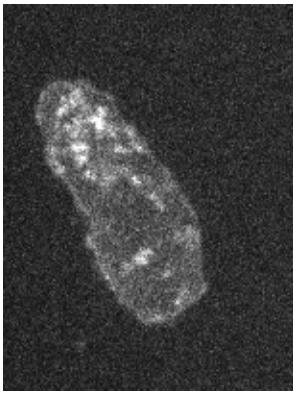, 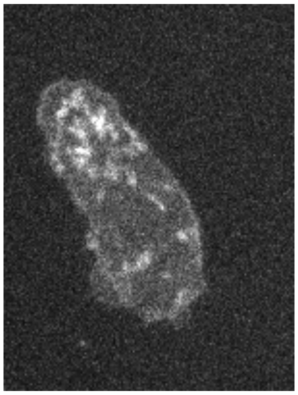,

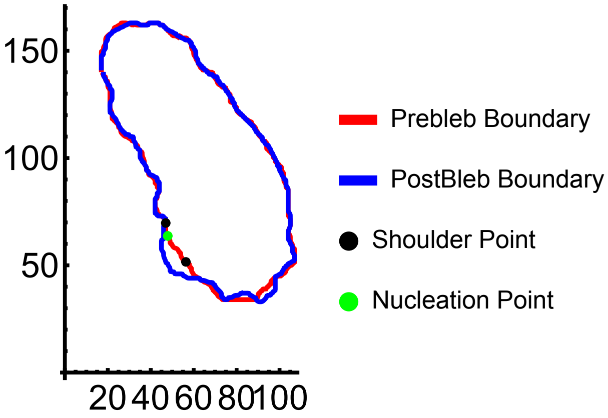

,

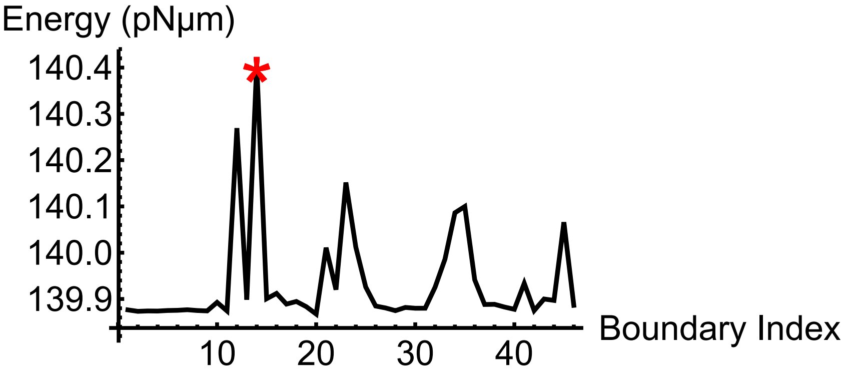

}

0423030-03

Out[ ]= {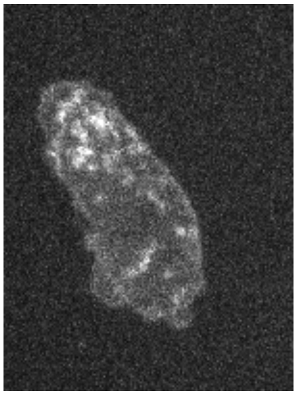, 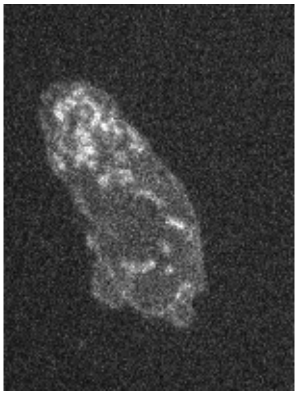,

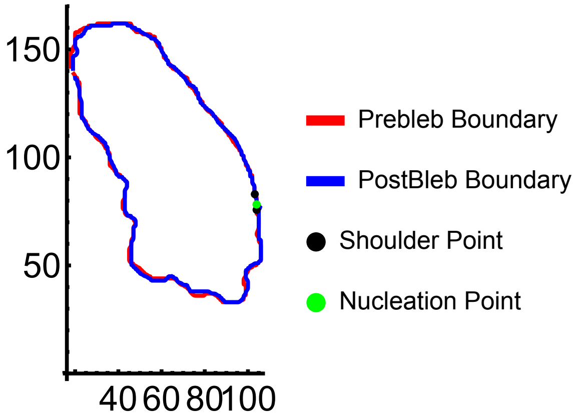

,

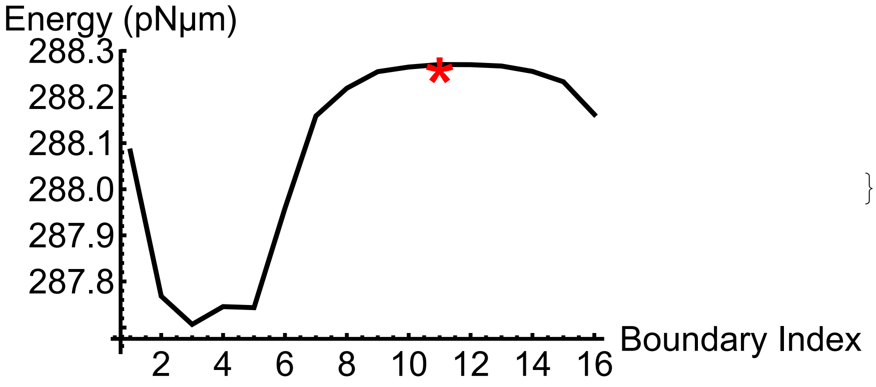

}

0423030-05

Out[•]= {

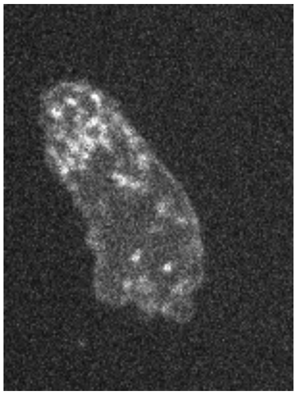

,

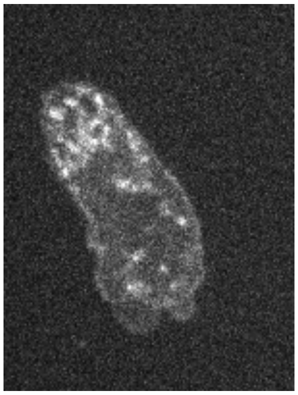

,

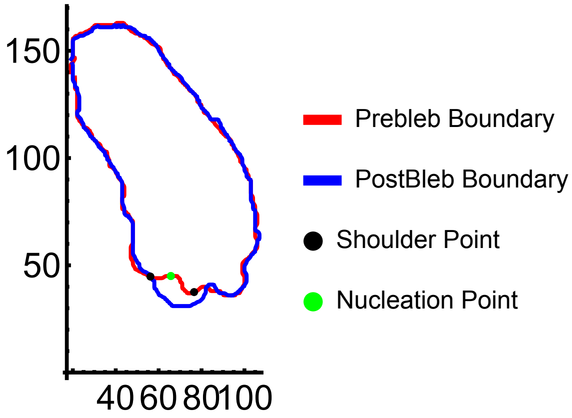

,

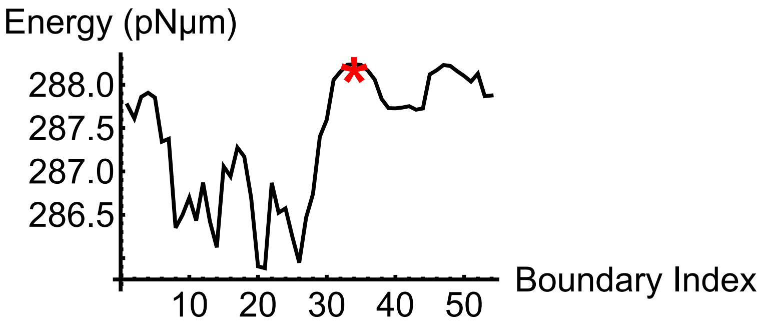

}

Bleb Nucleation Predictions for Experiment 0615

0615003-01

Out[•]= {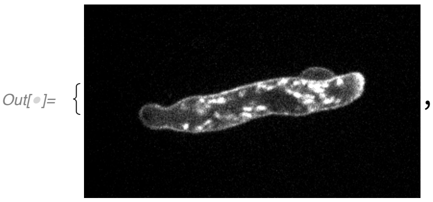, 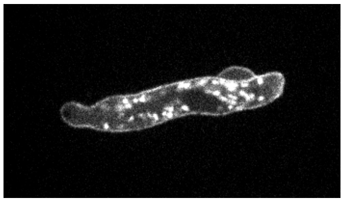,

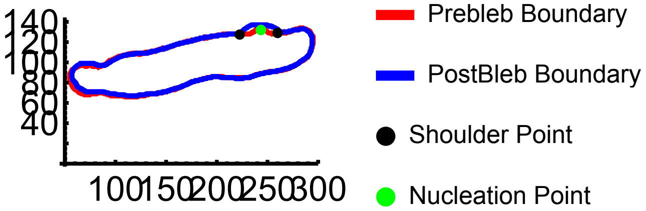

,

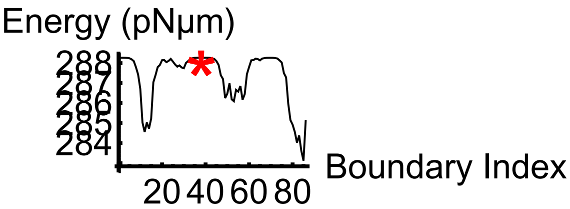

}

0615004-02

Out[•]= {

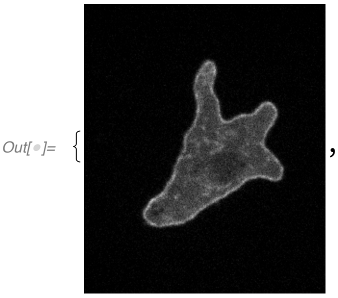

,

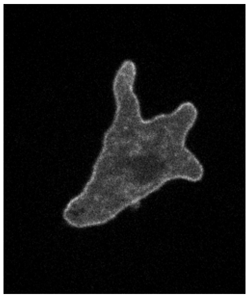

,

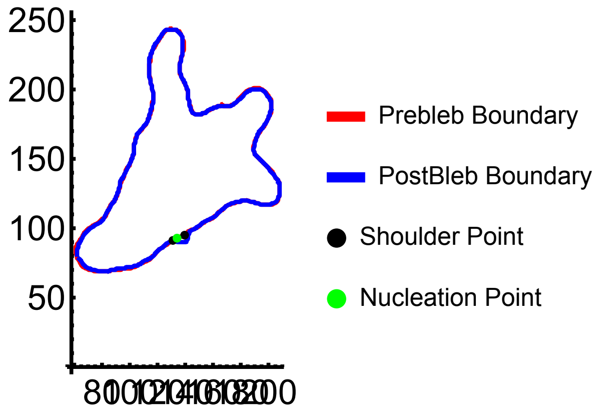

,

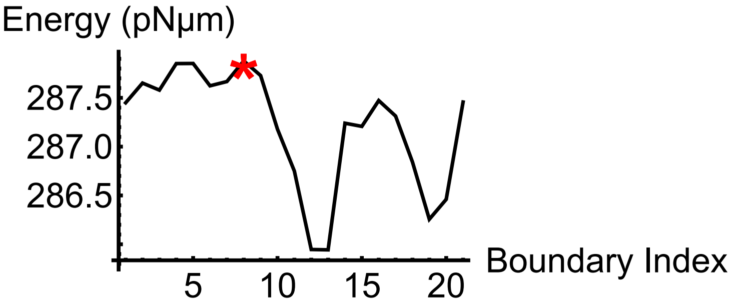

}

0615004-03

Out[•]= {

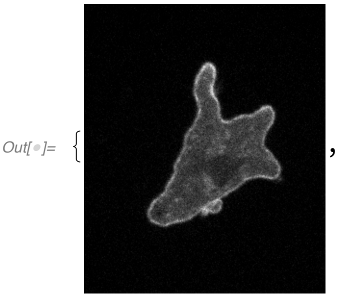

,

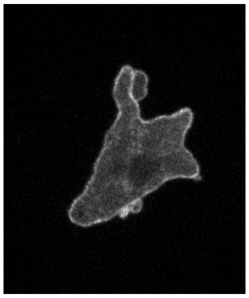

,

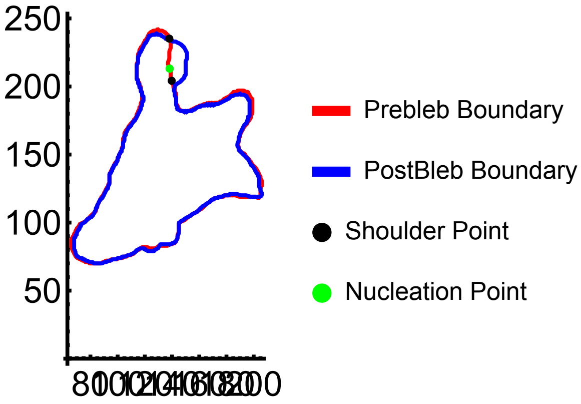

,

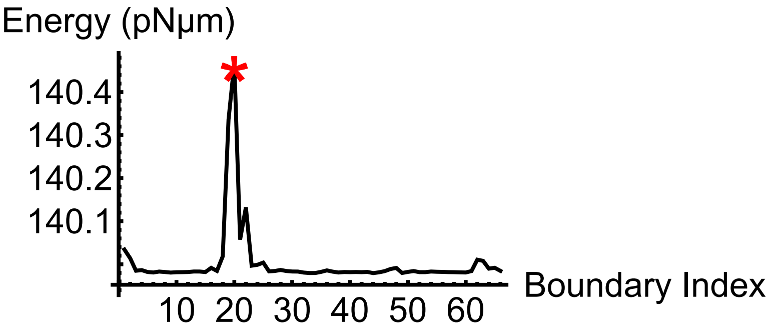

}

0615004-04

Out[ ]= {

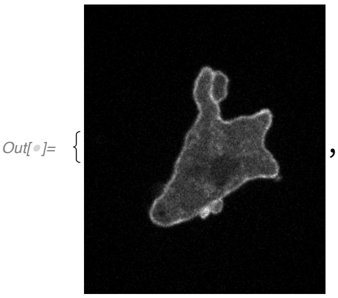

,

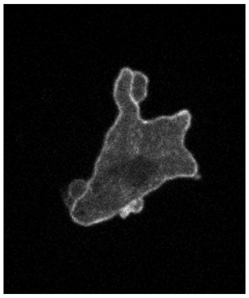

,

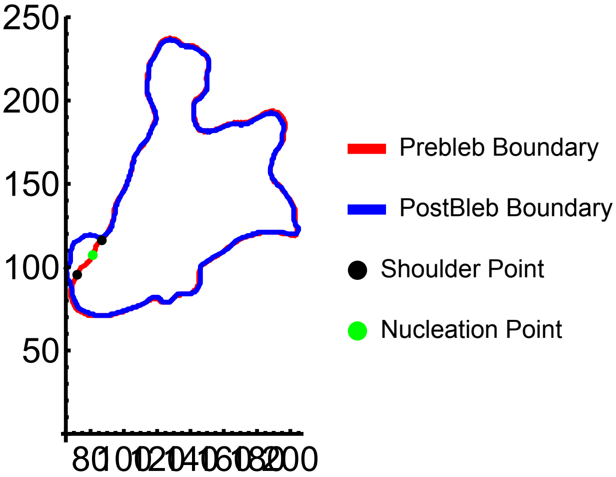

,

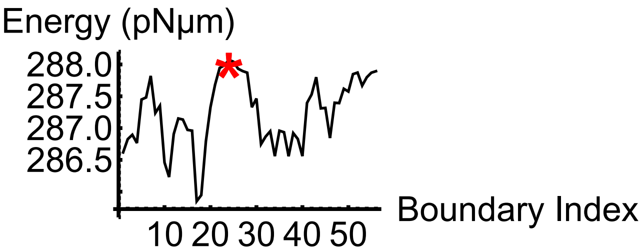

}

0615004-05

Out[ ]= {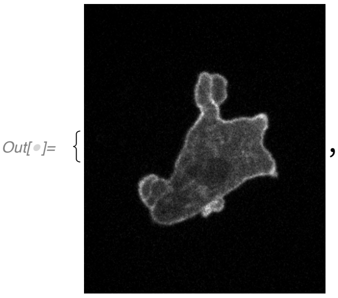, 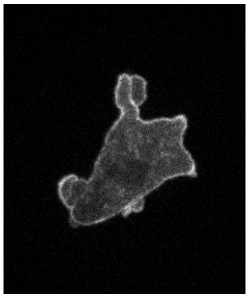, 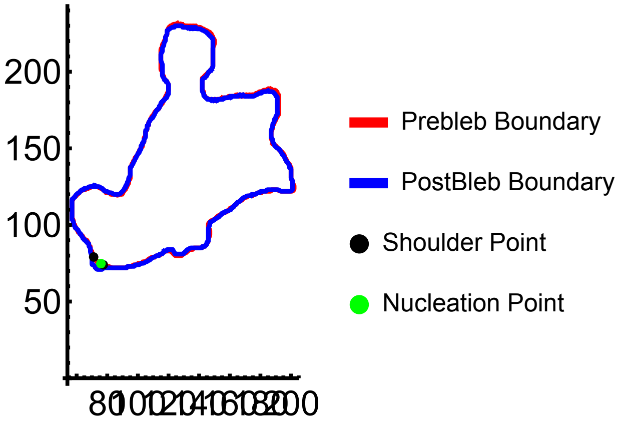

,
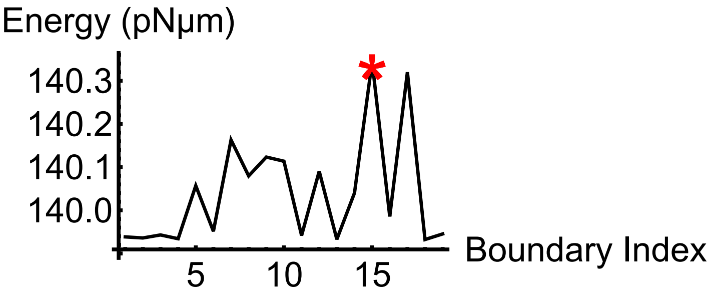

}

0615005-01

Out[ ]= {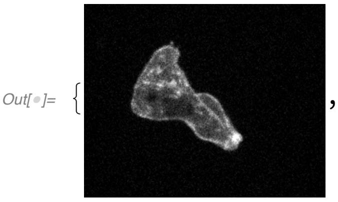, 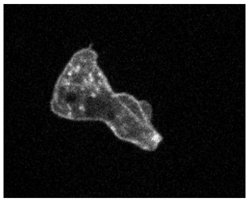, 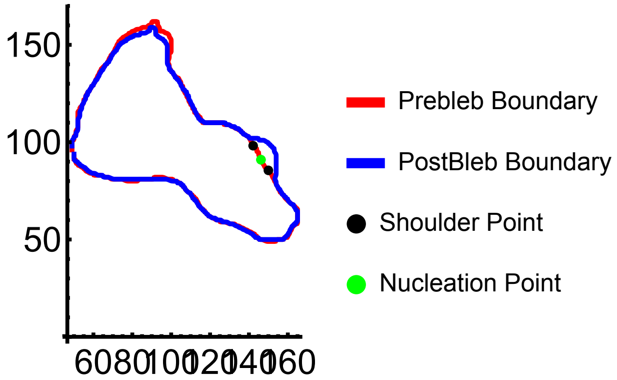

,
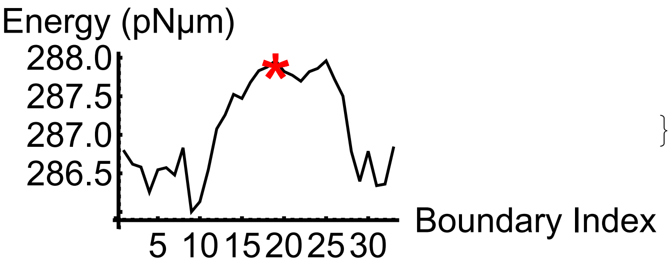

}

0615005-02

Out[•]= {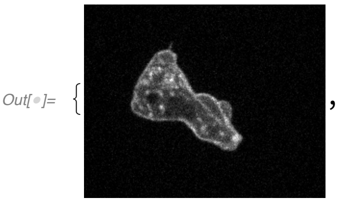, 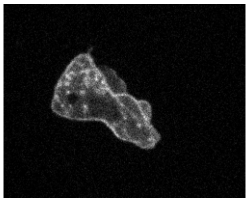, 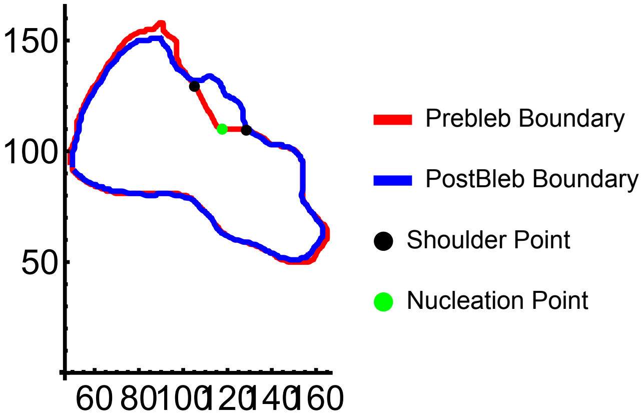

,
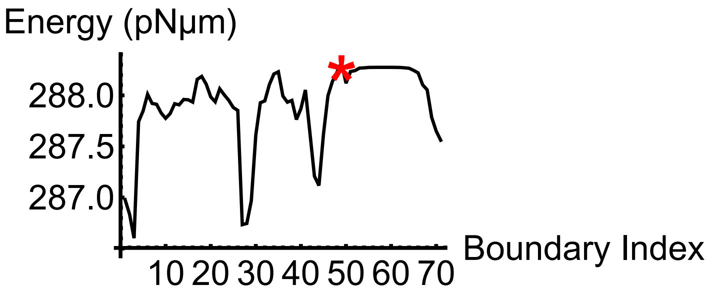

}

0615005-03

Out[ ]= {

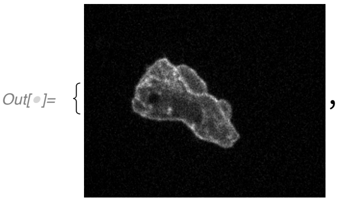

,

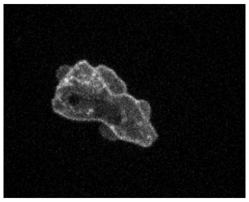

,

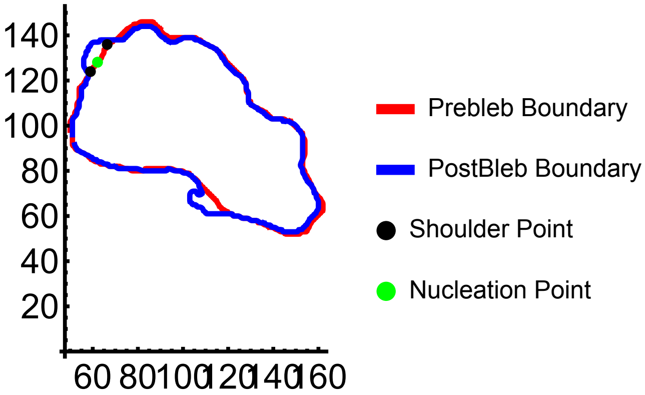

,

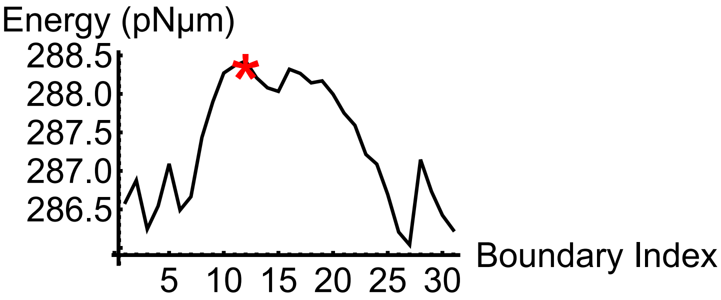

}

0615005-04

Out[ ]= {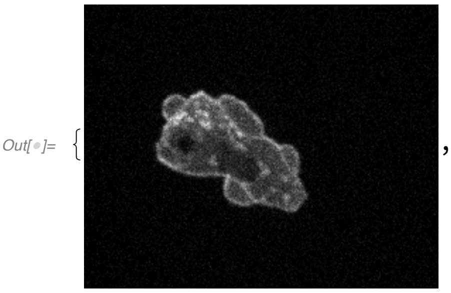, 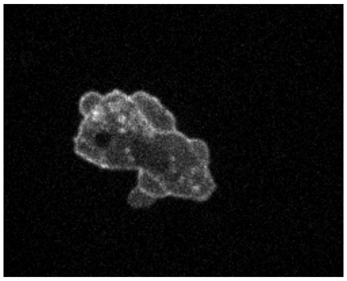,

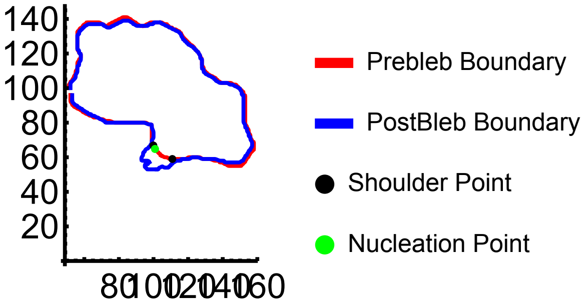

,

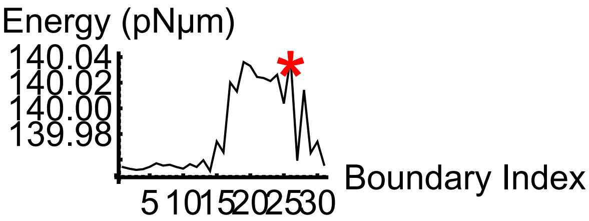

}

0615005-05

Out[•]= {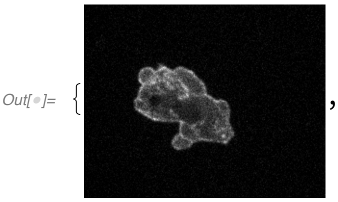, 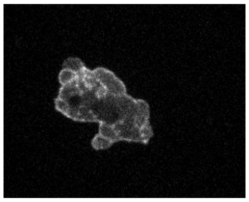, 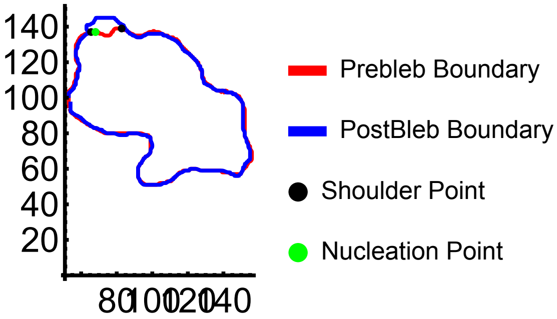

,

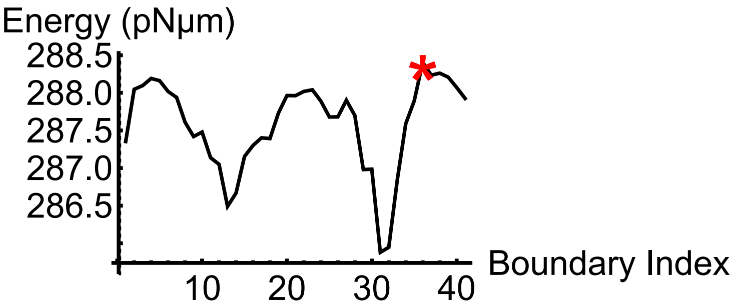

}

0615005-06

Out[•]= {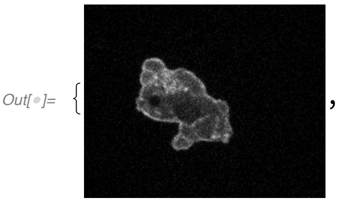, 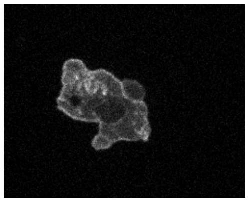, 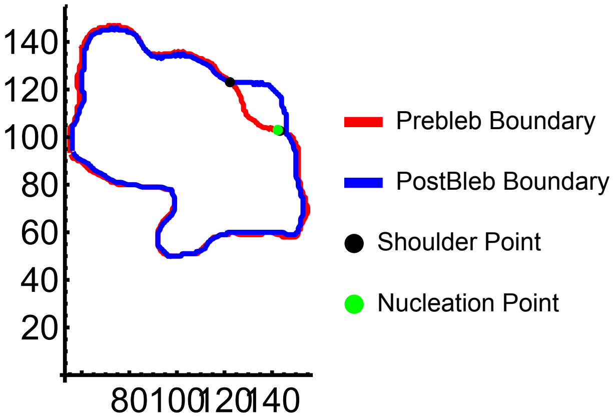

,

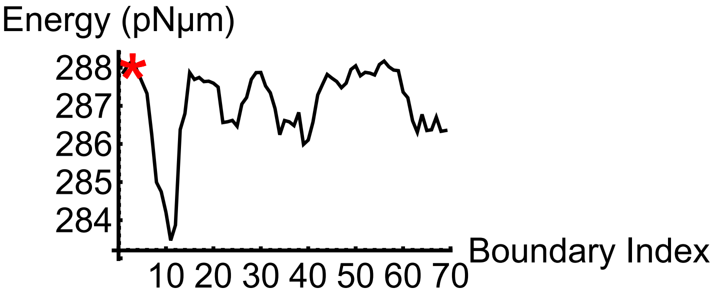

}

0615006-01

Out[ ]= { 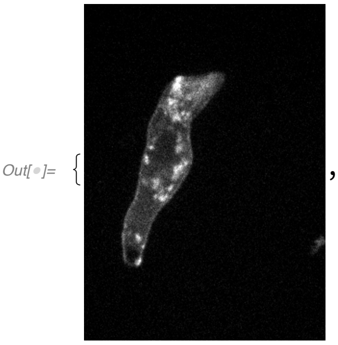, 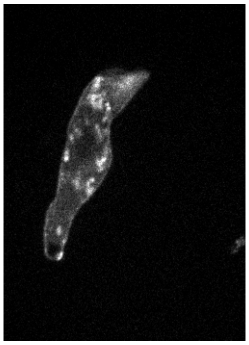, 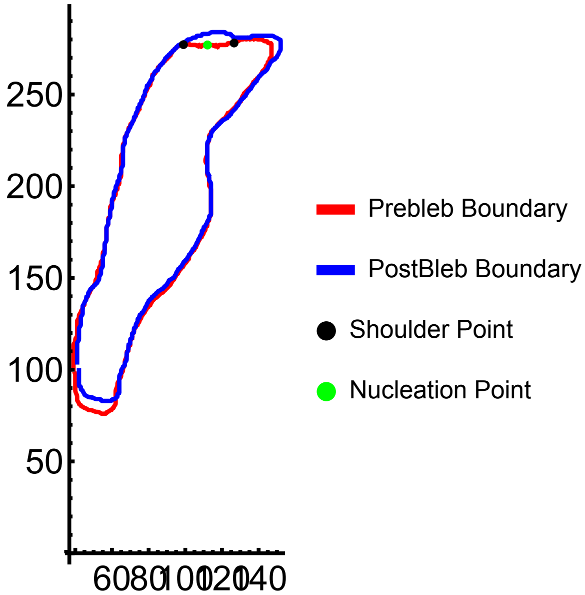

,

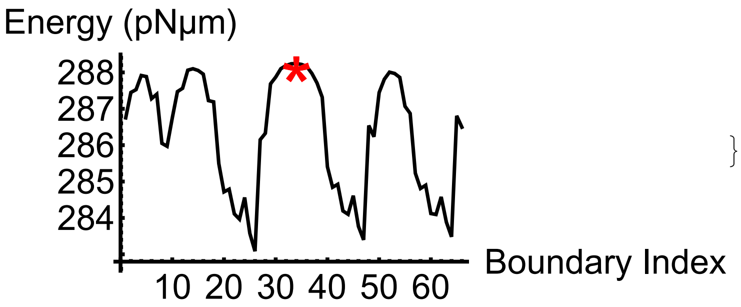

}

0615006-02

Out[ ]= {

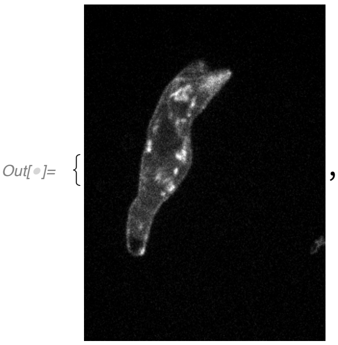

,

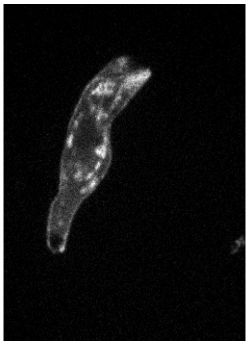

,

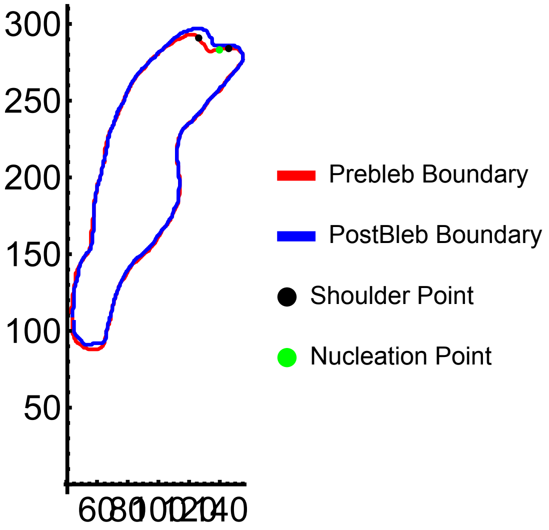

,

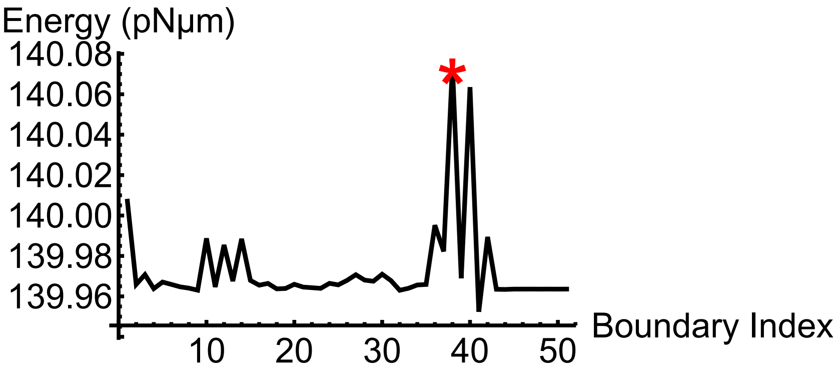

}

0615010-01

Out[•]= {

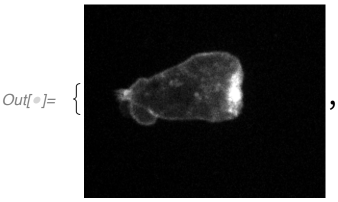

,

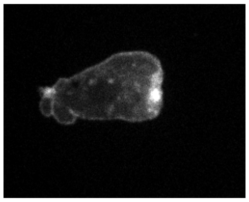

,

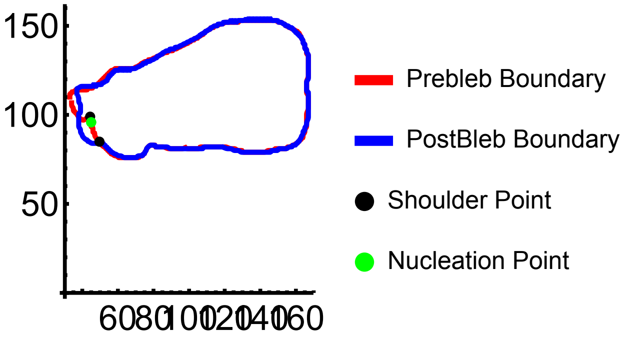

,

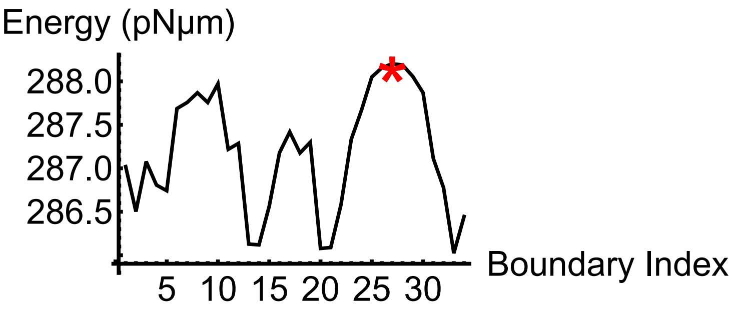

}

0615010-02

Out[•]= {

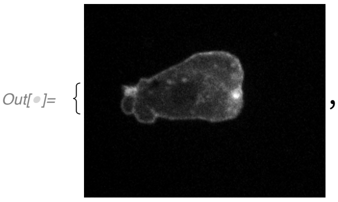

,

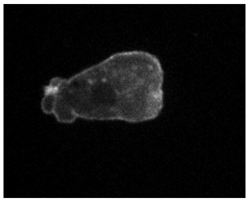

,

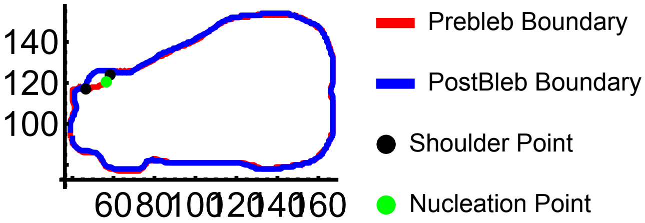

,

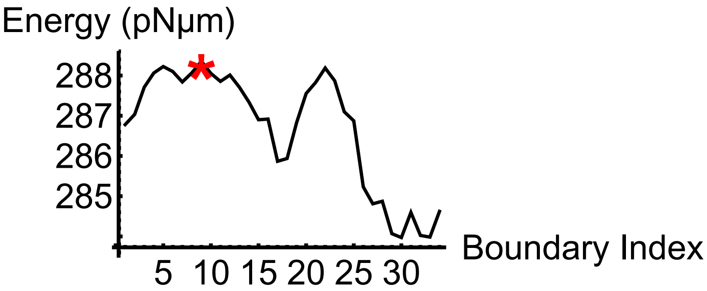

}

0615010-03

Out[●]= {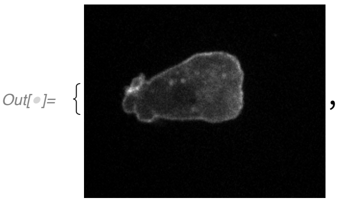, 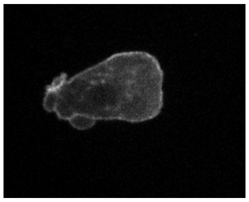,

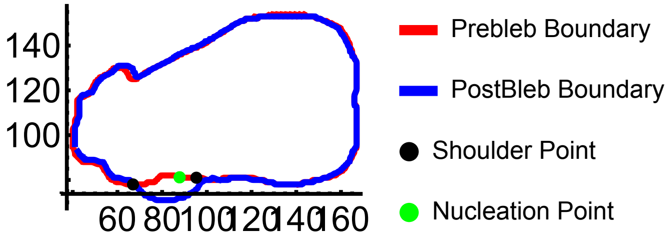

,

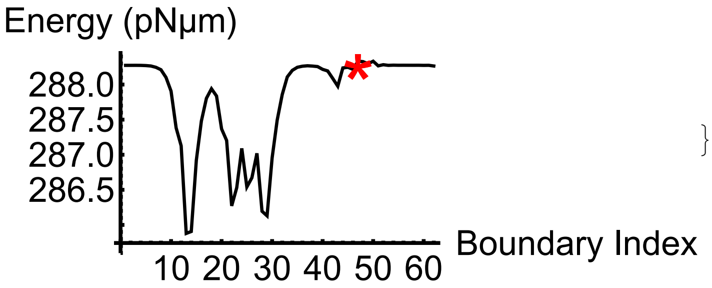

}

0615012-01

Out[]= {

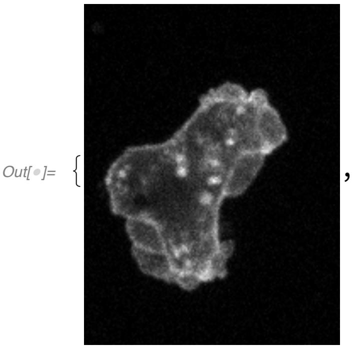

,

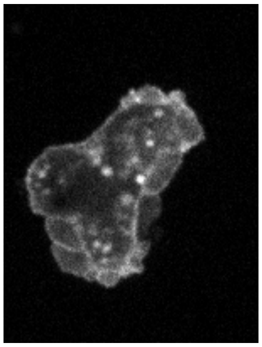

,

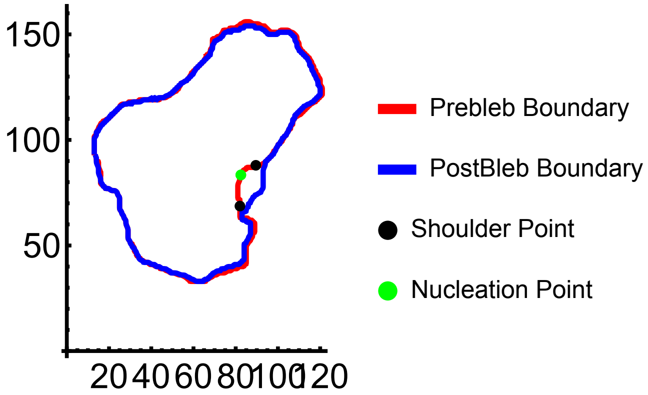

,

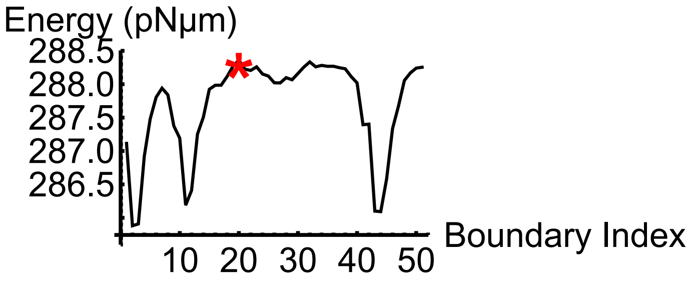

}

0615012-02

Out[•]= { 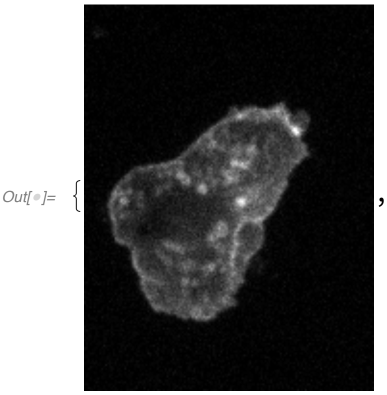 , 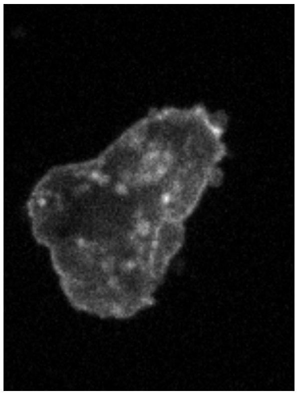 ,

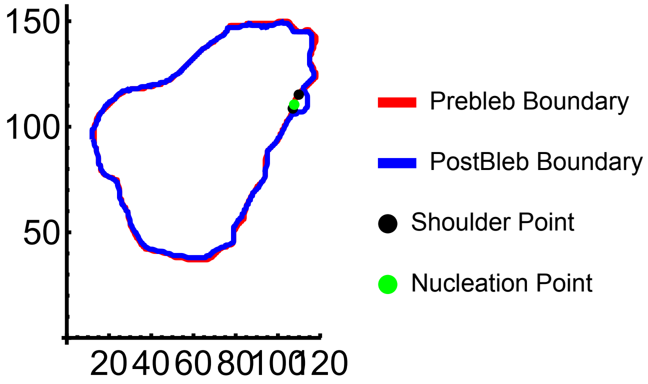

,

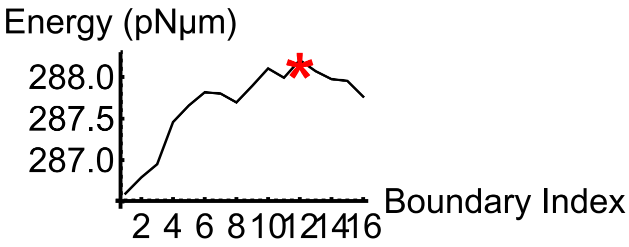

}

0615012-04

Out[ ]= {

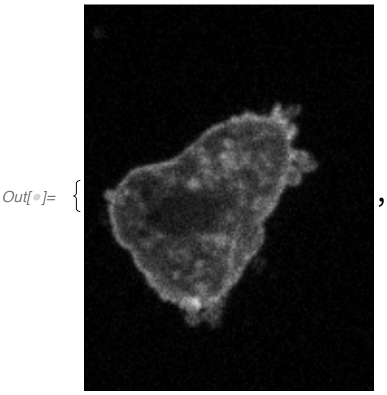

,

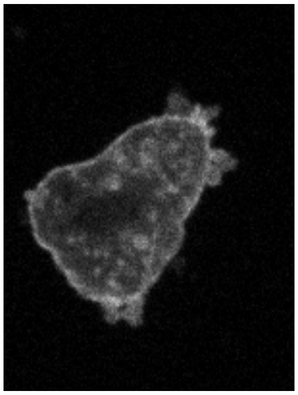

,

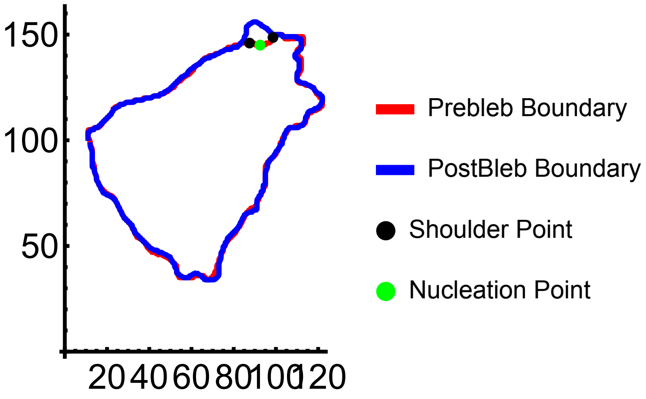

,

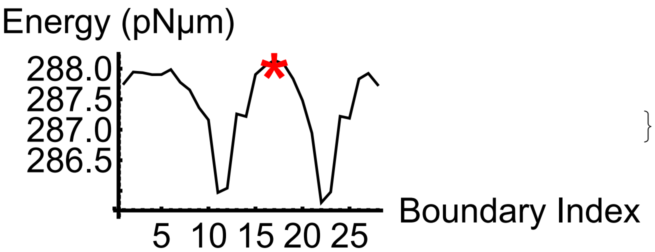

}

0615012-05

Out[•]= {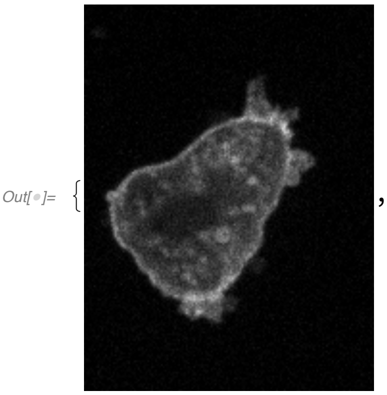, 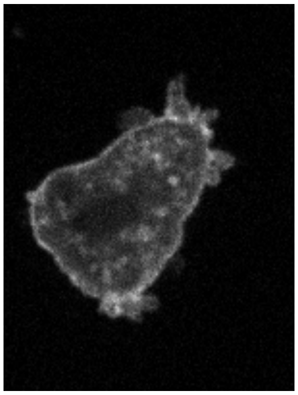,

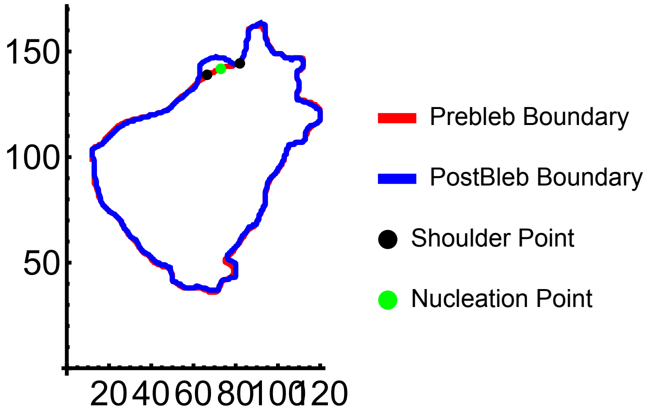

,

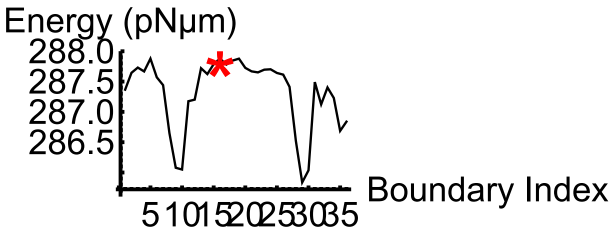

}

0615013-01

Out[ ]= {

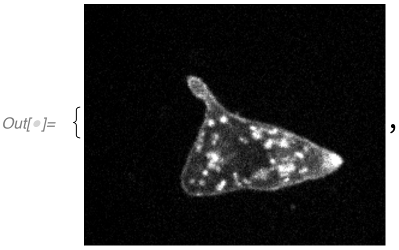

,

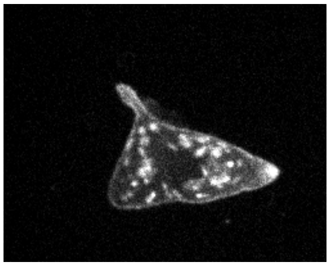

,

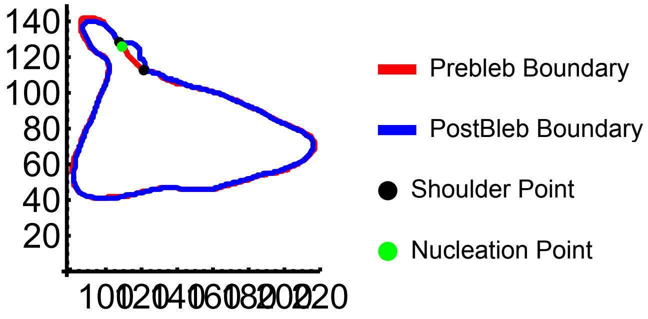

,

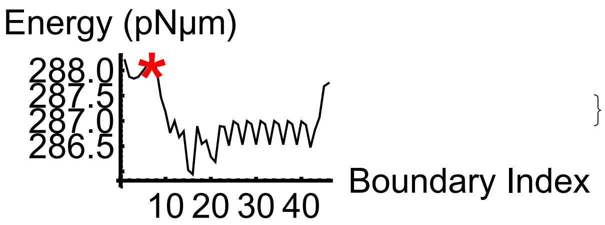

}

0615013-02

Out[•]= {

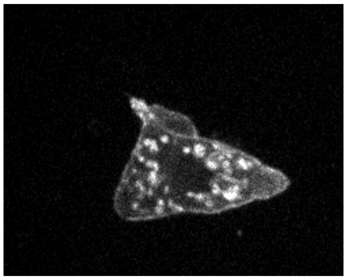

,

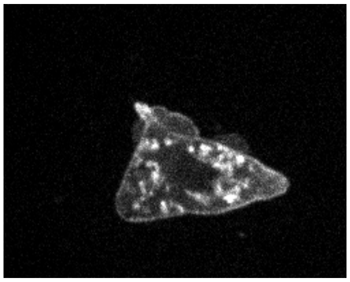

,

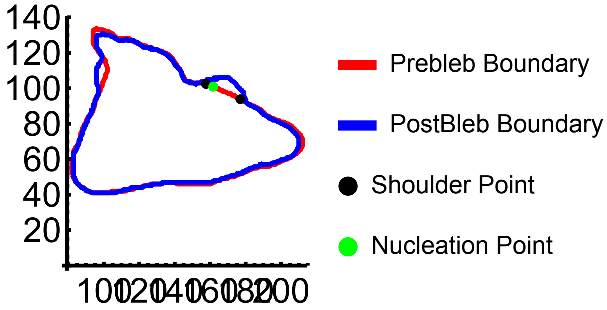

,

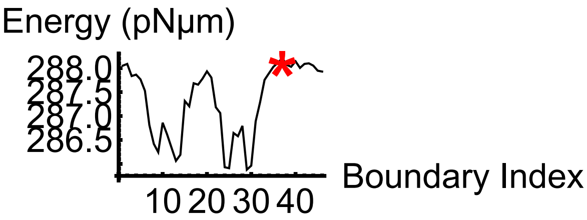

}

0615013-03

Out[•]= {

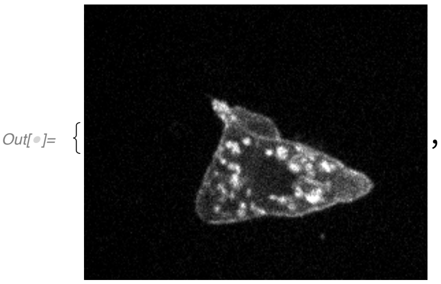

,

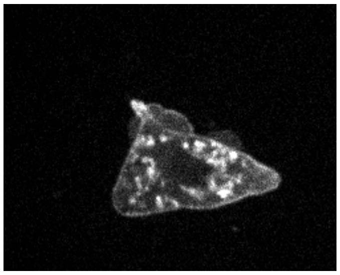

,

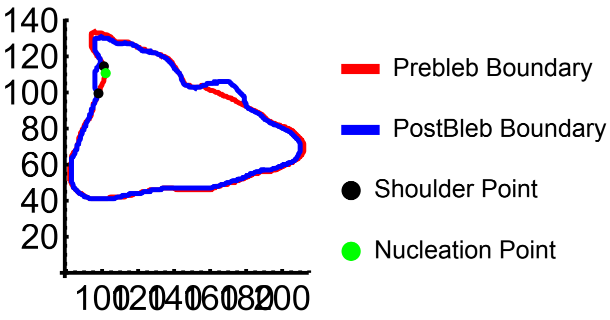

,

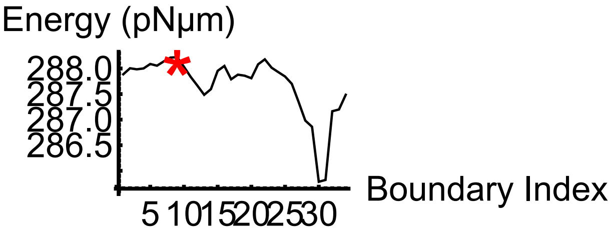

}

0615013-04

Out[ ]= {

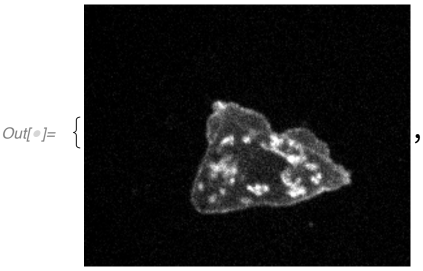

,

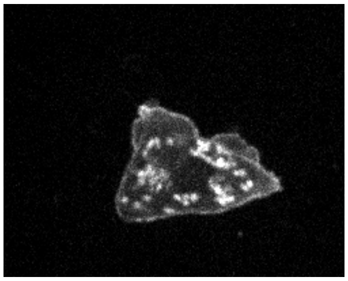

,

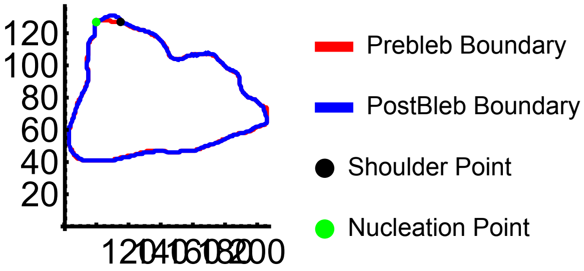

,

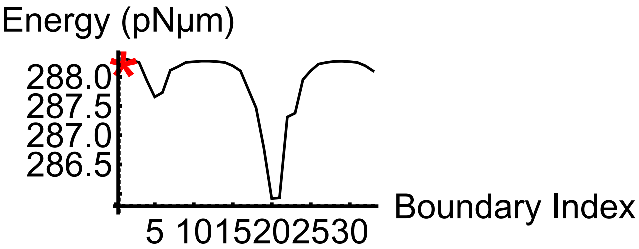

}

0615014-01

Out[●]= {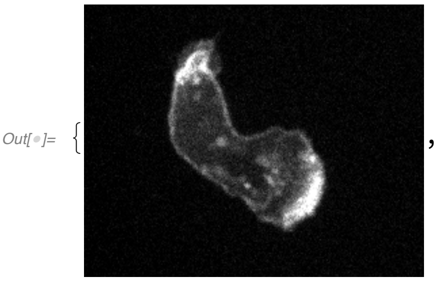, 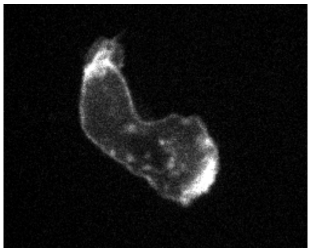,

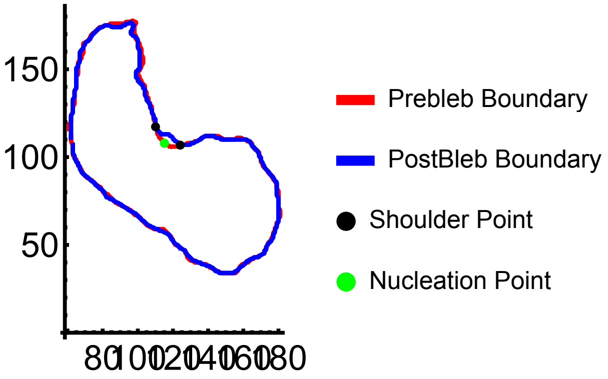

,

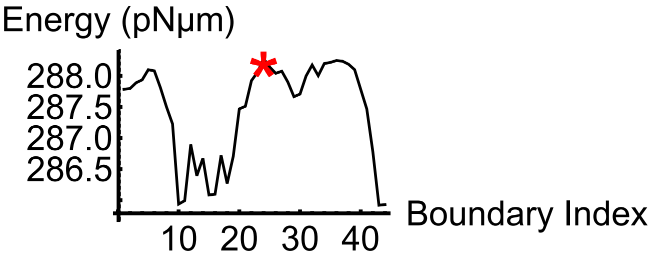

}

0615014-02

Out[•]= { 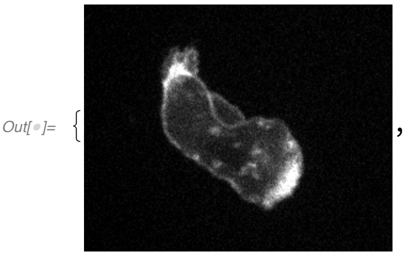 , 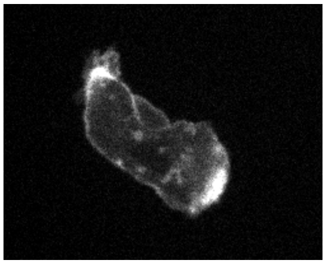 ,

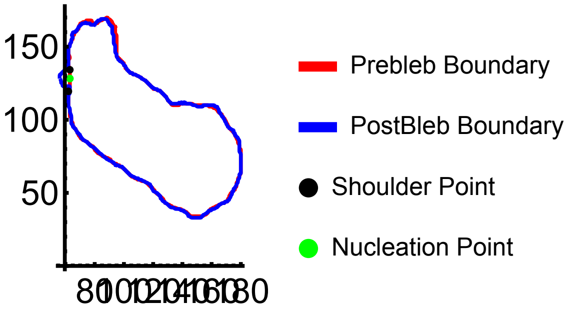

,

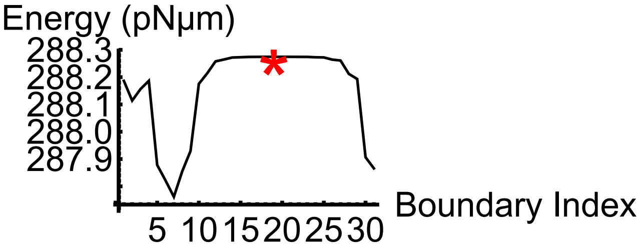

}

0615014-03

Out[ ]= { 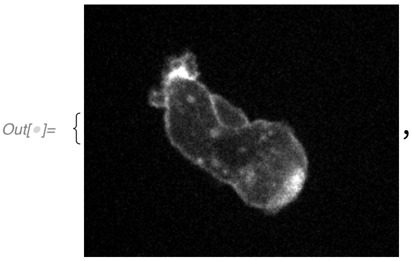 , 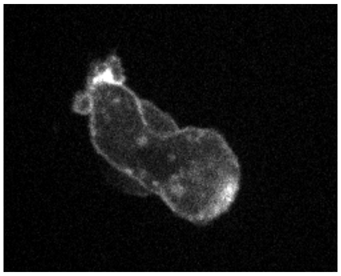 ,

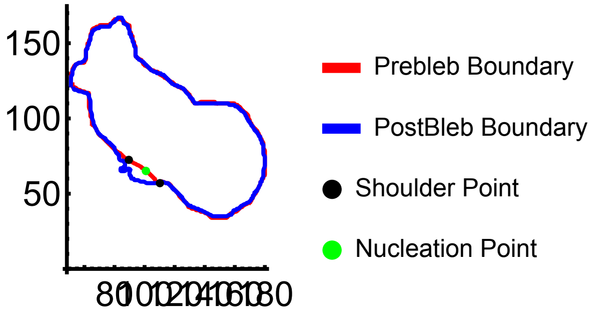

, 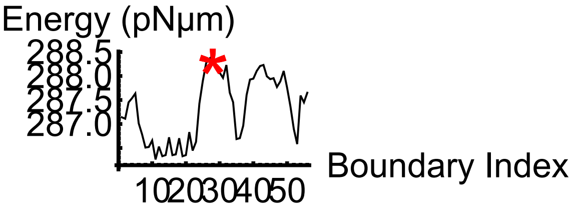

}

0615014-04

Out[ ]= {

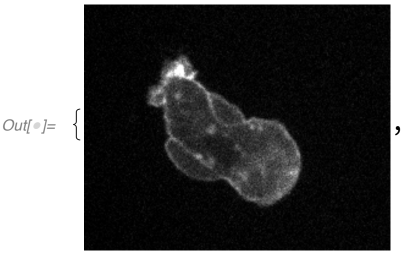

,

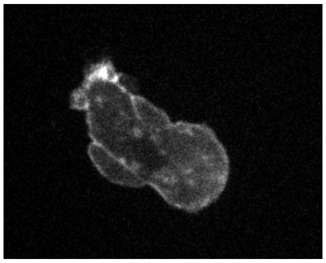

,

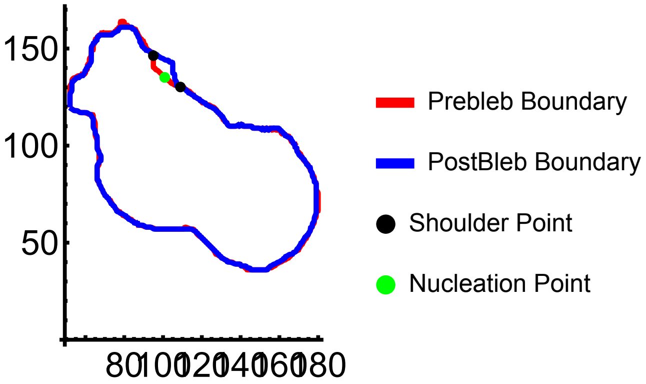

,

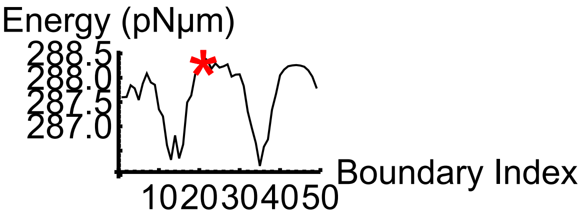

}

0615016-01

Out[ ]= {

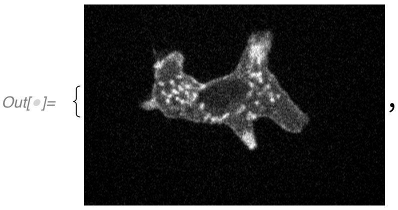

,

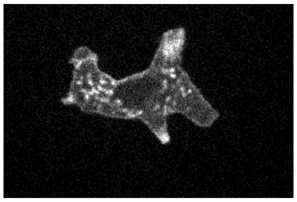

,

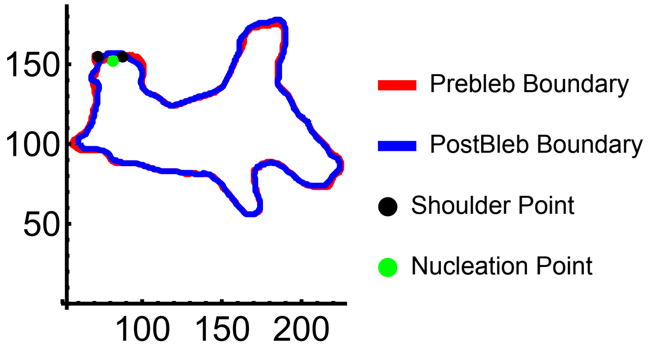

,

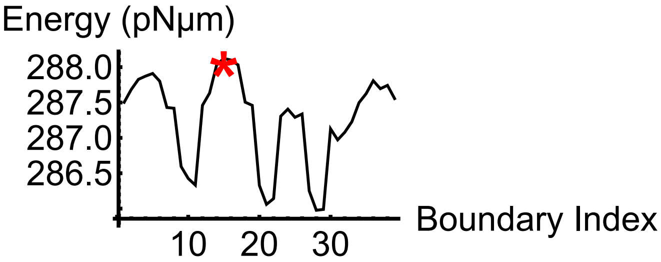

}

0615016-02

Out[ ]= {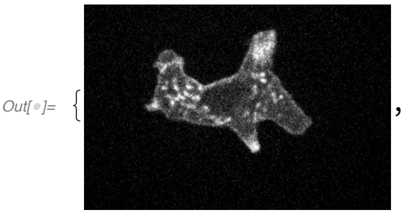, 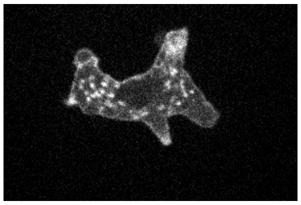, 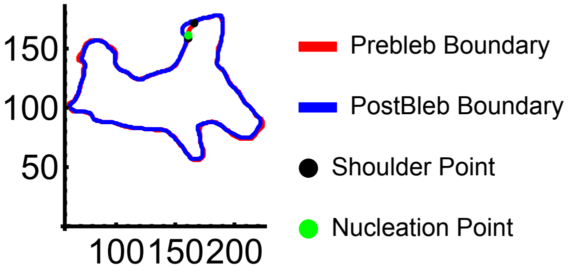

, 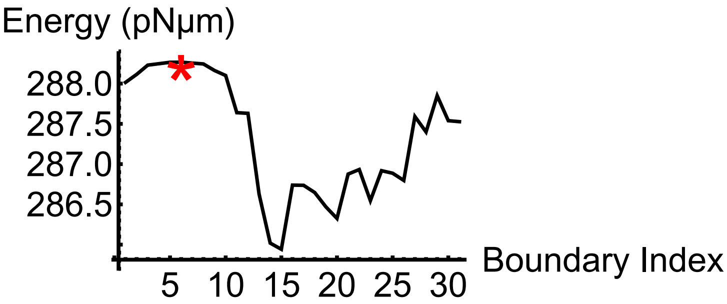

}

0615016-05

Out[•]= {

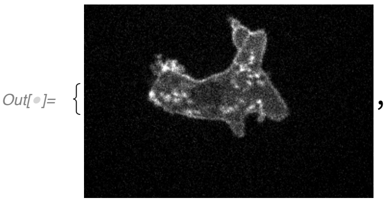

,

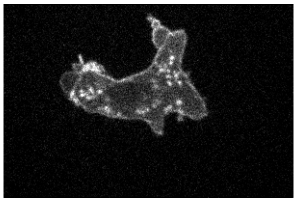

,

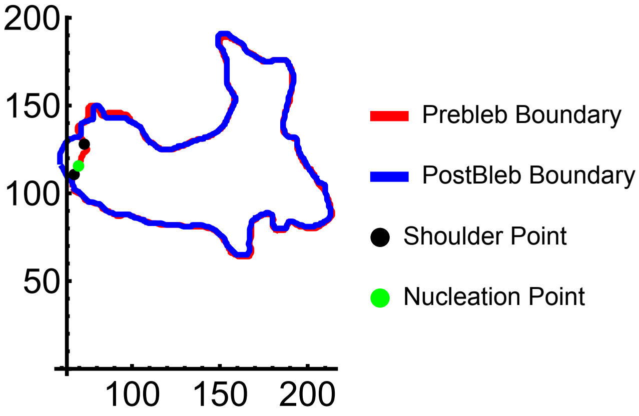

,

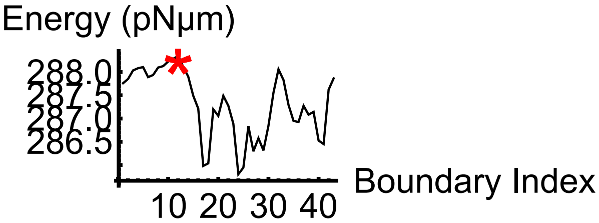

}

0615016-06

Out[ ]= {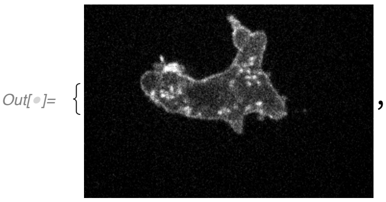, 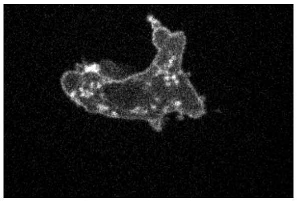,

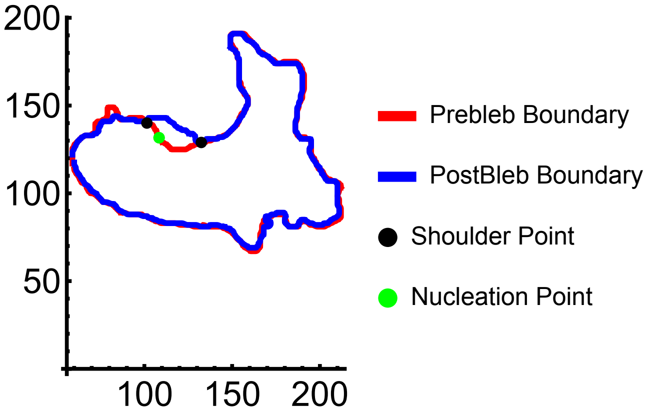

,

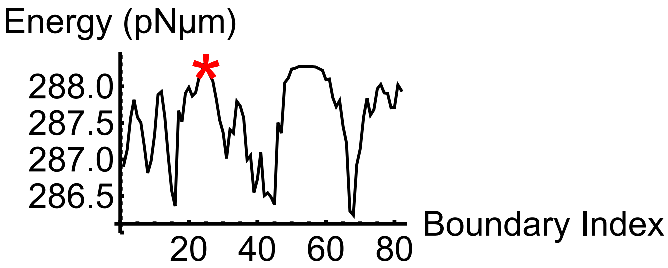

}

0615026-01

Out[ ]= {

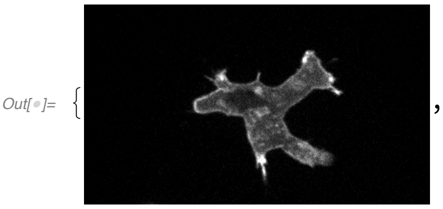

,

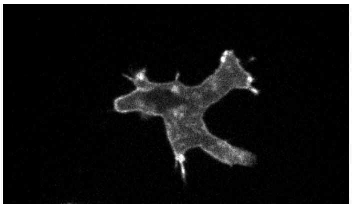

,

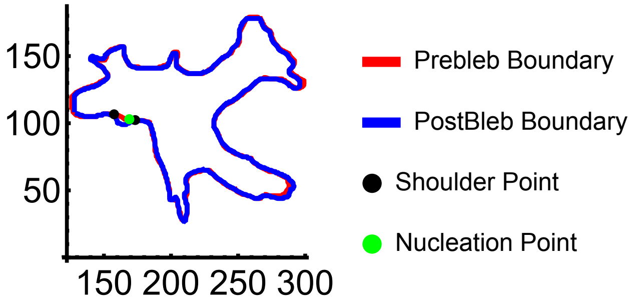

,

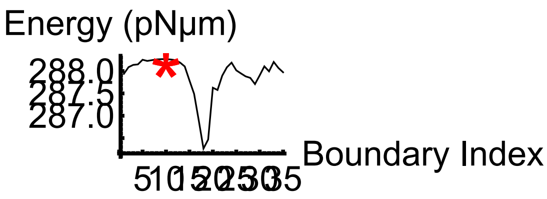

}

0615026-02

Out[ ]= { 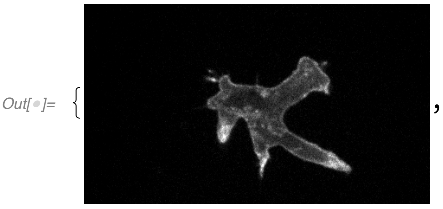 , 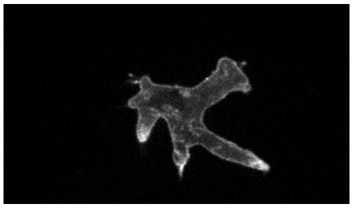 ,

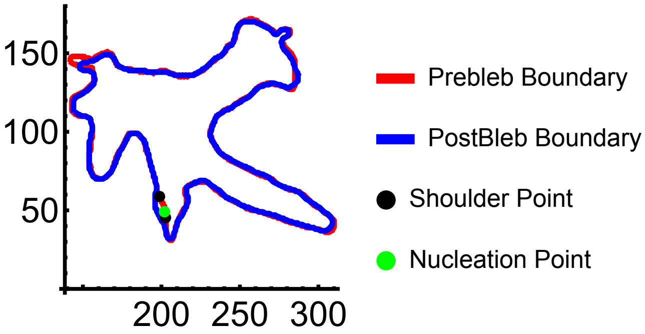

,

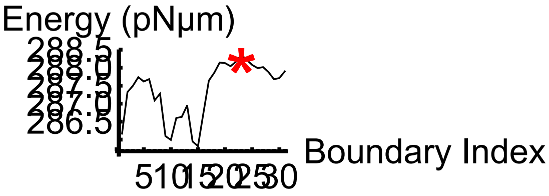

}

0615026-03

Out[ ]= {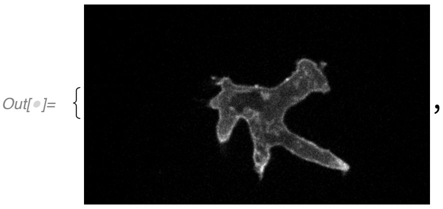, 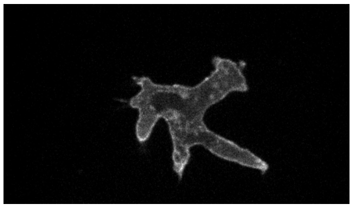,

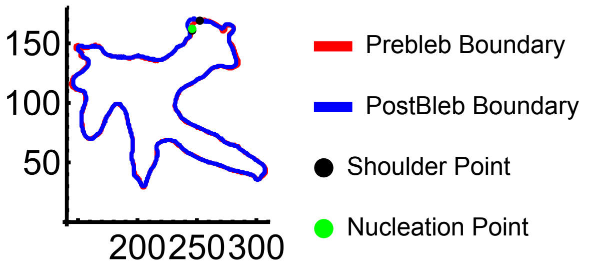

,

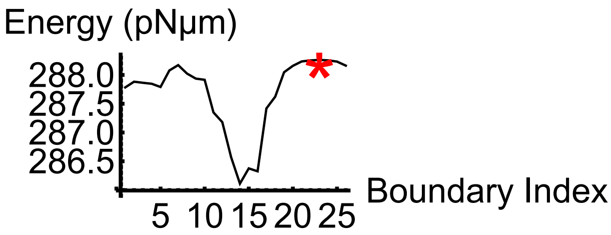

}

0615026-04

Out[ ]= {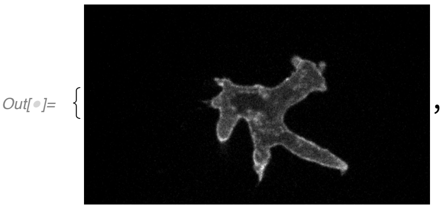, 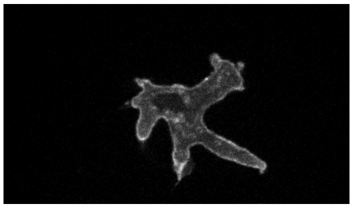,

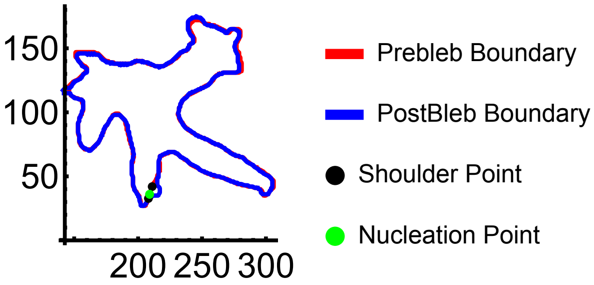

,

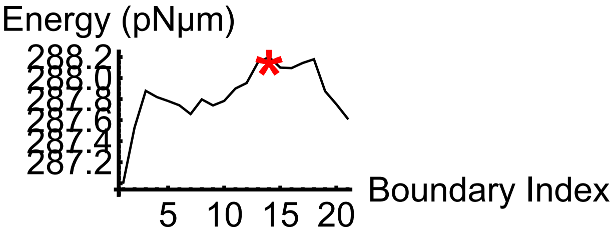

}

0615026-06

Out[ ]= {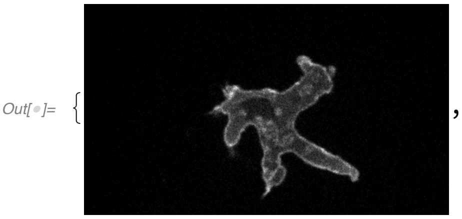, 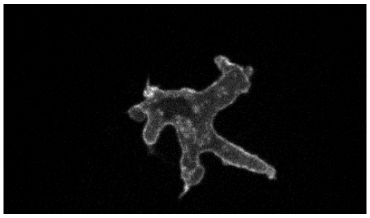,

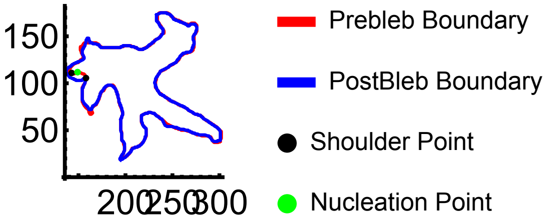

,

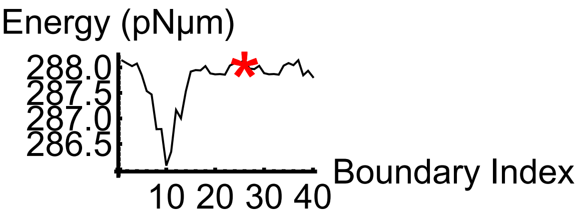

}

0615026-07

{ 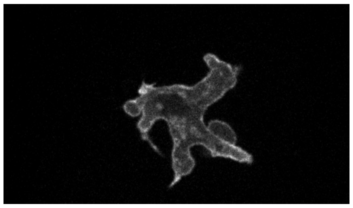 , 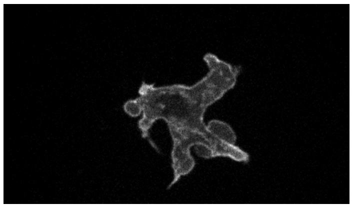 ,

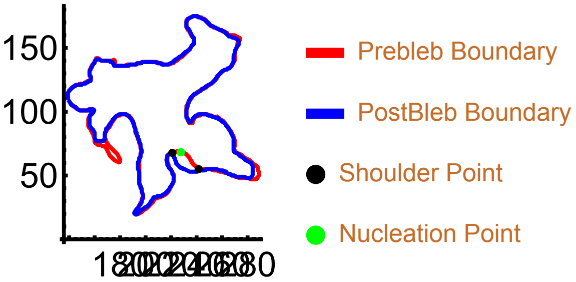
150
100
50
Prebleb Boundary
PostBleb Boundary
Shoulder Point
Nucleation Point

, 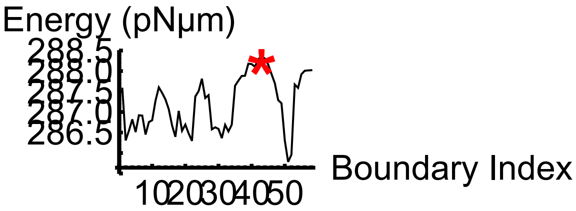
Energy (pNμm)
Boundary Index

}

Bleb Nucleation Predictions for Experiment 1204

1204601-01

Out[ ]= {

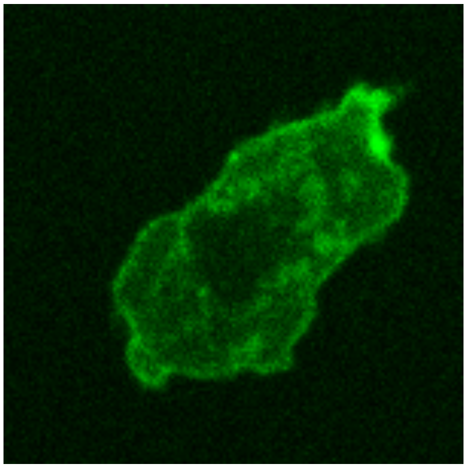

,

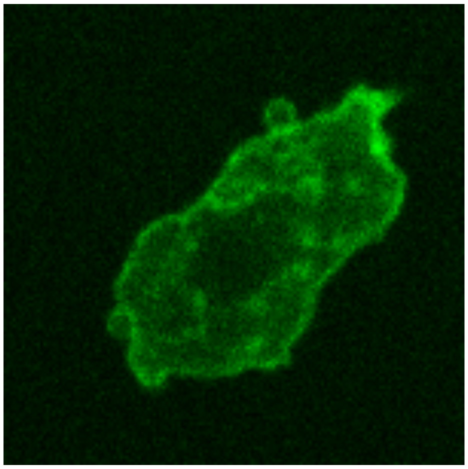

,

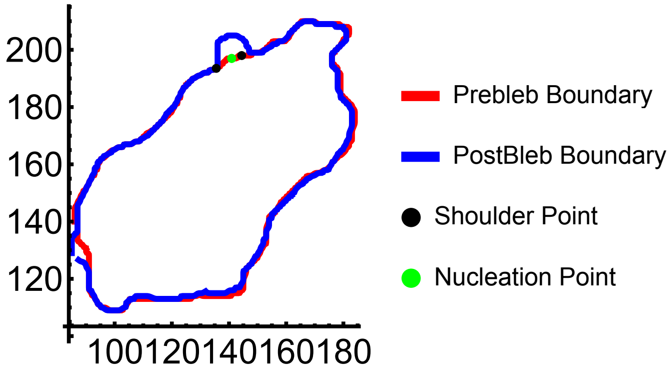

,

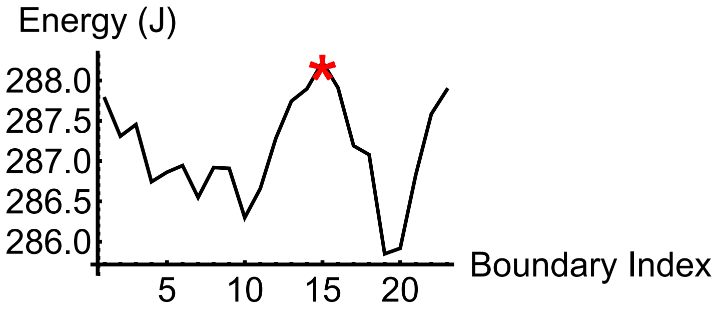

}

1204601-03

Out[•]= {

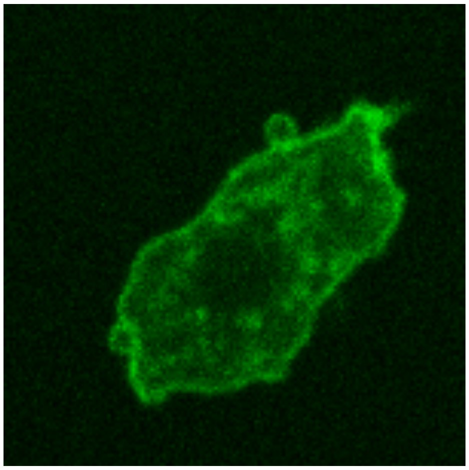

,

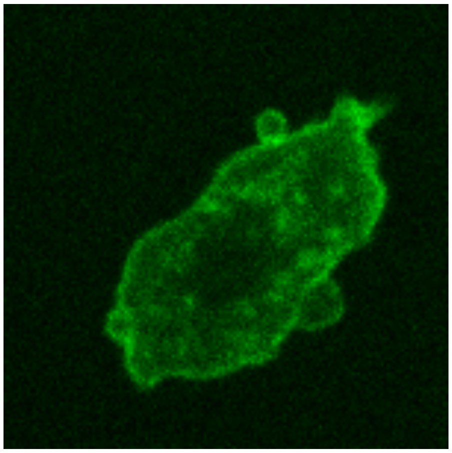

,

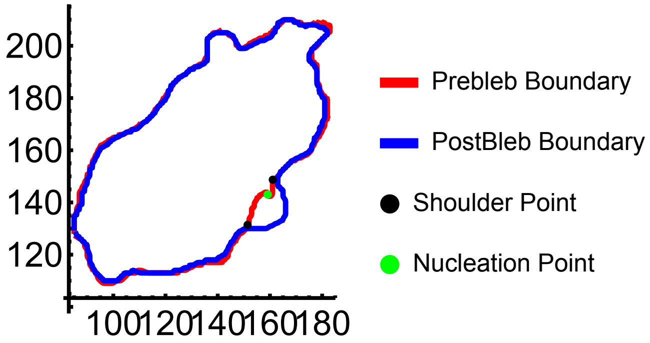

,

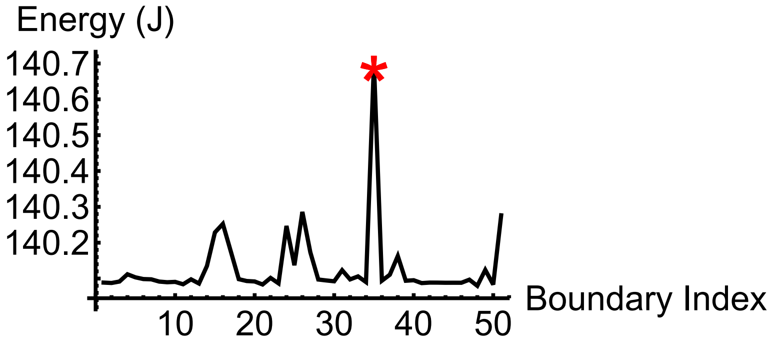

}

1204602-03

Out[•]= {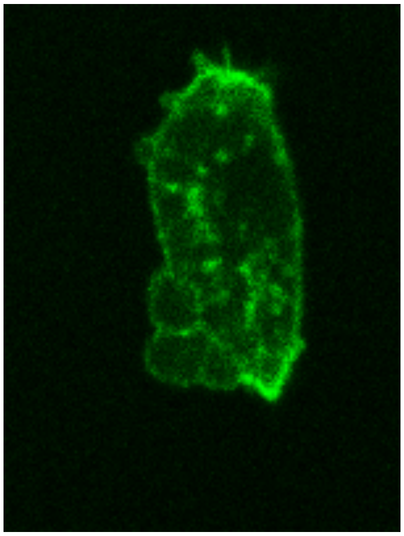,

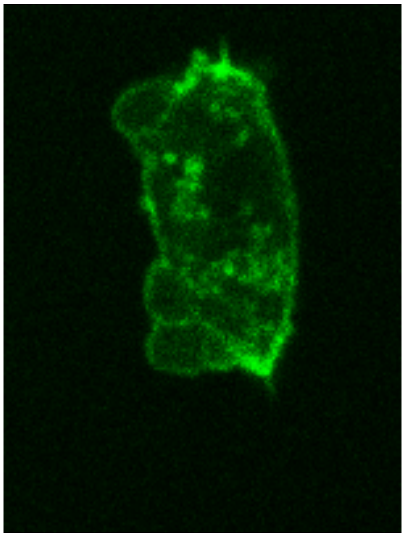, 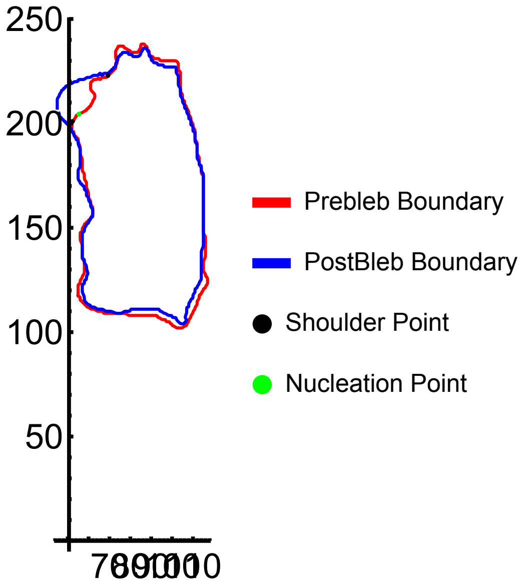
250
200
150
100
50
Prebleb Boundary
PostBleb Boundary
Shoulder Point
Nucleation Point
,

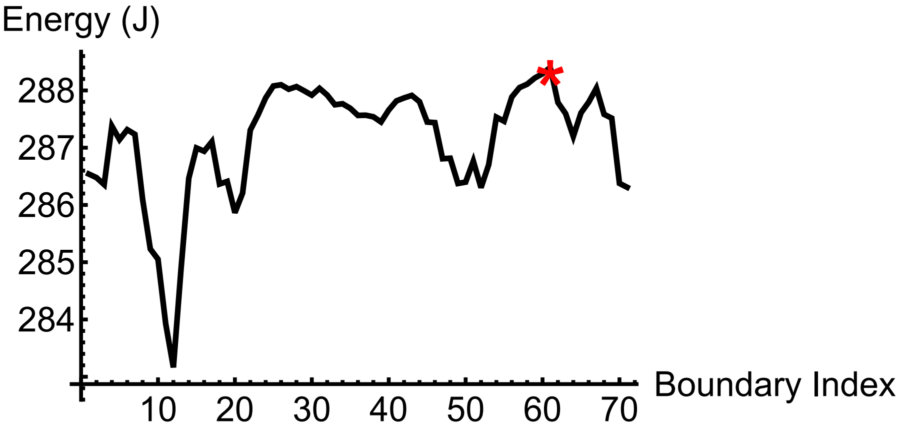
Energy (J)
288
287
286
285
284
10
20
30
40
50
60
70
Boundary Index
}

1204602-04

Out[∘]= {

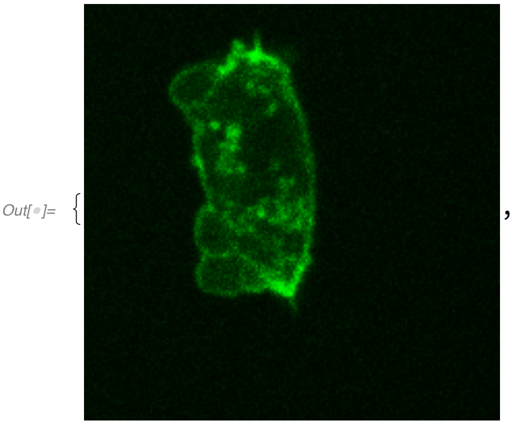

,

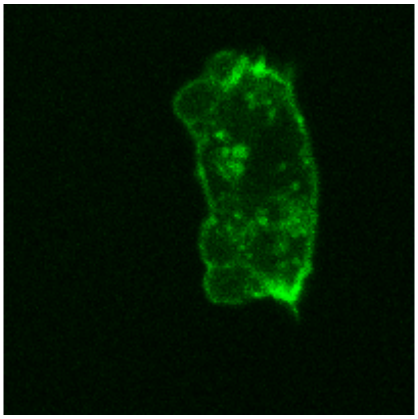

,

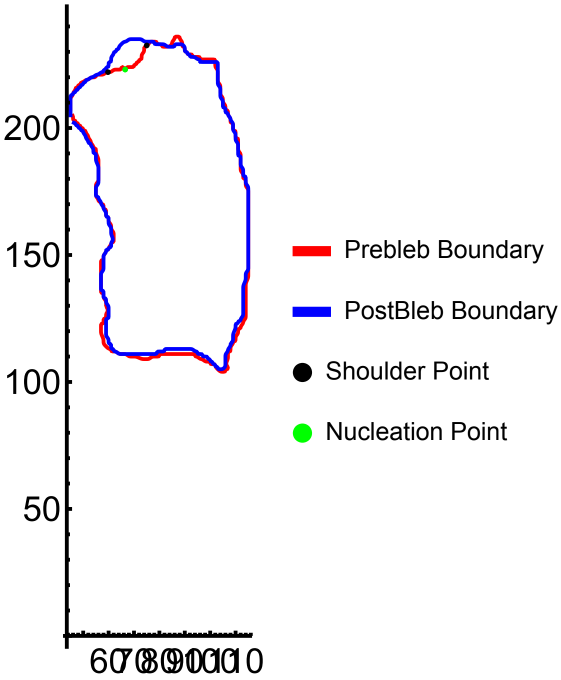
200
150
100
50
60708090100110
Prebleb Boundary
PostBleb Boundary
Shoulder Point
Nucleation Point

,

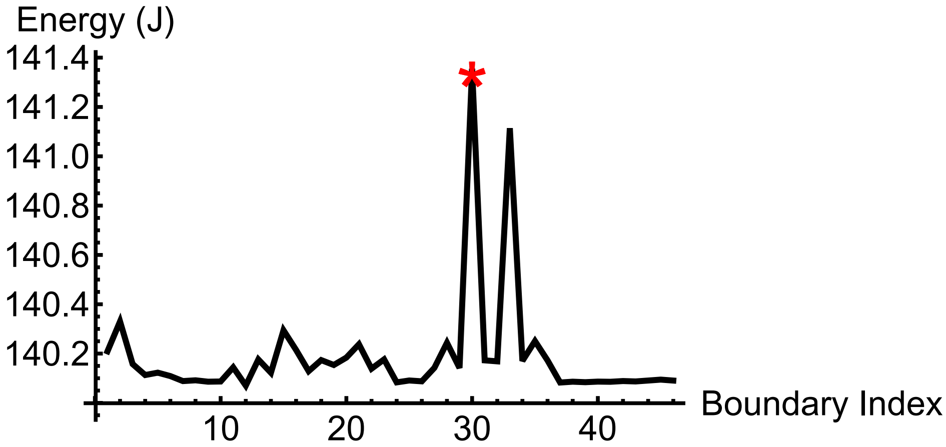
Energy (J)
141.4
141.2
141.0
140.8
140.6
140.4
140.2
10
20
30
40
Boundary Index

}

1204602-05

Out[•]= {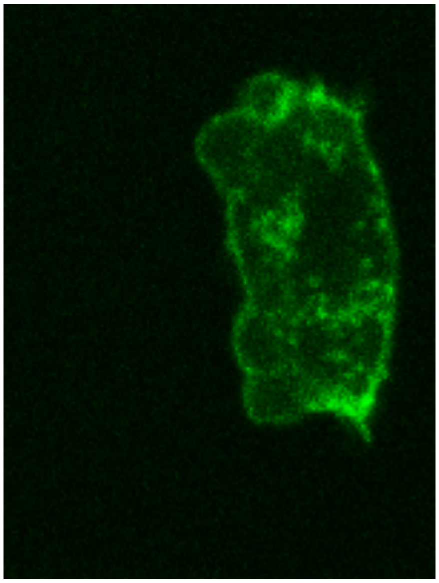,

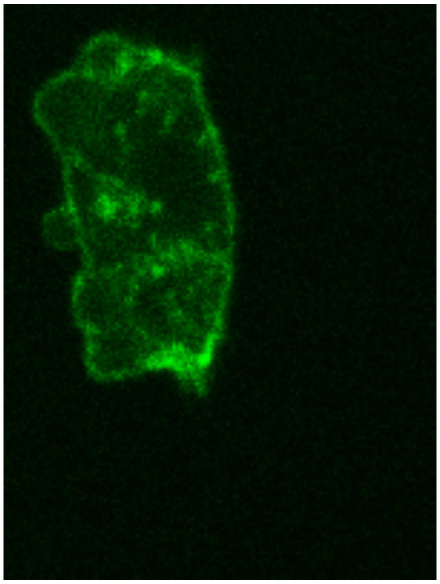, 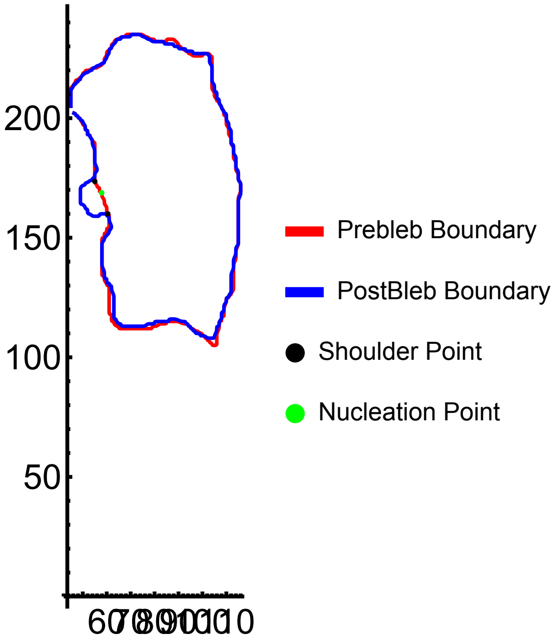

,

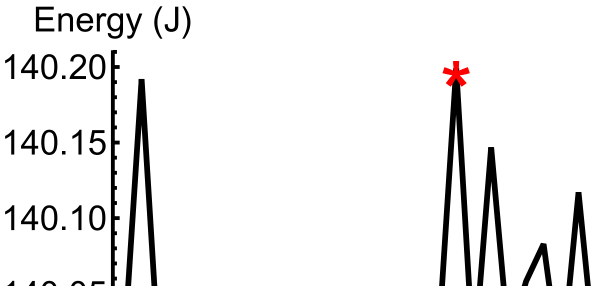

}

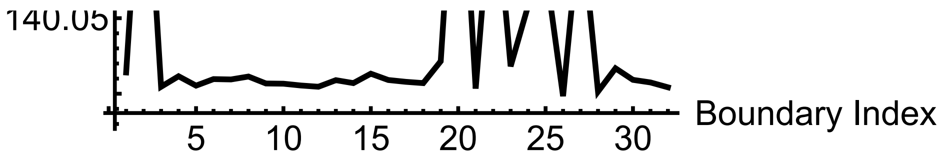

1204602-08

Out[•]= {

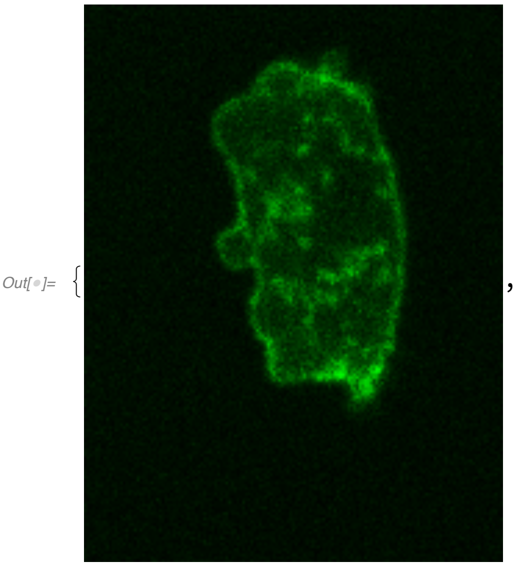

,

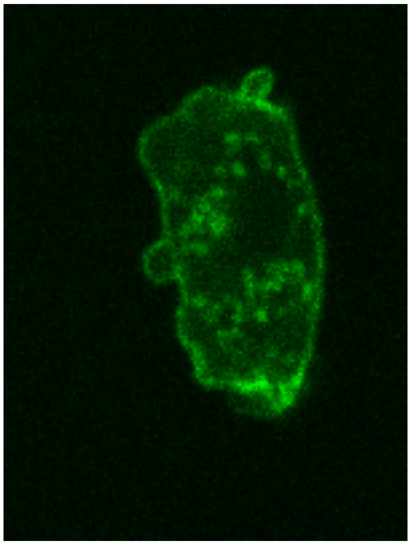

,

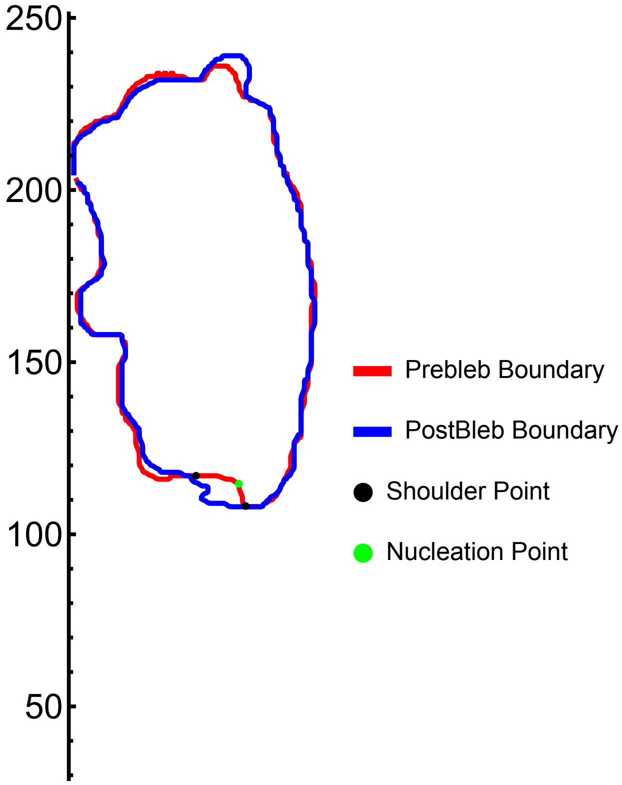

,

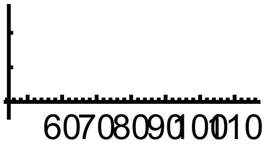

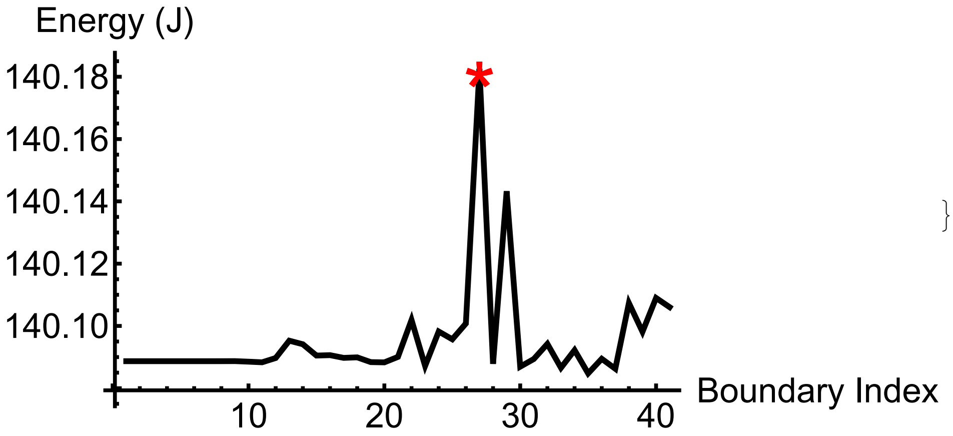

}

1204602-10

Out[]= {

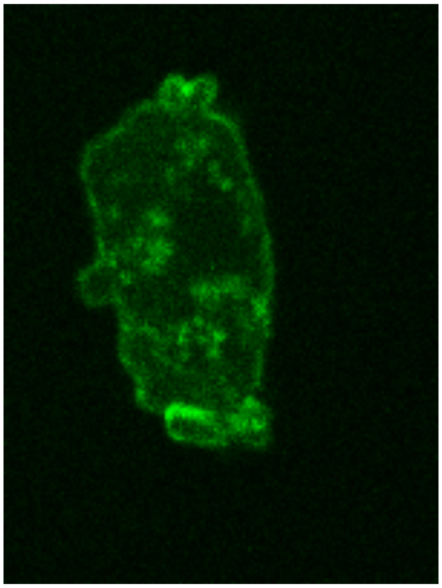

,

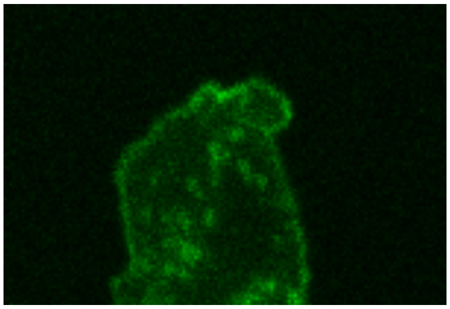

,

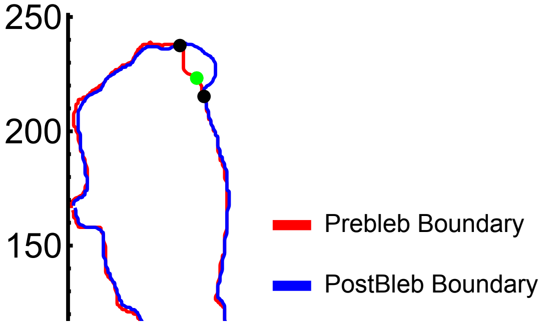

,

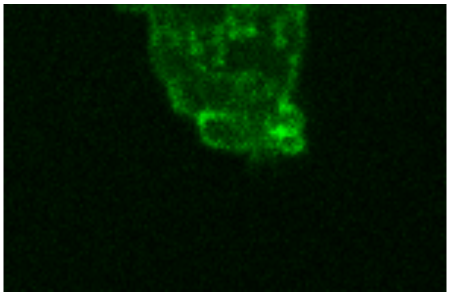

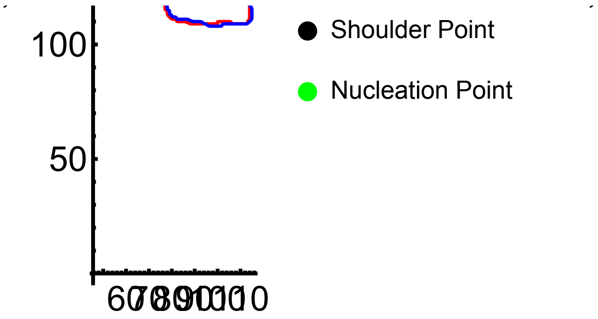
100
50
Shoulder Point
Nucleation Point

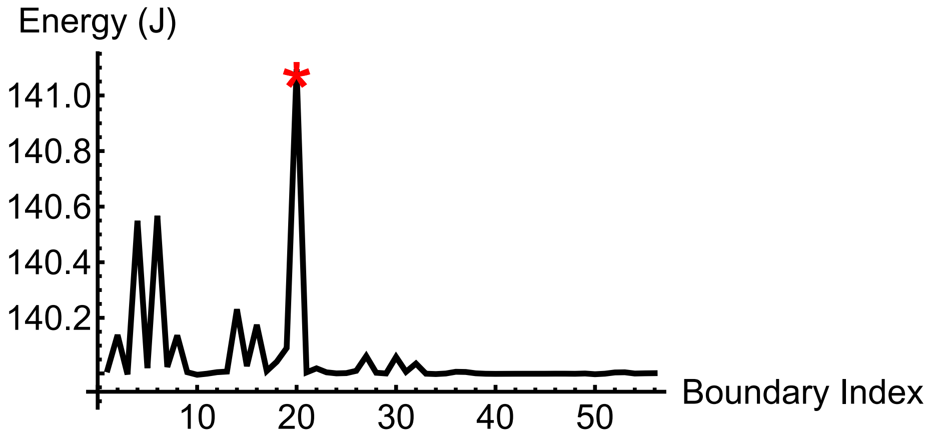
Energy (J)
141.0
140.8
140.6
140.4
140.2
10
20
30
40
50
Boundary Index

}

1204709-01

Out[ ]= {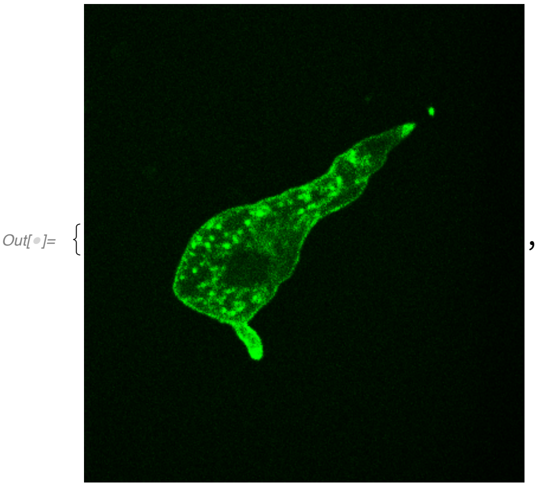, 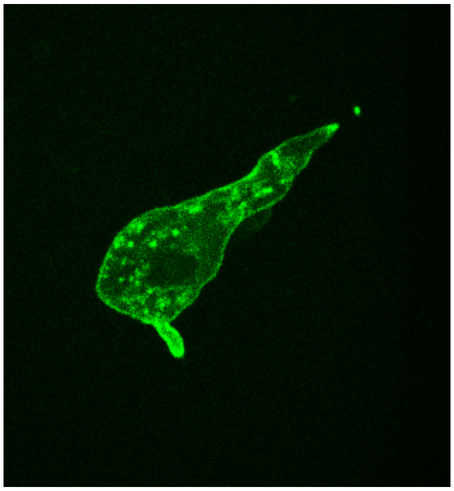,

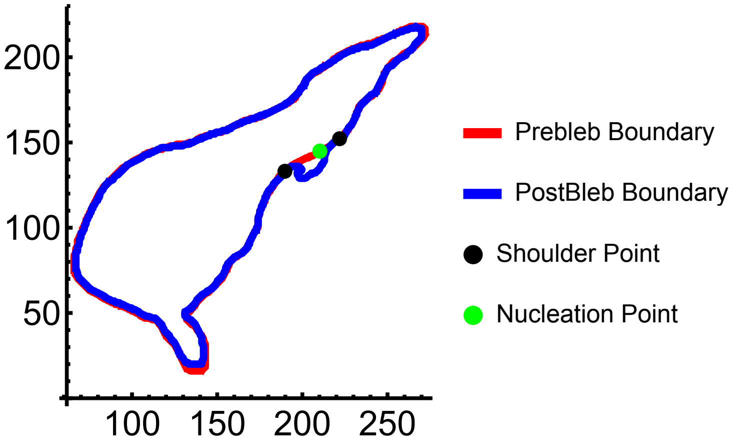
200
150
100
50
100
150
200
250
Prebleb Boundary
PostBleb Boundary
Shoulder Point
Nucleation Point
,

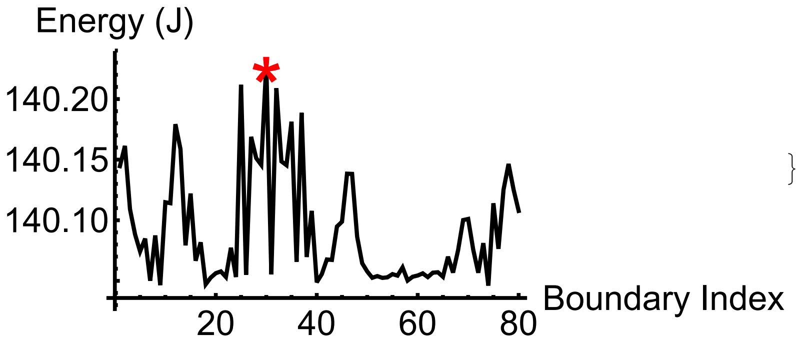
Energy (J)
140.20
140.15
140.10
20
40
60
80
Boundary Index
}

1204709-02

Out[•]= {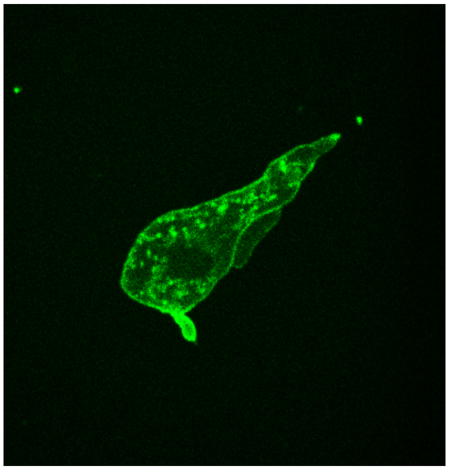, 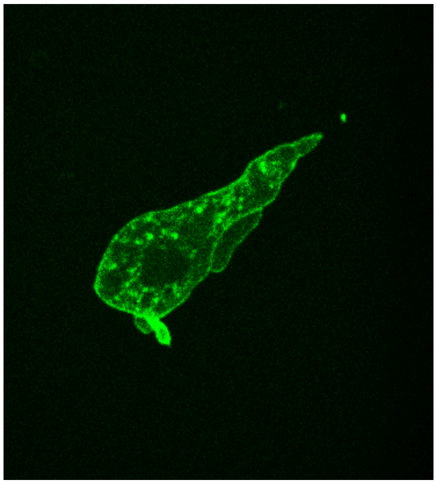,

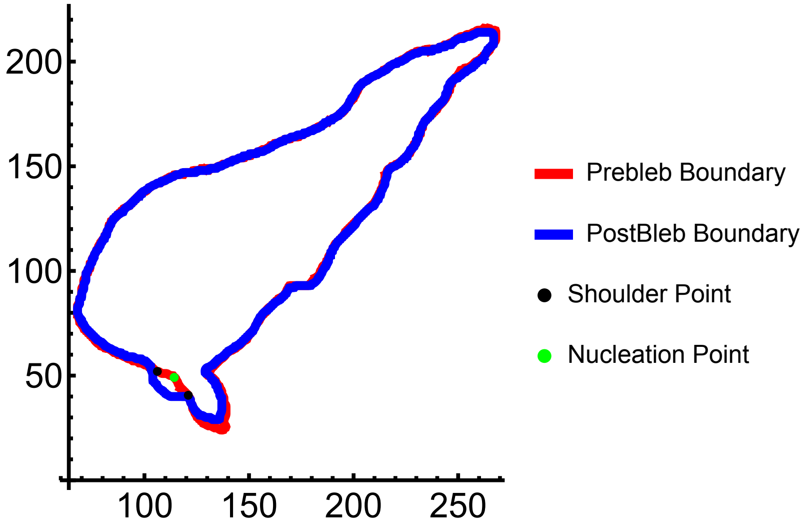

,

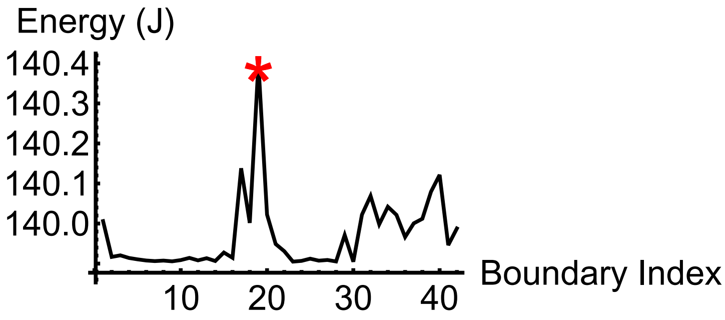

}

1204709-03

Out[•]= {

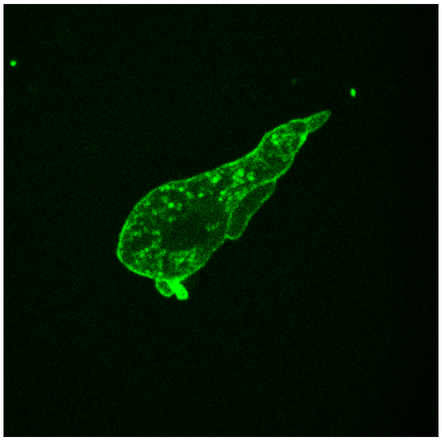

,

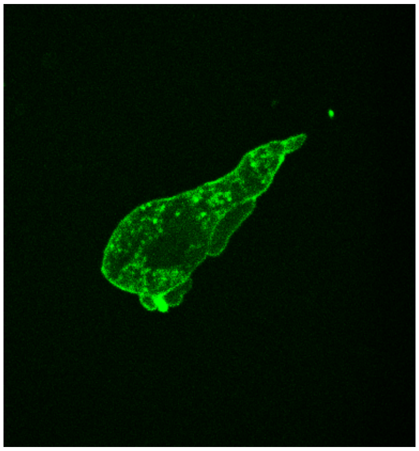

,

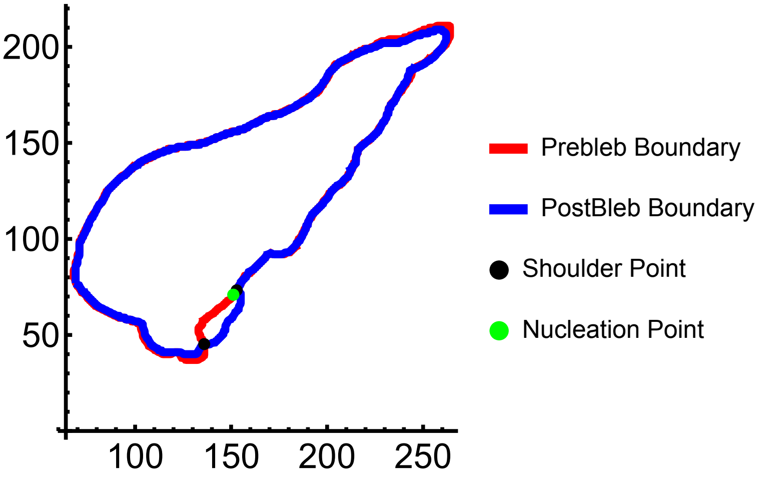

,

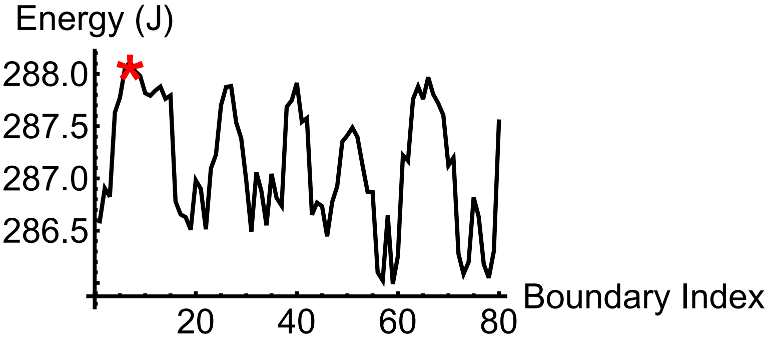

}

1204709-04

Out[ ]= {

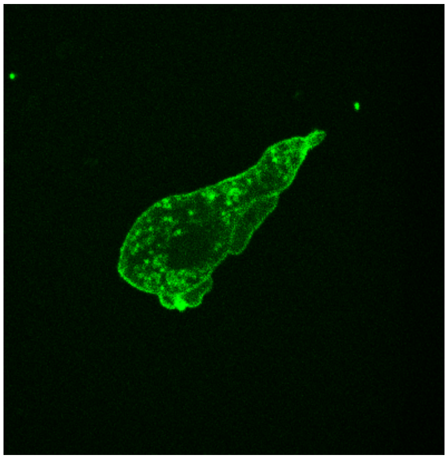

,

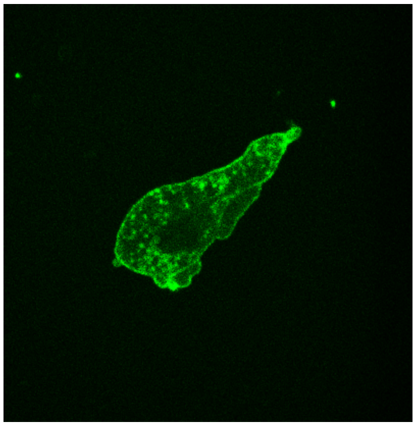

,

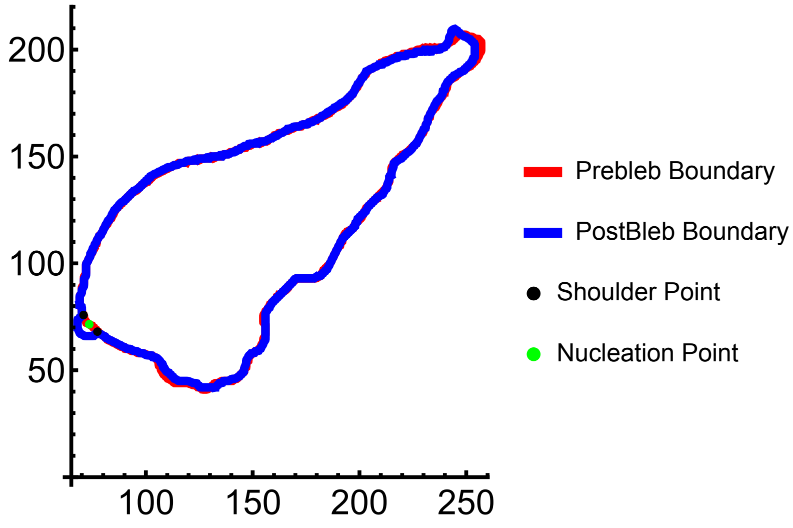

,

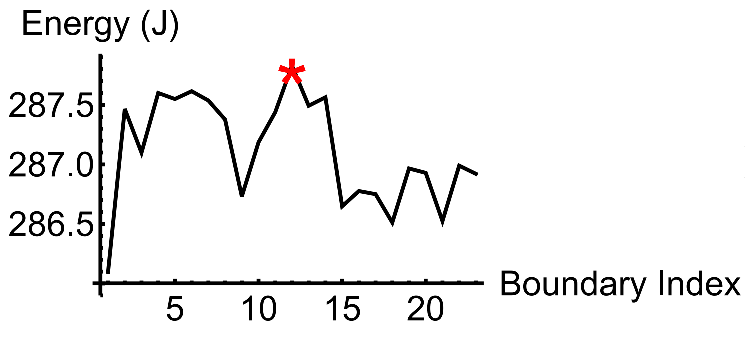

}

1204710-01

Out[•]= {

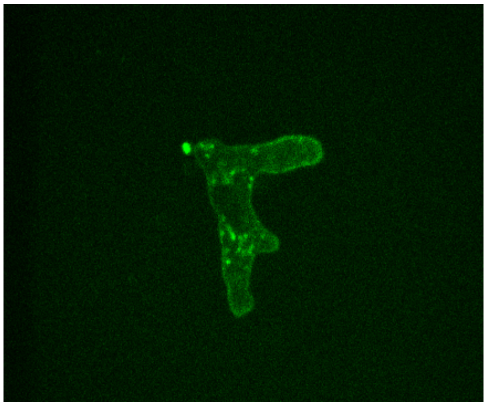

,

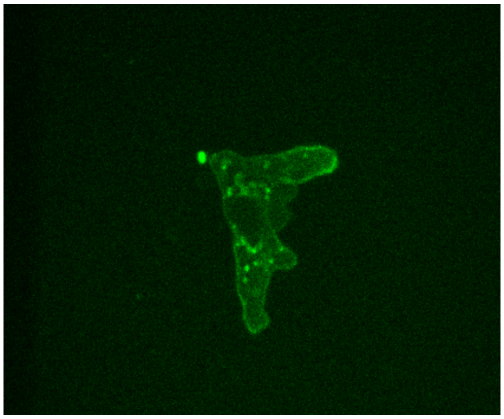

,

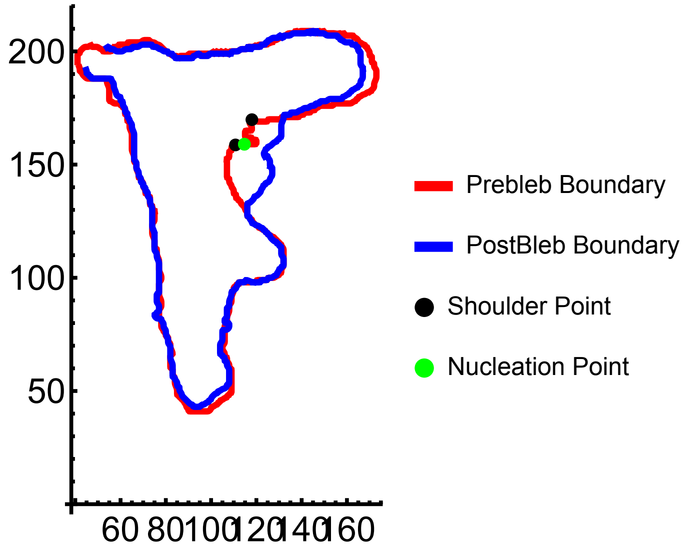

,

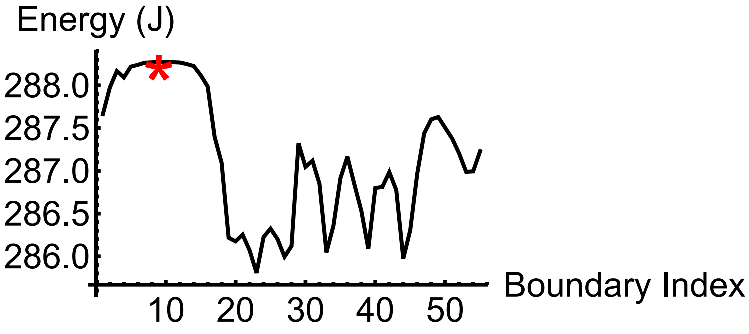

}

1204710-02

Out[ ]= {
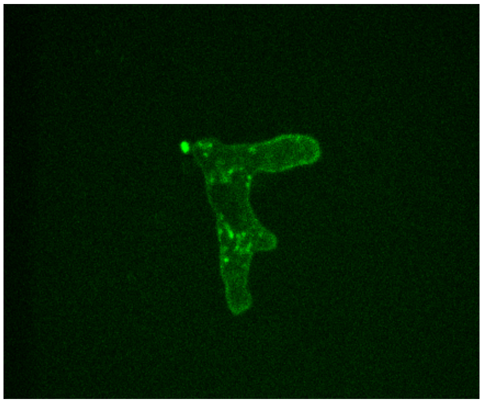
,
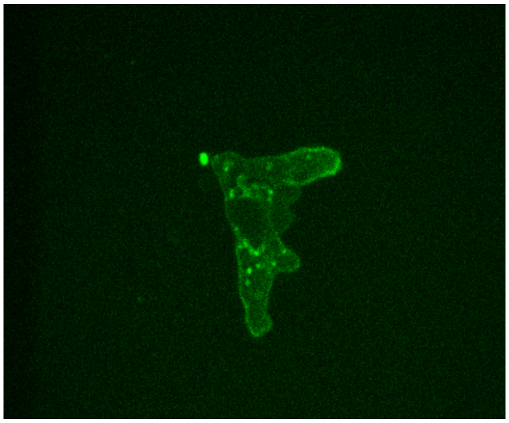
,
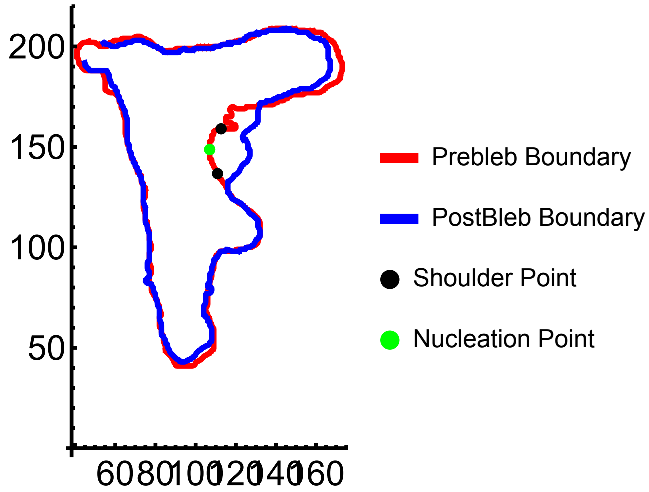

,
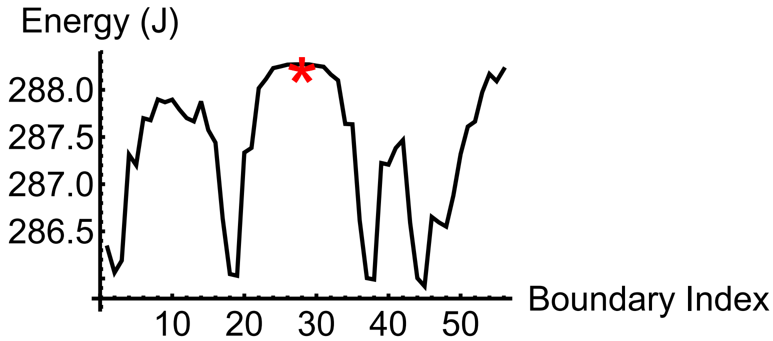

}

1204710-05

Out[●]= {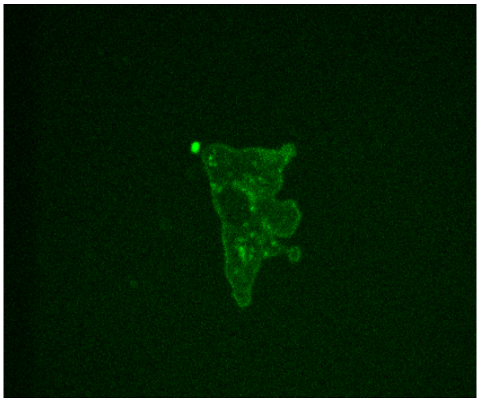,

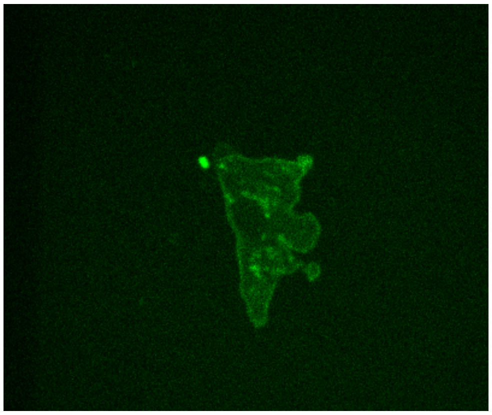, 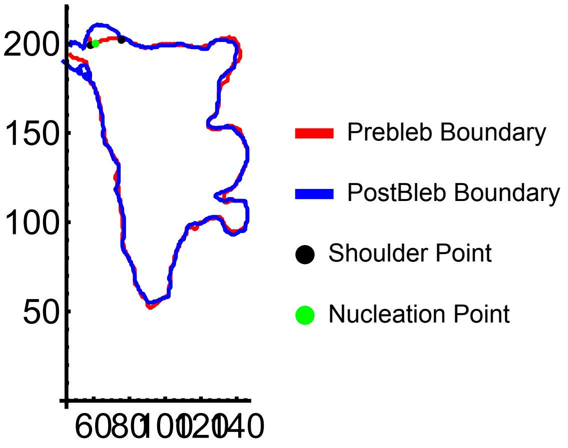

,

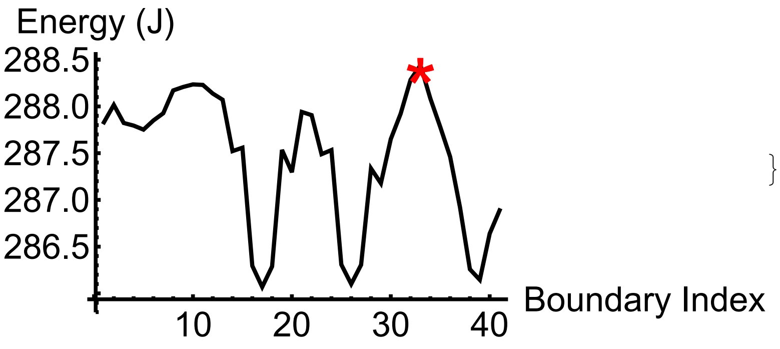

}

1204710-06

Out[]= {

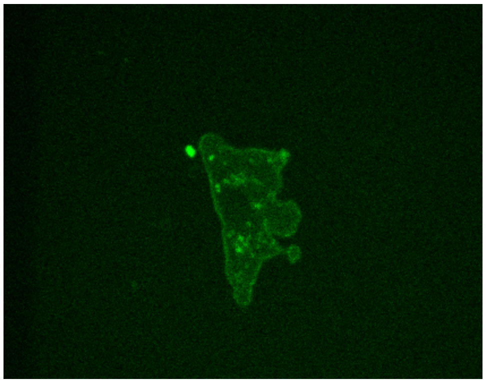

,

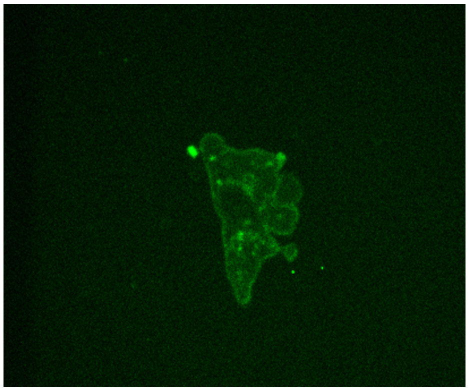

,

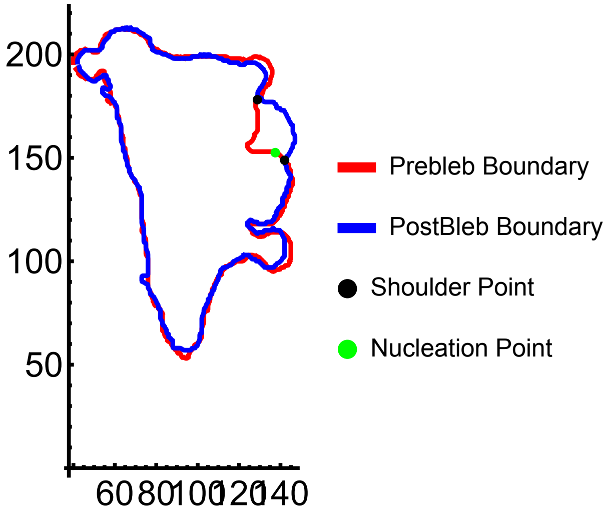

,

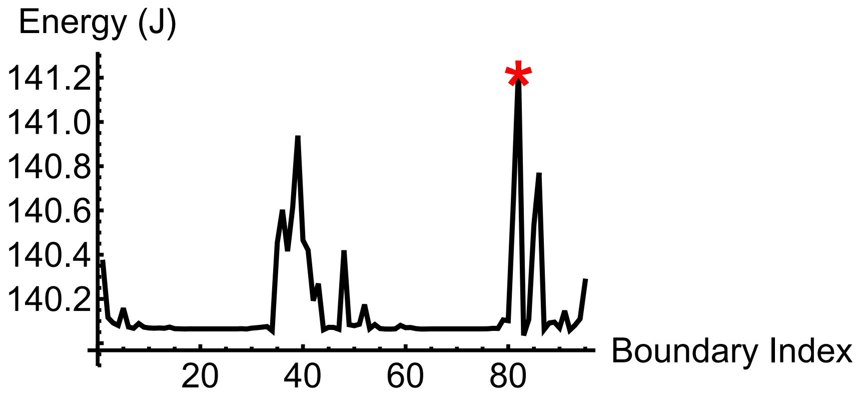

}
